# Electrochemical flow aziridination of unactivated alkenes

**DOI:** 10.1093/nsr/nwad187

**Published:** 2023-07-03

**Authors:** Shengchun Wang, Pengjie Wang, Shu-Jin Li, Yi-Hung Chen, Zhi-Jun Sun, Aiwen Lei

**Affiliations:** Institute for Advanced Studies (IAS), College of Chemistry and Molecular Sciences, Wuhan University, Wuhan 430079, China; Institute for Advanced Studies (IAS), College of Chemistry and Molecular Sciences, Wuhan University, Wuhan 430079, China; State Key Laboratory of Oral & Maxillofacial Reconstruction and Regeneration, Key Laboratory of Oral Biomedicine Ministry of Education, Hubei Key Laboratory of Stomatology, School & Hospital of Stomatology, Wuhan University, Wuhan 430079, China; Institute for Advanced Studies (IAS), College of Chemistry and Molecular Sciences, Wuhan University, Wuhan 430079, China; State Key Laboratory of Oral & Maxillofacial Reconstruction and Regeneration, Key Laboratory of Oral Biomedicine Ministry of Education, Hubei Key Laboratory of Stomatology, School & Hospital of Stomatology, Wuhan University, Wuhan 430079, China; Department of Oral Maxillofacial-Head Neck Oncology, School and Hospital of Stomatology, Wuhan University, Wuhan 430079, China; Institute for Advanced Studies (IAS), College of Chemistry and Molecular Sciences, Wuhan University, Wuhan 430079, China

**Keywords:** alkene aziridination, electro-oxidative cross-coupling, flow chemistry, *N*-centered radical, alkene radical cation

## Abstract

Aziridines derived from bioactive molecules may have unique pharmacological activities, making them useful in pharmacology (e.g. mitomycin C). Furthermore, the substitution of the epoxide moiety in epothilone B with aziridine, an analog of epoxides, yielded a pronounced enhancement in its anticancer efficacy. Thus, there is interest in developing novel synthetic technologies to produce aziridines from bioactive molecules. However, known methods usually require metal catalysts, stoichiometric oxidants and/or pre-functionalized amination reagents, causing difficulty in application. A practical approach without a metal catalyst and extra-oxidant for the aziridination of bioactive molecules is in demand, yet challenging. Herein, we report an electro-oxidative flow protocol that accomplishes an oxidant-free aziridination of natural products. This process is achieved by an oxidative sulfonamide/alkene cross-coupling, in which sulfonamide and alkene undergo simultaneous oxidation or alkene is oxidized preferentially. Further anticancer treatments in cell lines have demonstrated the pharmacological activities of these aziridines, supporting the potential of this method for drug discovery.

## Teaser

An electro-oxidative flow protocol has been established for the aziridination of natural products via a sulfamide/alkene cross-coupling, furnishing bioactive aziridines with cytotoxicity against human cancer cell lines.

## INTRODUCTION

**Figure 1.**
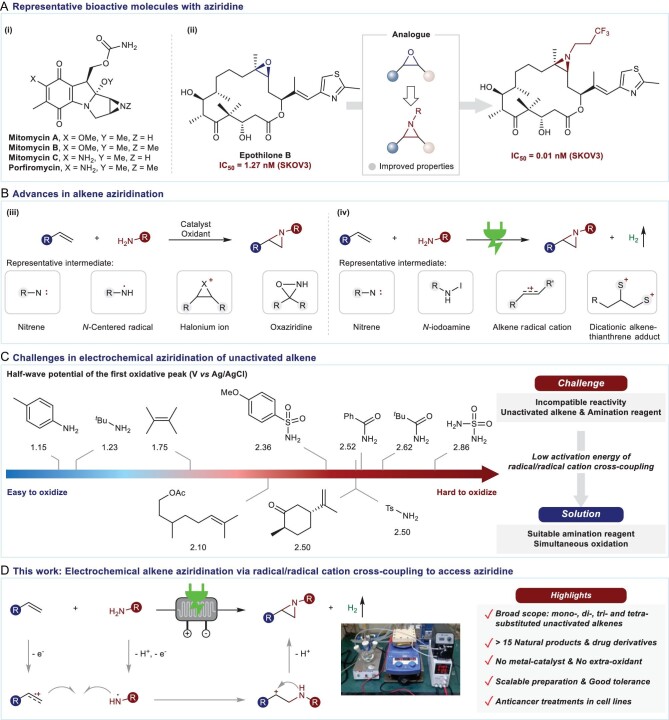
Introduction. (A) Several representative bioactive molecules with aziridines. (B) State-of-the-art: representative synthetic methods for intermolecular aziridination of terpenes. (C) Challenges in electrochemical aziridination of unactivated alkene. (D) This work: electrochemical alkene aziridination in continuous flow via an oxidative sulfonamide/alkene cross-coupling. SKOV3: one type of ovarian cancer cell line.

Due to the physiological properties of nitrogen-containing heterocyclic moieties, the construction of nitrogen-containing compounds has been regarded as one of the central issues in modern synthetic chemistry over the last decades [1–3]. Among these nitrogen-containing compounds, aziridines possess unique biological activities [4,5] that have led to their applications in a variety of areas, ranging from natural products (e.g. mitosanes [6], Fig. 1A-i) to bioactive molecules (e.g. epothilone B analog with anticancer activity for ovarian cancer SKOV3 [7], Fig. 1A-ii). Furthermore, as analogs of epoxide, aziridines derived from bioactive molecules may exhibit pharmacological activities [4,7]. As a result, there is interest in developing novel synthetic technologies to produce aziridines from bioactive natural products [8,9].

Terpenes are extensively utilized in medicinal chemistry [10,11]. Representatively, paclitaxel is a type of terpene with anticancer activity used in clinical application [12]. Moreover, terpene-derived secondary metabolites also play an essential role in anticancer drug discovery, such as the epoxides of lipids [13,14]. Hence, as the analog of epoxides, aziridine-derived terpenes may possess anticancer activities similar to those of terpene-derived secondary metabolites. In this context, we conceived that a practical method to access aziridines from bioactive natural products, especially terpenes, could potentially yield some molecules with anticancer activities, thus contributing to drug discovery [4]. To achieve this goal, we considered recent advances in aziridine synthesis. Catalytic alkene aziridination is one of the most efficient routes to producing aziridines using fundamental feedstocks—alkenes and amines [15,16]. To date, four strategies have been well established for realizing alkene aziridination (Fig. 1B-iii): (i) nitrene [15,17–22]; (ii) nitrogen-centered radical (NCR) [23–25]; (iii) halonium ion [26,27]; (iv) oxaziridine [28]. Despite their versatility, these reactions often require pre-functionalized amination reagents, stoichiometric oxidants, transition-metal catalysts or a combination of several components, compromising their atom economy and further application. Over the last two decades, the resurgence of electrochemical organic synthesis has provided environmentally friendly and sustainable methods for alkene aziridination through the activation of amines or alkenes (Fig. 1B-iv). In these transformations, nitrene [29,30], *N*-iodoamine [31–33], alkene radical cation [34–36] and dication alkene·thianthrene adduct [37] have each been employed as critical intermediates. Nevertheless, these electro-oxidative reactions initiated from the activation of alkenes typically necessitate particular substrates, such as electron-rich alkenes or monosubstituted unactivated alkenes, causing problems in the modification of natural products. For example, aryl alkenes have been commonly used in electrochemical aziridination via an alkene radical cation process [34,36]. Overall, practical methods without a metal catalyst and extra-oxidant for the aziridination of bioactive molecules remain limited.

Significant challenges remain in the electro-oxidative aziridination of unactivated alkenes (Fig. 1C). One challenge is the incompatible reactivity of unactivated alkene and amination reagent. In the pathway of amine activation, although primary alkyl amine and aniline are readily oxidized in the anode, preventing the formation of imine-type byproducts and competing aziridine oxidation is difficult. Electrophilic modification of amination reagent is usually required for electrochemical aziridination via the alkene activation route [34]. However, it causes difficulty in the nucleophilic attack of amination reagent to the alkene radical cation. Therefore, a suitable nitrogen source that is easily oxidized and possesses nucleophilicity may provide an alternative route for electrochemical aziridination.

Inspired by our previous works [38,39], we imagined that the simultaneous oxidation of an alkene and amination reagent might offer a means to achieve alkene aziridination via radical/radical cation cross-coupling [40]. The radical/radical cation species could react with one another since they are generated close to the anode. In addition, using flow reactors in electrochemistry may provide a scalable approach for alkene aziridination [41–47]. An electrochemical flow aziridination of aryl alkenes has been reported by Noël and co-workers [35]. Herein, we demonstrate the simultaneous oxidation of sulfonamide and alkene facilitates an *N*-centered radical/alkene radical cation cross-coupling, leading to the alkene aziridination in electrochemical continuous flow (Fig. 1D). This mechanistically distinct model is compatible with the use of various mono-, di-, tri- and even tetra-substituted unactivated alkenes. This method is suitable for >15 terpenes and drug derivatives, while eliminating the requirement for extra-oxidants and transition-metal catalysts. Further anticancer treatments in cell lines demonstrated the pharmacological effects of these aziridines on lung cancer NCI-460 and breast cancer MCF-7.

## RESULTS AND DISCUSSION

### Cyclic voltammetry studies

**Figure 2.**
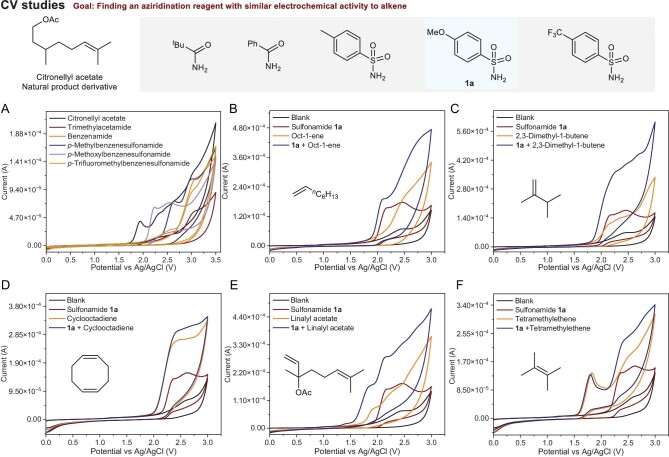
Cyclic voltammetry experiments. (A) CV studies for amides and sulfonamides. (B) CV studies for sulfonamide and monosubstituted alkene. (C) CV studies for sulfonamide and 1,2-disubstituted alkene. (D) CV studies for sulfonamide and 1,1-disubstituted alkene. (E) CV studies for sulfonamide and tri-substituted alkene. (F) CV studies for sulfonamide and tetra-substituted alkene. Blank: 4 mL DCE and 2 mL TFE with 0.1 M TBABF_4_.

Initially, we performed a set of cyclic voltammetry experiments to explore the electrochemical reactivities of terpenes and several aziridination reagents, including trimethylacetamide, benzenamide, *p*-methylbenzenesulfonamide, *p*-methoxylbenzenesulfonamide and *p*-trifluoromethylbenzenesulfonamide (Fig. 2). As shown in Fig. 2A, 4-methoxybenzenesulfonamide **1a** exhibited similar catalytic currents and oxidation potentials to citronellyl acetate. Therefore, 4-methoxybenzenesulfonamide **1a** was chosen for further cyclic voltammetry (CV) studies with different alkenes. Further CV experiments indicated that the catalytic current increased with the addition of mono-, 1,2-di- or tri-substituted alkene (Fig. 2B, C and E), supporting the simultaneous oxidation of both **1a** and alkene. However, the catalytic current of the first oxidative peak remained unchanged with the addition of 1,1-di- and tetra-substituted alkene (Fig. 2D and F). Overall, CV studies demonstrated the feasibility of the simultaneous oxidation of **1a** and alkene for electro-oxidative aziridination.

### Condition optimizations

Subsequently, we aimed to investigate our proposed electro-oxidative aziridination between readily available 4-methoxybenzenesulfonamide **1a** and (+)-3-carene **2a**. After some efforts, optimized reaction conditions were established (Supplementary Table S1) with carbon as the anode, Pt as the cathode, tetrabutylammonium acetate (TBAOAc) as the supporting electrolyte, KOAc as the base (dissolved in the solvent) and a mixture of 1,2-dichloroethane (DCE)/2,2,2-trifluoroethanol (TFE) as the solvent. The continuous-flow electro cell afforded aziridine **3a** in a 77% isolated yield from **1a** (2 mmol) and **2a** (2 equiv. 4 mmol) under a current of 100 mA for 6 h at 35°C (controlled by the heating module).

### Scope of bioactive molecules

**Figure 3.**
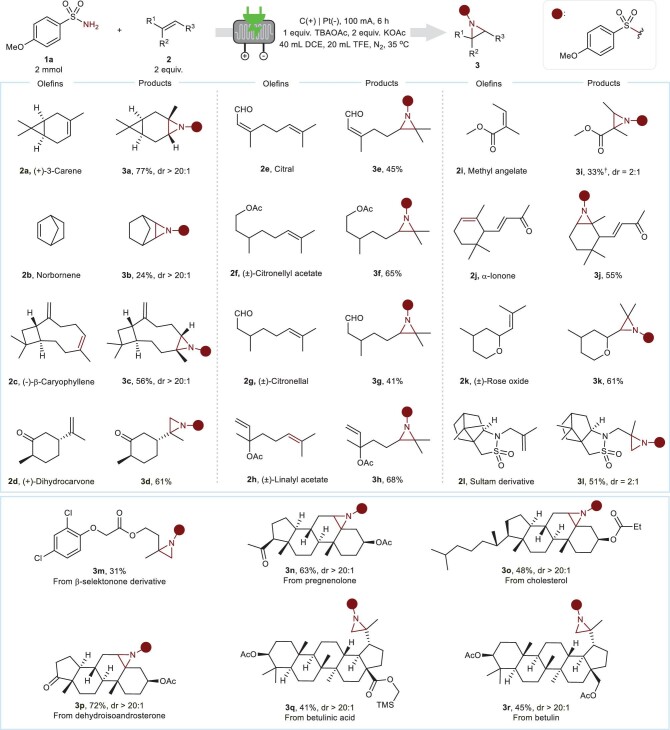
Scope of natural products or drug derivatives. Reaction conditions: **1a** (2 mmol), TBAOAc (2 mmol), KOAc (4 mmol), alkene 2 (2 equiv.), 40 mL of DCE and 20 mL of TFE with a continuous-flow cell under 100 mA of electrolysis for 6 h at 35°C. ^†^4 equiv. alkene used. Isolated yields are shown. Racemic 3d was obtained. Ac, acetyl; TMS, trimethylsilyl.

Then, we focused on the initial goal of the aziridination of bioactive molecules. A series of bioactive molecules, including natural products and drug derivatives, were examined under the optimized conditions (Fig. 3). To our delight, various terpenes were suitable radical acceptors for this aziridination, producing related products (**3a** to **3h**). Moreover, derivatives from bioactive molecules, such as sultam and selektonone, were also successfully converted into products in moderate yields (**3l** and **3m**). In addition, steroids and their derivatives were smoothly converted into the corresponding aziridines **3n** to **3p** in moderate yields. Notably, this electrochemical aziridination was well suited for terpenes with anticancer properties, including betulin and betulinic acid, both of which are active against 12-*O*-tetradecanoylphorbol-13-acetate (TPA)-induced tumors and melanoma xenographs in mice (**3q** and **3r**) [48].

### Synthetic applications

**Figure 4.**
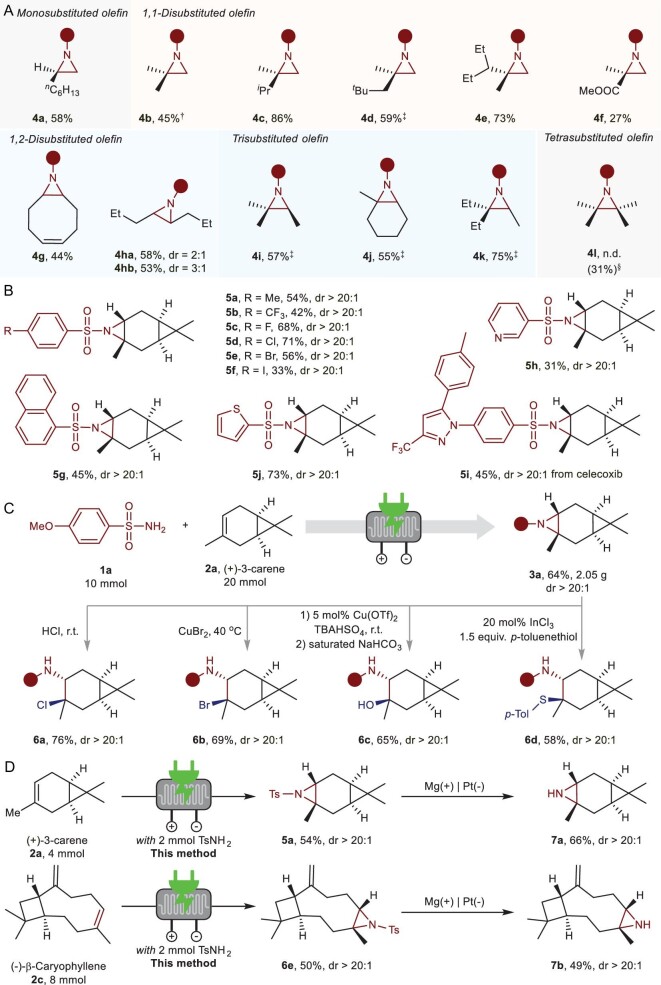
Synthetic applications. (A) Additional scope of unactivated alkenes. Reaction conditions: sulfonamide (2 mmol), TBAOAc (2 mmol), KOAc (4 mmol), alkene (4 equiv.), 40 mL of DCE and 20 mL of TFE with a continuous-flow cell under 100 mA of electrolysis for 6 h at 35°C. ^†^2 M of 2-methylpropene in THF was used. ^‡^2 equiv. alkene was used. ^§^Reaction run with TBAPF_6_ as the supporting electrolyte and 1,8-diazabicyclo[5.4.0]undec-7-ene (DBU) as the base, producing **4l** in 31% isolated yield. **4** **ha** from *cis*-oct-4-ene and **4hb** from *trans*-oct-4-ene. (B) Scope of sulfonamides. (C) Gram-scale experiments and further derivatizations. *p*-Tol, *p*-tolyl. (D) Synthesis of aziridines via electrochemical detosylation. Reaction conditions for aziridination: *p*-toluenesulfonamide (2 mmol), terpene (4 or 8 mmol), TBAOAc (2 mmol), KOAc (4 mmol), 40 mL of DCE and 20 mL of TFE with a continuous-flow cell under 100 mA of electrolysis for 6 h at 35°C.

To explore the applicability of this strategy, we investigated the scope of unactivated alkenes with sulfonamide **1a** (Fig. 4A). Remarkably, a set of mono-, di-, tri- and tetra-substituted alkenes were suitable substrates for the generation of aziridines in moderate to high yields (**4a** to **4l**). Subsequently, we examined the scope of sulfonamides with (+)-3-carene **2a** (Fig. 4B). In this case, *para*-substituted benzenesulfonamides with various functional groups (including Me, CF_3_ and halides) were compatible. In addition, sulfonamides modified by pyridinyl, naphthyl or thienyl were well tolerated under this electrochemical condition (**5h** to **5j**). With the respect to limitation, trimethylacetamide and benzamide failed in the desired transformation. Furthermore, to show the potential of this method in the application, scale-up experiments and derivatizations were carried out (Fig. 4C). Gratifyingly, gram-scale experiments showed that this electro-flow technology could effectively produce product **3a** in 64% isolated yields from 10 mmol of sulfonamide **1a** and 20 mmol of carene **2a**. Further derivatizations demonstrated that **3a** obtained via this method could be easily converted into other chemicals (**6a** to **6d**). Additional electrochemical detosylation was performed to synthesize aziridines (Fig. 4D). According to the work of Senboku and co-workers [49], the protecting group could be effectively removed, forming the target aziridines in moderate yield (**7a** and **7b**). Overall, the results illustrate the effectiveness and scalability of the methodology and suggest its potential utility in further synthetic applications.

### Anticancer treatments in cell lines

**Figure 5.**
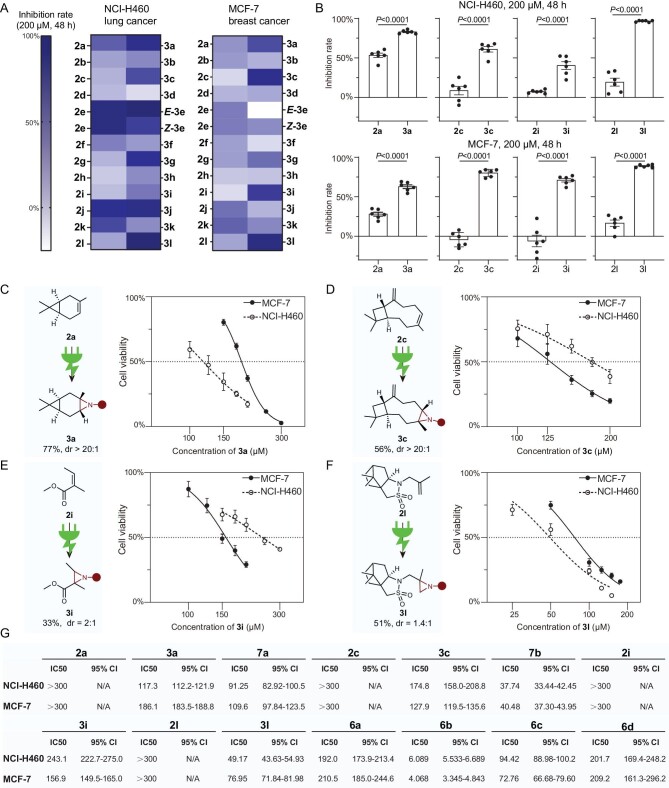
Anticancer effects in cell lines. (A) 48-h inhibition rates of paired compounds in NCI-H460 and MCF-7 cell lines. (B) 48-h inhibition rates of selected paired compounds in cell lines. (C)–(F) Dose–response curves of **3a, 3c, 3i** and **3l**, respectively. (G) Half-maximal inhibitory concentrations (IC50) of selected paired compounds. 95% CI, 95% confidence internal.

In order to determine whether this method has the potential for drug discovery, we investigated the anticancer effects of these aziridines (Fig. 5). According to GLOBOCAN 2020 [50], lung cancer is the leading cause of cancer death with the highest mortality (18%) and breast cancer was the most commonly diagnosed cancer. Therefore, NCI-H460 (lung cancer) and MCF-7 (breast cancer) cell lines were chosen to determine whether the aziridines showed superior anticancer activities when compared with their alkene precursors. As shown in Fig. 5A, high-throughput screening experiments exhibited that most terpenes and aziridines showed moderate inhibition of cell growth in both NCI-H460 and MCF-7. Significantly, aziridines **3a, 3c, 3i** and **3l** performed higher inhibition rates of cell growth than terpenes, supporting their better anticancer effects in NCI-H460 and MCF-7 cell lines (Fig. 5B). Furthermore, a series of anticancer treatments of these alkenes, aziridines and their derivatives were performed under different concentrations to explore their half-maximal inhibitory concentration (IC50) (Fig. 5C–G). For example, in the cytotoxicity assay of the NCI-H460 cell line, while alkene **2l** exhibited lower anticancer activity with an IC50 value of >300 μM, aziridine **3l** was anticancer-active with an IC50 value of <50 μM. In addition, the comparison between **3c** and **7b** shows that removing the protecting group leads to a further decrease in the IC50 value (Supplementary Fig. S8). Moreover, the ring-open derivatives **6b** demonstrated IC50 values of 4 and 6 μM in MCF-7 and NCI-H460, respectively. These results displayed the enhanced anticancer effects of aziridines and their derivatives that could be effectively produced via this method (**3a, 3c, 3i, 3l, 6b** and **7b**), thus supporting the potential for drug discovery.

### Plausible mechanism

**Figure 6.**
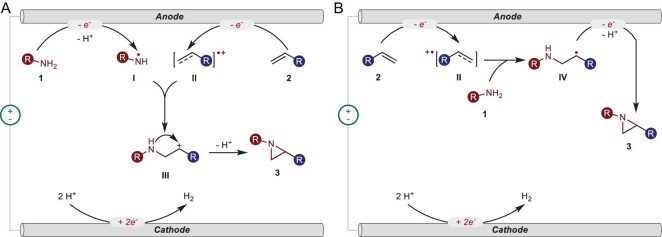
Plausible mechanisms. (A) Simultaneous oxidation route for mono-, 1,2-di- and tri-substituted alkene. (B) Nucleophilic attack way for alkenes that are more easily oxidized than sulfonamide.

Based on the results shown above, two plausible mechanisms were proposed (Fig. 6). For mono-, 1,2-di- and tri-substituted alkene, the mechanism of simultaneous oxidation was proposed (Fig. 6A). Initially, sulfonamide **1** was oxidized at the anode and lost a proton to generate *N*-centered radical **I**. Simultaneously, alkene **2** was oxidized at the anode to form corresponding alkene radical cation **II**. Then, a radical/radical cation cross-coupling between **I** and **II** occurred to form cation intermediate **III**, which could quickly transform into aziridine **3** via an intramolecular cyclization. When the alkene was preferentially oxidized, nucleophilic attack was proposed and is illustrated in Fig. 6B. Alkene **2** was oxidized at the anode to form corresponding alkene radical cation **II**. Then, **II** reacted with sulfonamide **1** via nucleophilic attack to generate radical intermediate **IV**. Finally, **IV** was transformed into aziridine **3** via an oxidative deprotonation. In the cathode, protons were reduced to produce hydrogen.

## CONCLUSION

We have demonstrated a practical method to achieve the aziridination of unactivated alkene in an electro-flow cell with hydrogen evolution, avoiding the use of a transition-metal catalyst and extra-oxidant. This mechanistically distinct technology is realized via an oxidative amine/alkene cross-coupling, thereby exhibiting good compatibility with various bioactive molecules with >15 kinds of natural products and drug derivatives. Further synthetic transformation of aziridine and anticancer treatments supported the potential of this electro-oxidative reaction in the electrochemical and medical industries. We expect that the concept outline here will provide a novel synthetic strategy for alkene aziridination and exhibit an alternative way to access drug candidates.

## METHODS

### Electrochemical aziridination

An oven-dried three-neck flask was charged with a solution of sulfonamide **1** (2 mmol), TBAOAc (2 mmol), KOAc (4 mmol) and alkene **2** (from 2 to 6 equiv.) in the mixture of 40 mL DCE (anhydrous) and 20 mL TFE (anhydrous). The flow cell was equipped with carbon paper (9.3 cm × 9.3 cm × 0.2 mm) as the anode (contact area 1.6 cm^2^) and platinum plate (9.3 cm × 9.3 cm × 0.3 mm) as the cathode (contact area 1.6 cm^2^). The system was flashed with nitrogen before the direct electrolysis. The solution was pumped through the electro cell at a fixed flow rate of 8 mL/min and electrolysed at a constant current of 100 mA under 35°C for 6 h. The reaction was monitored by using TLC and GC via sampling experiments. After 6 h, the reaction mixture was concentrated in a vacuum and purified by using flash column chromatography on silica gel to obtain the expected aziridine.

### Electrochemical detosylation

Aziridine (0.3 mmol) was added to a solution of Et_4_NBr (0.6 mmol) and naphthalene (0.15 mmol) in anhydrous DMF (6 mL) and placed in an oven-dried undivided cell equipped with a Pt plate cathode (1.5 cm × 1.5 cm) and a Mg plate cathode (1.5 cm × 1.5 cm). A constant current electrolysis (5 mA/cm^2^, 4 F/mol) was carried out at 0°C under a nitrogen atmosphere. After the end of the electrolysis, the reaction mixture was diluted using 50 mL of ether and extracted using 1 M of HCl (25 mL × 3). The combined aqueous phase was basified using 1.5 M of aqueous NaOH to pH 8–9 and the basified aqueous phase was extracted using ether (25 mL × 3) and dried over anhydrous MgSO_4_. The organic phase was concentrated under a vacuum to provide the parent aziridine.

## Supplementary Material

nwad187_Supplemental_File
