# Supplementary material for: Electrochemical flow aziridination of unactivated alkenes
Source: Natl Sci Rev. 2023 Jul 3;10(10):nwad187. doi: 10.1093/nsr/nwad187 (PMC10697417; doi:10.1093/nsr/nwad187)
Supplement: nwad187_Supplemental_File [file nwad187_supplemental_file.pdf]

Supplementary Information

Electrochemical Flow Aziridination of Unactivated Alkenes

Shengchun Wang*, Pengjie Wang*, Shu-Jin Li*, Yi-Hung Chen✉, Zhi-Jun Sun✉ and Aiwen Lei✉

*These authors contributed equally to this work

✉Corresponding author. Email: yihungchen@whu.edu.cn; sunzj@whu.edu.cn; aiwenlei@whu.edu.cn

Contents

General Information 3
Experimental Procedures 4
Mechanistic Studies 12
Bioactivity Investigation 18
NOESY spectra 22
Characterization of Products 22
NMR Spectra 43
References 94

General Information

All glass wares were oven dried at 110 °C overnight and cooled down under an argon atmosphere. Unless otherwise noted, materials were commercially available and without further purification. Thin layer chromatography (TLC) employed glass 0.25 mm silica gel plates. Gradient flash chromatography was conducted eluting with a continuous gradient from petroleum ether (bp. 60-90 °C) to the ethyl acetate. All the new compounds were characterized by ^{1}H NMR, ^{13}C NMR and HRMS. The ^{1}H NMR, ^{13}C NMR and ^{19}F NMR spectra were recorded on a Bruker 400 MHz NMR spectrometer. The chemical shifts (δ) were given in part per million $CDCl_3$ (7.26 ppm for ^{1}H NMR and 77.00 ppm for ^{13}C NMR). All ^{1}H NMR spectra were reported in delta (δ) units, parts per million (ppm) downfield from the internal standard. Coupling constants are reported in Hertz (Hz). The multiplicities of signals are designated by the following abbreviations: s (singlet), d (doublet), t (triplet), q (quarter), m (multiplet), dd (doublet and doublet), dt (doublet and triplet), td (triplet and doublet). GC yields were recorded with a Shimadzu GC-2014. High resolution mass spectra (HRMS) were measured with a Bruker UltiMate 3000 & Compact and accurate masses were reported for the molecular ion + Hydrogen ($M+H^+$) or molecular ion - water + Hydrogen ($M-H_2O+H^+$).

In all electrochemical continuous-flow reactions, a homemade flow cell was used[1], together with a switching DC voltage regulator (HYELEC-HY3005B) (made in China). The cell consists of a working electrode and a counter electrode, with a PTFE (Polytetrafluoroethylene) gasket of 1.0 mm thick, containing a rectangular reaction channel (total length: 784 mm, width: 1.0 mm) in between. The material used for the electrodes were platinum plate and graphite paper. The reactor volume is 1.6 mL. This results in an undivided electrochemical cell. In the cell, direct contact between the electrode surface and the reaction mixture is established. The reaction mixture is pumped through the system via a peristaltic pump and then collected in a three-neck flask. The pump used in the experiment is a KCS PRO model peristaltic pump manufactured by Kamoer.

Experimental Procedures

General procedure for synthesis of sulfonamides

An oven dried 100 mL round bottom flask equipped with a stir bar, charged with sulfonyl chloride (10 mmol) and 20 mL DCM under nitrogen atmosphere, then flask was cooled down in ice-water bath. 25 mL ammonia was dropped into flask slowly via a syringe. The reaction mixture was stirred violently at room temperature overnight. Then reaction mixture was extracted with ethyl acetate, combined organic layers were dried over anhydrous sodium sulphate and filtered. The filtrate was collected, and solvent was removed in vacuum to offer corresponding sulfonamide as a white solid or crystal.

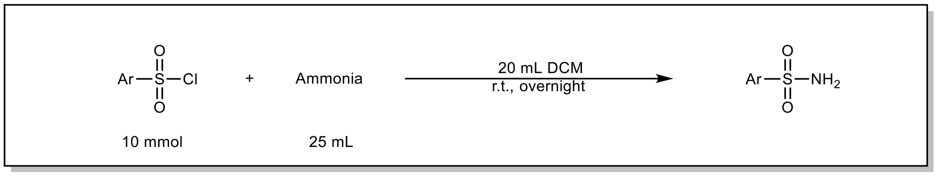

Procedure for synthesis of 2l

A solution of (2*R*)-bornane-10,2-sultam (5 mmol), 3-bromo-2-methylpropene (6 mmol), cesium carbonate (6 mmol) in 10 mL DCM was stirred under nitrogen atmosphere at room temperature for 24 h. Then reaction mixture was diluted with distilled water and extracted with ethyl acetate (3×10 mL), combined organic layers were dried over anhydrous sodium sulphate and filtered. The collected filtrate was concentrated in vacuum and purified by flash column chromatography on silica gel to obtain **2l** in 62% yield.

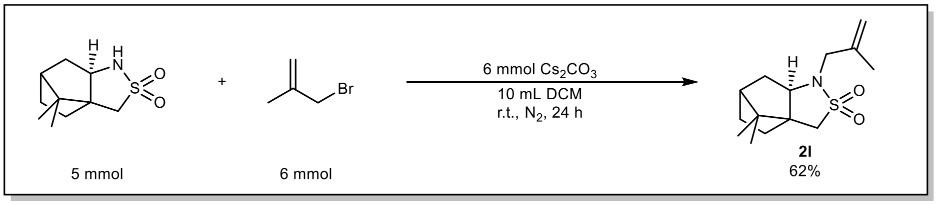

Procedure for synthesis of 2m

A solution of 2,4-dichlorophenoxyacetic acid (10 mmol), 3-methylbut-3-en-1-ol (15 mmol), EDC (3-(ethyliminomethylideneamino)-*N*,*N*-dimethylpropan-1-amine, hydrochloride, 15 mmol) and DMAP (4-dimethylaminopyridine, 0.5 mmol) in 20 mL DCM was stirred under nitrogen atmosphere at room temperature overnight. Then reaction mixture was diluted with distilled water and extracted with ethyl acetate (3×15 mL), combined organic layers were dried over anhydrous sodium sulphate and filtered. The collected filtrate was concentrated in vacuum and purified by flash column chromatography on silica gel to obtain **2m** in 92% yield.

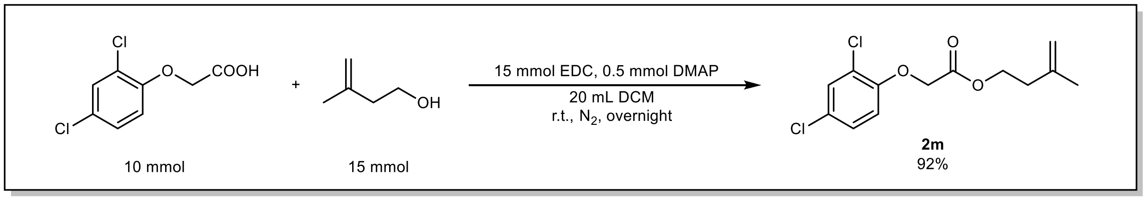

Procedure for synthesis of 2q[2]

To a solution of betulinic acid (5 mmol) in 200 mL $CHCl_3$, diazomethyl(trimethyl)silane (25 mmol, 2M in hexane) was added into solution dropwise via a syringe, then reaction mixture was stirred for 24 h under nitrogen atmosphere at room temperature. Then solvent was removed in vacuum and purified by flash column chromatography on silica gel to obtain crude product.

Crude product was added to a suspension of 10 mL acetic anhydride and 5 mL pyridine. Reaction mixture was heated to reflux for 6 h. Then reaction mixture was diluted with distilled water and extracted with ethyl acetate (3×15 mL) after cooled down to room temperature. combined organic layers were dried over anhydrous sodium sulphate and filtered. The collected filtrate was concentrated in vacuum and purified by flash column chromatography on silica gel to obtain **2q** in 52% yield.

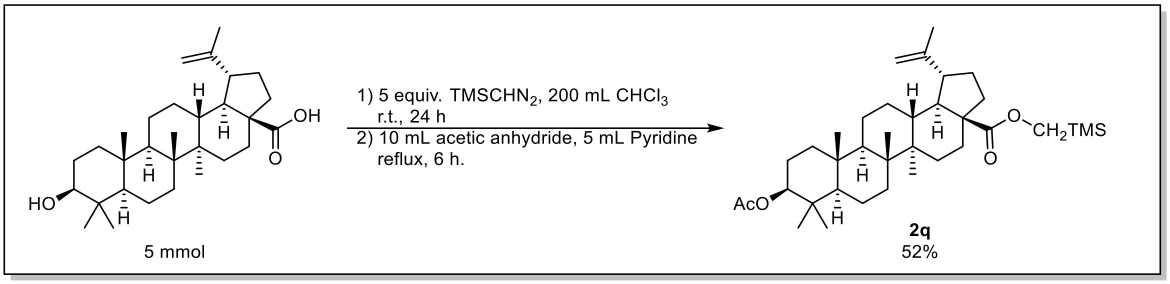

Procedure for synthesis of 2r[3]

To a solution of betulin (5 mmol), DMAP (4-dimethylaminopyridine, 0.5 mmol) in 20 mL pyridine, acetic anhydride (15 mmol) was added via a syringe dropwise, then reaction mixture was stirred for 1 h under nitrogen atmosphere at room temperature. Reaction mixture was pulled into a 250 mL bulk and diluted with 50 mL DCM and 50 mL distilled water in ice-water bath, acidified with concentrate Hydrogen Chloride to pH = 7. then extracted with ethyl acetate (3×15 mL), and combined organic layers were dried over anhydrous sodium sulphate and filtered. The collected filtrate was concentrated in vacuum and purified by flash column chromatography on silica gel to obtain **2r** in 92% yield.

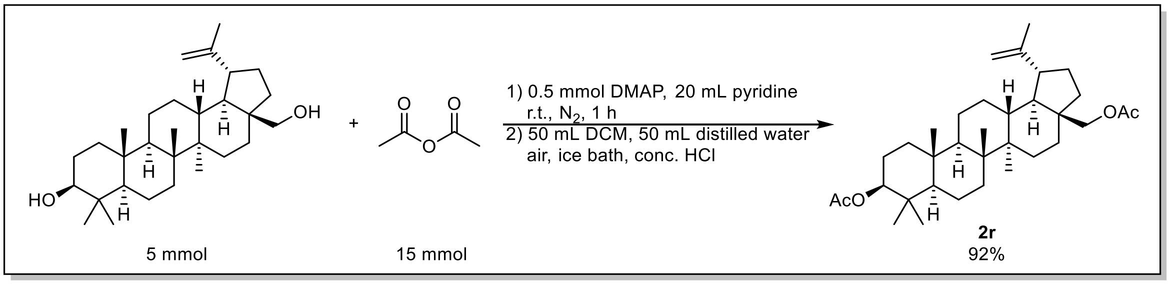

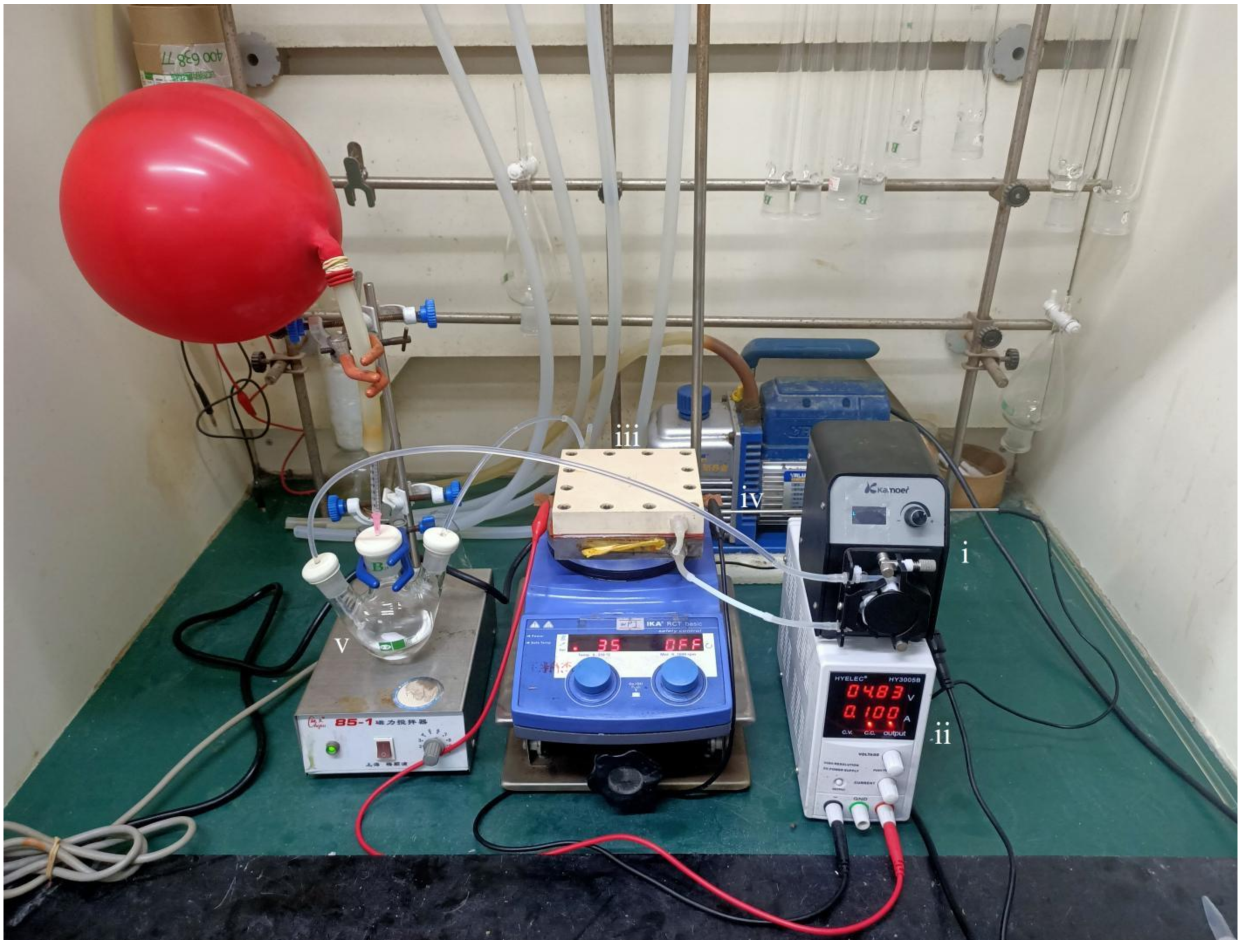

a: picture of the electrochemical continuous-flow reactor

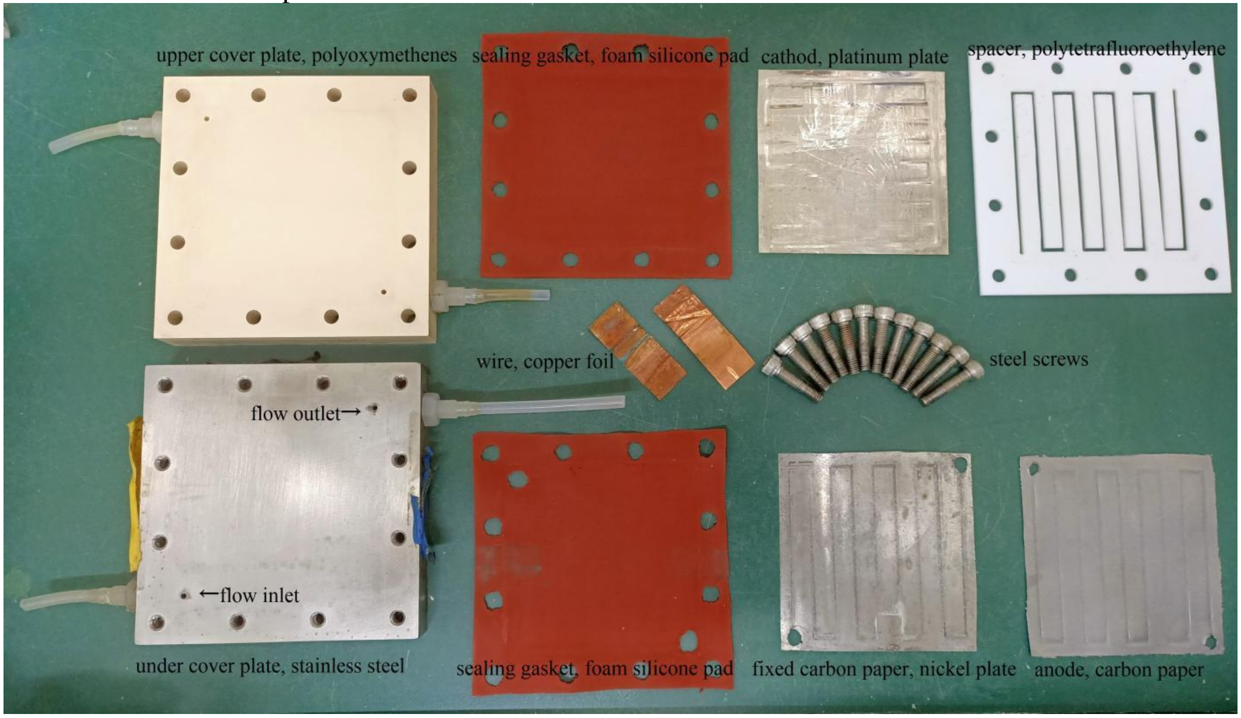

b: physical map of device

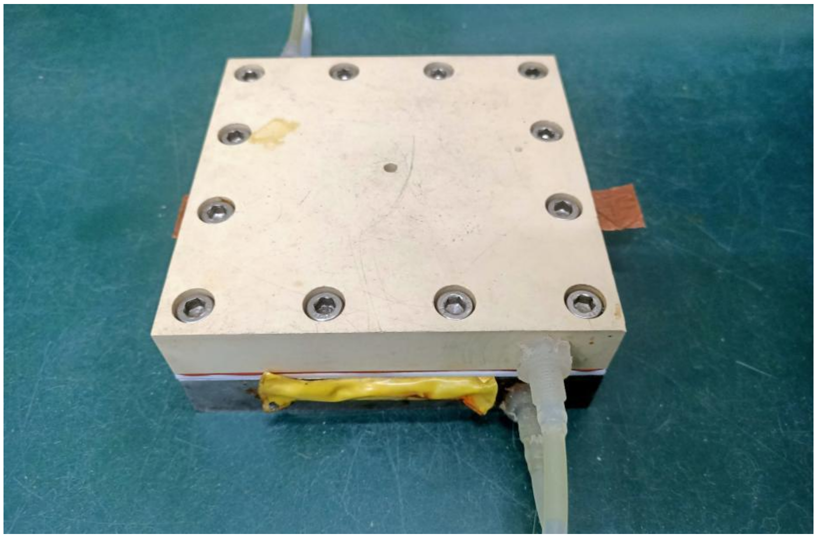

c: flow cell

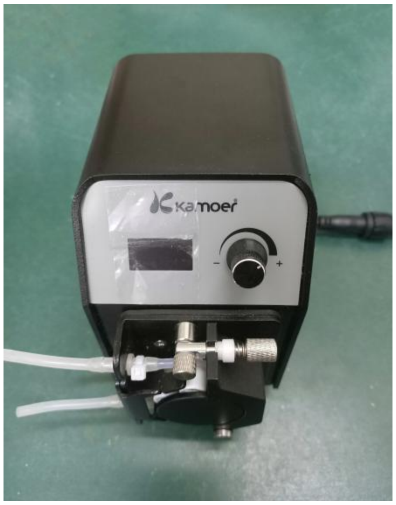

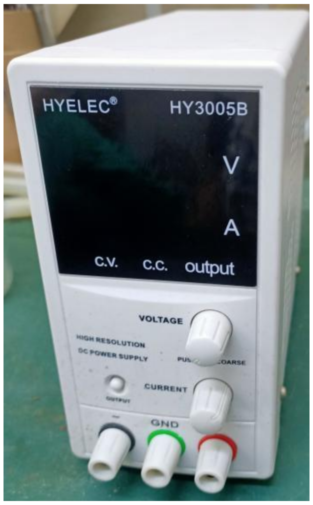

d: peristaltic pump **e**: switching DC voltage regulator

Supplementary Figure 1. The components of electrochemical continuous-flow reactor. a) Picture of the electrochemical continuous-flow reactor and electrolysis device: (i) peristaltic pump, (ii) switching DC voltage regulator, (iii) electrochemical flow reactor, (iv) temperature sensor and (v) collection three-neck flask. b) Physical map of device. c) Flow cell. d) Peristaltic pump. e) Switching DC voltage regulator.

Method for assembling a flow cell and electrolysis device

The plate was covered with a dried sealing gasket, nickel plate (for holding graphite paper), graphite paper, and the PTFE spacer, respectively, and aligning the reaction channel before covered with the platinum plate, another dried sealing gasket and cover plate, respectively. Two pieces of copper foils were inserted between electrodes and sealing gaskets for connecting with a DC voltage regulator. The assembled flow cell was held together by steel screws.

The assembled flow cell was connected with a peristaltic pump and fixing flow rate with a mixture of DCE and TFE, then connected the flow cell with a peristaltic pump, and a three-neck flask. After violently stirring to dissolve KOAc, transparent solution was pumped from flask to peristaltic pump and flow cell in sequence, and returned into flask, then flow cell was heated to

35 ^{o}C. Connected the flow cell with a DC voltage regulator via copper foils and powered up to electrolysis.

General procedure A for electrochemical aziridination

An oven dried three-neck flask was charged with a solution of sulfonamide (2 mmol), TBAOAc (2 mmol), KOAc (4 mmol) and terpene (2 equiv.) in 40 mL DCE and 20 mL TFE, The flow cell was equipped with carbon paper (9.3 cm×9.3 cm×0.2 mm) as the anode (contact area 1.6 cm^2) and platinum plate (9.3 cm×9.3 cm×0.3 mm) as the cathode (contact area 1.6 cm^2). The system was flashed with nitrogen before the direct electrolysis. The solution was pumped through the electro cell at a fixed flowrate of 8 mL/min and electrolyzed at a constant current of 100 mA under 35 ^{o}C for 6 h. Reaction was monitored by TLC and GC. After completion of the reaction, reaction mixture was concentrated in vacuum and purified by flash column chromatography on silica gel to obtain the expected aziridine.

General procedure B for electrochemical aziridination

An oven dried three-neck flask was charged with a solution of sulfonamide (2 mmol), TBAOAc (2 mmol), KOAc (4 mmol) and olefin (2 or 4 or 6 equiv.) in 40 mL DCE and 20 mL TFE, The flow cell was equipped with carbon paper (9.3 cm×9.3 cm×0.2 mm) as the anode (contact area 1.6 cm^2) and platinum plate (9.3 cm×9.3 cm×0.3 mm) as the cathode (contact area 1.6 cm^2). The system was flashed with nitrogen before the direct electrolysis. The solution was pumped through the electro cell at a fixed flowrate of 8 mL/min and electrolyzed at a constant current of 100 mA under 35 ^{o}C for 6 h. Reaction was monitored by TLC and GC. After completion of the reaction, reaction mixture was concentrated in vacuum and purified by flash column chromatography on silica gel to obtain the expected aziridine.

Procedure for tetrasubstituted olefin aziridination

An oven dried three-neck flask was charged with a solution of 4-methoxybenzenesulfonamide (2 mmol), $TBAPF_6$ (2 mmol), DBU (4 mmol) and tetramethyl ethylene (4 equiv.) in 40 mL DCE and 20 mL TFE, The flow cell was equipped with carbon paper (9.3 cm×9.3 cm×0.2 mm) as the anode (contact area 1.6 cm^2) and platinum plate (9.3 cm×9.3 cm×0.3 mm) as the cathode (contact area 1.6 cm^2). The system was flashed with nitrogen before the direct electrolysis. The solution was pumped through the electro cell at a fixed flowrate of 8 mL/min and electrolyzed at a constant current of 100 mA under 35 ^{o}C for 6 h. Reaction was monitored by TLC. After completion of the reaction, reaction mixture was concentrated in vacuum and purified by flash column chromatography on silica gel to obtain aziridine **4l**.

Optimization of aziridination of car-3-ene

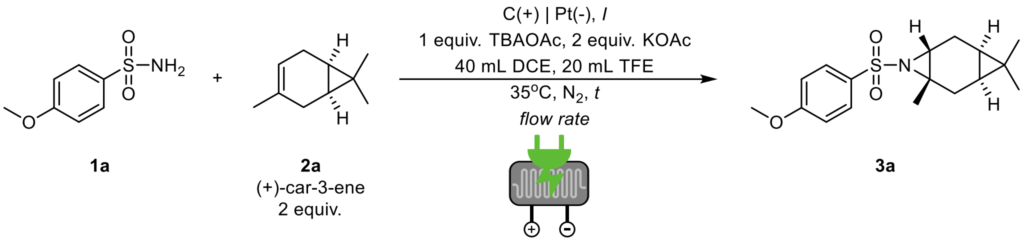

Entry	Flow rate	Current	Time	Yield[a]
1	8 mL/min	100 mA	6 h	77%
2	4 mL/min	100 mA	6 h	66%
3	12 mL/min	100 mA	6 h	70%
4	8 mL/min	125 mA	4.8 h	63%
5	8 mL/min	75 mA	8 h	70%
6	8 mL/min	100 mA	3 h	45%[b]
7	8 mL/min	100 mA	4 h	51%[b]
8	8 mL/min	100 mA	5 h	58%[b] (55%)

Supplementary Table 1. A solution of 4-methoxybenzenesulfonamide (2 mmol), (+)-car-3-ene (2 equiv.), TBAOAc (2 mmol) and KOAc (4 mmol) in 40 mL DCE and 20 mL TFE was stirred at 35 °C under nitrogen atmosphere and pumped into electrochemical flow cell. Then, the product was purified by flash column chromatography on silica gel to obtain aziridine **3a**. [a]Isolated yield. [b]GC yield with biphenyl as internal standard.

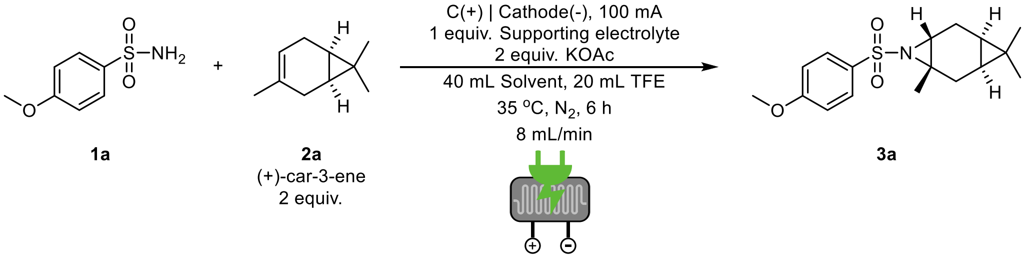

Entry	Cathode	Supporting electrolyte	Solvent	Yield[a]
1	Pt	TBAOAc	40 mL DCE	77%
2	Pt	TBAOAc	40 mL MeCN	60%
3	Pt	TBAOAc	40 mL $PhCF_3$	52%
4	Pt	TBACl	40 mL DCE	54%
5	Pt	KOAc	40 mL DCE	60%
6	Pt	None	40 mL DCE	55%
7	Ni	TBAOAc	40 mL DCE	56%

Supplementary Table 2. A solution of 4-methoxybenzenesulfonamide (2 mmol), (+)-car-3-ene (2 equiv.), TBAOAc (2 mmol) and KOAc (4 mmol) in corresponding solvent and 20 mL TFE was stirred at 35 °C under nitrogen atmosphere and pumped into electrochemical flow cell. Then, the product was purified by flash column chromatography on silica gel to obtain aziridine **3a**. [a]Isolated yield.

Procedure for scale-up reaction

An oven dried three-neck flask was charged with a solution of 4-methoxybenzenesulfonamide (10 mmol), TBAOAc (10 mmol), KOAc (20 mmol) and (+)-car-3-ene (20 mmol) in 200 mL DCE and 100 mL TFE, The flow cell was equipped with carbon paper (9.3 cm×9.3 cm×0.2 mm) as the anode (contact area 1.6 cm^2) and platinum plate (9.3 cm×9.3 cm×0.3 mm) as the cathode (contact area 1.6 cm^2). The system was flashed with nitrogen before the direct electrolysis. The solution was pumped through the electro cell at a fixed flowrate of 8 mL/min and electrolyzed at a constant current of 200 mA under 35 °C for 15 h. Reaction was monitored by TLC and GC. After completion of the reaction, reaction mixture was concentrated in vacuum and purified by flash column chromatography on silica gel to obtain aziridine **3a** in 64% isolated yield.

General procedure C for ring-opening reaction of aziridine 3a[4-6]

A solution of aziridine **3a** (0.2 mmol), Lewis acid and nucleophile (or inorganic acid) was stirred under nitrogen atmosphere. Reaction was monitored by TLC. Saturated solution of $NaHCO_3$ was added to reaction when all aziridine **3a** was consumed. Then reaction mixture was extracted with ethyl acetate (3×10 mL), and combined organic layers were dried over anhydrous sodium sulphate and filtered. The collected filtrate was concentrated in vacuum and purified by flash column chromatography on silica gel to give corresponding product.

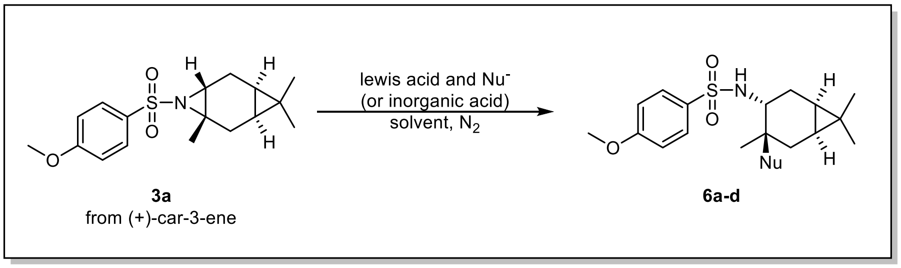

Procedure for electrochemical detosylation of aziridine 3a to synthesize 7a[7]

Aziridine **5a** (0.3 mmol) was added to a solution of Et_4NBr (0.6 mmol) and naphthalene (0.15 mmol) in anhydrous DMF (6 mL) placed in an oven dried undivided cell equipped with a Pt plate cathode (1.5 cm×1.5 cm) and a Mg plate anode (1.5 cm×1.5 cm). A constant current electrolysis (5 mA/cm^2, 4 F/mol) was carried out at 0 °C under nitrogen atmosphere. After finish of electrolysis, the reaction mixture was diluted with 50 mL ether and extracted with 1 M HCl (25 mL×3) immediately. Combined aqueous phase was basified with 1.5 M aqueous NaOH to pH 8-9, basified aqueous phase was extracted with ether (25 mL×3) and dried over anhydrous sodium sulphate. Organic phase was concentrated under reduced pressure to provide the parent aziridine **7a** in 66% isolated yield. The stereoscopic configuration is consistent with that reported in literature.[8]

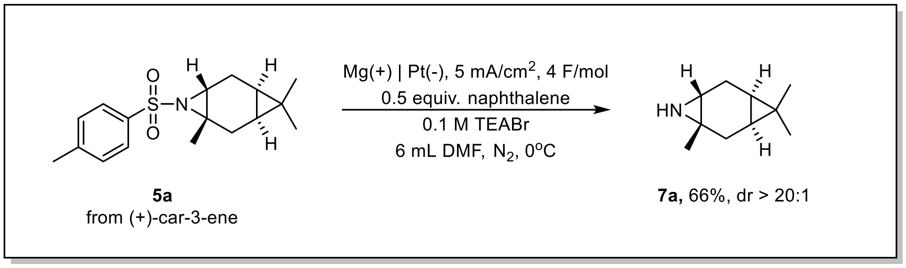

Procedure for electrochemical detosylation of aziridine 6e to synthesize 7b[7]

Aziridine **6e** (0.3 mmol) was added to a solution of Et_4NBr (0.6 mmol) and naphthalene (0.15 mmol) in anhydrous DMF (6 mL) placed in an oven dried undivided cell equipped with a Pt plate cathode (1.5 cm×1.5 cm) and a Mg plate anode (1.5 cm×1.5 cm). A constant current electrolysis (5 mA/cm^2, 4 F/mol) was carried out at 0 °C under nitrogen atmosphere. After finish of electrolysis, the reaction mixture was diluted with 50 mL ether and extracted with 1 M HCl (25 mL×3) immediately. Combined aqueous phase was basified with 1.5 M aqueous NaOH to pH 8-9, basified aqueous phase was extracted with ether (25 mL×3) and dried over anhydrous sodium sulphate. Organic phase was concentrated under reduced pressure to provide the parent aziridine **7b** in 49% isolated yield. The stereoscopic configuration is consistent with that reported in literature.[8]

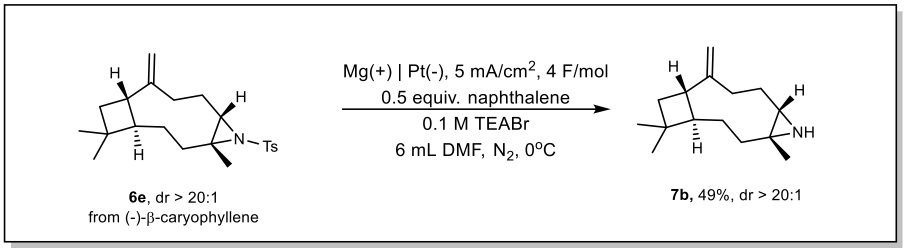

Mechanistic Studies

General procedure A for CV experiments:

CV was performed in a three-electrode cell with 4 mL DCE and 2 mL TFE at 10 ^{o}C. Glassy carbon was used as working electrode, Pt wire as the counter electrode and Ag/AgCl as the reference electrode. The scan rate was 25 mV/s, range was 0 V to 3V or 3.5 V. $TBABF_4$ (0.1 M) was used as the supporting electrolyte. 2 mM substrate was used in CV experiments unless otherwise noted.

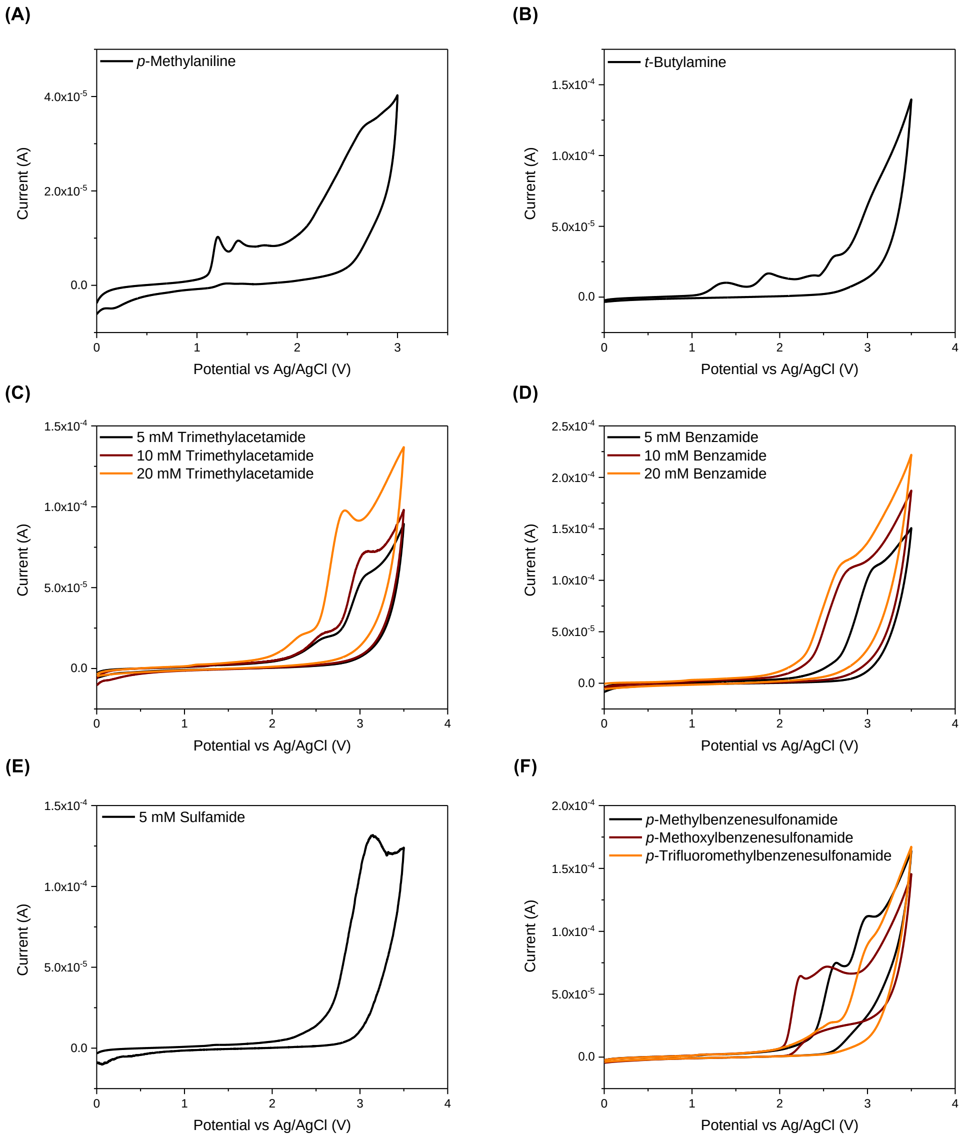

Supplementary Figure 2. Cyclic voltammetry experiments for amines. (A) CV studies for *p*-methylaniline. (B) CV studies for *t*-butylamine. (C) CV studies for trimethylacetamide. (D) CV studies for benzamide. (E) CV studies for sulfamide. (F) CV studies for *p*-methylbenzenesulfonamide, *p*-methoxylbenzenesulfonamide and *p*-trifluoromethylbenzenesulfonamide.

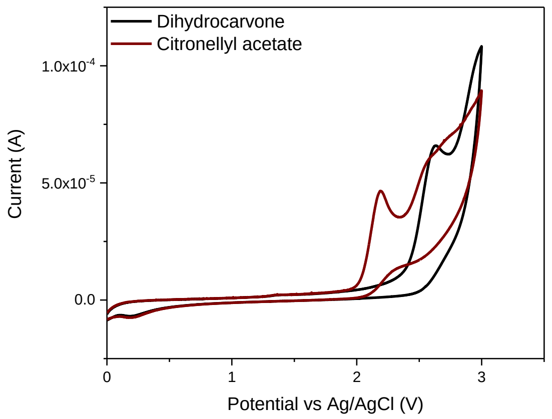

Supplementary Figure 3. Cyclic voltammetry experiments for natural products. (A) CV study for dihydrocarvone. (B) CV study for citronellyl acetate.

General procedure B for CV experiments:

CV was performed in a three-electrode cell with 4 mL DCE and 2 mL TFE at 30 °C. Glassy carbon was used as working electrode, Pt wire as the counter electrode and Ag/AgCl as the reference electrode. The scan rate was 100 mV/s, range was 0 V to 3 V. $TBABF_4$ (0.1 M) was used as supporting electrolyte. 2 mM substrate was used in CV experiments.

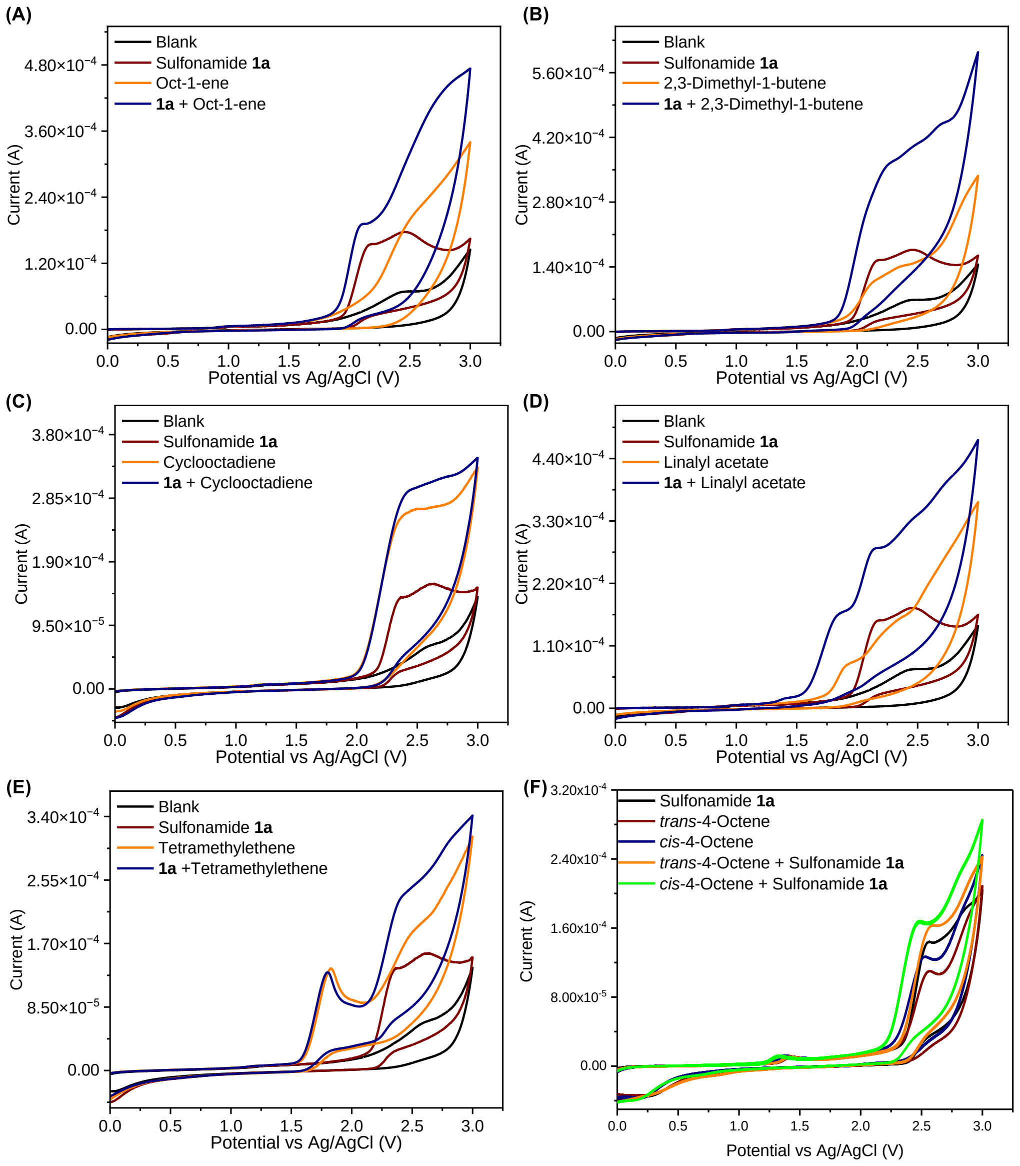

Supplementary Figure 4. Cyclic voltammetry experiments for olefins. (A) CV studies for **1a** and oct-1-ene. (B) CV studies for **1a** and 2,3-dimethyl-1-butene. (C) CV studies for **1a** and cyclooctadiene. (D) CV studies for **1a** and linalyl acetate. (E) CV studies for **1a** and tetramethylethene. (F) CV studies for **1a**, *trans*-4-octene and *cis*-4-octene. Experiment conditions: a mixture of sulfonamide **1a** (2 mM), and olefin (4 mM) in 4 mL DCE and 2 mL TFE with 0.1 M $TBABF_4$.

With the addition of TBAOAc and KOAc, the oxidative peak of **1a** was not changed obviously. Moreover, the oxidative peak of the mixture of **1a** and olefins was a superposition, supporting a simultaneous oxidation of **1a** and olefins (Supplementary Figure 4.A-C, F, **1a** with mono-, di-, and tri-substituted olefin). However, when olefin (Supplementary Figure 4.E, **1a** with tetra-substituted olefin)was oxidized preferentially under standard conditions, the desired transformation was failed. Based on above results, a mechanism of simultaneous oxidation of sulfonamides and olefins was proposed.

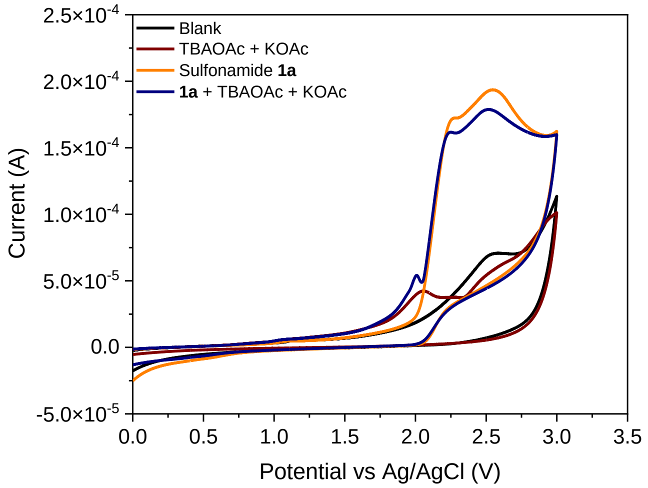

Supplementary Figure 5. Cyclic voltammetry experiments for **1a** and base.

Red line: Cyclic voltammetry of a mixture of TBAOAc (2 mM) with KOAc (2 mM) in 4 mL DCE and 2 mL TFE with 0.1 M $TBABF_4$. **Orange line**: Sulfonamide **1a** (2 mM) in 4 mL DCE and 2 mL TFE with 0.1 M $TBABF_4$. **Blue line**: A mixture of sulfonamide **1a** (2 mM) and TBAOAc (2 mM) and KOAc (2 mM) in 4 mL DCE and 2 mL TFE with 0.1 M $TBABF_4$.

With the addition of TBAOAc and KOAc, the oxidative peak of **1a** was not changed obviously. Therefore, proton-coupled electron transfer (PCET) was not a major route in the desired aziridination of olefins.

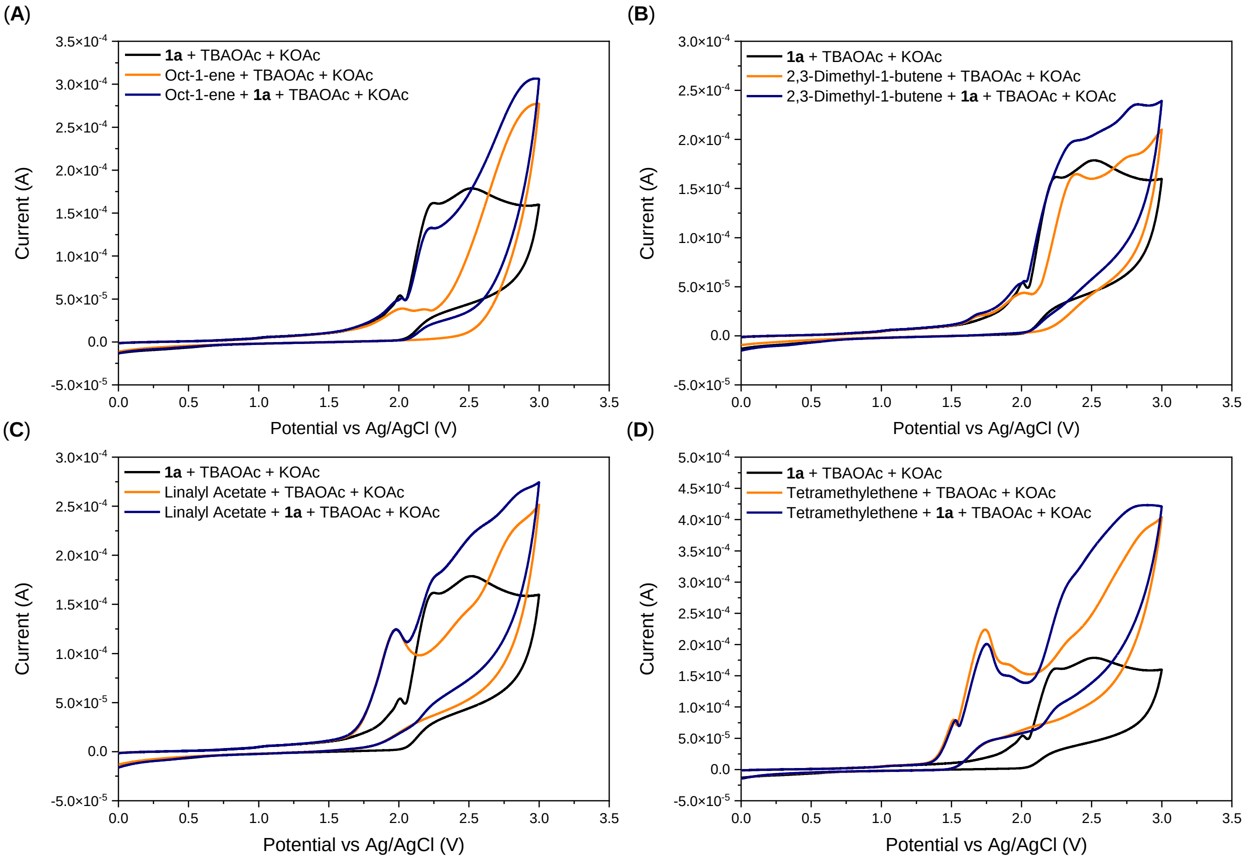

Supplementary Figure 6. Cyclic voltammetry experiments for sulfonamide **1a** and olefins with TBAOAc and KOAc. (A) CV studies for **1a** and oct-1-ene. (B) CV studies for **1a** and 2,3-dimethyl-1-butene. (C) CV studies for **1a** and linalyl acetate. (D) CV studies for **1a** and tetramethylethene.

A mixture of **1a** (2 mM), TBAOAc (2 mM), KOAc (2 mM) and olefin (4 mM) in 4 mL DCE and 2 mL TFE with 0.1 M $TBABF_4$ was used for CV experiments.

Discussion about diastereoselectivity:

Entry	Substrate	Product	Configuration of aziridine
1	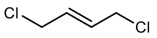	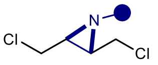	76%, *trans*
2	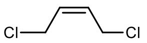	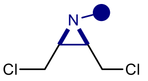	73%, *cis*
3	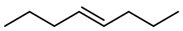	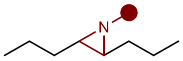	53%, dr = 3:1
4	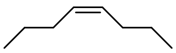	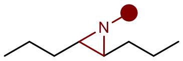	58%, dr = 2:1

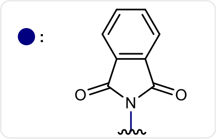

Yudin's works:
Electrochemical nitrene pathway
Reference:
J. Am. Chem. Soc., **2002,** *124,* 530
J. Org. Chem., **2005,** *70,* 932

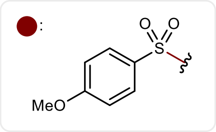

This work:
Electrochemical radical pathway

Supplementary Figure 7. Diastereoselectivity in electrochemical aziridination.

The diastereoselectivity in this work is distinct from an electrochemical nitrene pathway. For example, the discrepancy of diastereoselectivity was 2/1 or 3/1 when *cis*- and *trans*-4-octene were used respectively (Supplementary Figure 7). Differently, in Yudin's work, the diastereoselectivity with 1,2-disubstituted internal olefins is > 20:1 and consistent with chemical oxidation method.[9,10]

Bioactivity Investigation

Cell culture

NCI-H460 and MCF-7 cells were obtained from the China Center for Type Culture Collection (Wuhan University, Wuhan, China) and were grown in RPMI 1640 medium supplemented with 10% FBS and 2 mM *L*-glutamine. All cells were grown at 37 °C in a 5% CO_2 incubator and routinely tested to be mycoplasma-free by the standard PCR method. The identity of the cells was frequently checked by their morphological features.

Cell viability and death detection

Cell viability was evaluated with a Cell Counting Kit-8 (Biosharp Life Sciences, China) according to the manufacturer's instructions. Cells are seeded in 96-well flat bottom microtiter plates at a density of 3000 cells per well and cultured for 24 h. Then the cells were treated with compounds at indicated concentrations for 48 h. The absorbance was measured on a microplate reader at 450 nm. The % growth inhibition was calculated according to the following formula: % of control cell growth = $(OD_{sample} - \text{mean } OD_{day\ 0}) / (\text{mean } OD_{neg\ control} - \text{mean } OD_{day\ 0}) \times 100$, % growth inhibition = 100 - % of control cell growth. The IC50 values and the corresponding asymmetrical confidence intervals were calculated by nonlinear regression using GraphPad Prism version 9.5.0.

Statistical methods

Statistical analysis was performed using GraphPad Prism version 9.5.0. Independent, two-tailed *t*-test with Welch's correction was used to determine whether the variance between two means is similar. *P* values of less than 0.05 were considered significant.

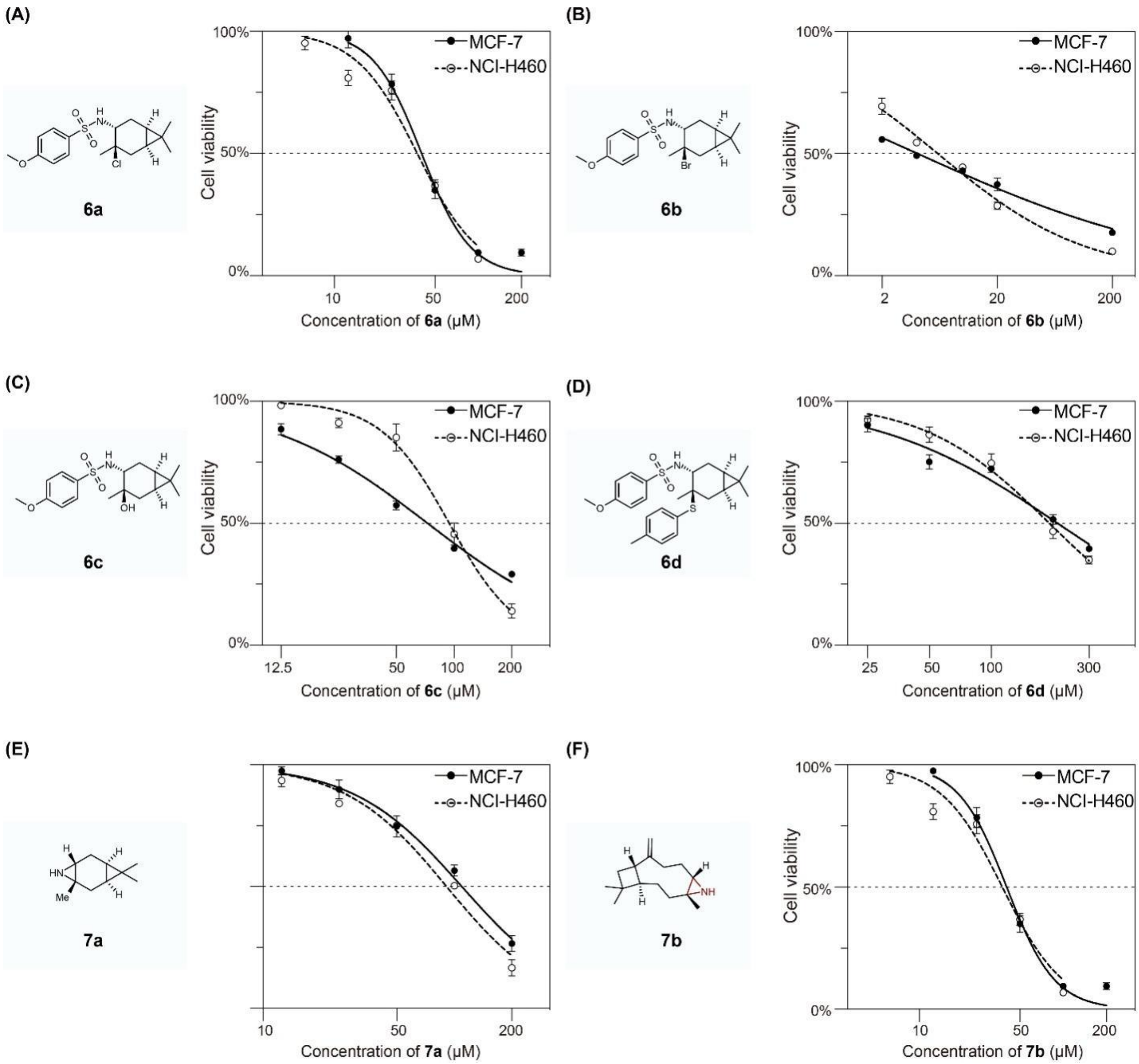

Supplementary Figure 8. (**A**-**F**) Dose-response curves of **6a**, **6b**, **6c**, **6d**, **7a**, and **7b**, respectively.

NOESY spectra

6a NOESY

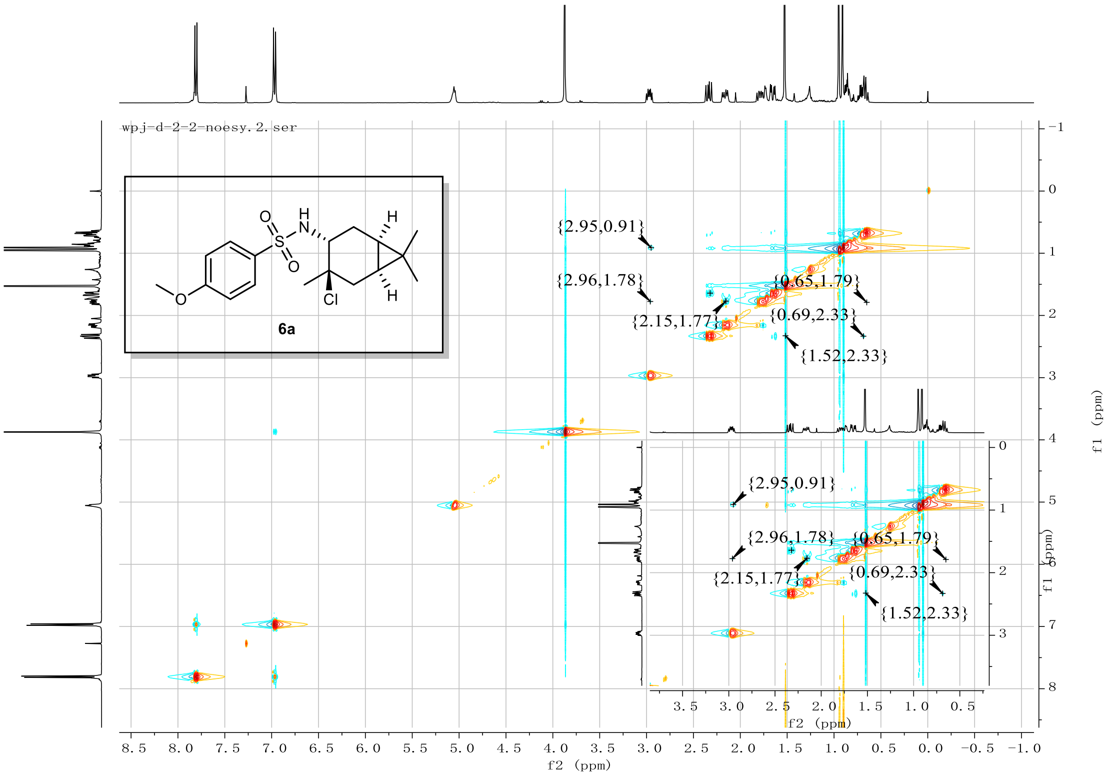

6b NOESY

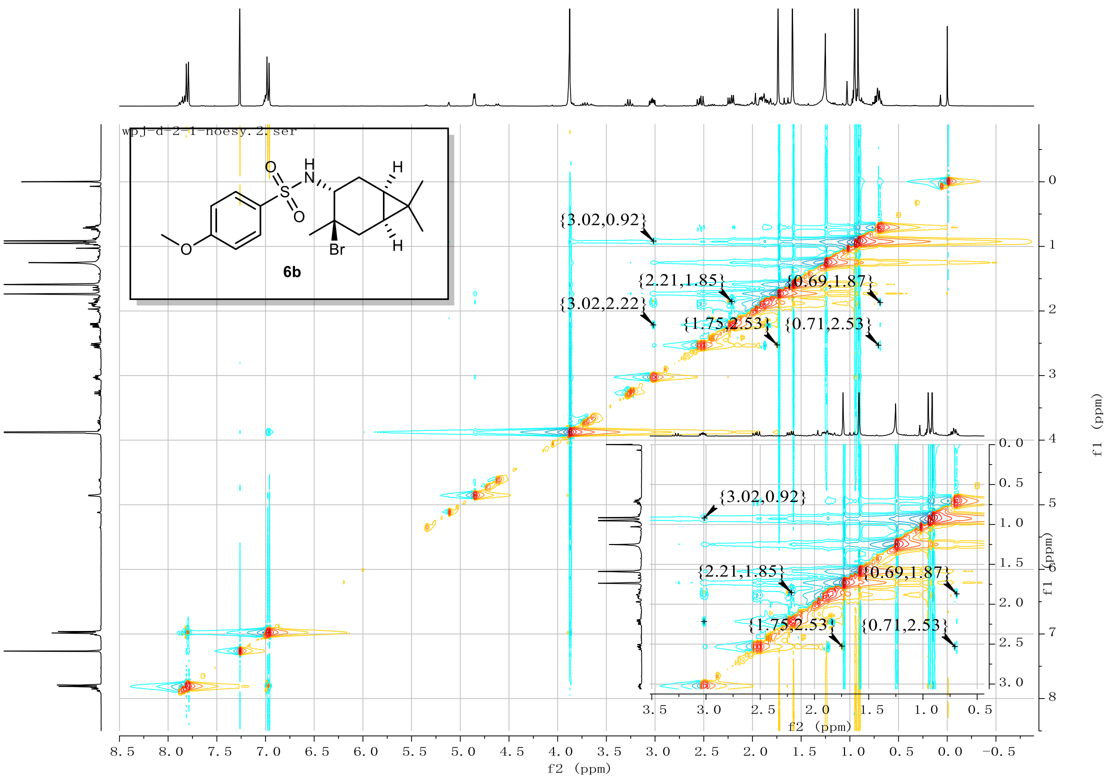

6c NOESY

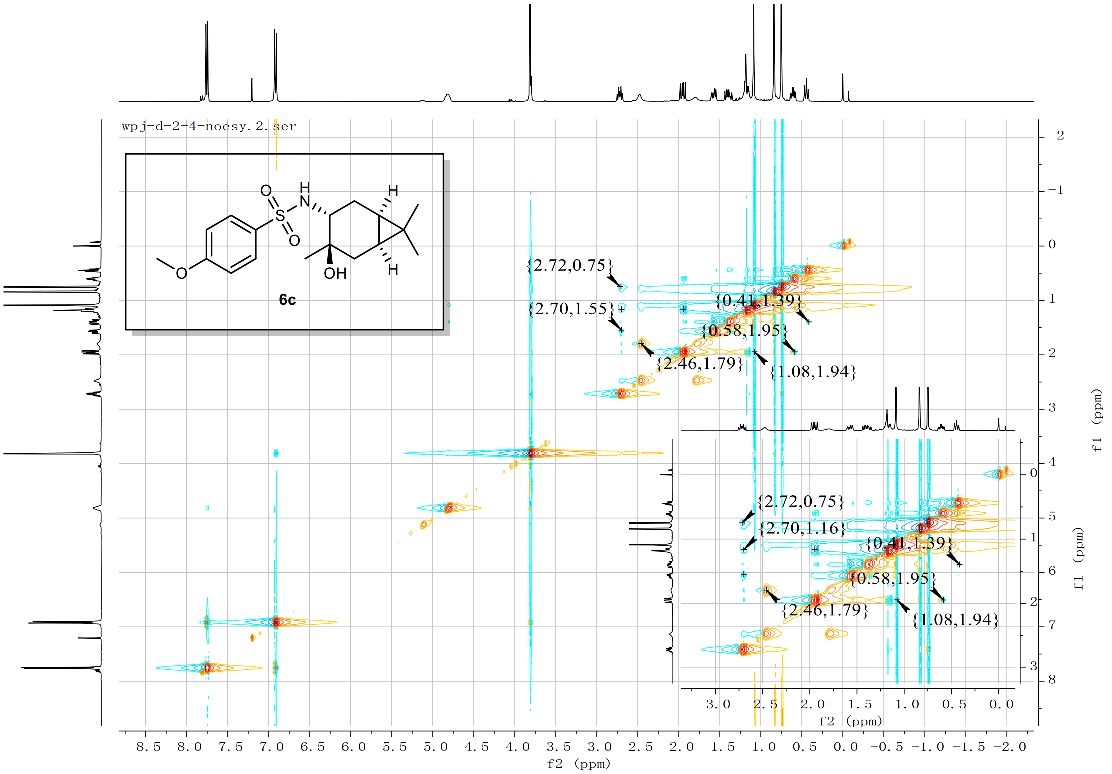

6d NOESY

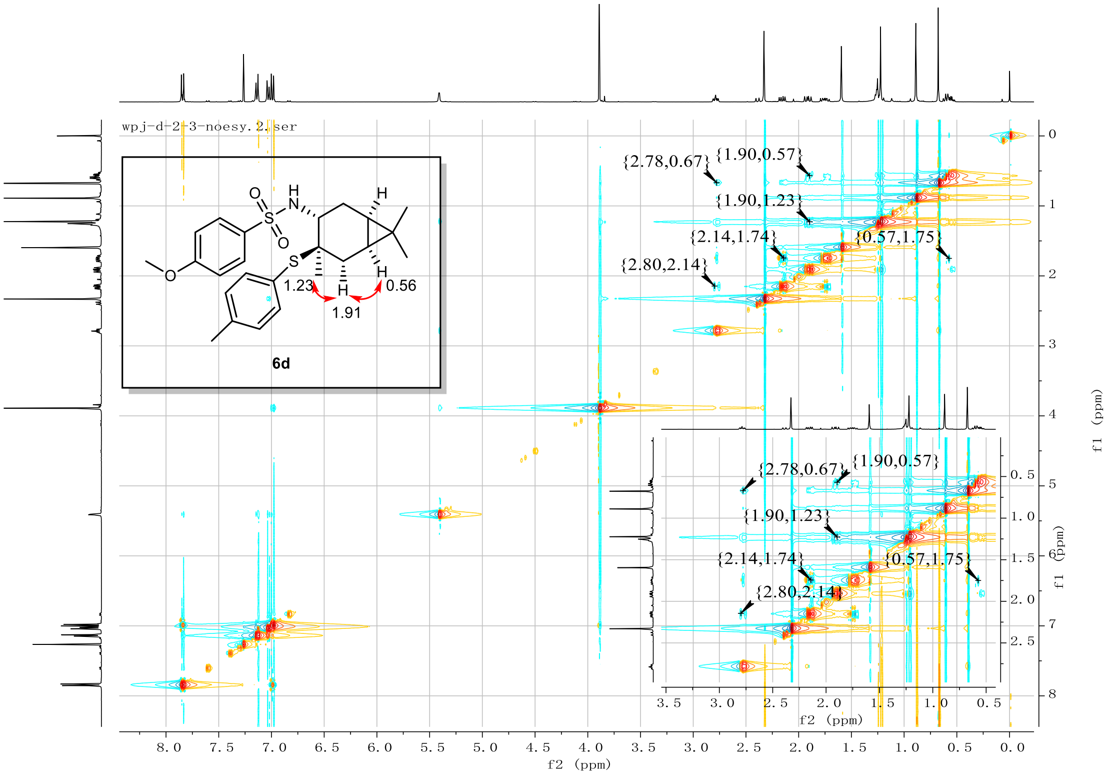

Characterization of Products

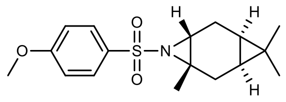

3a

(1*S*,3*S*,5*R*,7*R*)-4-((4-methoxyphenyl)sulfonyl)-3,8,8-trimethyl-4-azatricyclo[5.1.0.0^{3,5}] octane (3a):

The reaction was carried out following the procedure A using 4-methoxybenzenesulfonamide and 2 equivalent (+)-car-3-ene. Then 496.0 mg faint yellow oily liquid was obtained in 77% isolated yield, dr > 20:1.

^{1}H NMR (400 MHz, $CDCl_3$) δ 7.87 (d, *J* = 8.2 Hz, 2H), 6.97 (d, *J* = 8.3 Hz, 2H), 3.87 (s, 3H), 2.89 (s, 1H), 2.26 (dd, *J* = 15.9, 9.4 Hz, 1H), 2.00 (dd, *J* = 16.1, 9.6 Hz, 1H), 1.63 (s, 3H), 1.44 (dt, *J* = 16.1, 2.6 Hz, 1H), 1.25 (dd, *J* = 15.8, 2.9 Hz, 1H), 0.97 (s, 3H), 0.68 (s, 3H), 0.51 (td, *J* = 9.3, 2.7 Hz, 1H), 0.32 (td, *J* = 9.3, 2.9 Hz, 1H).

^{13}C NMR (101 MHz, $CDCl_3$) δ 162.77, 133.70, 129.02, 113.90, 55.54, 48.48, 45.74, 27.59, 25.20, 19.36, 17.61, 16.33, 16.08, 15.03, 13.62.

HRMS (ESI) exact mass calculated for $[M+H]^+$ ($C_{17}H_{24}NO_3S^+$) *m/z* 322.1471, found *m/z* 322.1469.

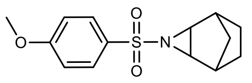

3b

3-((4-methoxyphenyl)sulfonyl)-3-azatricyclo[3.2.1.0^{2,4}]octane (3b):

The reaction was carried out following the procedure A using 4-methoxybenzenesulfonamide and 2 equivalent norbornene. Then 136.1 mg faint yellow solid was obtained in 24% isolated yield. dr > 20:1.

^{1}H NMR (400 MHz, $CDCl_3$) δ 7.94 – 7.78 (m, 2H), 7.03 – 6.93 (m, 2H), 3.87 (s, 3H), 2.88 (s, 2H), 2.43 (s, 2H), 1.53 – 1.38 (m, 3H), 1.27 – 1.20 (m, 2H), 0.74 (d, *J* = 10.1 Hz, 1H).

^{13}C NMR (101 MHz, $CDCl_3$) δ 163.29, 130.37, 129.76, 114.10, 55.60, 41.86, 35.79, 28.24, 25.58.

HRMS (ESI) exact mass calculated for $[M+H]^+$ ($C_{14}H_{18}NO_3S^+$) *m/z* 280.1002, found *m/z* 280.0996.

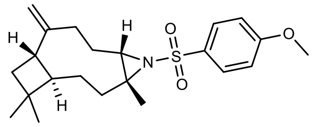

3c

(1R,4R,6S,10S)-5-((4-methoxyphenyl)sulfonyl)-4,12,12-trimethyl-9-methylene-5-azatricyclo[8.2.0.0^{4,6}]dodecane (3c):

The reaction was carried out following the procedure A using 4-methoxybenzenesulfonamide and 2 equivalent (-)-*β*-caryophyllene. Then 434.5 mg faint yellow oily liquid was obtained in 56% isolated yield. dr > 20:1.

^{1}H NMR (400 MHz, $CDCl_3$) δ 7.89 – 7.80 (m, 2H), 7.00 – 6.93 (m, 2H), 4.87 (d, *J* = 60.6 Hz, 2H), 3.86 (s, 3H), 3.11 – 3.03 (m, 1H), 2.62 (q, *J* = 10.0 Hz, 1H), 2.36 – 2.25 (m, 1H), 2.20 – 1.92 (m, 4H), 1.85 (t, *J* = 9.8 Hz, 1H), 1.75 – 1.59 (m, 3H), 1.54 – 1.41 (m, 1H), 1.38 – 1.28 (m, 1H), 1.20 (s, 3H), 1.00 (d, *J* = 5.5 Hz, 6H).
^{13}C NMR (101 MHz, $CDCl_3$) δ 162.85, 151.30, 133.53, 129.15, 129.00, 113.94, 112.88, 55.57, 54.24, 52.15, 49.52, 48.88, 39.53, 34.45, 34.41, 30.13, 29.86, 29.65, 27.37, 21.45, 18.84.
HRMS (ESI) exact mass calculated for $[M+H]^+$ ($C_{22}H_{32}NO_3S^+$) *m/z* 390.2097, found *m/z* 390.2092.

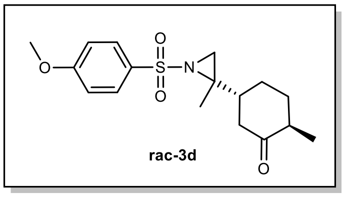

Racemic-(2*R*,5*R*)-5-(1-((4-methoxyphenyl)sulfonyl)-2-methylaziridin-2-yl)-2-methylcyclohexan-1-one (rac-3d):
The reaction was carried out following the procedure A using 4-methoxybenzenesulfonamide and 2 equivalent (+)-dihydrocarvone. Then 411.68 mg colorless oily liquid was obtained in 61% isolated yield.
^{1}H NMR (400 MHz, $CDCl_3$) δ 7.90 – 7.80 (m, 2H), 7.02 – 6.91 (m, 2H), 3.85 (s, 3H), 2.59 (d, *J* = 7.7 Hz, 1H), 2.42 – 2.27 (m, 2H), 2.25 – 2.15 (m, 2H), 2.14 – 2.04 (m, 1H), 1.95 – 1.86 (m, 1H), 1.67 (d, *J* = 12.8 Hz, 3H), 1.65 – 1.57 (m, 2H), 1.33 – 1.23 (m, 1H), 0.98 (d, *J* = 6.5 Hz, 3H).
^{13}C NMR (101 MHz, $CDCl_3$) δ 211.53, 211.38, 163.18, 163.17, 132.23, 132.19, 129.48, 129.47, 114.01, 113.99, 55.58, 51.91, 51.56, 47.25, 46.98, 44.66, 44.58, 43.90, 43.41, 40.35, 40.06, 34.07, 34.03, 27.59, 27.36, 14.88, 14.49, 14.17, 14.15.
HRMS (ESI) exact mass calculated for $[M+H]^+$ ($C_{17}H_{24}NO_4S^+$) *m/z* 338.1421, found *m/z* 338.1409.

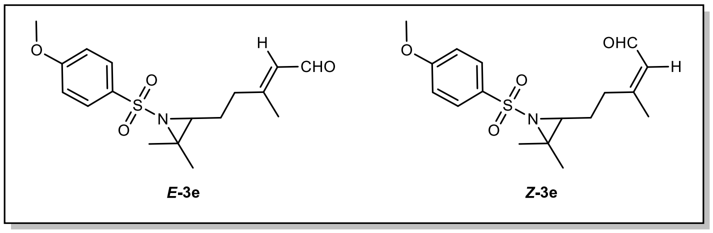

(*E*)-5-(1-((4-methoxyphenyl)sulfonyl)-3,3-dimethylaziridin-2-yl)-3-methylpent-2-enal and (*Z*)-5-(1-((4-methoxyphenyl)sulfonyl)-3,3-dimethylaziridin-2-yl)-3-methylpent-2-enal (mixture, *E*-3e:*Z*-3e=1:1) (3e):
The reaction was carried out following the procedure A using 4-methoxybenzenesulfonamide and 2 equivalent citral. Then 341.2 mg faint yellow oily liquid was obtained in 51% isolated yield. The ratio of different products is 1:1 and determined by isolated.
(*Z*)-5-(1-((4-methoxyphenyl)sulfonyl)-3,3-dimethylaziridin-2-yl)-3-methylpent-2-enal
^{1}H NMR (400 MHz, $CDCl_3$) δ 9.59 (d, *J* = 8.0 Hz, 1H), 7.93 – 7.83 (m, 2H), 7.04 – 6.94 (m, 2H), 5.83 – 5.74 (m, 1H), 3.88 (s, 3H), 2.83 (dd, *J* = 8.3, 5.0 Hz, 1H), 2.48 – 2.32 (m, 2H), 1.87 (d, *J* = 1.2 Hz, 3H), 1.84 – 1.73 (m, 1H), 1.70 (s, 3H), 1.44 (m, 1H), 1.29 (s, 3H).
^{13}C NMR (101 MHz, $CDCl_3$) δ 190.16, 163.25, 162.13, 132.68, 129.61, 128.52, 114.03, 55.70, 51.77, 51.66, 30.50, 27.62, 24.82, 21.27, 21.13.

HRMS (ESI) exact mass calculated for [M+H]$^+$ ($C_{17}H_{24}NO_4S^+$) *m/z* 338.1421, found *m/z* 338.1418.

(*E*)-5-(1-((4-methoxyphenyl)sulfonyl)-3,3-dimethylaziridin-2-yl)-3-methylpent-2-enal

^{1}H NMR (400 MHz, $CDCl_3$) δ 9.93 (d, *J* = 7.9 Hz, 1H), 7.92 – 7.83 (m, 2H), 7.02 – 6.94 (m, 2H), 5.76 – 5.68 (m, 1H), 3.87 (s, 3H), 2.80 (dd, *J* = 8.3, 5.0 Hz, 1H), 2.16 – 2.06 (m, 1H), 2.05 (d, *J* = 1.0 Hz, 3H), 2.01 – 1.92 (m, 1H), 1.77 – 1.70 (m, 1H), 1.69 (s, 3H), 1.51 – 1.40 (m, 1H), 1.28 (s, 3H).

^{13}C NMR (101 MHz, $CDCl_3$) δ 190.95, 163.13, 161.79, 132.65, 129.58, 127.18, 113.95, 55.63, 51.69, 51.35, 37.91, 25.60, 21.22, 21.03, 17.60.

HRMS (ESI) exact mass calculated for [M+H]$^+$ ($C_{17}H_{24}NO_4S^+$) *m/z* 338.1421, found *m/z* 338.1416.

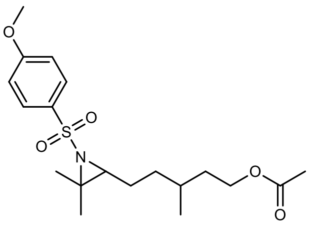

3f

5-(1-((4-methoxyphenyl)sulfonyl)-3,3-dimethylaziridin-2-yl)-3-methylpentyl acetate (3f):

The reaction was carried out following the procedure A using 4-methoxybenzenesulfonamide and 2 equivalent (±)-citronellyl acetate. Then 501.5 mg colorless oily liquid was obtained in 65% isolated yield.

^{1}H NMR (400 MHz, $CDCl_3$) δ 7.87 – 7.82 (m, 2H), 6.98 – 6.93 (m, 2H), 4.03 – 3.91 (m, 2H), 3.85 (s, 3H), 2.75 (ddd, *J* = 7.7, 5.3, 1.7 Hz, 1H), 2.01 (d, *J* = 1.7 Hz, 3H), 1.68 (d, *J* = 1.2 Hz, 3H), 1.56 – 1.38 (m, 3H), 1.32 (dd, *J* = 13.7, 6.8 Hz, 1H), 1.26 (d, *J* = 0.8 Hz, 3H), 1.24 – 1.12 (m, 1H), 1.11 – 0.84 (m, 2H), 0.77 (d, *J* = 6.4 Hz, 3H).

^{13}C NMR (101 MHz, $CDCl_3$) δ 171.04, 162.95, 132.93, 129.49, 113.79, 62.54, 62.49, 55.53, 52.67, 52.54, 51.68, 51.61, 35.12, 34.26, 29.32, 29.20, 25.25, 25.02, 21.20, 21.18, 21.16, 21.13, 20.94, 19.16, 18.98.

HRMS (ESI) exact mass calculated for [M+H]$^+$ ($C_{19}H_{30}NO_5S^+$) *m/z* 384.1839, found *m/z* 384.1832.

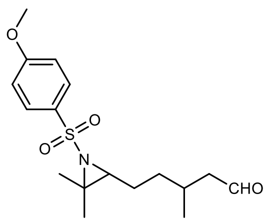

3g

5-(1-((4-methoxyphenyl)sulfonyl)-3,3-dimethylaziridin-2-yl)-3-methylpentanal (3g):

The reaction was carried out following the procedure A using 4-methoxybenzenesulfonamide and 2 equivalent (±)-citronellal. Then 278.1 mg colorless oily liquid was obtained in 41% isolated yield.

^{1}H NMR (400 MHz, $CDCl_3$) δ 9.68 (dt, *J* = 12.4, 2.2 Hz, 1H), 7.95 – 7.84 (m, 2H), 7.05 – 6.94 (m, 2H), 3.91 (d, *J* = 1.0 Hz, 3H), 2.87 – 2.75 (m, 1H), 2.31 – 2.12 (m, 2H), 2.06 – 1.91 (m, 1H),

1.73 (d, J = 2.7 Hz, 3H), 1.61 – 1.47 (m, 1H), 1.31 (s, 3H), 1.28 – 1.21 (m, 1H), 1.18 – 1.08 (m, 1H), 1.06 – 0.96 (m, 1H), 0.88 (dd, J = 6.7, 1.6 Hz, 3H).
^{13}C NMR (101 MHz, $CDCl_3$) δ 202.33, 202.22, 163.01, 132.84, 129.55, 129.54, 113.85, 55.59, 52.40, 52.23, 51.70, 51.65, 50.73, 50.70, 34.37, 34.31, 27.64, 27.56, 25.36, 25.21, 21.19, 21.19, 21.16, 21.14, 19.61, 19.56, 0.99.
HRMS (ESI) exact mass calculated for $[M+H]^+$ ($C_{17}H_{26}NO_4S^+$) *m/z* 340.1577, found *m/z* 340.1571.

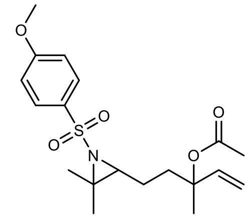

3h

5-(1-((4-methoxyphenyl)sulfonyl)-3,3-dimethylaziridin-2-yl)-3-methylpent-1-en-3-yl acetate (3h):
The reaction was carried out following the procedure A using 4-methoxybenzenesulfonamide and 2 equivalent (±)-linalyl acetate. Then 518.1 mg colorless oily liquid was obtained in 68% isolated yield.
^{1}H NMR (400 MHz, $CDCl_3$) δ 7.90 – 7.82 (m, 2H), 7.01 – 6.93 (m, 2H), 5.91 – 5.69 (m, 1H), 5.11 – 4.94 (m, 2H), 3.86 (d, J = 1.0 Hz, 3H), 2.82 – 2.71 (m, 1H), 1.96 (d, J = 3.3 Hz, 3H), 1.85 – 1.75 (m, 1H), 1.69 (d, J = 1.7 Hz, 3H), 1.48 – 1.42 (m, 1H), 1.41 (s, 2H), 1.38 (s, 1H), 1.37 – 1.29 (m, 2H), 1.26 (d, J = 3.2 Hz, 3H).
^{13}C NMR (101 MHz, $CDCl_3$) δ 169.76, 163.08, 141.35, 140.99, 132.92, 129.65, 113.91, 113.89, 113.54, 113.41, 82.20, 82.02, 55.64, 52.17, 51.88, 51.87, 37.34, 37.32, 23.70, 23.40, 22.34, 22.32, 22.11, 22.08, 21.20, 21.18.
HRMS (ESI) exact mass calculated for $[M+H]^+$ ($C_{19}H_{28}NO_5S^+$) *m/z* 382.1683, found *m/z* 382.1672

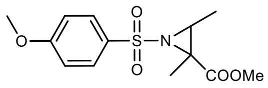

3i

methyl 1-((4-methoxyphenyl)sulfonyl)-2,3-dimethylaziridine-2-carboxylate (3i):
The reaction was carried out following the procedure B using 4-methoxybenzenesulfonamide and 4 equivalent methyl angelate. Then 196.2 mg colorless oily liquid was obtained in 33% isolated yield, dr = 2:1.
^{1}H NMR (400 MHz, $CDCl_3$) δ 7.95 – 7.80 (m, 2H), 7.03 – 6.91 (m, 2H), 3.86 (s, 3H), 3.80 (s, 2H), 3.73 (s, 1H), 3.32 (dq, J = 225.1, 5.8 Hz, 1H), 1.90 (s, 1H), 1.45 (s, 2H), 1.24 (d, J = 5.8 Hz, 2H), 1.16 (d, J = 5.8 Hz, 1H).
^{13}C NMR (101 MHz, $CDCl_3$) δ 168.70, 168.58, 163.33, 163.24, 132.03, 131.67, 129.50, 129.48, 114.11, 114.00, 55.55, 52.98, 52.58, 52.43, 52.03, 46.83, 43.98, 15.81, 15.37, 13.36, 12.41.
HRMS (ESI) exact mass calculated for $[M+H]^+$ ($C_{13}H_{18}NO_5S^+$) *m/z* 300.0900, found *m/z* 300.0899.

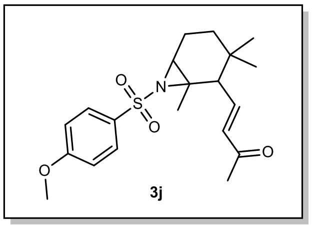

(*E*)-4-(7-((4-methoxyphenyl)sulfonyl)-1,3,3-trimethyl-7-azabicyclo[4.1.0]heptan-2-yl)but-3-en-2-one (3j):

The reaction was carried out following the procedure A using 4-methoxybenzenesulfonamide and 2 equivalent *a*-ionone. Then 425.6 mg faint yellow oily liquid was obtained in 55% isolated yield .

^{1}H NMR (400 MHz, $CDCl_3$) δ 7.83 – 7.78 (m, 2H), 6.98 – 6.93 (m, 2H), 6.61 (dd, *J* = 16.0, 10.0 Hz, 1H), 5.98 (d, *J* = 16.0 Hz, 1H), 3.85 (s, 3H), 3.17 (d, *J* = 3.1 Hz, 1H), 2.19 (s, 3H), 1.97 (d, *J* = 10.0 Hz, 1H), 1.92 – 1.82 (m, 1H), 1.76 – 1.67 (m, 1H), 1.64 (s, 3H), 1.33 – 1.21 (m, 1H), 0.98 (dt, *J* = 13.3, 5.2 Hz, 1H), 0.85 (s, 3H), 0.69 (s, 3H).

^{13}C NMR (101 MHz, $CDCl_3$) δ 198.47, 163.03, 146.67, 133.06, 132.57, 129.16, 114.07, 55.56, 53.51, 52.37, 46.15, 31.30, 29.08, 28.14, 26.59, 26.53, 20.78, 20.24.

HRMS (ESI) exact mass calculated for $[M+H]^+$ ($C_{20}H_{28}NO_4S^+$) *m/z* 378.1734, found *m/z* 378.1723.

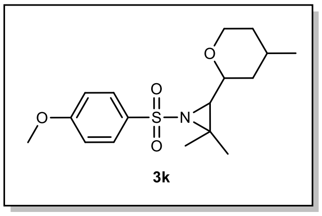

1-((4-methoxyphenyl)sulfonyl)-2,2-dimethyl-3-(4-methyltetrahydro-2H-pyran-2-yl) aziridine (3k):

The reaction was carried out following the procedure A using 4-methoxybenzenesulfonamide and 2 equivalent 4-methyl-2-(2-methylprop-1-enyl)tetrahydropyran. Then 417.5 mg faint yellow oily liquid was obtained in 61% isolated yield.

^{1}H NMR (400 MHz, $CDCl_3$) δ 7.96 – 7.82 (m, 2H), 7.01 – 6.85 (m, 2H), 3.85 (d, *J* = 6.4 Hz, 4H), 3.33 – 3.17 (m, 1H), 3.08 – 2.91 (m, 1H), 2.91 – 2.71 (dd, *J* = 50.8, 8.4 Hz, 1H), 1.71 (d, *J* = 6.1 Hz, 3H), 1.57 – 1.44 (m, 2H), 1.32 (d, *J* = 8.1 Hz, 3H), 1.28 – 0.97 (m, 3H), 0.93 (d, *J* = 6.3 Hz, 2H), 0.77 (d, *J* = 6.4 Hz, 1H).

^{13}C NMR (101 MHz, $CDCl_3$) δ 163.13, 162.75, 132.98, 132.28, 129.82, 129.47, 113.75, 113.47, 75.90, 74.95, 67.93, 67.52, 55.56, 55.46, 53.93, 51.72, 49.89, 38.64, 37.29, 34.05, 29.63, 29.33, 22.18, 21.95, 21.82, 21.48, 21.25, 20.91.

HRMS (ESI) exact mass calculated for $[M+H]^+$ ($C_{17}H_{26}NO_4S^+$) *m/z* 340.1577, found *m/z* 340.1569.

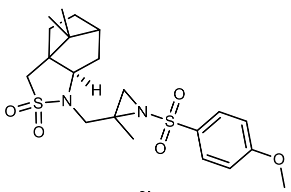

3l

(7a*R*)-1-((1-((4-methoxyphenyl)sulfonyl)-2-methylaziridin-2-yl)methyl)-8,8-dimethylhexahydro-3*H*-3a,6-methanobenzo[*c*]isothiazole 2,2-dioxide (3l):

The reaction was carried out following the procedure A using 4-methoxybenzenesulfonamide and 2 equivalent (*2R*)-Bornane-10,2-sultam derivative. Then 464.2mg faint yellow oily liquid was obtained in 51% isolated yield, dr = 2:1.

^{1}H NMR (400 MHz, $CDCl_3$) δ 7.87 – 7.80 (m, 2H), 7.02 – 6.94 (m, 3H), 3.87 (d, *J* = 3.7 Hz, 5H), 3.52 (d, *J* = 15.8 Hz, 1H), 3.16 (d, *J* = 2.3 Hz, 2H), 3.14 – 2.94 (m, 5H), 2.85 (d, *J* = 15.8 Hz, 1H), 2.56 (dd, 2H), 2.29 – 2.09 (m, 2H), 1.98 – 1.77 (m, 5H), 1.75 (d, *J* = 9.4 Hz, 3H), 1.72 – 1.59 (m, 2H), 1.44 – 1.34 (m, 2H), 1.30 – 1.23 (m, 2H), 1.20 (s, 3H), 0.93 (s, 3H), 0.82 (s, 2H), 0.72 (s, 2H).

^{13}C NMR (101 MHz, $CDCl_3$) δ 163.19, 163.16, 132.25, 132.15, 129.42, 129.33, 114.02, 113.91, 68.81, 68.52, 55.62, 50.78, 49.96, 49.74, 49.50, 48.93, 48.25, 48.05, 47.70, 47.45, 47.26, 44.53, 44.37, 41.51, 38.67, 35.57, 34.88, 32.17, 32.10, 26.89, 26.82, 20.32, 19.91, 19.86, 19.60, 16.83, 16.53.

HRMS (ESI) exact mass calculated for $[M+H]^+$ ($C_{21}H_{31}N_2O_5S_2^+$) *m/z* 455.1669, found *m/z* 455.1665

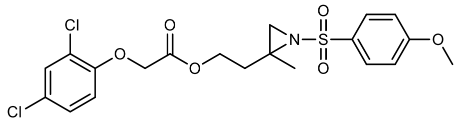

3m

2-(1-((4-methoxyphenyl)sulfonyl)-2-methylaziridin-2-yl)ethyl 2-(2,4-dichlorophenoxy) acetate (3m):

The reaction was carried out following the procedure A using 4-methoxybenzenesulfonamide and 2 equivalent b-selektonone derivative. Then 291.9 mg faint yellow oily liquid was obtained in 31% isolated yield.

^{1}H NMR (400 MHz, $CDCl_3$) δ 7.89 – 7.81 (m, 2H), 7.39 (d, *J* = 2.5 Hz, 1H), 7.14 (dd, *J* = 8.8, 2.5 Hz, 1H), 7.03 – 6.94 (m, 2H), 6.79 (d, *J* = 8.8 Hz, 1H), 4.72 (d, *J* = 1.2 Hz, 2H), 4.43 – 4.26 (m, 2H), 3.87 (s, 3H), 2.53 (s, 1H), 2.26 (s, 1H), 2.16 – 2.05 (m, 1H), 1.97 – 1.86 (m, 1H), 1.66 (s, 3H).

^{13}C NMR (101 MHz, $CDCl_3$) δ 167.99, 163.25, 152.26, 132.18, 130.28, 129.47, 127.57, 127.00, 124.04, 114.54, 114.09, 66.19, 62.23, 55.63, 47.73, 41.04, 36.22, 18.81.

HRMS (ESI) exact mass calculated for $[M+H]^+$ ($C_{20}H_{22}NO_6SCl_2^+$) *m/z* 474.0539, found *m/z* 474.0540.

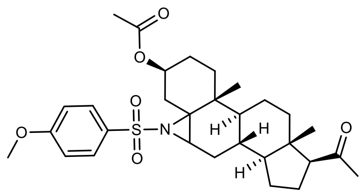

3n

(3*S*,6a*S*,6b*S*,9*S*,9a*S*,11a*S*,11b*R*)-9-acetyl-5-((4-methoxyphenyl)sulfonyl)-9a,11b-dimethylhexadecahydro-2*H*-cyclopenta[1,2]phenanthro[8a,9-*b*]azirin-3-yl acetate (3n):
The reaction was carried out following the procedure A using 4-methoxybenzenesulfonamide and 2 equivalent pregnenolone acetate. Then 683.3 mg white solid was obtained in 63% isolated yield. dr > 20:1.
^{1}H NMR (400 MHz, $CDCl_3$) δ 7.89 – 7.78 (m, 2H), 7.00 – 6.90 (m, 2H), 4.79 – 4.86 (m, 1H), 3.87 (s, 3H), 3.14 (s, 1H), 2.58 – 0.64 (m, 30H), 0.52 (s, 3H).
^{13}C NMR (101 MHz, $CDCl_3$) δ 209.15, 170.51, 162.93, 133.56, 128.99, 113.88, 70.39, 63.44, 56.69, 56.06, 55.59, 49.58, 48.26, 43.69, 38.69, 36.20, 34.23, 32.34, 31.45, 30.41, 29.90, 26.54, 24.27, 22.67, 22.01, 21.28, 20.61, 13.06.
HRMS (ESI) exact mass calculated for $[M+H]^+$ ($C_{30}H_{42}NO_6S^+$) *m/z* 544.2727, found *m/z* 544.2712.

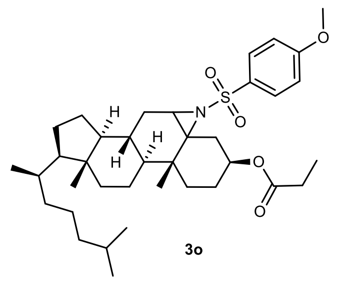

3o

(3*S*,6a*S*,6b*S*,9*R*,9a*R*,11a*S*,11b*R*)-5-((4-methoxyphenyl)sulfonyl)-9a,11b-dimethyl-9-((*R*)-6-methylheptan-2-yl)hexadecahydro-2*H*-cyclopenta[1,2]phenanthro[8a,9-*b*]azirin-3-yl propionate (3o):
The reaction was carried out following the procedure A using 4-methoxybenzenesulfonamide and 2 equivalent cholesterol propionates. Then 605.4 mg white solid was obtained in 48% isolated yield. dr > 20:1.
^{1}H NMR (400 MHz, $CDCl_3$) δ 7.83 (d, *J* = 8.9 Hz, 2H), 6.95 (d, *J* = 8.9 Hz, 2H), 5.02 – 4.84 (m, 1H), 3.86 (s, 3H), 3.12 (s, 1H), 2.56 – 2.43 (m, 1H), 2.35 – 0.59 (m, 44H), 0.56 (s, 3H).
^{13}C NMR (101 MHz, $CDCl_3$) δ 173.92, 162.87, 133.79, 129.03, 113.87, 70.30, 56.95, 56.06, 55.98, 55.61, 49.88, 48.24, 42.18, 39.72, 39.48, 36.18, 36.08, 35.70, 34.13, 32.41, 30.50, 29.92, 28.09, 28.00, 27.82, 26.63, 24.13, 23.77, 22.82, 22.56, 22.06, 20.68, 18.64, 11.75, 9.11.
HRMS (ESI) exact mass calculated for $[M+H]^+$ ($C_{37}H_{58}NO_5S^+$) *m/z* 628.4030, found *m/z* 628.4032.

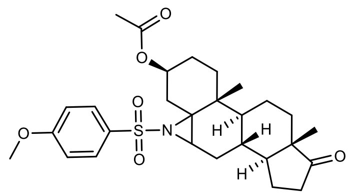

3p

(3*S*,6a*S*,6b*S*,9*S*,9a*S*,11a*S*,11b*R*)-5-((4-methoxyphenyl)sulfonyl)-9a,11b-dimethyl-9-oxohexadecahydro-2*H*-cyclopenta[1,2]phenanthro[8a,9-*b*]azirin-3-yl acetate (3p):

The reaction was carried out following the procedure A using 4-methoxybenzenesulfonamide and 2 equivalent dehydroisoandrosterone-3-acetate. Then 738.9 mg white solid was obtained in 72% isolated yield . dr > 20:1.

^{1}H NMR (400 MHz, $CDCl_3$) δ 7.88 – 7.80 (m, 2H), 7.01 – 6.92 (m, 2H), 4.95 – 4.84 (m, 1H), 3.86 (s, 3H), 3.18 (s, 1H), 2.51 – 1.00 (m, 24H), 0.76 (s, 3H), 0.69 (dt, *J* = 11.8, 6.0 Hz, 1H).

^{13}C NMR (101 MHz, $CDCl_3$) δ 170.43, 162.92, 133.31, 129.02, 113.86, 70.24, 56.59, 55.56, 50.85, 49.19, 48.41, 47.22, 36.31, 35.52, 34.06, 32.12, 31.28, 29.50, 29.36, 26.40, 21.55, 21.21, 20.54, 13.36.

HRMS (ESI) exact mass calculated for $[M+H]^+$ ($C_{28}H_{38}NO_6S^+$) *m/z* 516.2414, found *m/z* 516.2407.

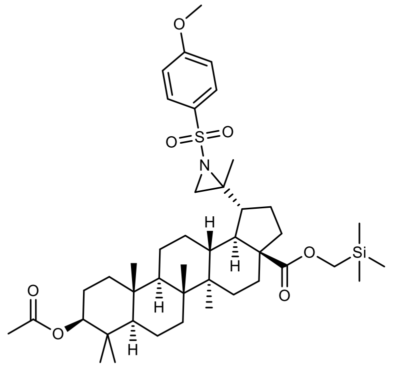

3q

(trimethylsilyl)methyl (1*R*,3a*S*,5a*R*,5b*R*,7a*R*,9*S*,11a*R*,11b*R*,13a*R*,13b*S*)-9-acetoxy-1-((*S*)-1-((4-methoxyphenyl)sulfonyl)-2-methylaziridin-2-yl)-5a,5b,8,8,11a-pentamethylicosahydro-3a*H*-cyclopenta[*a*]chrysene-3a-carboxylate (3q):

The reaction was carried out following the procedure A using 4-methoxybenzenesulfonamide and 2 equivalent betulinic acid derivative. Then 632.1 mg white solid was obtained in 41% isolated yield. dr > 20:1.

^{1}H NMR (400 MHz, $CDCl_3$) δ 7.89 – 7.78 (m, 2H), 7.01 – 6.91 (m, 2H), 4.53 – 4.42 (m, 1H), 3.87 (d, *J* = 2.6 Hz, 3H), 3.68 (s, 2H), 2.66 (d, *J* = 11.7 Hz, 1H), 2.22 (d, *J* = 5.3 Hz, 2H), 2.17 – 0.70 (m, 44H), 0.07 (d, *J* = 2.5 Hz, 9H).

^{13}C NMR (101 MHz, CDCl3) δ 176.71, 176.55, 171.01, 162.95, 133.09, 129.42, 129.35, 113.94, 113.86, 80.85, 57.96, 57.50, 57.05, 55.59, 55.58, 55.37, 55.08, 54.87, 50.53, 50.28, 49.14, 47.69, 42.52, 42.44, 40.77, 40.69, 38.44, 37.91, 37.77, 37.43, 37.10, 37.07, 36.72, 34.22, 32.30, 29.68, 29.65, 29.34, 29.30, 29.06, 27.92, 27.25, 26.75, 23.66, 21.30, 20.95, 18.13, 16.49, 16.47, 16.26, 16.15, 16.12, 16.00, 14.81, 14.75, 14.58, -3.02.

HRMS (ESI) exact mass calculated for $[M+H]^+$ ($C_{43}H_{68}NO_7SSi^+$) *m/z* 770.4480, found *m/z* 770.4480.

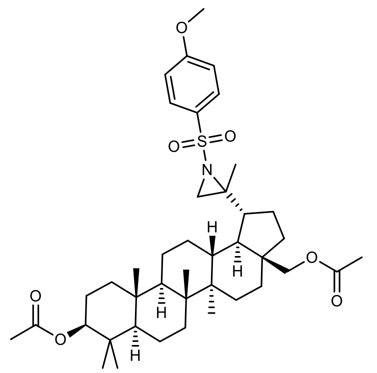

3r

((1*R*,3a*S*,5a*R*,5b*R*,7a*R*,9*S*,11a*R*,11b*R*,13a*R*,13b*S*)-9-acetoxy-1-((*S*)-1-((4-methoxyphenyl) sulfonyl)-2-methylaziridin-2-yl)-5a,5b,8,8,11a-pentamethylicosahydro-3a*H*-cyclopenta[*a*]chrysen-3a-yl) methyl acetate (3r):
The reaction was carried out following the procedure A using 4-methoxybenzenesulfonamide and 2 equivalent betulin-3,28-diactate. Then 639.7 mg white solid was obtained in 45% isolated yield . dr > 20:1.
^{1}H NMR (400 MHz, $CDCl_3$) δ 7.82 (t, *J* = 9.2 Hz, 2H), 7.03 – 6.91 (m, 2H), 4.54 – 4.43 (m, 1H), 4.22 – 4.10 (m, 1H), 3.86 (s, 3H), 3.69 (d, *J* = 11.0 Hz, 1H), 2.67 (d, *J* = 2.4 Hz, 1H), 2.18 (s, 1H), 2.04 (d, *J* = 2.6 Hz, 6H), 1.86 – 0.77 (m, 43H).
^{13}C NMR (101 MHz, $CDCl_3$) δ 171.51, 171.03, 163.04, 132.84, 129.39, 129.37, 114.23, 113.95, 80.82, 68.13, 62.39, 55.60, 55.29, 54.88, 50.06, 48.74, 48.19, 47.07, 42.76, 40.86, 38.39, 37.76, 37.01, 36.68, 34.06, 29.72, 29.68, 27.91, 26.74, 26.61, 23.63, 21.31, 20.99, 20.86, 18.11, 16.47, 16.11, 15.97, 14.76, 14.62.
HRMS (ESI) exact mass calculated for $[M+H]^+$ ($C_{41}H_{62}NO_7S^+$) *m/z* 712.4242, found *m/z* 712.4232.

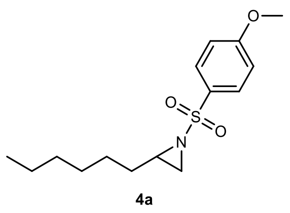

4a

2-hexyl-1-((4-methoxyphenyl)sulfonyl)aziridine (4a):
The reaction was carried out following the procedure B using 4-methoxybenzenesulfonamide and 4 equivalent oct-1-ene. Then 345.0 mg faint yellow oily liquid was obtained in 58% isolated yield.
^{1}H NMR (400 MHz, $CDCl_3$) δ 7.96 – 7.88 (m, 2H), 7.09 – 6.98 (m, 2H), 3.91 (s, 3H), 2.76 – 2.69 (m, 1H), 2.65 (d, *J* = 7.0 Hz, 1H), 2.08 (d, *J* = 4.5 Hz, 1H), 1.62 – 1.51 (m, 1H), 1.35 – 1.15 (m, 9H), 0.88 (t, *J* = 7.0 Hz, 3H).
^{13}C NMR (101 MHz, $CDCl_3$) δ 163.53, 130.11, 129.64, 114.14, 55.62, 40.42, 33.70, 31.58, 31.28, 28.65, 26.72, 22.42, 14.00.

HRMS (ESI) exact mass calculated for $[M+H]^+$ ($C_{15}H_{24}NO_3S^+$) *m/z* 298.1471, found *m/z* 298.1467.

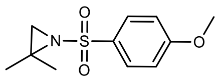

4b

1-((4-methoxyphenyl)sulfonyl)-2,2-dimethylaziridine (4b):
The reaction was carried out following the procedure B using 4-methoxybenzenesulfonamide and 6 equivalent isobutene (2.4M in THF). Then 217.2 mg white solid was obtained in 45% isolated yield.
^{1}H NMR (400 MHz, $CDCl_3$) δ 7.95 – 7.80 (m, 2H), 7.02 – 6.95 (m, 2H), 3.87 (s, 3H), 2.42 (s, 2H), 1.53 (s, 6H).
^{13}C NMR (101 MHz, $CDCl_3$) δ 163.12, 132.66, 129.47, 114.05, 55.66, 47.76, 41.90, 22.79.
HRMS (ESI) exact mass calculated for $[M+H]^+$ ($C_{11}H_{16}NO_3S$) *m/z* 242.0845, found *m/z* 242.0845.

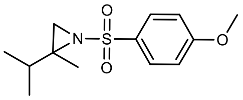

4c

2-isopropyl-1-((4-methoxyphenyl)sulfonyl)-2-methylaziridine (4c):
The reaction was carried out following the procedure B using 4-methoxybenzenesulfonamide and 4 equivalent 2,3-dimethyl-1-butene. Then 465.6 mg colorless oily liquid was obtained in 86% isolated yield .
^{1}H NMR (400 MHz, $CDCl_3$) δ 7.95 – 7.87 (m, 2H), 7.05 – 6.97 (m, 2H), 3.89 (s, 3H), 2.61 (s, 1H), 2.22 (s, 1H), 1.60 (s, 3H), 1.50 (p, *J* = 6.9 Hz, 1H), 1.00 (d, *J* = 6.8 Hz, 3H), 0.96 (d, *J* = 7.0 Hz, 3H).
^{13}C NMR (101 MHz, $CDCl_3$) δ 163.01, 132.69, 129.45, 113.92, 55.57, 54.66, 41.03, 36.03, 18.23, 13.23, 0.98.
HRMS (ESI) exact mass calculated for $[M+H]^+$ ($C_{13}H_{20}NO_3S^+$) *m/z* 270.1158, found *m/z* 270.1151.

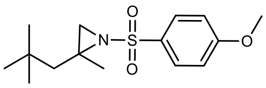

4d

1-((4-methoxyphenyl)sulfonyl)-2-methyl-2-neopentylaziridine (4d):
The reaction was carried out following the procedure B using 4-methoxybenzenesulfonamide and 2 equivalent 2,4,4-trimethyl-1-pentene. Then 352.2 mg colorless oily liquid was obtained in 59% isolated yield.
^{1}H NMR (400 MHz, $CDCl_3$) δ 7.92 – 7.80 (m, 2H), 7.02 – 6.92 (m, 2H), 3.86 (s, 3H), 2.53 (s, 1H), 2.23 (s, 1H), 1.73 (s, 3H), 1.59 (d, *J* = 3.7 Hz, 2H), 1.02 (s, 9H).
^{13}C NMR (101 MHz, $CDCl_3$) δ 163.00, 133.03, 129.35, 113.97, 55.58, 51.31, 50.12, 42.27, 31.37, 30.55, 20.81.

HRMS (ESI) exact mass calculated for $[M+H]^+$ ($C_{15}H_{24}NO_3S^+$) *m/z* 298.1471, found *m/z* 298.1466.

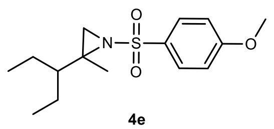

4e

1-((4-methoxyphenyl)sulfonyl)-2-methyl-2-(pentan-3-yl)aziridine (4e):
The reaction was carried out following the procedure B using 4-methoxybenzenesulfonamide and 2 equivalent 3-ethyl-2-methylpent-1-ene. Then 105.8 mg colorless oily liquid was obtained in 73% isolated yield.
^{1}H NMR (400 MHz, $CDCl_3$) δ 7.94 – 7.77 (m, 2H), 7.01 – 6.89 (m, 2H), 3.85 (s, 3H), 2.64 (s, 1H), 2.14 (s, 1H), 1.57 (s, 3H), 1.54 – 1.18 (m, 4H), 0.97 (t, *J* = 7.5 Hz, 3H), 0.88 (t, *J* = 7.5 Hz, 3H), 0.85 – 0.80 (m, 1H).
^{13}C NMR (101 MHz, $CDCl_3$) δ 162.97, 132.86, 129.36, 113.91, 55.54, 53.65, 50.31, 42.03, 24.81, 24.00, 12.99, 12.25, 12.12.
HRMS (ESI) exact mass calculated for $[M+H]^+$ ($C_{15}H_{24}NO_3S^+$) *m/z* 298.1471, found *m/z* 298.1466.

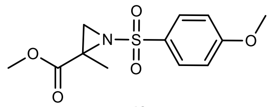

4f

methyl 1-((4-methoxyphenyl)sulfonyl)-2-methylaziridine-2-carboxylate (4f):
The reaction was carried out following the procedure B using 4-methoxybenzenesulfonamide and 4 equivalent methyl methacrylate. Then 154.4 mg colorless oily liquid was obtained in 27% isolated yield.
^{1}H NMR (400 MHz, $CDCl_3$) δ 7.96 – 7.84 (m, 2H), 7.03 – 6.93 (m, 2H), 3.87 (s, 3H), 3.74 (s, 3H), 2.77 (s, 1H), 2.70 (s, 1H), 1.89 (s, 3H).
^{13}C NMR (101 MHz, $CDCl_3$) δ 169.06, 163.55, 131.35, 129.87, 114.16, 55.66, 53.05, 46.41, 38.87, 15.10.
HRMS (ESI) exact mass calculated for $[M+H]^+$ ($C_{12}H_{16}NO_5S^+$) m/z 286.0744, found m/z 286.0744.

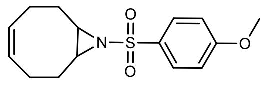

4g

(*Z*)-9-((4-methoxyphenyl)sulfonyl)-9-azabicyclo[6.1.0]non-4-ene (4g):
The reaction was carried out following the procedure B using 4-methoxybenzenesulfonamide and 4 equivalent 1,5-cyclooctadiene. Then 258.1 mg faint yellow oily liquid was obtained in 44% isolated yield.

^{1}H NMR (400 MHz, $CDCl_3$) δ 7.94 – 7.81 (m, 2H), 7.02 – 6.94 (m, 2H), 5.82 – 5.634 (m, 2H), 3.86 (s, 3H), 2.66 (dd, *J* = 7.0, 4.2 Hz, 2H), 2.39 – 2.27 (m, 2H), 2.25 – 2.15 (m, 2H), 2.15 – 2.06 (m, 2H), 1.58 (s, 2H).
^{13}C NMR (101 MHz, $CDCl_3$) δ 163.21, 131.74, 130.33, 129.46, 114.09, 55.60, 47.86, 25.98, 25.73.
HRMS (ESI) exact mass calculated for $[M+H]^+$ ($C_{15}H_{20}NO_3S^+$) *m/z* 294.1158, found *m/z* 294.1152.

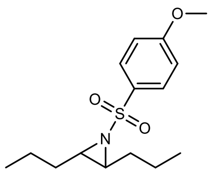

4ha
from *cis*-oct-4-ene

1-((4-methoxyphenyl)sulfonyl)-2,3-dipropylaziridine (4ha):
The reaction was carried out following the procedure B using 4-methoxybenzenesulfonamide and 4 equivalent *cis*-oct-4-ene. Then 345.1 mg colorless oily liquid was obtained in 58% isolated yield, dr = 2:1.
^{1}H NMR (400 MHz, $CDCl_3$) δ 7.99 – 7.86 (m, 2H), 7.06 – 6.96 (m, 2H), 3.90 (d, *J* = 2.4 Hz, 3H), 2.83 – 2.59 (m, 2H), 1.84 – 1.62 (m, 3H), 1.52 – 1.30 (m, 5H), 0.92 (dt, *J* = 9.5, 7.3 Hz, 6H).
^{13}C NMR (101 MHz, $CDCl_3$) δ 163.37, 163.05, 132.61, 130.09, 129.50, 114.00, 113.88, 55.57, 49.53, 44.91, 31.80, 28.72, 20.73, 20.56, 13.73, 13.69.
HRMS (ESI) exact mass calculated for $[M+H]^+$ ($C_{15}H_{24}NO_3S^+$) *m/z* 298.1471, found *m/z* 298.1469.

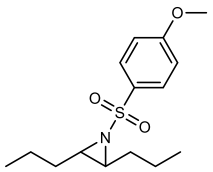

4hb
from *trans*-oct-4-ene

1-((4-methoxyphenyl)sulfonyl)-2,3-dipropylaziridine (4hb):
The reaction was carried out following the procedure B using 4-methoxybenzenesulfonamide and 4 equivalent *trans*-oct-4-ene. Then 315.8 mg colorless oily liquid was obtained in 53% isolated yield, dr = 3:1.
^{1}H NMR (400 MHz, $CDCl_3$) δ 7.87 (d, *J* = 8.7 Hz, 2H), 7.03 – 6.89 (m, 2H), 3.87 (s, 3H), 2.80 – 2.58 (m, 2H), 1.81 – 1.57 (m, 4H), 1.43 – 1.29 (m, 4H), 0.90 (t, *J* = 7.4 Hz, 6H).
^{13}C NMR (101 MHz, $CDCl_3$) δ 163.38, 163.06, 132.64, 130.11, 129.52, 113.90, 55.58, 49.55, 44.93, 31.82, 28.74, 20.75, 20.58, 13.75, 13.71.
HRMS (ESI) exact mass calculated for $[M+H]^+$ ($C_{15}H_{24}NO_3S^+$) *m/z* 298.1471, found *m/z* 298.1468.

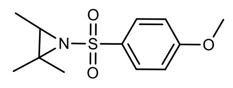

1-((4-methoxyphenyl)sulfonyl)-2,2,3-trimethylaziridine (4i):
The reaction was carried out following the procedure B using 4-methoxybenzenesulfonamide and 2 equivalent 2-methylbut-2-ene. Then 290.7 mg white solid was obtained in 57% isolated yield.
^{1}H NMR (400 MHz, $CDCl_3$) δ 7.88 – 7.81 (m, 2H), 6.99 – 6.92 (m, 2H), 3.85 (s, 3H), 2.92 (q, *J* = 5.8 Hz, 1H), 1.68 (s, 3H), 1.26 (s, 3H), 1.12 (d, *J* = 5.9 Hz, 3H).
^{13}C NMR (101 MHz, $CDCl_3$) δ 162.85, 133.35, 129.03, 113.93, 55.53, 51.60, 47.68, 20.96, 20.87, 12.84.
HRMS (ESI) exact mass calculated for $[M+H]^+$ ($C_{12}H_{18}NO_3S^+$) *m/z* 256.1002, found *m/z* 256.0999.

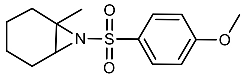

7-((4-methoxyphenyl)sulfonyl)-1-methyl-7-azabicyclo[4.1.0]heptane (4j):
The reaction was carried out following the procedure B using 4-methoxybenzenesulfonamide and 2 equivalent 1-methyl-1-cyclohexene. Then 348.8 mg colorless oily liquid was obtained in 62% isolated yield .
^{1}H NMR (400 MHz, $CDCl_3$) δ 7.91 – 7.82 (m, 2H), 7.00 – 6.90 (m, 2H), 3.85 (s, 3H), 3.01 (d, *J* = 5.2 Hz, 1H), 2.02 (dt, *J* = 13.3, 4.8 Hz, 1H), 1.87 – 1.73 (m, 1H), 1.68 (s, 3H), 1.58 – 1.44 (m, 2H), 1.43 – 1.31 (m, 2H), 1.30 – 1.21 (m, 1H), 1.16 – 1.00 (m, 1H).
^{13}C NMR (101 MHz, $CDCl_3$) δ 162.74, 133.58, 128.98, 113.85, 55.50, 51.05, 47.06, 32.02, 22.79, 20.31, 19.74, 19.48.
HRMS (ESI) exact mass calculated for $[M+H]^+$ ($C_{14}H_{20}NO_3S^+$) *m/z* 282.1158, found *m/z* 282.1151.

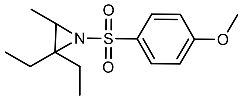

2,2-diethyl-1-((4-methoxyphenyl)sulfonyl)-3-methylaziridine (4k):
The reaction was carried out following the procedure B using 4-methoxybenzenesulfonamide and 2 equivalent 3-ethyl-2-pentene. Then 424.9 mg colorless oily liquid was obtained in 75% isolated yield .
^{1}H NMR (400 MHz, $CDCl_3$) δ 7.91 – 7.82 (m, 2H), 7.01 – 6.93 (m, 2H), 3.86 (s, 3H), 2.94 (q, *J* = 5.9 Hz, 1H), 2.22 – 2.10 (m, 1H), 2.03 – 1.91 (m, 1H), 1.65 – 1.54 (m, 1H), 1.50 – 1.37 (m, 1H), 1.15 (d, *J* = 5.9 Hz, 3H), 1.05 (t, *J* = 7.5 Hz, 3H), 0.97 (t, *J* = 7.4 Hz, 3H).
^{13}C NMR (101 MHz, $CDCl_3$) δ 162.76, 133.36, 129.02, 113.82, 60.37, 55.47, 47.78, 23.58, 22.53, 12.55, 11.04, 9.45.

HRMS (ESI) exact mass calculated for $[M+H]^+$ ($C_{14}H_{22}NO_3S^+$) *m/z* 284.1315, found *m/z* 284.1309.

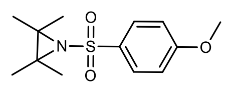

4l

2,2-diethyl-1-((4-methoxyphenyl)sulfonyl)-3-methylaziridine (4l):

The reaction was carried out following the procedure for tretrasubstrated olefin azaridiantaion using 4-methoxybenzenesulfonamide and 4 equivalent tetramethylethene. Then 167.1 mg faint yellow oily liquid was obtained in 31% isolated yield.

^{1}H NMR (400 MHz, $CDCl_3$) δ 7.89 – 7.80 (m, 2H), 6.99 – 6.91 (m, 2H), 3.85 (s, 3H), 1.44 (s, 12H).

^{13}C NMR (101 MHz, $CDCl_3$) δ 162.61, 134.53, 128.86, 113.79, 55.49, 52.81, 20.10.

HRMS (ESI) exact mass calculated for $[M+H]^+$ ($C_{13}H_{20}NO_3S^+$) *m/z* 270.1158, found *m/z* 270.1153.

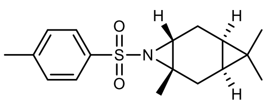

5a

(1*S*,3*S*,5*R*,7*R*)-3,8,8-trimethyl-4-tosyl-4-azatricyclo[5.1.0.0^{3,5}]octane (5a):

The reaction was carried out following the procedure A using toluene-4-sulfonamide and 2 equivalent (+)-car-3-ene. Then 330.1 mg colorless oily liquid was obtained in 54% isolated yield. dr > 20:1.

^{1}H NMR (400 MHz, $CDCl_3$) δ 7.82 (d, *J* = 8.3 Hz, 2H), 7.29 (d, *J* = 8.1 Hz, 2H), 2.92 (t, *J* = 2.3 Hz, 1H), 2.43 (s, 3H), 2.27 (dd, *J* = 15.9, 9.4 Hz, 1H), 2.06 – 1.97 (m, 1H), 1.64 (s, 3H), 1.44 (dt, *J* = 16.0, 3.0 Hz, 1H), 1.25 (dd, *J* = 15.9, 3.2 Hz, 1H), 0.97 (s, 3H), 0.68 (s, 3H), 0.52 (td, *J* = 9.4, 3.2 Hz, 1H), 0.33 (td, *J* = 9.4, 3.2 Hz, 1H).

^{13}C NMR (101 MHz, $CDCl_3$) δ 143.25, 139.01, 129.38, 126.87, 48.60, 45.91, 27.58, 25.19, 21.55, 19.46, 17.61, 16.34, 16.09, 15.03, 13.61.

HRMS (ESI) exact mass calculated for $[M+H]^+$ ($C_{17}H_{24}NO_2S^+$) *m/z* 306.1522, found *m/z* 306.1519.

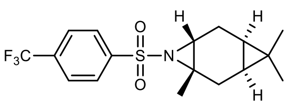

5b

(1*S*,3*S*,5*R*,7*R*)-3,8,8-trimethyl-4-((4-(trifluoromethyl)phenyl)sulfonyl)-4-azatricyclo [5.1.0.0^{3,5}] octane (5b):

The reaction was carried out following the procedure A using 4-(trifluoromethyl) benzenesulfonamide and 2 equivalent (+)-car-3-ene. Then 301.1 mg white solid was obtained in 42% isolated yield. dr > 20:1.

^{1}H NMR (400 MHz, $CDCl_3$) δ 8.07 (d, *J* = 8.2 Hz, 2H), 7.78 (d, *J* = 8.3 Hz, 2H), 3.00 (t, *J* = 2.2 Hz, 1H), 2.32 (dd, *J* = 16.0, 9.4 Hz, 1H), 2.09 – 1.93 (m, 1H), 1.68 (s, 3H), 1.47 (dt, *J* = 16.1, 3.0 Hz, 1H), 1.35 – 1.27 (m, 1H), 0.98 (s, 3H), 0.70 (s, 3H), 0.53 (td, *J* = 9.3, 3.2 Hz, 1H), 0.32 (td, *J* = 9.4, 3.3 Hz, 1H).
^{13}C NMR (101 MHz, $CDCl_3$) δ 145.30, 145.29, 134.20 (q, J = 33.0 Hz), 127.35, 126.05, 125.97, 49.79, 46.90, 27.52, 25.09, 19.86, 17.62, 16.44, 15.98, 15.01, 13.43, 0.99.
^{19}F NMR (377 MHz, $CDCl_3$) δ -63.05.
HRMS (ESI) exact mass calculated for $[M+H]^+$ ($C_{17}H_{21}F_3NO_2S^+$) *m/z* 360.1240, found *m/z* 360.1244.

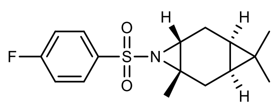

5c

(1*S*,3*S*,5*R*,7*R*)-4-((4-fluorophenyl)sulfonyl)-3,8,8-trimethyl-4-azatricyclo[5.1.0.0^{3,5}]octane (5c):
The reaction was carried out following the procedure A using 4-fluorobenzenesulfonamide and 2 equivalent (+)-car-3-ene. Then 421.1 mg colorless oily liquid was obtained in 68% isolated yield. dr > 20:1.
^{1}H NMR (400 MHz, $CDCl_3$) δ 8.01 – 7.87 (m, 2H), 7.24 – 7.12 (m, 2H), 2.94 (t, *J* = 2.3 Hz, 1H), 2.29 (dd, *J* = 16.0, 9.4 Hz, 1H), 2.04 – 1.96 (m, 1H), 1.65 (s, 3H), 1.45 (dt, *J* = 16.1, 3.0 Hz, 1H), 1.27 (dd, *J* = 15.9, 3.2 Hz, 1H), 0.98 (s, 3H), 0.69 (s, 3H), 0.52 (td, *J* = 9.4, 3.2 Hz, 1H), 0.32 (td, *J* = 9.4, 3.2 Hz, 1H).
^{13}C NMR (101 MHz, $CDCl_3$) δ 165.00 (d, J = 254.3 Hz), 137.97 (d, J = 3.3 Hz), 129.60 (d, J = 9.4 Hz), 115.98 (d, J = 22.5 Hz), 49.13, 46.31, 27.55, 25.14, 19.57, 17.60, 16.38, 16.01, 15.01, 13.50.
^{19}F NMR (377 MHz, $CDCl_3$) δ -105.56.
HRMS (ESI) exact mass calculated for $[M+H]^+$ ($C_{16}H_{21}FNO_2S^+$) *m/z* 310.1272, found *m/z* 310.1270.

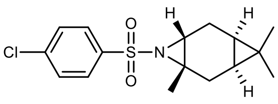

5d

(1*S*,3*S*,5*R*,7*R*)-4-((4-chlorophenyl)sulfonyl)-3,8,8-trimethyl-4-azatricyclo[5.1.0.0^{3,5}]octane (5d):
The reaction was carried out following the procedure A using 4-chlorobenzenesulfonamide and 2 equivalent (+)-car-3-ene. Then 453.5 mg colorless oily liquid was obtained in 71% isolated yield. dr > 20:1.
^{1}H NMR (400 MHz, $CDCl_3$) δ 7.88 (d, *J* = 8.6 Hz, 2H), 7.48 (d, *J* = 8.6 Hz, 2H), 2.95 (t, *J* = 2.0 Hz, 1H), 2.29 (dd, *J* = 16.0, 9.4 Hz, 1H), 2.08 – 1.95 (m, 1H), 1.65 (s, 3H), 1.45 (dt, *J* = 16.2, 3.0 Hz, 1H), 1.27 (dd, *J* = 16.0, 3.1 Hz, 2H), 0.98 (s, 3H), 0.69 (s, 3H), 0.52 (td, *J* = 9.3, 3.2 Hz, 1H), 0.32 (td, *J* = 9.4, 3.2 Hz, 1H).
^{13}C NMR (101 MHz, $CDCl_3$) δ 140.37, 139.00, 129.08, 128.34, 49.30, 46.45, 27.53, 25.11, 19.64, 17.59, 16.39, 15.99, 15.01, 13.47.

HRMS (ESI) exact mass calculated for $[M+H]^+$ ($C_{16}H_{21}ClNO_2S^+$) *m/z* 326.0976, found *m/z* 326.0977.

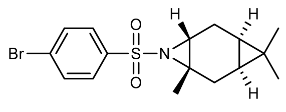

5e

(1*S*,3*S*,5*R*,7*R*)-4-((4-bromophenyl)sulfonyl)-3,8,8-trimethyl-4-azatricyclo[5.1.0.0^{3,5}]octane (5e):

The reaction was carried out following the procedure A using 4-bromobenzenesulfonamide and 2 equivalent (+)-car-3-ene. Then 414.9 mg colorless oily liquid was obtained in 56% isolated yield. dr > 20:1.

^{1}H NMR (400 MHz, $CDCl_3$) δ 7.84 – 7.77 (m, 2H), 7.70 – 7.58 (m, 2H), 2.94 (t, *J* = 2.3 Hz, 1H), 2.29 (dd, *J* = 16.0, 9.4 Hz, 1H), 2.08 – 1.94 (m, 1H), 1.65 (s, 3H), 1.45 (dt, *J* = 16.1, 3.0 Hz, 1H), 1.27 (dd, *J* = 15.9, 3.1 Hz, 1H), 0.97 (s, 3H), 0.69 (s, 3H), 0.51 (td, *J* = 9.4, 3.2 Hz, 1H), 0.32 (td, *J* = 9.4, 3.3 Hz, 1H).

^{13}C NMR (101 MHz, $CDCl_3$) δ 140.89, 132.05, 128.43, 127.50, 49.31, 46.47, 27.53, 25.10, 19.64, 17.58, 16.38, 15.98, 15.00, 13.46.

HRMS (ESI) exact mass calculated for $[M+H]^+$ ($C_{16}H_{21}BrNO_2S^+$) *m/z* 370.0471, found *m/z* 370.0473.

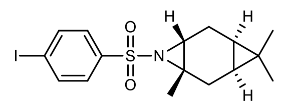

5f

(1*S*,3*S*,5*R*,7*R*)-4-((4-iodophenyl)sulfonyl)-3,8,8-trimethyl-4-azatricyclo[5.1.0.0^{3,5}]octane (5f):

The reaction was carried out following the procedure A using 4-iodobenzenesulfonamide and 2 equivalent (+)-car-3-ene. Then 275.1 mg colorless oily liquid was obtained in 33% isolated yield. dr > 20:1.

^{1}H NMR (400 MHz, $CDCl_3$) δ 7.93 – 7.82 (m, 2H), 7.74 – 7.62 (m, 2H), 2.94 (t, *J* = 2.3 Hz, 1H), 2.29 (dd, *J* = 16.0, 9.4 Hz, 1H), 2.07 – 1.97 (m, 1H), 1.65 (s, 3H), 1.45 (dt, *J* = 16.2, 3.0 Hz, 1H), 1.27 (dd, *J* = 15.4, 2.4 Hz, 1H), 0.98 (s, 3H), 0.69 (s, 3H), 0.52 (td, *J* = 9.4, 3.2 Hz, 1H), 0.32 (td, *J* = 9.4, 3.3 Hz, 1H).

^{13}C NMR (101 MHz, $CDCl_3$) δ 141.59, 138.05, 128.32, 100.01, 49.35, 46.50, 27.56, 25.13, 19.69, 17.62, 16.41, 16.02, 15.03, 13.50.

HRMS (ESI) exact mass calculated for $[M+H]^+$ ($C_{16}H_{21}INO_2S^+$) *m/z* 418.0332, found *m/z* 418.0337.

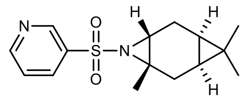

5g

(1*S*,3*S*,5*R*,7*R*)-3,8,8-trimethyl-4-(pyridin-3-ylsulfonyl)-4-azatricyclo[5.1.0.0^{3,5}]octane (5g):

The reaction was carried out following the procedure A using pyridine-3-sulfonamide and 2 equivalent (+)-car-3-ene. Then 181.2 mg faint yellow oily liquid was obtained in 31% isolated yield. dr > 20:1.
^{1}H NMR (400 MHz, $CDCl_3$) δ 9.15 (d, *J* = 1.8 Hz, 1H), 8.79 (dd, *J* = 4.9, 1.6 Hz, 1H), 8.21 (dt, *J* = 8.0, 1.8 Hz, 1H), 7.55 – 7.40 (m, 1H), 3.00 (t, *J* = 2.3 Hz, 1H), 2.32 (dd, *J* = 16.1, 9.4 Hz, 1H), 2.06 – 1.98 (m, 1H), 1.68 (s, 3H), 1.46 (dt, *J* = 16.2, 3.0 Hz, 1H), 1.28 (dd, *J* = 16.0, 3.3 Hz, 1H), 0.97 (s, 3H), 0.69 (s, 3H), 0.52 (td, *J* = 9.3, 3.3 Hz, 1H), 0.32 (td, *J* = 9.4, 3.3 Hz, 1H).
^{13}C NMR (101 MHz, $CDCl_3$) δ 153.05, 147.88, 138.45, 134.42, 123.46, 49.97, 46.90, 27.51, 25.09, 19.87, 17.61, 16.45, 15.96, 15.01, 13.41.
HRMS (ESI) exact mass calculated for $[M+H]^+$ ($C_{15}H_{21}N_2O_2S^+$) *m/z* 293.1318, found *m/z* 293.1310.

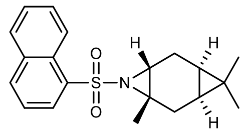

5h

(1*S*,3*S*,5*R*,7*R*)-3,8,8-trimethyl-4-(naphthalen-1-ylsulfonyl)-4-azatricyclo[5.1.0.0^{3,5}]octane (5h):
The reaction was carried out following the procedure A using naphthalene-2-sulfonamide and 2 equivalent (+)-car-3-ene. Then 291.2 mg colorless oily liquid was obtained in 45% isolated yield. dr > 20:1.
^{1}H NMR (400 MHz, $CDCl_3$) δ 8.90 (d, *J* = 8.5 Hz, 1H), 8.25 (dd, *J* = 7.3, 1.0 Hz, 1H), 8.06 (d, *J* = 8.2 Hz, 1H), 7.92 (d, *J* = 8.1 Hz, 1H), 7.72 – 7.63 (m, 1H), 7.63 – 7.48 (m, 2H), 3.05 (t, *J* = 2.2 Hz, 1H), 2.26 (dd, *J* = 15.9, 9.5 Hz, 1H), 2.00 – 1.90 (m, *J* = 16.0, 9.5, 1.9 Hz, 1H), 1.69 (s, 3H), 1.39 (dt, *J* = 16.0, 3.0 Hz, 1H), 1.24 (dd, *J* = 12.6, 3.3 Hz, 1H), 0.92 (s, 3H), 0.67 (s, 3H), 0.45 (td, *J* = 9.4, 3.4 Hz, 1H), 0.14 (td, *J* = 9.4, 3.5 Hz, 1H).
^{13}C NMR (101 MHz, $CDCl_3$) δ 136.90, 134.16, 128.49, 127.95, 127.58, 126.66, 125.92, 123.98, 49.17, 46.10, 27.53, 25.21, 19.68, 17.72, 16.34, 16.05, 15.04, 13.67.
HRMS (ESI) exact mass calculated for $[M+H]^+$ ($C_{20}H_{24}NO_2S^+$) *m/z* 324.1522, found *m/z* 324.1523.

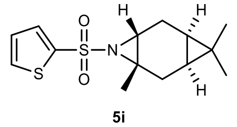

5i

(1*S*,3*S*,5*R*,7*R*)-3,8,8-trimethyl-4-(thiophen-2-ylsulfonyl)-4-azatricyclo[5.1.0.0^{3,5}]octane (5i):
The reaction was carried out following the procedure A using 2-thiophenesulfonamide and 2 equivalent (+)-car-3-ene. Then 434.7 mg colorless oily liquid was obtained in 73% isolated yield. dr > 20:1.
^{1}H NMR (400 MHz, $CDCl_3$) δ 7.64 (dd, *J* = 3.7, 1.3 Hz, 1H), 7.58 (dd, *J* = 5.0, 1.3 Hz, 1H), 7.05 (dd, *J* = 5.0, 3.8 Hz, 1H), 2.94 (t, *J* = 2.3 Hz, 1H), 2.30 (dd, *J* = 16.0, 9.4 Hz, 1H), 2.17 – 2.01 (m, 1H), 1.62 (s, 3H), 1.44 (dt, *J* = 16.1, 3.0 Hz, 1H), 1.25 (dd, *J* = 16.0, 3.2 Hz, 1H), 0.97 (s, 3H), 0.68 (s, 3H), 0.54 (td, *J* = 9.4, 3.3 Hz, 1H), 0.38 (td, *J* = 9.4, 3.3 Hz, 1H).

^{13}C NMR (101 MHz, $CDCl_3$) δ 142.53, 131.97, 131.52, 126.87, 49.56, 46.45, 27.51, 25.08, 19.40, 17.50, 16.34, 15.96, 14.99, 13.51.
HRMS (ESI) exact mass calculated for $[M+H]^+$ ($C_{14}H_{20}NO_2S_2^+$) *m/z* 298.0930, found *m/z* 298.0926.

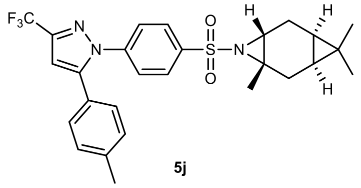

5j

(1*S*,3*S*,5*R*,7*R*)-3,8,8-trimethyl-4-((4-(5-(*p*-tolyl)-3-(trifluoromethyl)-1*H*-pyrazol-1-yl)phenyl)sulfonyl)-4-azatricyclo[5.1.0.0^{3,5}]octane (5j):
The reaction was carried out following the procedure A using celecoxib and 2 equivalent (+)-car-3-ene. Then 464.7 mg faint yellow oily liquid was obtained in 45% isolated yield. dr > 20:1.
^{1}H NMR (400 MHz, $CDCl_3$) δ 8.00 – 7.87 (m, 2H), 7.51 – 7.39 (m, 2H), 7.16 (d, *J* = 8.1 Hz, 2H), 7.10 (d, *J* = 8.1 Hz, 2H), 6.74 (s, 1H), 2.94 (t, *J* = 2.1 Hz, 1H), 2.36 (s, 3H), 2.27 (dd, *J* = 16.0, 9.4 Hz, 1H), 2.02 – 1.93 (m, 1H), 1.65 (s, 3H), 1.44 (dt, *J* = 16.1, 2.9 Hz, 1H), 1.26 (dd, *J* = 16.0, 3.1 Hz, 2H), 0.97 (s, 3H), 0.68 (s, 3H), 0.50 (td, *J* = 9.4, 3.2 Hz, 1H), 0.29 (td, *J* = 9.4, 3.2 Hz, 1H).
^{13}C NMR (101 MHz, $CDCl_3$) δ 145.21, 143.92 (q, J = 38.6 Hz), 142.32, 141.28, 139.66, 129.64, 128.65, 127.92, 125.62, 125.28, 106.11, 49.44, 46.48, 27.51, 25.10, 21.23, 19.59, 17.54, 16.36, 15.97, 14.95, 13.45.
HRMS (ESI) exact mass calculated for $[M+H]^+$ ($C_{27}H_{29}F_3N_3O_2S^+$) *m/z* 516.1927, found *m/z* 516.1922.

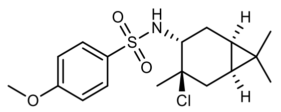

6a

***N*-((1*R*,3*R*,4*R*,6*S*)-4-chloro-4,7,7-trimethylbicyclo[4.1.0]heptan-3-yl)-4-methoxybenzenesulfonamide (6a):**
The reaction was carried out following the procedure C using 0.2 mmol aziridine **3a** and 2 mL concentrate hydrogen chloride in ethyl acetate. Reaction was carried out at room temperature. Then 54.2 mg white solid was obtained in 76% isolated yield. dr > 20:1.
^{1}H NMR (400 MHz, $CDCl_3$) δ 7.85 – 7.77 (m, 2H), 7.02 – 6.89 (m, 2H), 5.01 (d, *J* = 5.0 Hz, 1H), 3.86 (s, 3H), 3.01 – 2.91 (m, 1H), 2.33 (dd, *J* = 14.8, 9.6 Hz, 1H), 2.16 (dd, *J* = 15.1, 7.1 Hz, 1H), 1.84 – 1.71 (m, 1H), 1.69 – 1.61 (m, 1H), 1.52 (s, 3H), 0.94 (s, 3H), 0.90 (s, 3H), 0.75 – 0.61 (m, 2H).
^{13}C NMR (101 MHz, $CDCl_3$) δ 162.82, 131.36, 129.44, 114.00, 72.05, 59.24, 55.54, 37.90, 28.28, 27.78, 22.80, 19.72, 19.62, 17.93, 15.41.
HRMS (ESI) exact mass calculated for $[M+H]^+$ ($C_{17}H_{25}ClNO_3S^+$) *m/z* 358.1238, found *m/z* 358.1233.

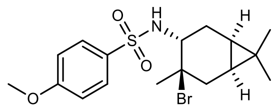

6b

***N*-((1*R*,3*R*,4*R*,6*S*)-4-bromo-4,7,7-trimethylbicyclo[4.1.0]heptan-3-yl)-4-methoxybenzenesulfonamide (6b):**

The reaction was carried out following the procedure C using 0.2 mmol aziridine **3a**, 3 equivalent $CuBr_2$ and 2 mL ethyl acetate. Reaction was carried out at 40 ^oC. Then 55.7 mg white solid was obtained in 69% isolated yield. dr > 20:1.

^{1}H NMR (400 MHz, $CDCl_3$) δ 7.91 – 7.79 (m, 2H), 7.04 – 6.93 (m, 2H), 5.24 (d, *J* = 5.7 Hz, 1H), 3.89 (s, 3H), 3.18 – 3.06 (m, 1H), 2.56 (dd, *J* = 14.8, 9.5 Hz, 1H), 2.14 (dd, *J* = 15.1, 7.2 Hz, 1H), 1.94 (dd, *J* = 14.8, 4.4 Hz, 1H), 1.90 – 1.80 (m, 1H), 1.74 (s, 3H), 0.95 (d, *J* = 8.1 Hz, 6H), 0.78 – 0.65 (m, 2H).

^{13}C NMR (101 MHz, $CDCl_3$) δ 162.81, 131.29, 129.49, 113.97, 70.05, 59.74, 55.53, 39.49, 28.19, 27.65, 24.21, 20.18, 19.74, 17.91, 15.42.

HRMS (ESI) exact mass calculated for $[M+H]^+$ ($C_{17}H_{25}BrNO_3S^+$) *m/z* 402.0733, found *m/z* 402.0730.

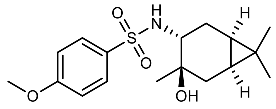

6c

***N*-((1*R*,3*R*,4*R*,6*S*)-4-hydroxy-4,7,7-trimethylbicyclo[4.1.0]heptan-3-yl)-4-methoxybenzenesulfonamide (6c):**

The reaction was carried out following the procedure C using 0.2 mmol aziridine **3a** and 2 equivalent $TBAHSO_4$, 5 mol% $Cu(OTf)_2$ and 2 mL ethanol. Reaction was carried out at room temperature. 5 mL satured $NaHCO_3$ aqueous was added to reaction mixture and stirred for 12 h. Then extracted with ethyl acetate (3×10 mL), and combined organic layers were dried over anhydrous sodium sulphate and filtered. Then 45.3 mg colorless oily liquid was obtained in 65% isolated yield. dr > 20:1.

^{1}H NMR (400 MHz, $CDCl_3$) δ 7.86 – 7.76 (m, 2H), 7.03 – 6.91 (m, 2H), 4.83 (d, *J* = 7.9 Hz, 1H), 3.87 (s, 3H), 2.78 (dt, *J* = 11.2, 7.5 Hz, 1H), 2.50 (s, 1H), 2.01 (dd, *J* = 14.5, 10.1 Hz, 1H), 1.65 (dd, *J* = 14.6, 7.2 Hz, 1H), 1.52 – 1.40 (m, 1H), 1.23 (dd, *J* = 14.5, 4.7 Hz, 1H), 1.15 (s, 3H), 0.90 (s, 3H), 0.81 (s, 3H), 0.72 – 0.62 (m, 1H), 0.51 (t, *J* = 8.5 Hz, 1H).

^{13}C NMR (101 MHz, $CDCl_3$) δ 162.96, 131.57, 129.24, 114.26, 70.50, 58.92, 55.60, 33.88, 28.51, 27.31, 20.51, 19.78, 19.40, 17.55, 15.20.

HRMS (ESI) exact mass calculated for $[M+H-H_2O]^+$ ($C_{17}H_{24}NO_3S^+$) *m/z* 322.1471, found *m/z* 322.1460.

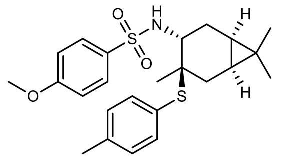

6d

4-methoxy-*N*-((1*R*,3*R*,4*R*,6*S*)-4,7,7-trimethyl-4-(*p*-tolylthio)bicyclo[4.1.0]heptan-3-yl) benzenesulfonamide (6d):
The reaction was carried out following the procedure C using 0.2 mmol aziridine **3a**, 20 mol% $InCl_3$, 1.5 equivalent *p*-toluenethiol and 2 mL DCM. Reaction was carried out at room temperature. Then 52.0 mg faint yellow oily liquid was obtained in 58% isolated yield. dr > 20:1.
^{1}H NMR (400 MHz, $CDCl_3$) δ 7.90 – 7.85 (m, 2H), 7.18 (d, *J* = 8.1 Hz, 2H), 7.06 (d, *J* = 7.9 Hz, 2H), 7.04 – 6.98 (m, 2H), 5.50 (d, *J* = 3.3 Hz, 1H), 3.91 (s, 3H), 2.88 – 2.81 (m, 1H), 2.35 (s, 3H), 2.15 (dd, *J* = 15.0, 6.9 Hz, 1H), 1.93 (dd, *J* = 14.7, 9.6 Hz, 1H), 1.82 – 1.73 (m, 1H), 1.32 – 1.26 (m, 1H), 1.25 (s, 3H), 0.91 (s, 3H), 0.71 (s, 3H), 0.66 – 0.54 (m, 2H).
^{13}C NMR (101 MHz, $CDCl_3$) δ 162.75, 139.22, 137.31, 132.22, 129.35, 125.94, 114.06, 55.53, 55.50, 51.71, 33.56, 28.39, 25.95, 21.17, 19.64, 18.80, 18.03, 17.44, 15.22.
HRMS (ESI) exact mass calculated for $[M+H]^+$ ($C_{24}H_{32}NO_3S_2^+$) *m/z* 446.1818, found *m/z* 446.1805.

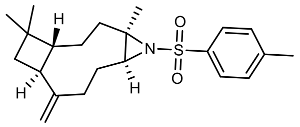

6e

(1*R*,4*R*,6*S*,10*S*)-4,12,12-trimethyl-9-methylene-5-tosyl-5-azatricyclo[8.2.0.0^{4,6}]dodecane (6e):
The reaction was carried out following the procedure A using toluene-4-sulfonamide and 4 equivalent (-)-*β*-caryophyllene. Then 372.2 mg colorless oily liquid was obtained in 50% isolated yield. dr > 20:1.
^{1}H NMR (400 MHz, $CDCl_3$) δ 7.78 (d, *J* = 8.2 Hz, 1H), 7.29 (d, *J* = 8.1 Hz, 1H), 4.87 (d, *J* = 61.3 Hz, 1H), 3.10 (dd, *J* = 11.1, 3.9 Hz, 1H), 2.62 (q, *J* = 9.9 Hz, 1H), 2.42 (s, 2H), 2.34 – 2,24 (m, 1H), 2.22 – 1.92 (m, 4H), 1.85 (t, *J* = 9.9 Hz, 1H), 1.77 – 1.62 (m, 2H), 1.53 – 1.41 (m, 1H), 1.38 – 1.28 (m, 1H), 1.20 (s, 1H), 1.00 (s, 2H), 0.99 (s, 1H).
^{13}C NMR (101 MHz, $CDCl_3$) δ 151.26, 143.38, 138.78, 129.41, 126.83, 112.90, 54.39, 52.30, 49.46, 48.87, 39.50, 34.55, 34.41, 30.09, 29.85, 29.60, 27.36, 21.56, 21.44, 18.82.
HRMS (ESI) exact mass calculated for $[M+H]^+$ ($C_{22}H_{32}NO_2S^+$) *m/z* 374.2148, found m/z 374.2155.

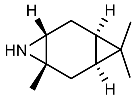

7a

(1*S*,3*S*,5*R*,7*R*)-3,8,8-trimethyl-4-azatricyclo[5.1.0.0^{3,5}]octane (7a):

The reaction was carried out following the procedure for electrochemical detosylation of aziridine **3a** . Then 29.8 mg yellow oily liquid was obtained in 66% isolated yield. dr > 20:1.
^{1}**H NMR** (400 MHz, $CDCl_3$) δ 2.21 – 2.11 (m, 1H), 1.99 (d, *J* = 9.5 Hz, 1H), 1.73 (t, *J* = 2.2 Hz, 1H), 1.54 (dt, *J* = 15.7, 3.1 Hz, 1H), 1.34 (dd, *J* = 15.5, 3.5 Hz, 1H), 1.13 (s, 2H), 1.01 (s, 2H), 0.74 (s, 2H), 0.45 (td, *J* = 9.2, 3.5 Hz, 1H), 0.33 (td, *J* = 9.2, 3.5 Hz, 1H).
^{13}**C NMR** (101 MHz, $CDCl_3$) δ 36.61, 32.81, 27.81, 24.66, 23.01, 17.97, 16.38, 15.43, 14.72, 12.81.
HRMS (ESI) exact mass calculated for $[M+H]^+$ ($C_{10}H_{18}N^+$) *m/z* 152.1434, found *m/z* 152.1435.

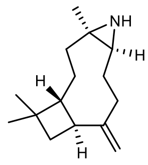

7b

(1*R*,4*R*,6*S*,10*S*)-4,12,12-trimethyl-9-methylene-5-5-azatricyclo[8.2.0.0^{4,6}]dodecane (7b):
The reaction was carried out following the procedure for electrochemical detosylation of aziridine **6e** . Then 32.5 mg yellow oily liquid was obtained in 49% isolated yield. dr > 20:1.
^{1}**H NMR** (400 MHz, $CDCl_3$) δ 4.85 (d, *J* = 51.6 Hz, 1H), 2.55 (q, *J* = 9.3 Hz, 1H), 2.34 – 2.26 (m, 1H), 2.19 – 2.07 (m, 2H), 2.01 – 1.93 (m, 1H), 1.91 – 1.83 (m, 1H), 1.78 (t, *J* = 8.5 Hz, 1H), 1.70 (t, *J* = 9.4 Hz, 1H), 1.61 (dd, *J* = 9.4, 4.3 Hz, 1H), 1.56 – 1.49 (m, 1H), 1.47 – 1.36 (m, 1H), 1.05 (s, 1H), 0.95 (s, 1H), 0.92 (s, 1H).
^{13}**C NMR** (101 MHz, $CDCl_3$) δ 152.44, 111.61, 51.70, 48.62, 42.46, 41.98, 39.18, 37.19, 33.67, 31.93, 31.12, 29.79, 27.55, 21.60, 17.70.
HRMS (ESI) exact mass calculated for $[M+H]^+$ ($C_{15}H_{26}N^+$) *m/z* 220.2060, found *m/z* 220.2054.

NMR Spectra

^{1}H NMR (400 MHz, $CDCl_3$):

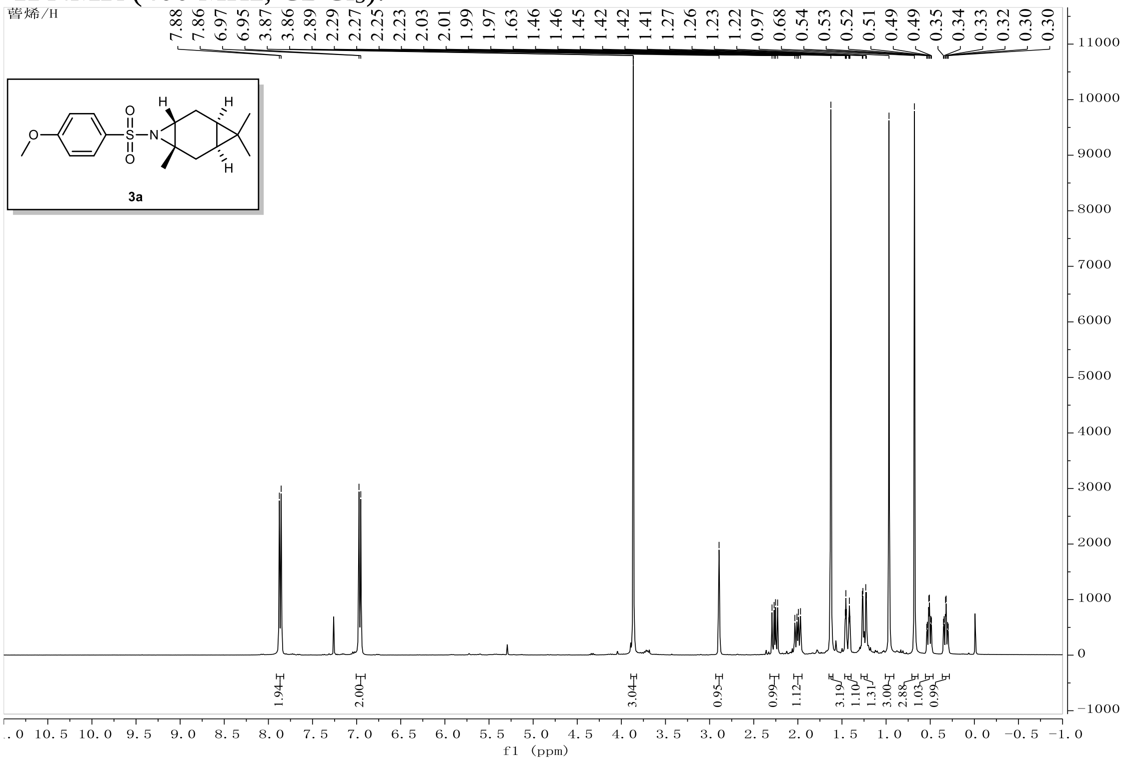

^{13}C NMR (101 MHz, $CDCl_3$):

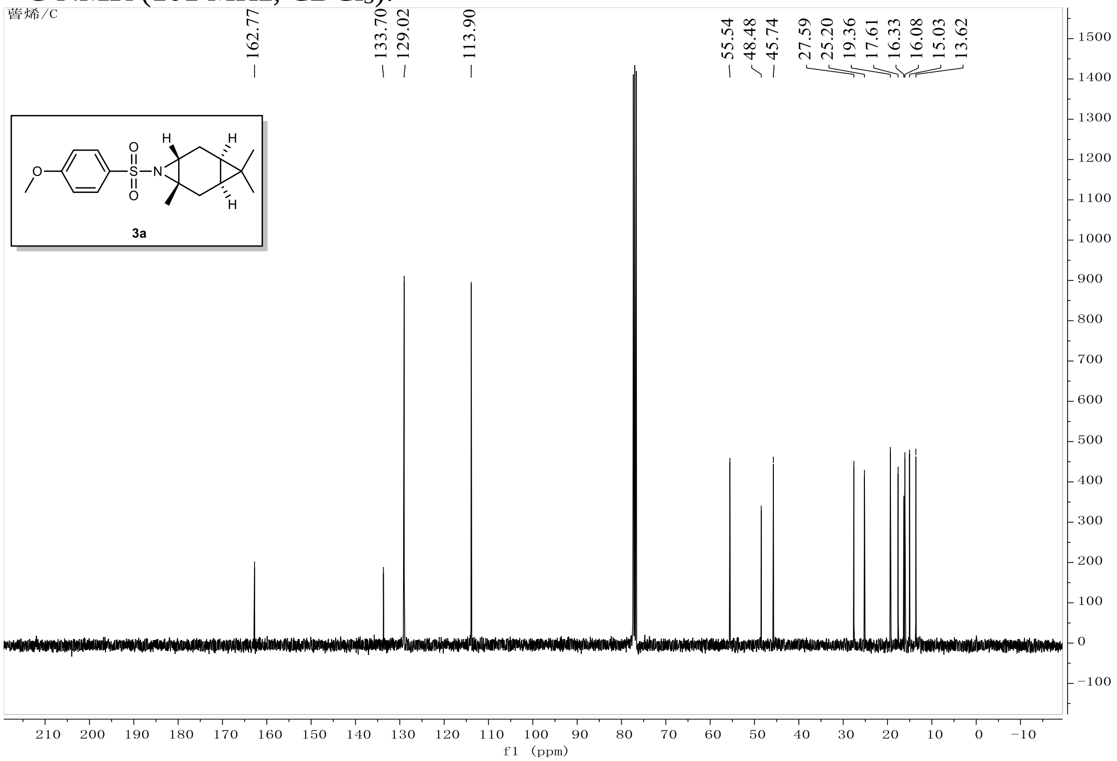

^{1}H NMR (400 MHz, $CDCl_3$):

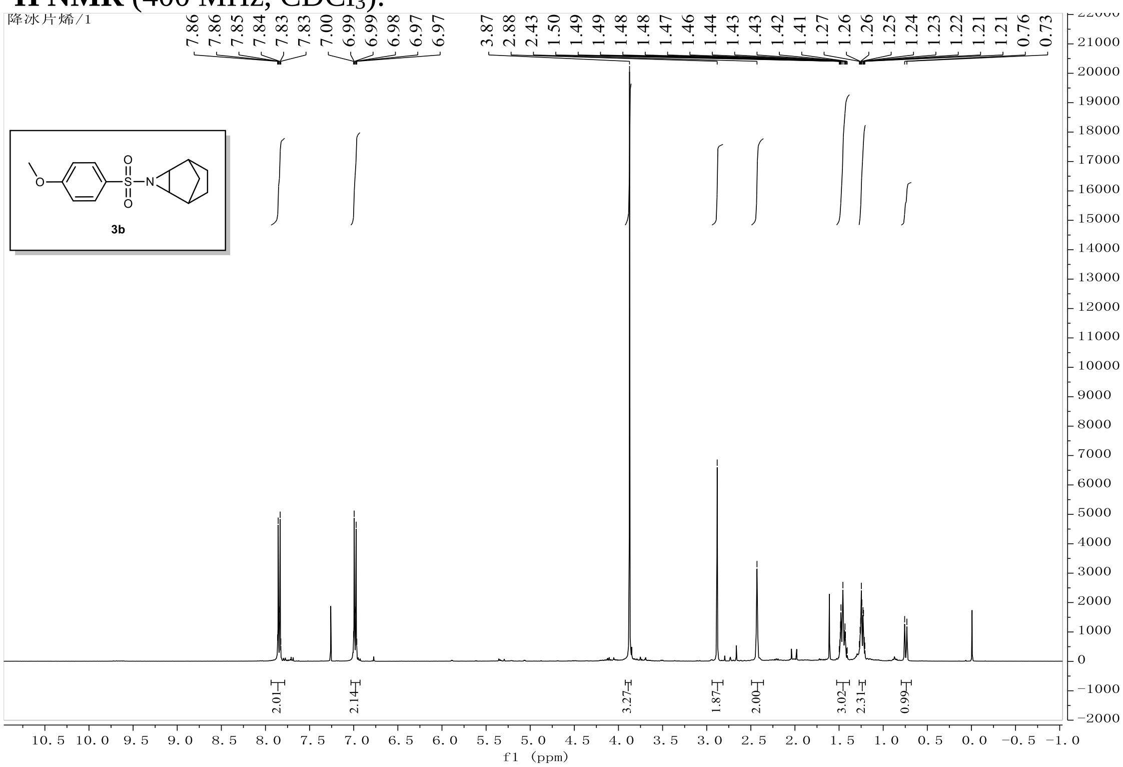

^{13}C NMR (101 MHz, $CDCl_3$):

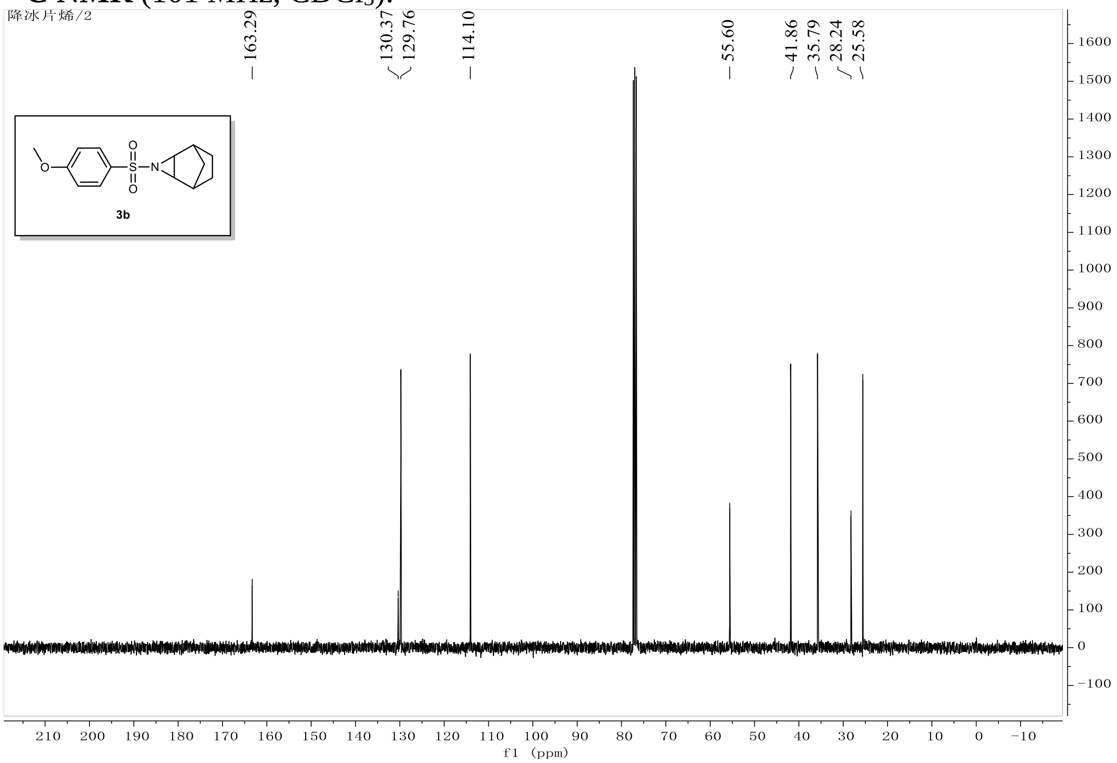

¹H NMR (400 MHz, $CDCl_3$):

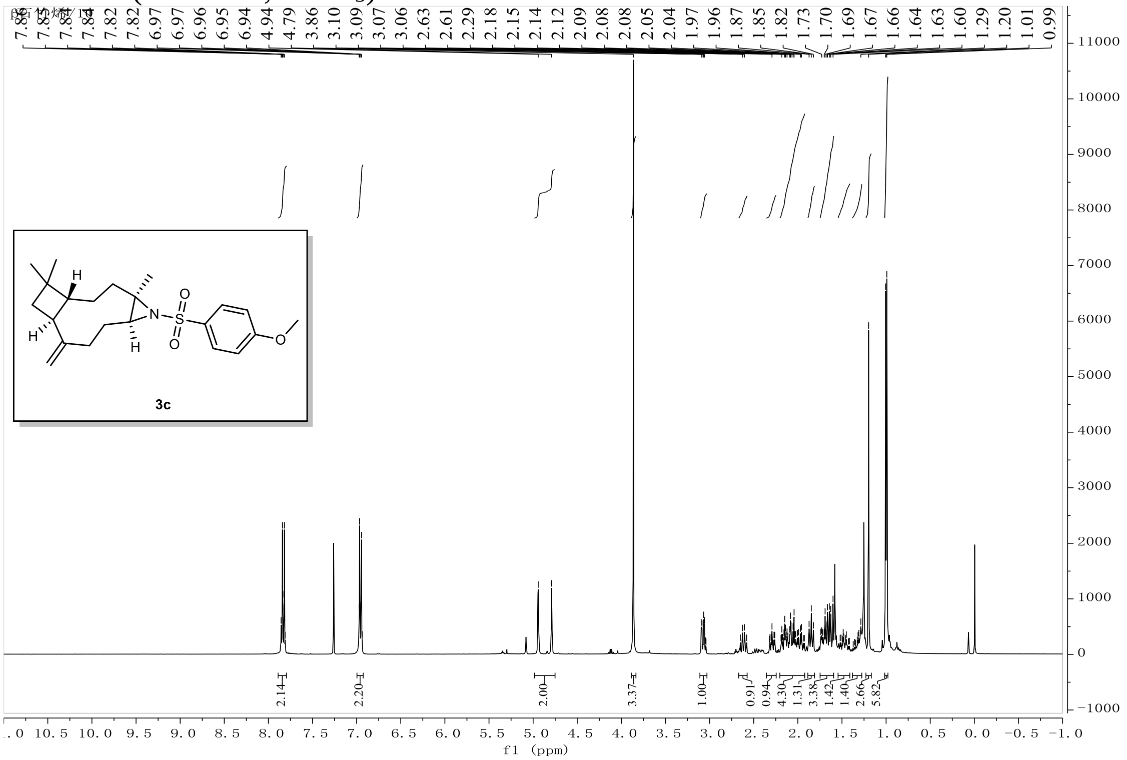

¹³C NMR (101 MHz, $CDCl_3$):

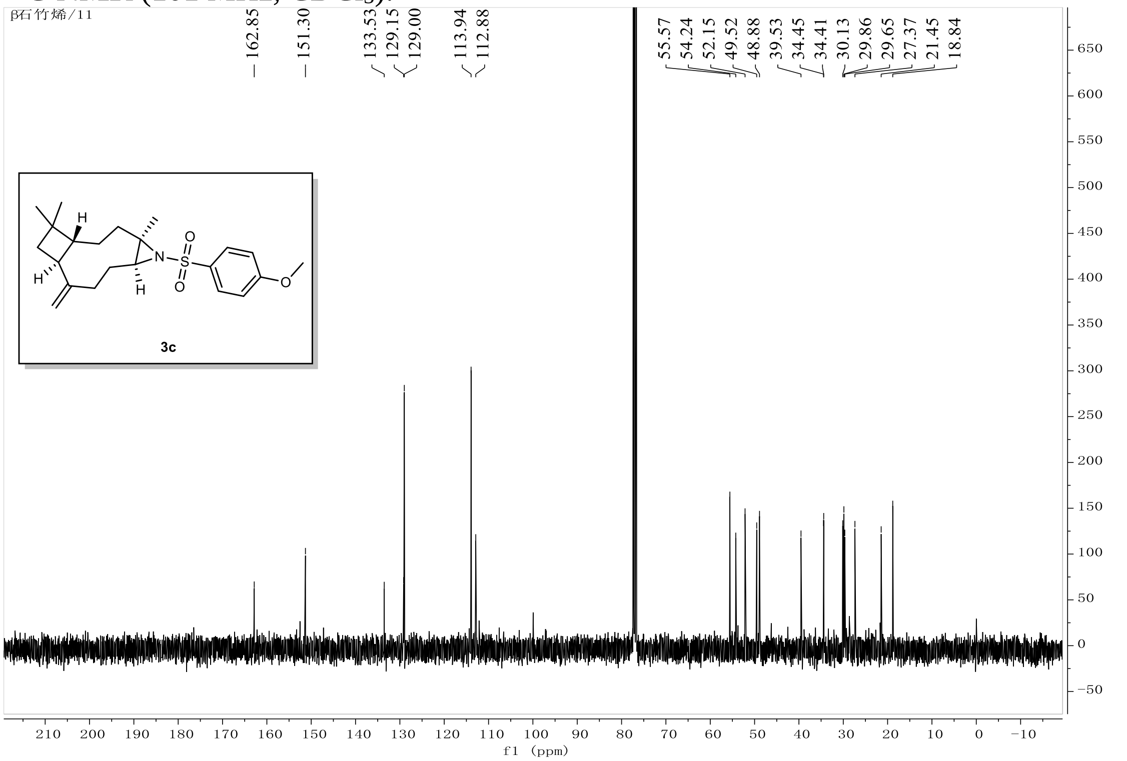

^{1}H NMR (400 MHz, $CDCl_3$):

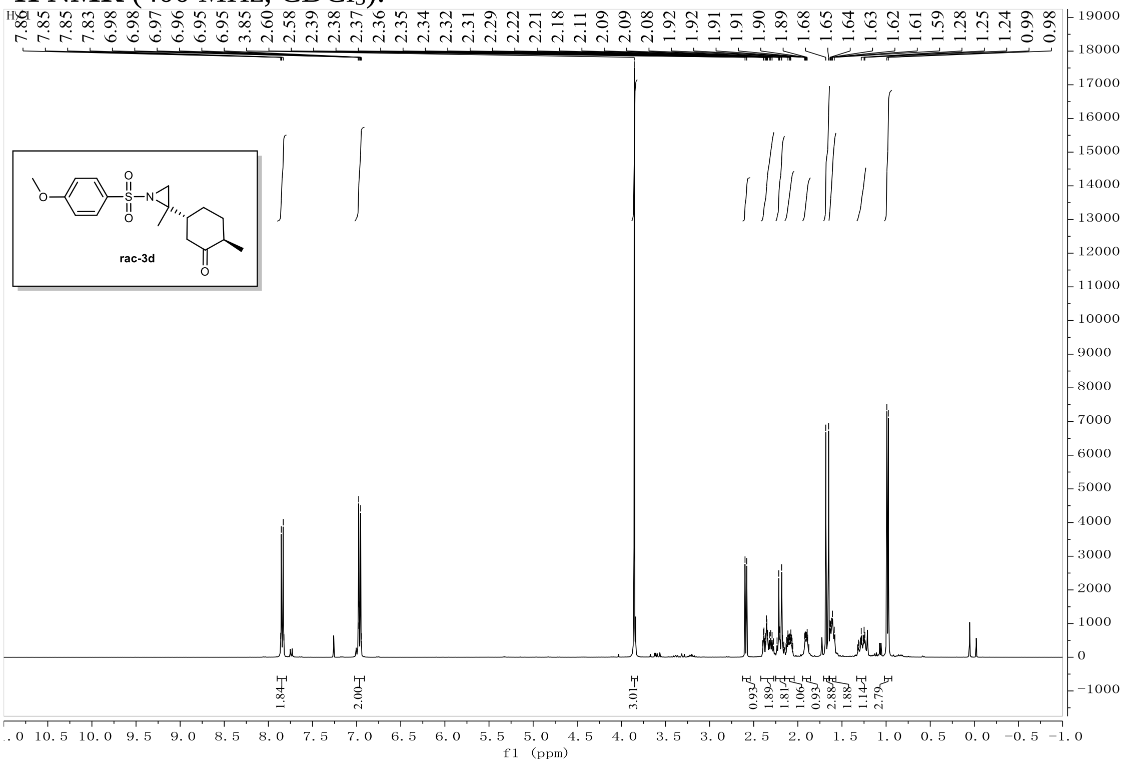

^{13}C NMR (101 MHz, $CDCl_3$):

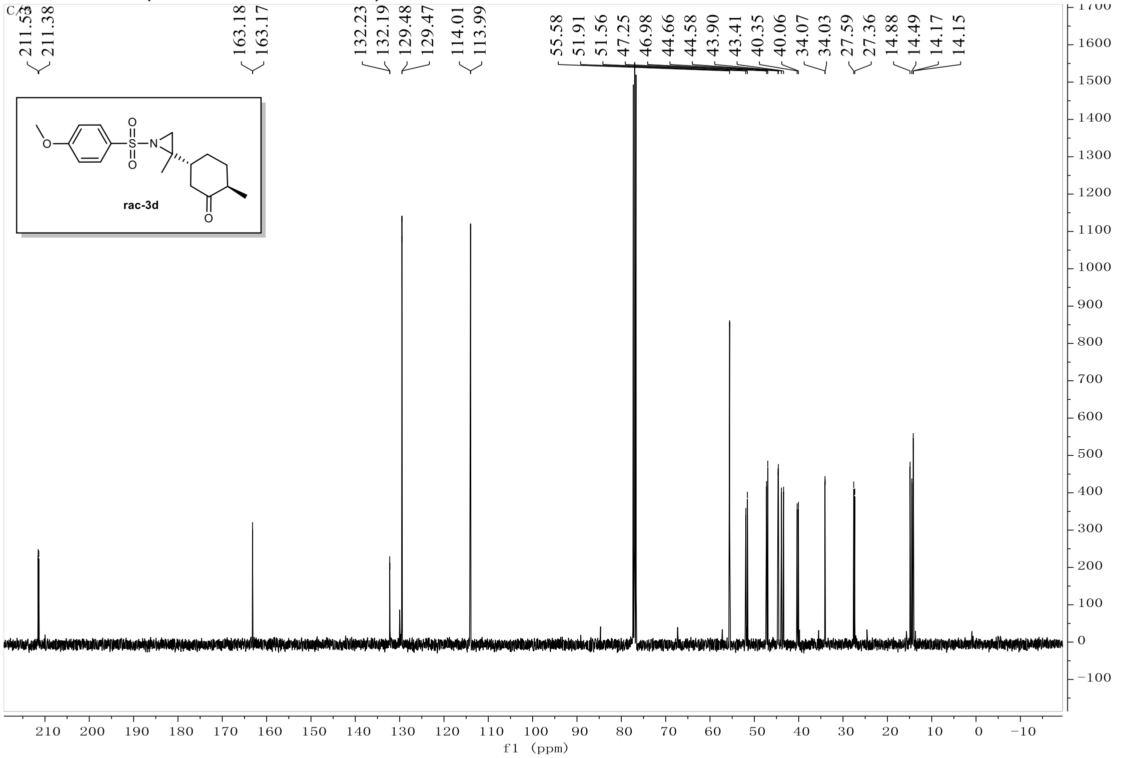

^{1}H NMR (400 MHz, $CDCl_3$):

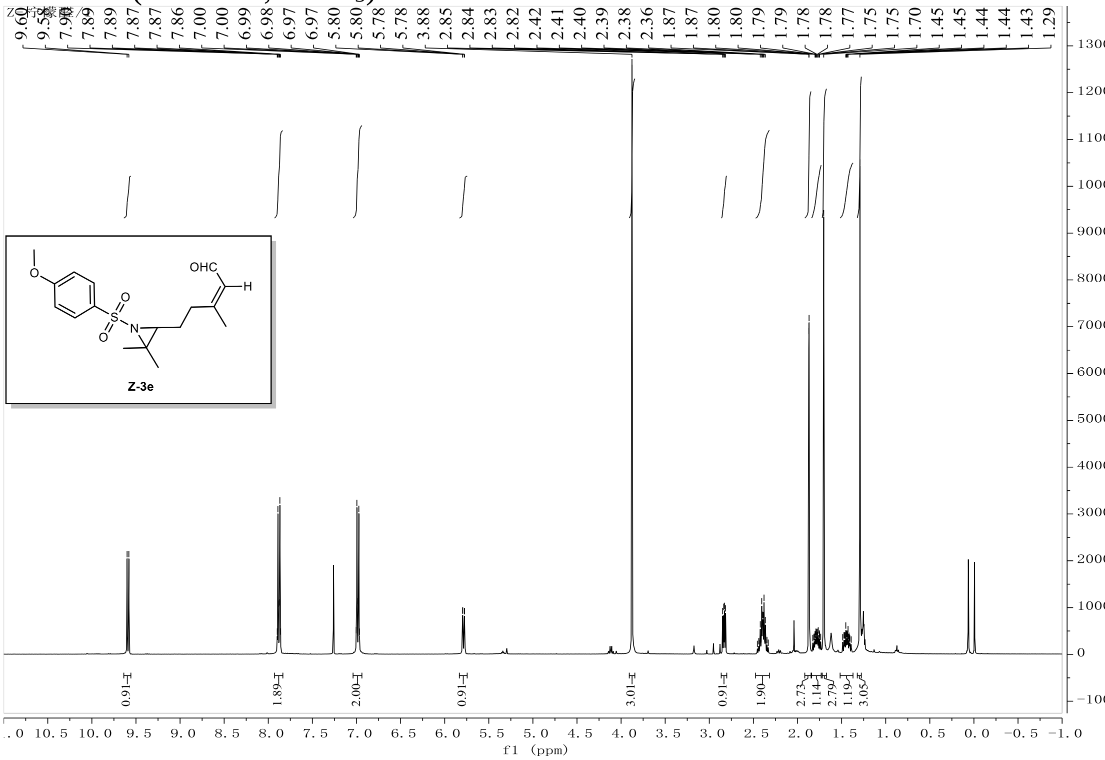

^{13}C NMR (101 MHz, $CDCl_3$):

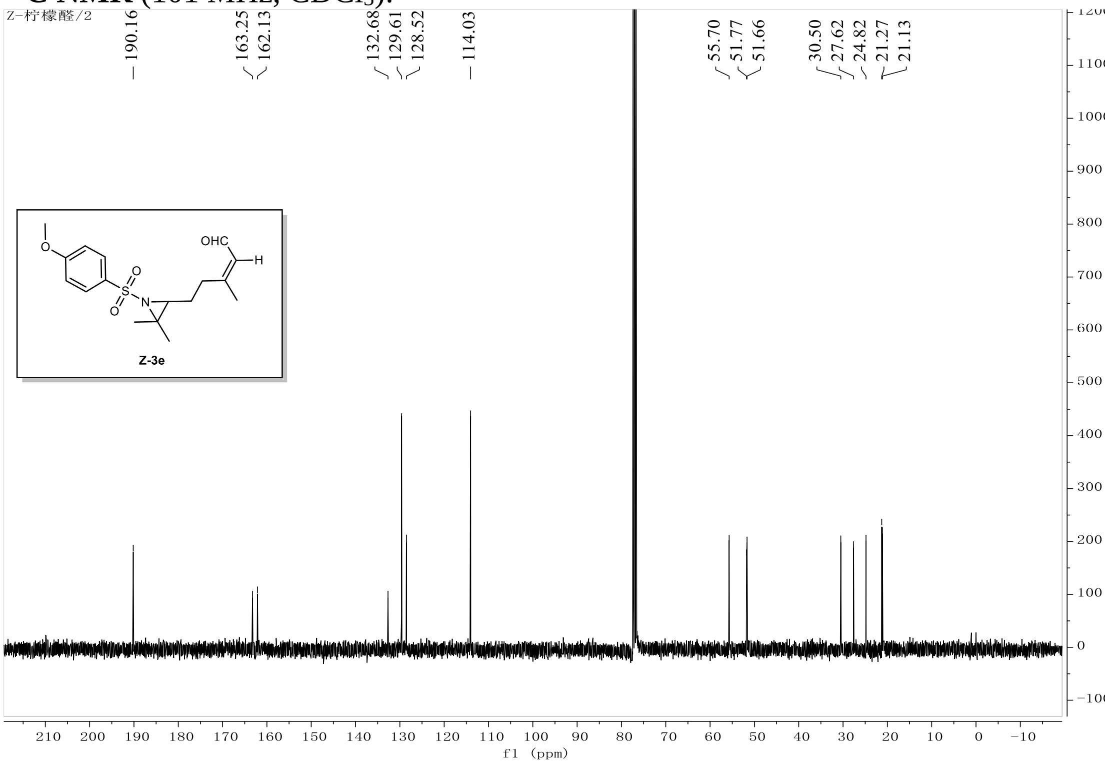

^{1}H NMR (400 MHz, $CDCl_3$):

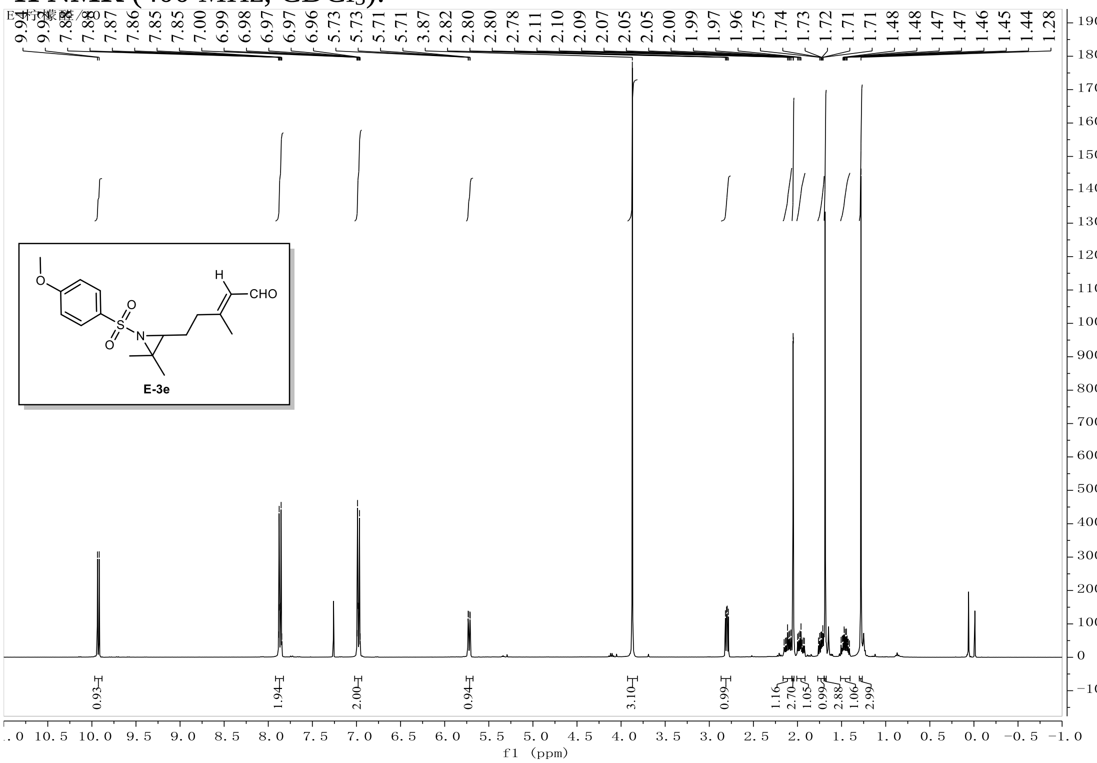

^{13}C NMR (101 MHz, $CDCl_3$):

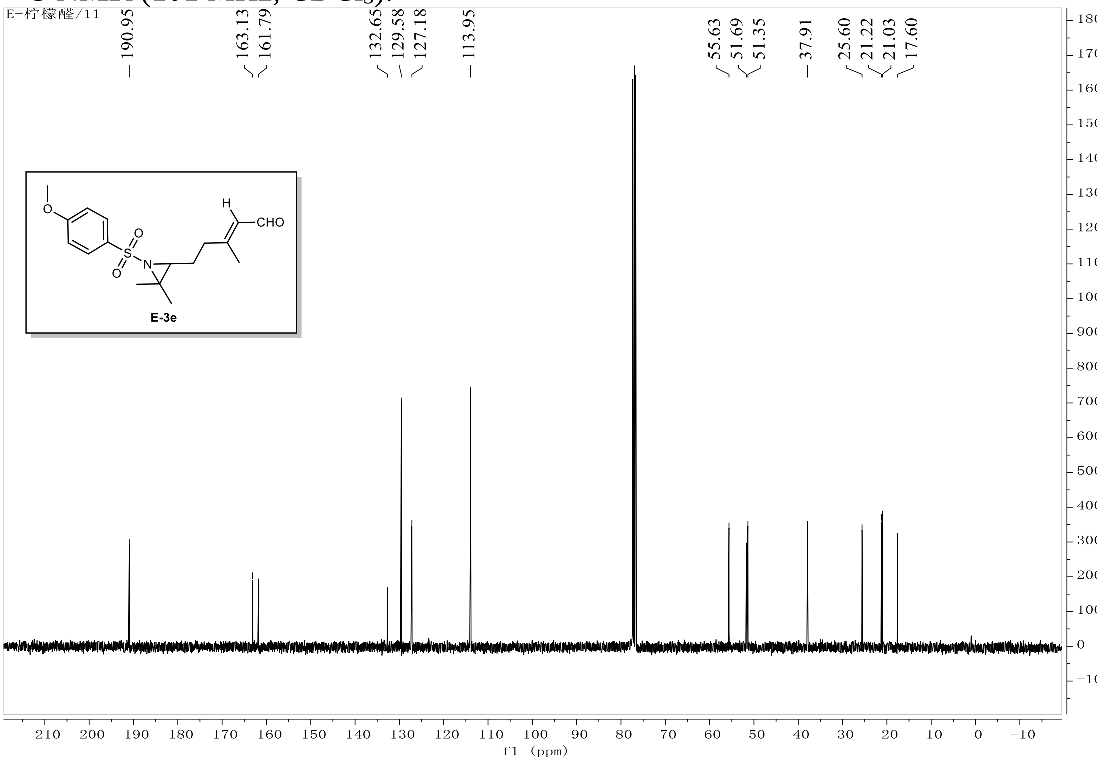

^{1}H NMR (400 MHz, $CDCl_3$):

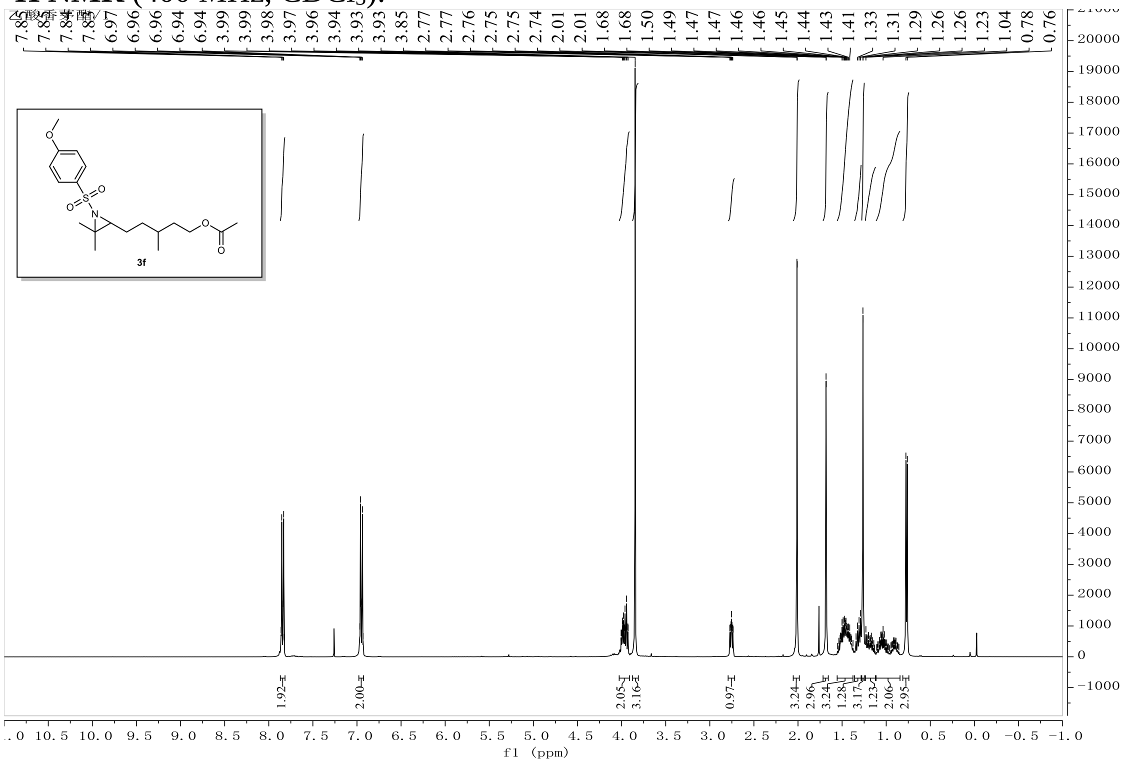

^{13}C NMR (101 MHz, $CDCl_3$):

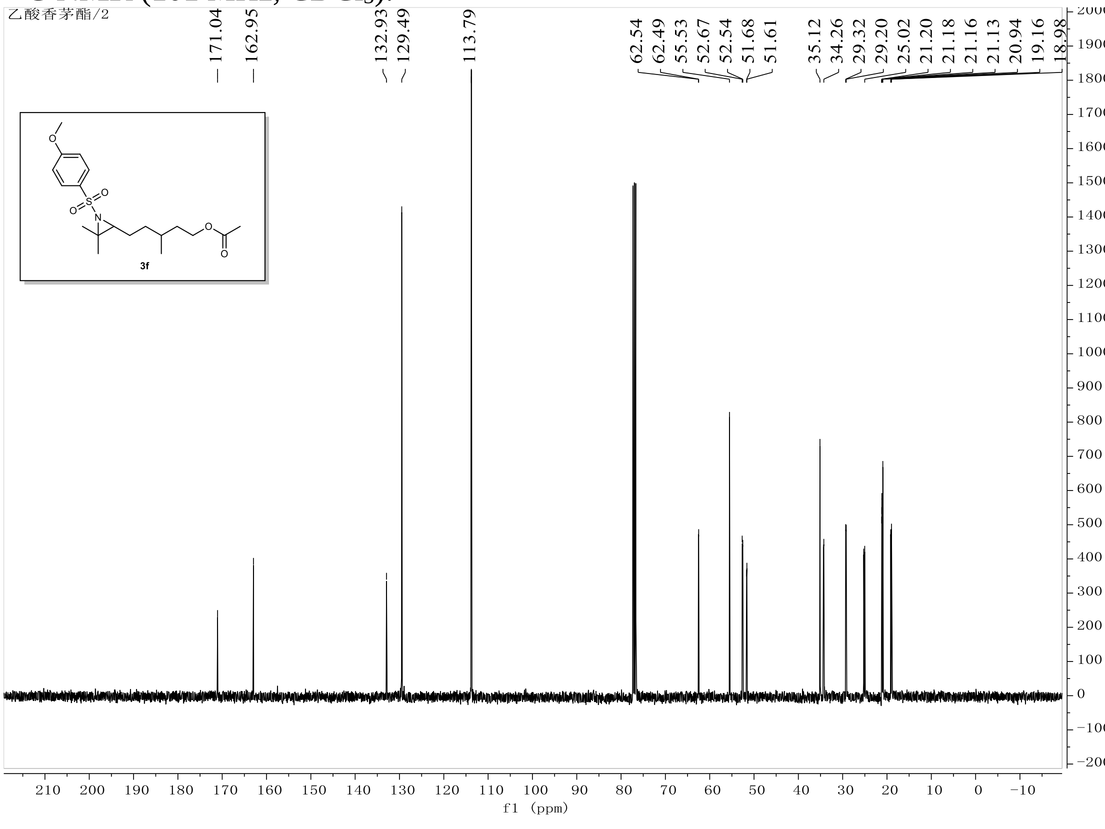

^{1}H NMR (400 MHz, $CDCl_3$):

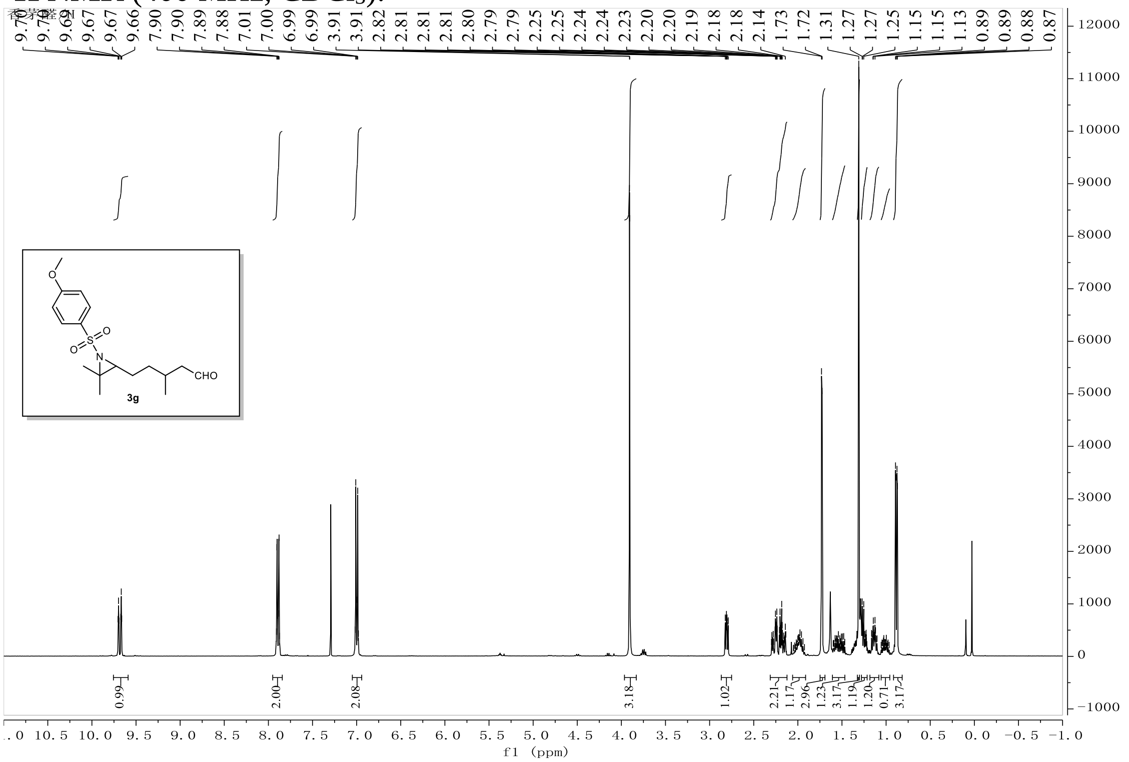

^{13}C NMR (101 MHz, $CDCl_3$):

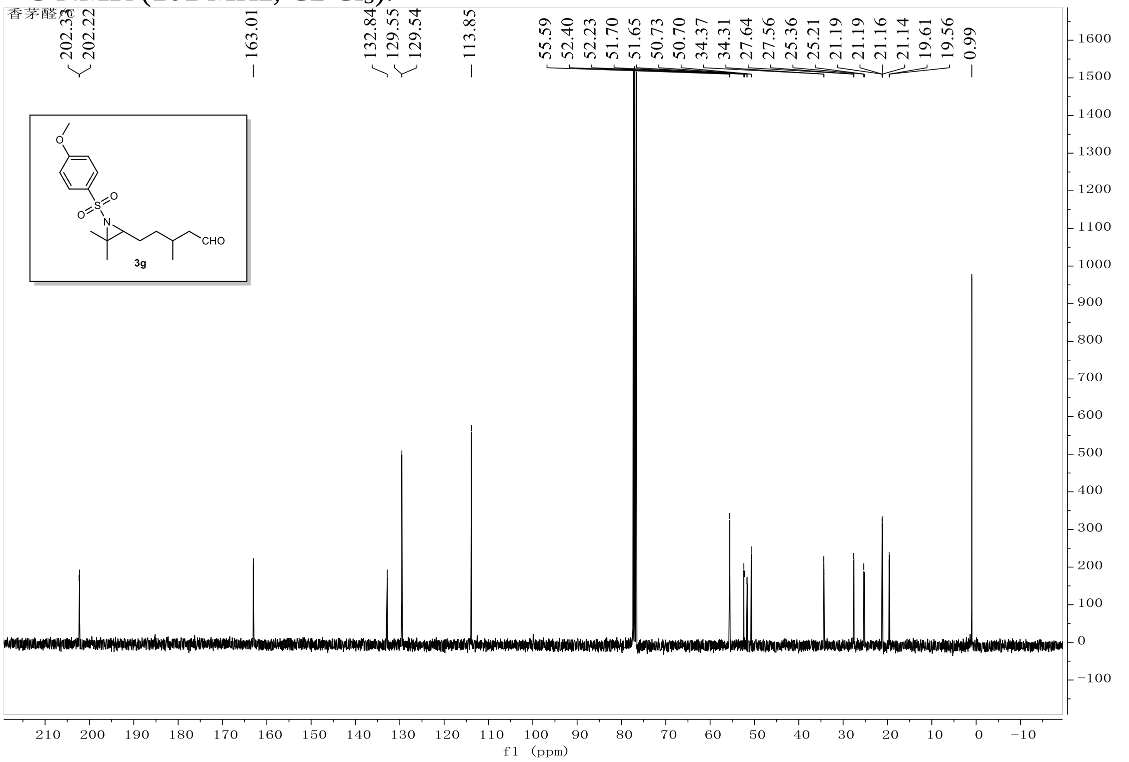

^{1}H NMR (400 MHz, $CDCl_3$):

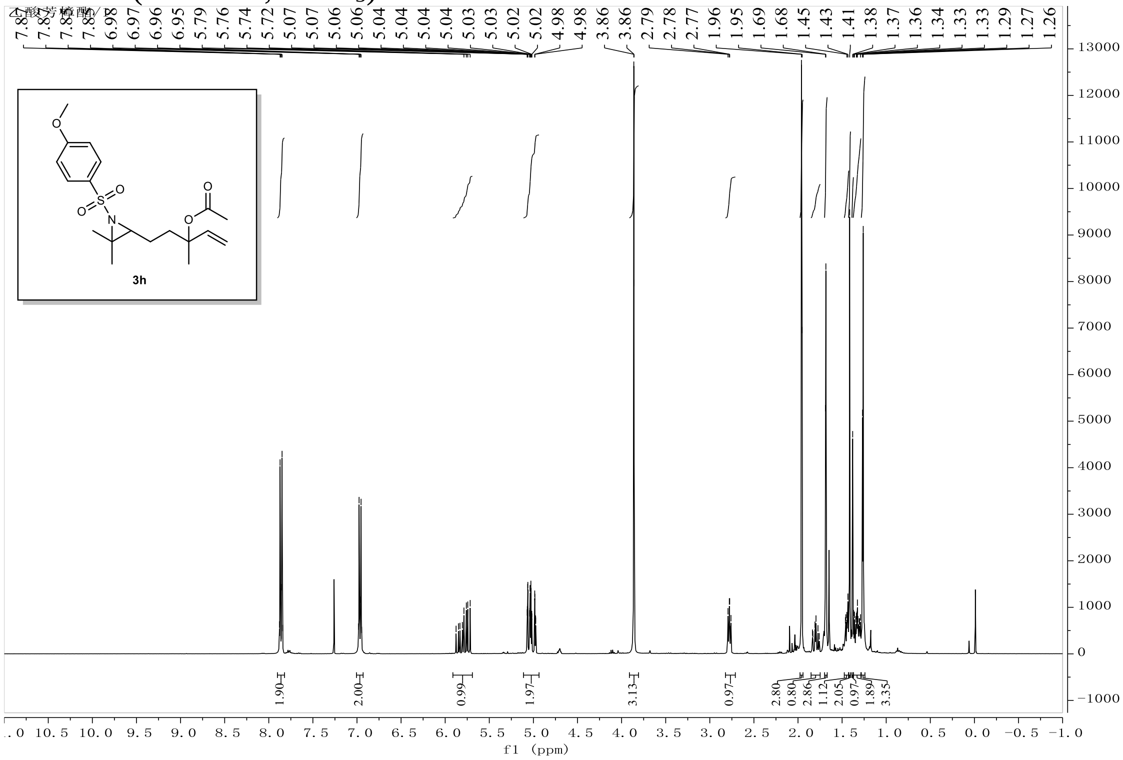

^{13}C NMR (101 MHz, $CDCl_3$):

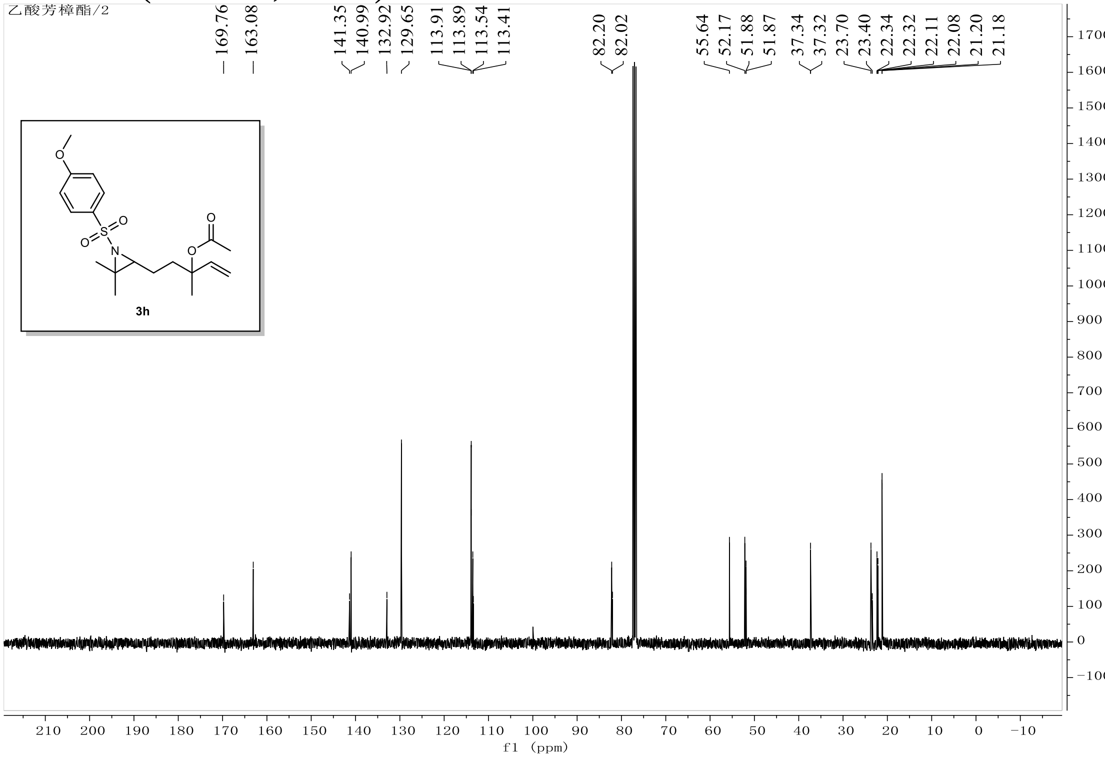

^{1}H NMR (400 MHz, $CDCl_3$):

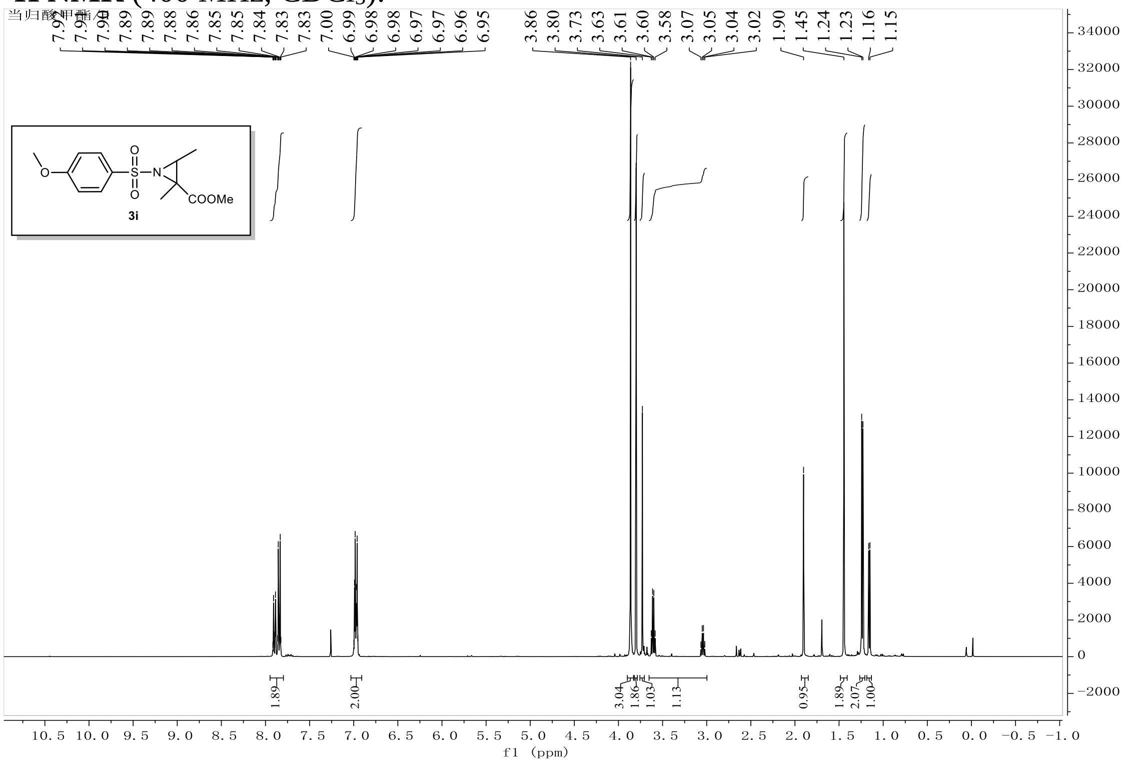

^{13}C NMR (101 MHz, $CDCl_3$):

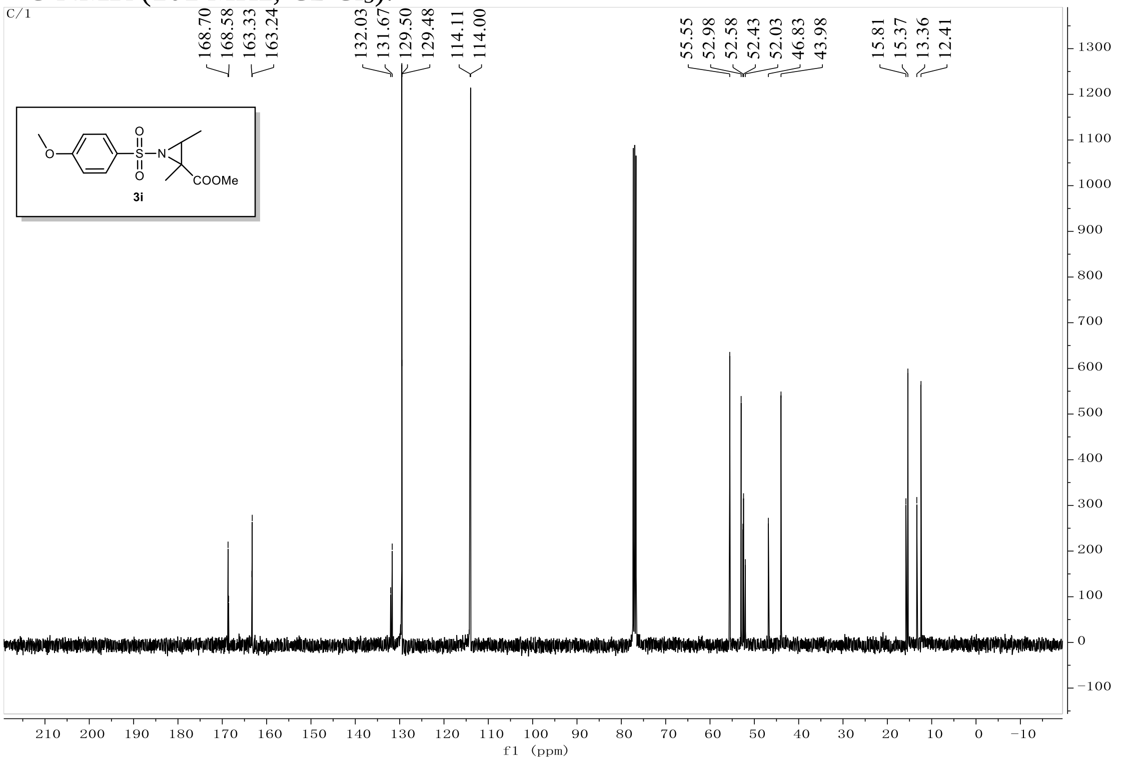

^{1}H NMR (400 MHz, $CDCl_3$):

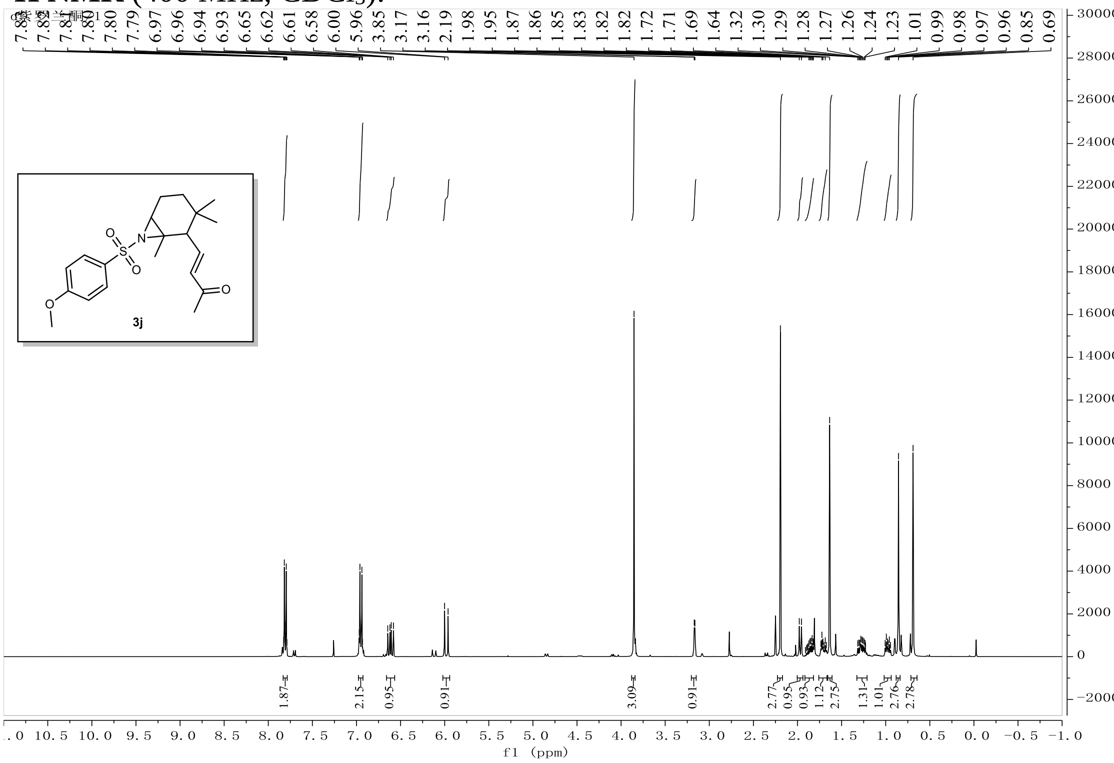

^{13}C NMR (101 MHz, $CDCl_3$):

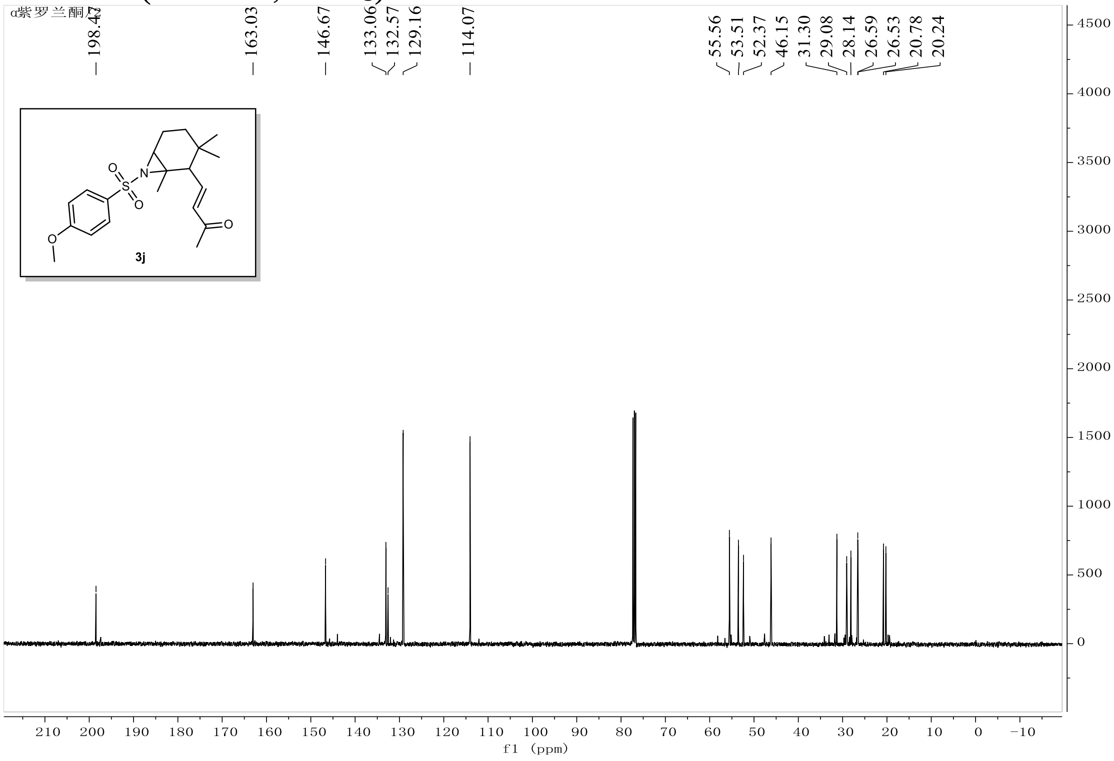

^{1}H NMR (400 MHz, $CDCl_3$):

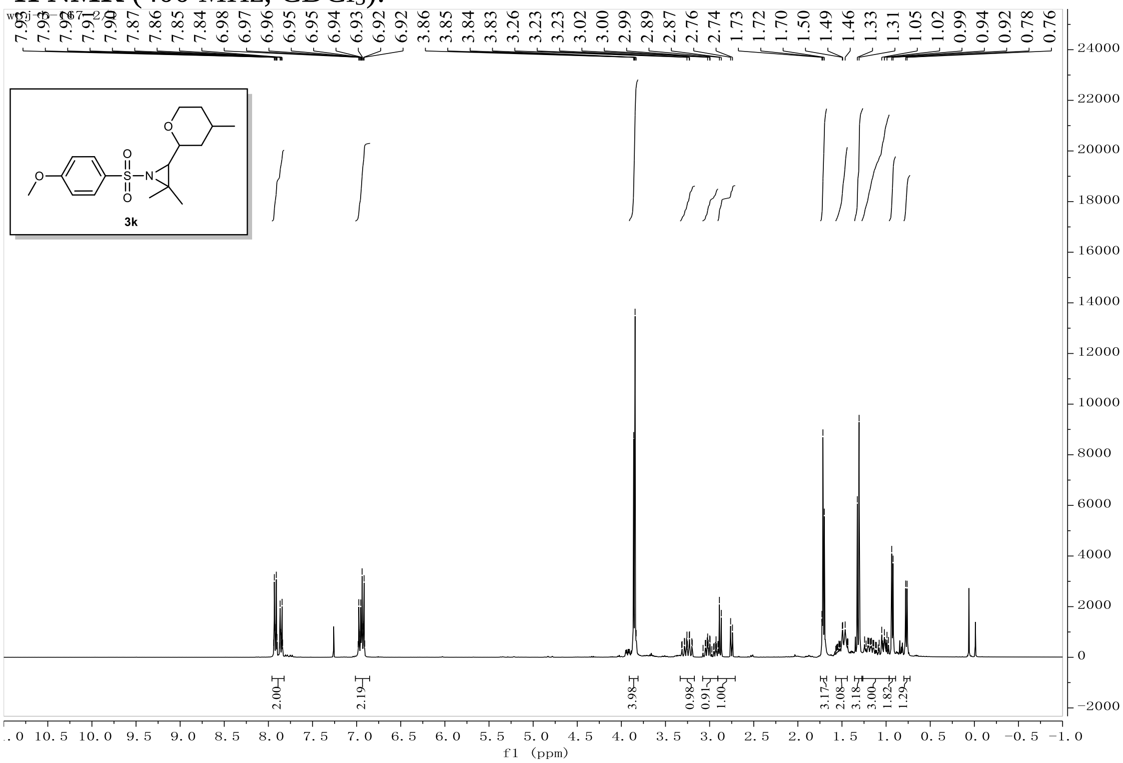

^{13}C NMR (101 MHz, $CDCl_3$):

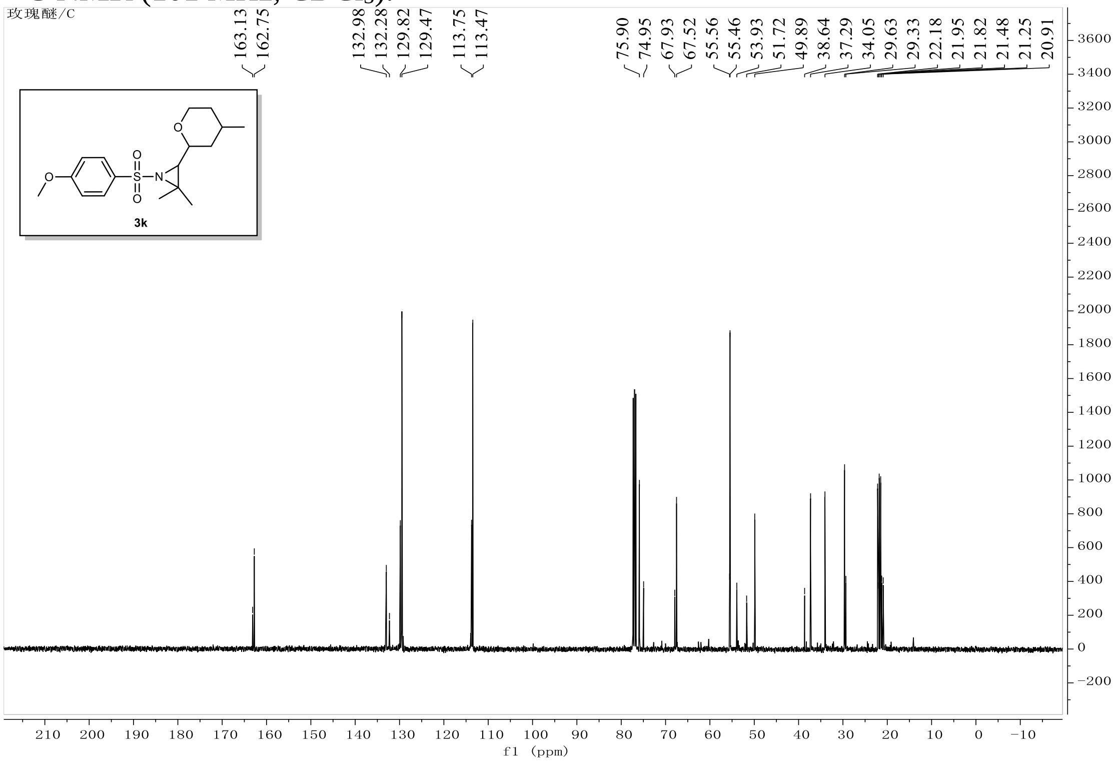

¹H NMR (400 MHz, $CDCl_3$):

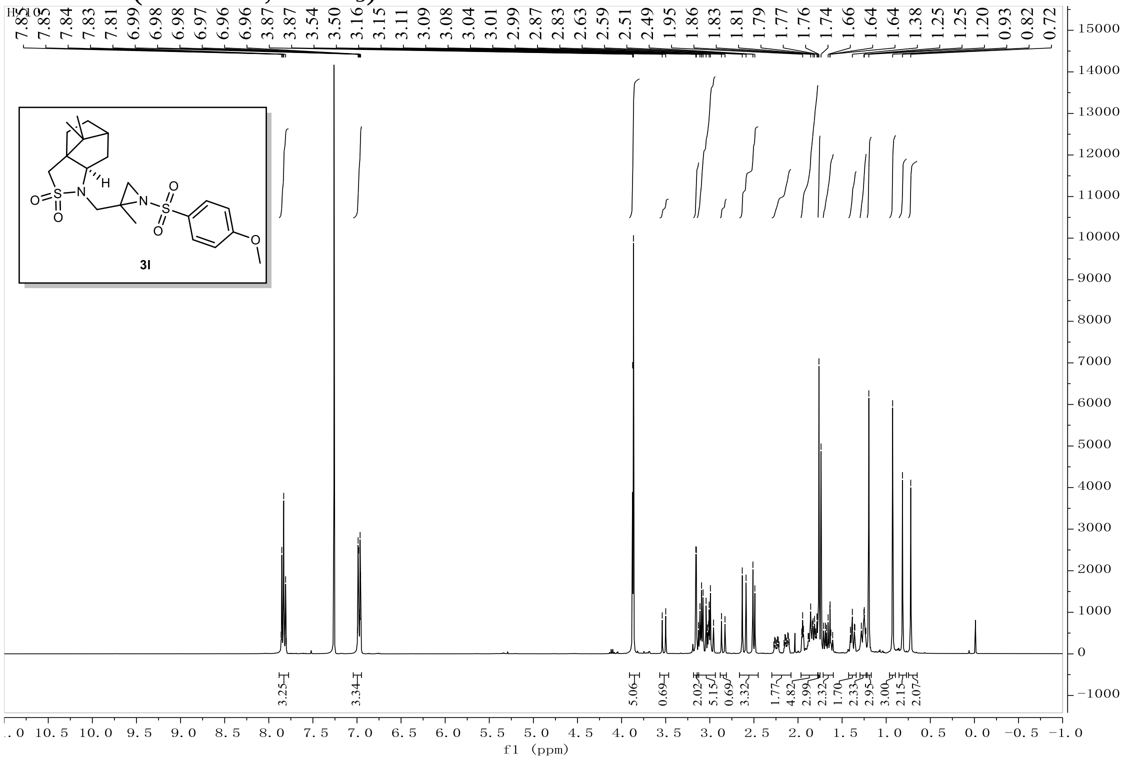

¹³C NMR (101 MHz, $CDCl_3$):

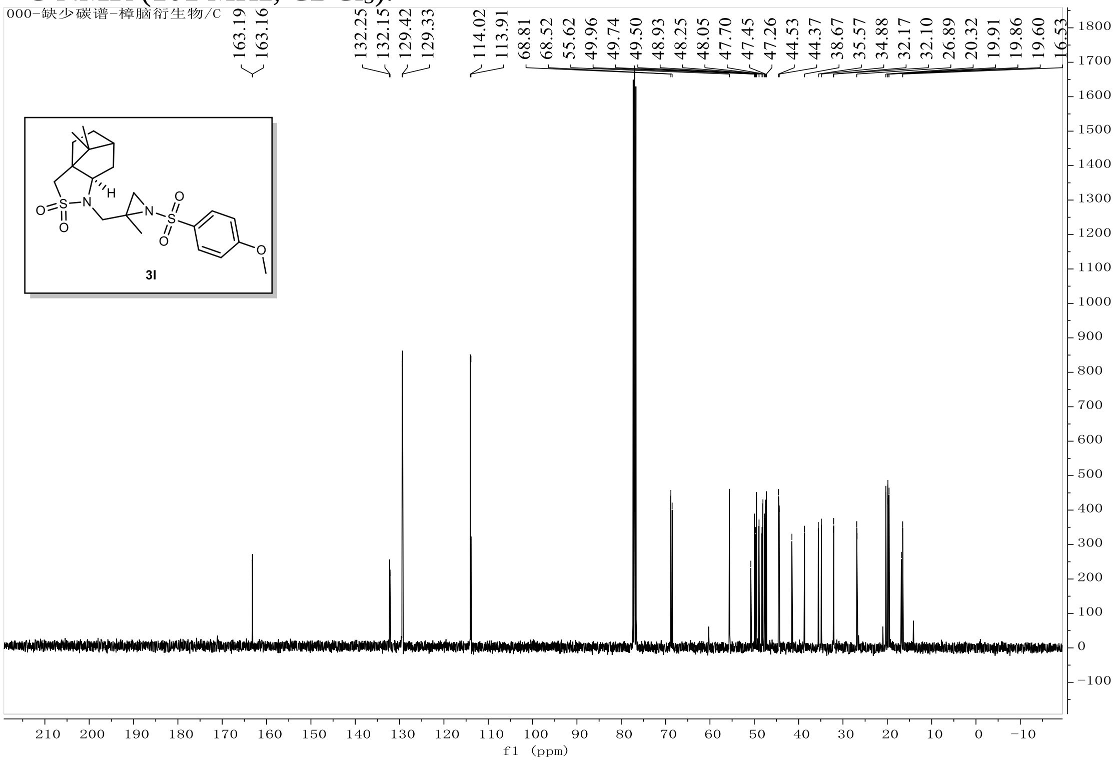

^{1}H NMR (400 MHz, $CDCl_3$):

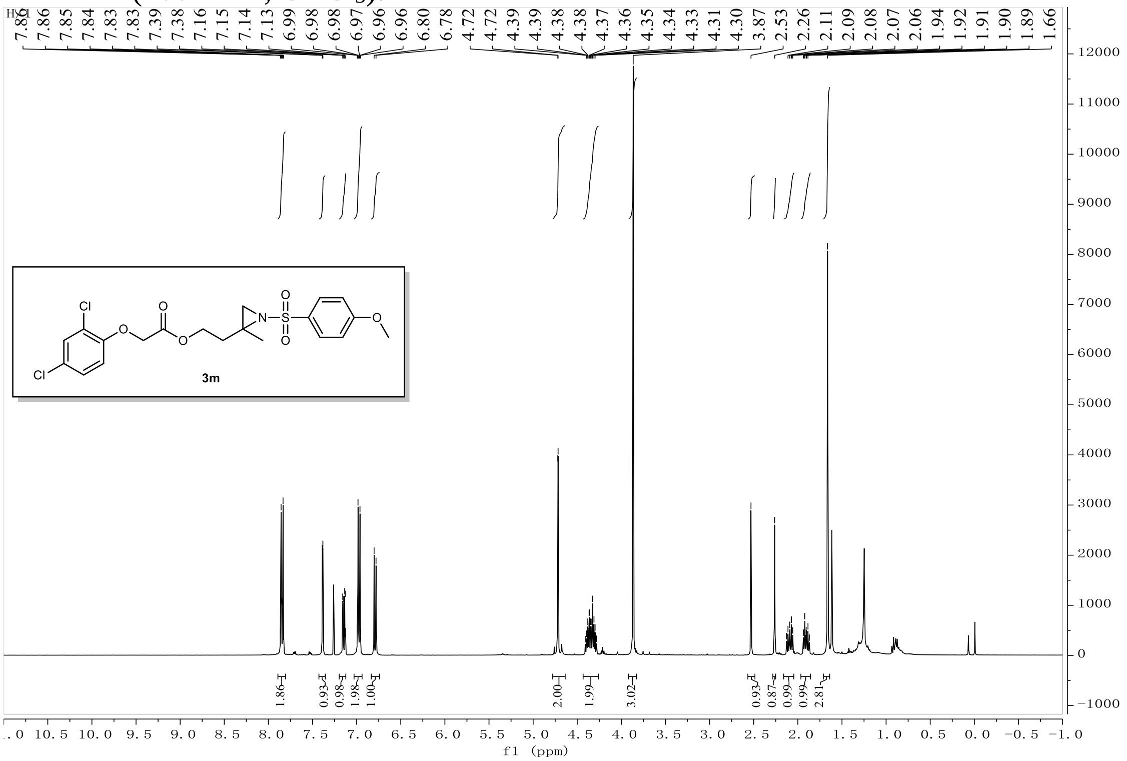

^{13}C NMR (101 MHz, $CDCl_3$):

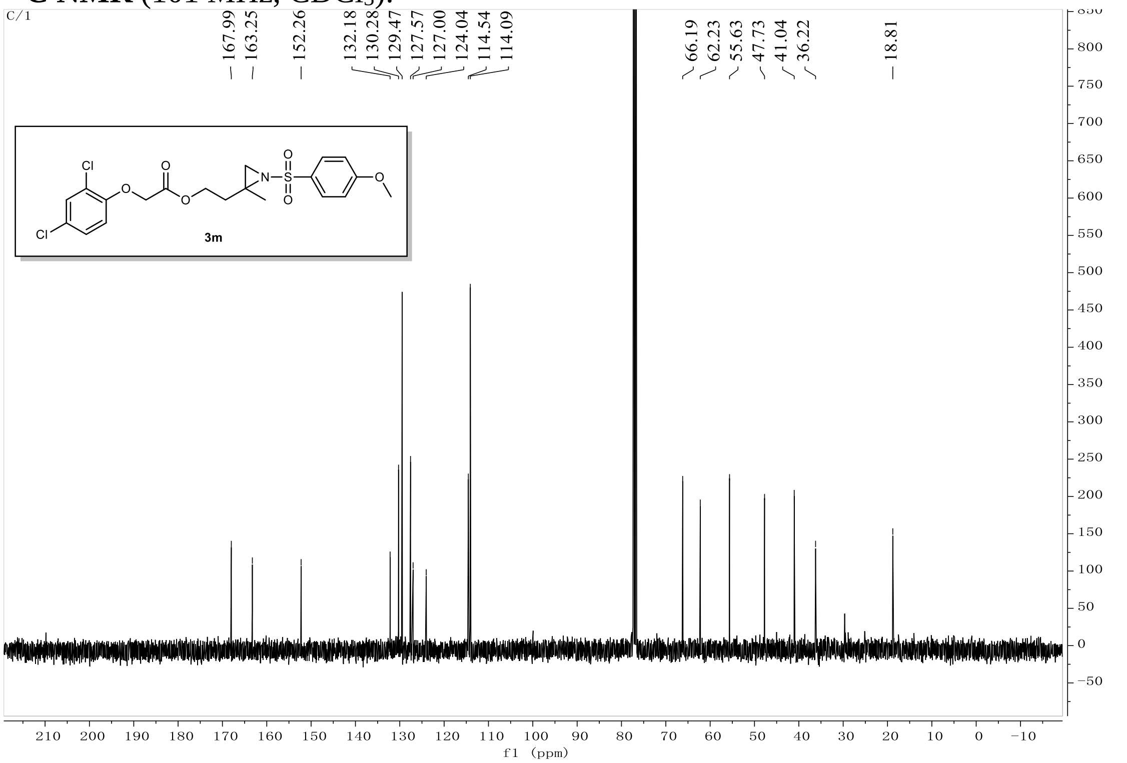

^{1}H NMR (400 MHz, $CDCl_3$):

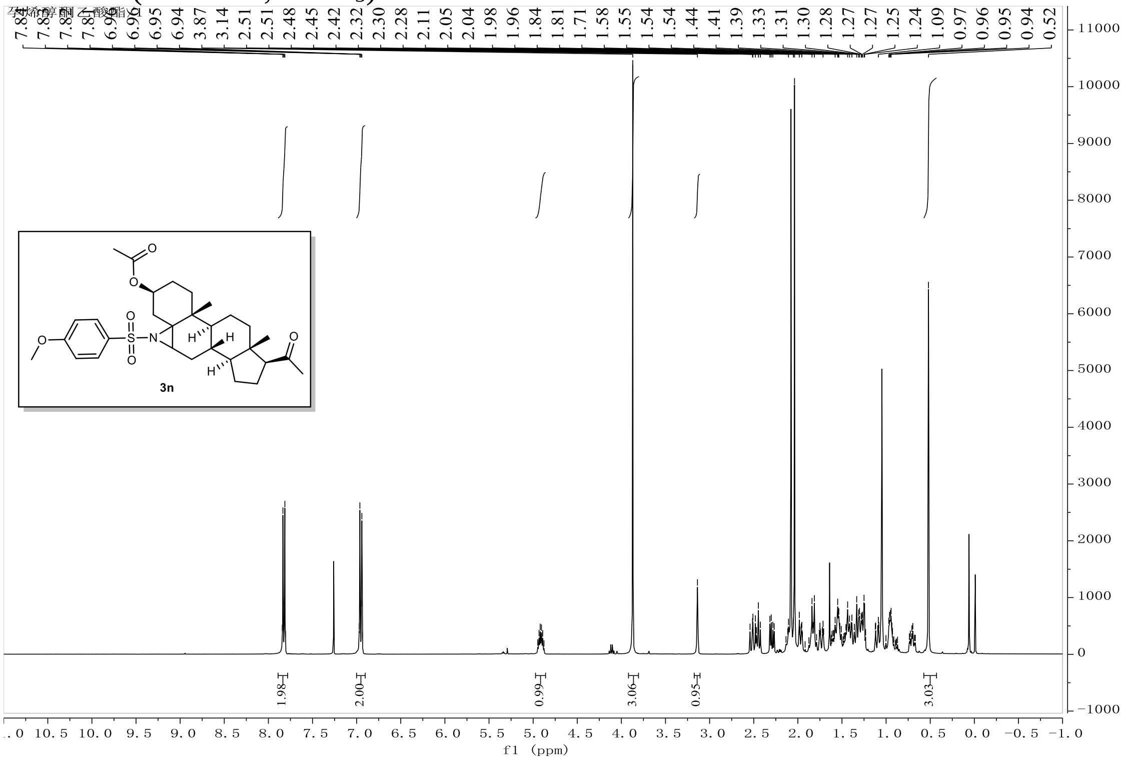

^{13}C NMR (101 MHz, $CDCl_3$):

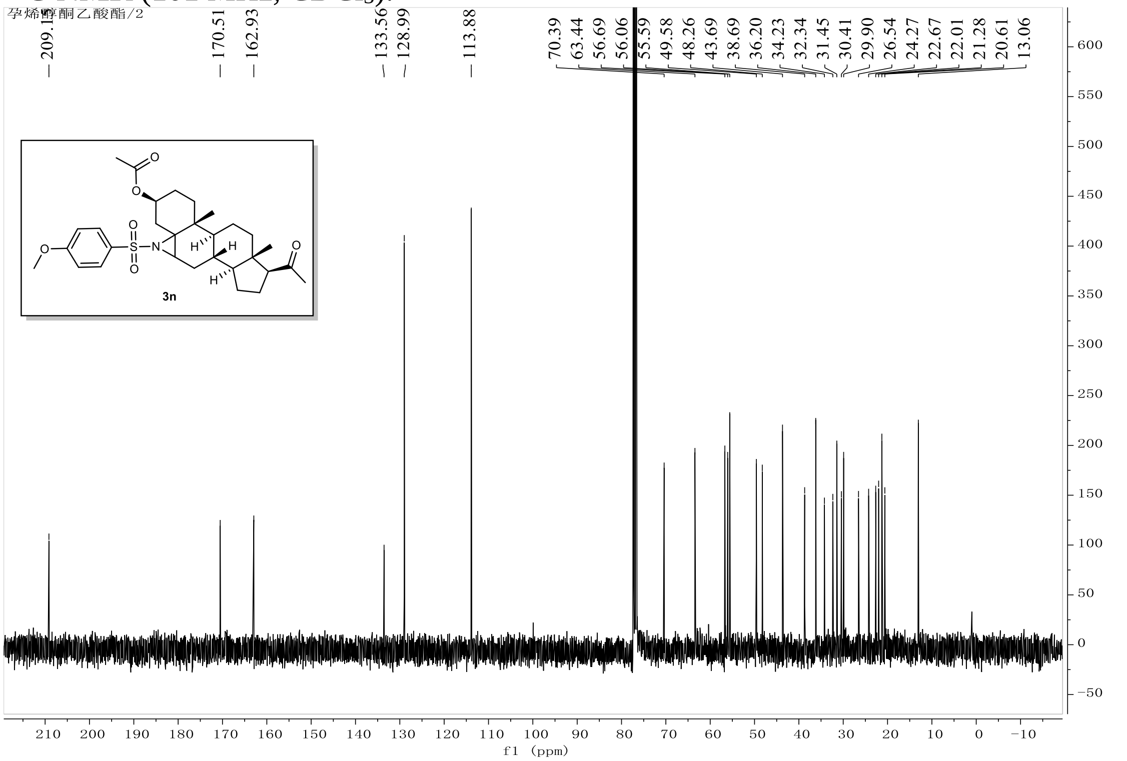

^{1}H NMR (400 MHz, $CDCl_3$):

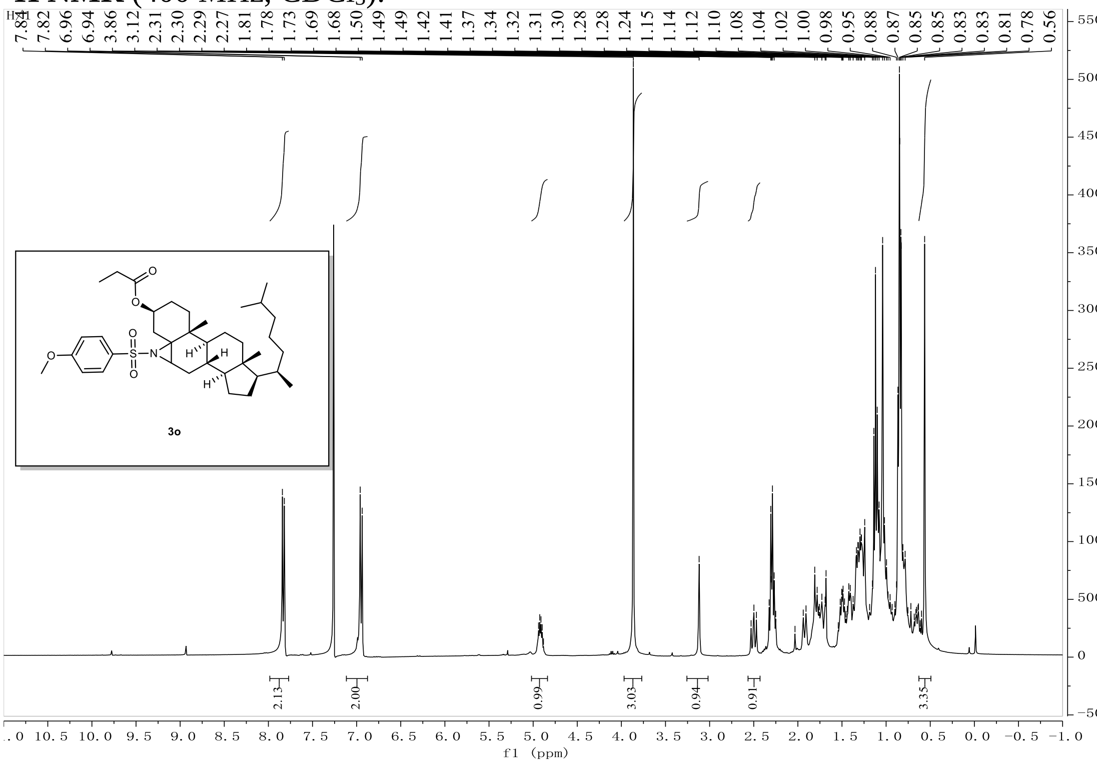

^{13}C NMR (101 MHz, $CDCl_3$):

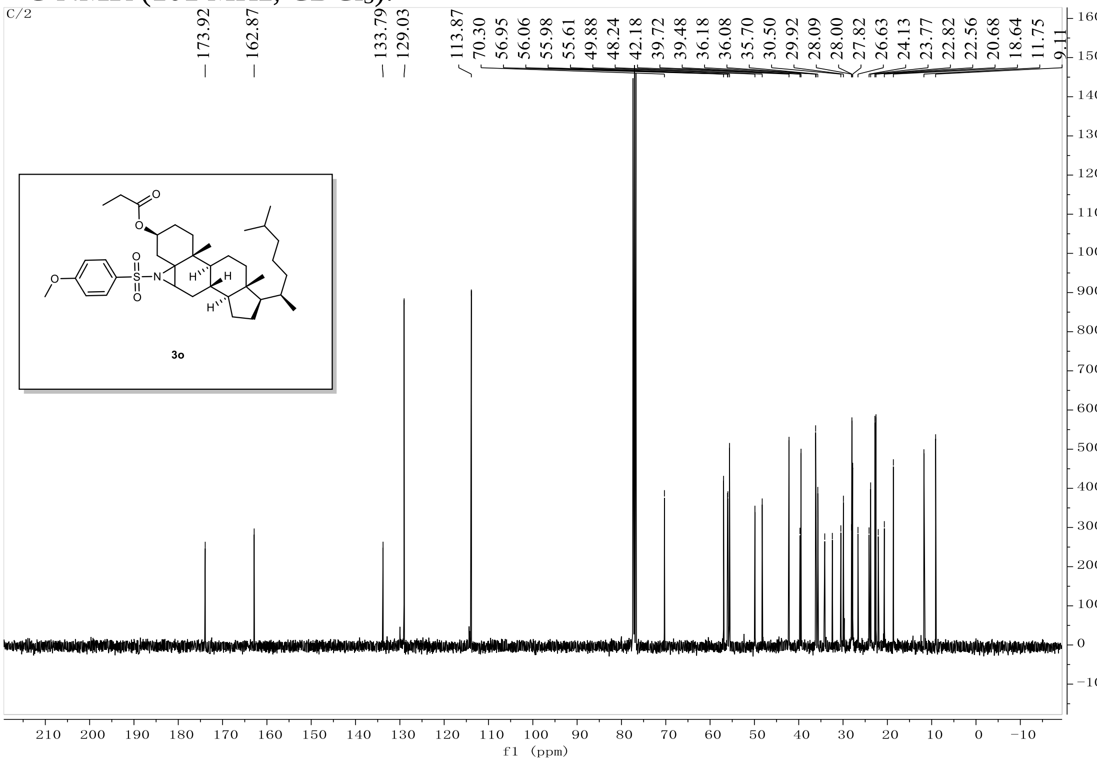

^{1}H NMR (400 MHz, $CDCl_3$):

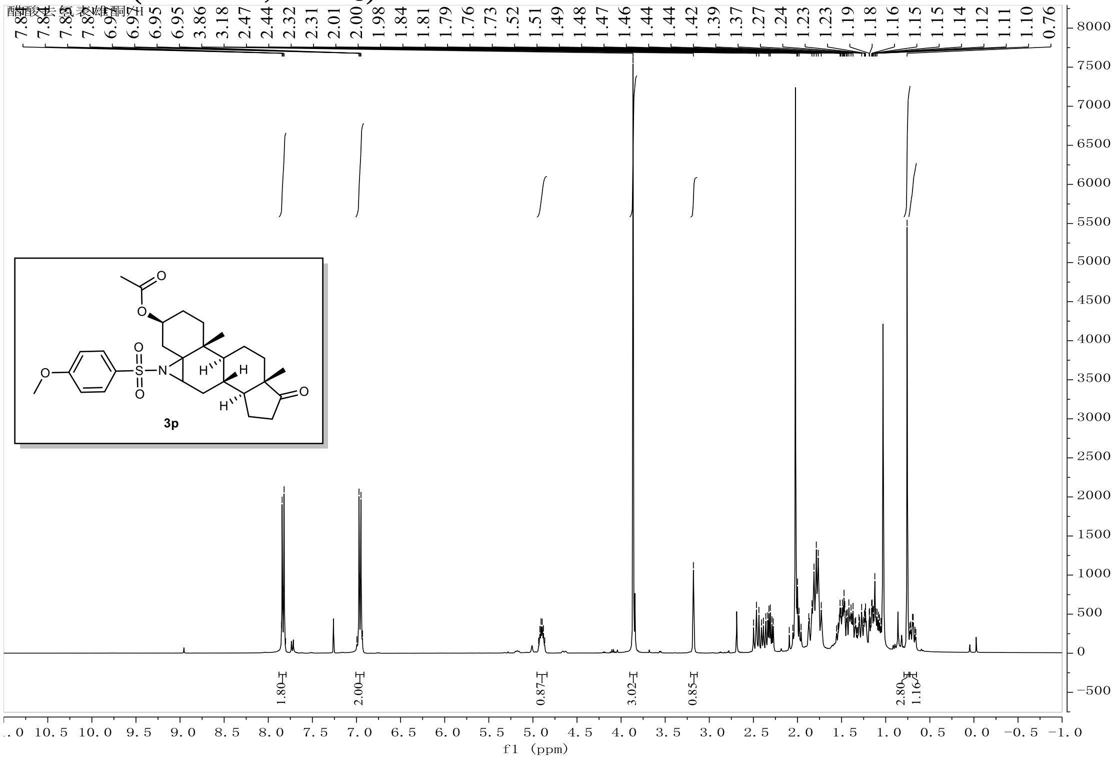

^{13}C NMR (101 MHz, $CDCl_3$):

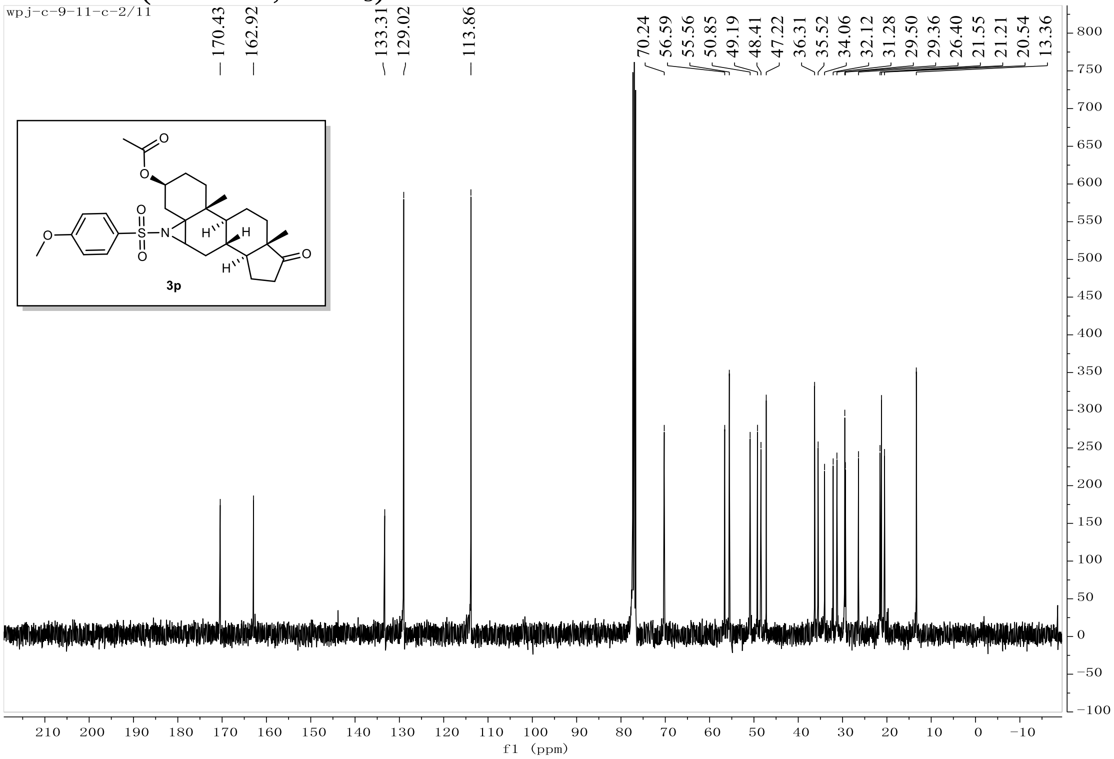

^{1}H NMR (400 MHz, $CDCl_3$):

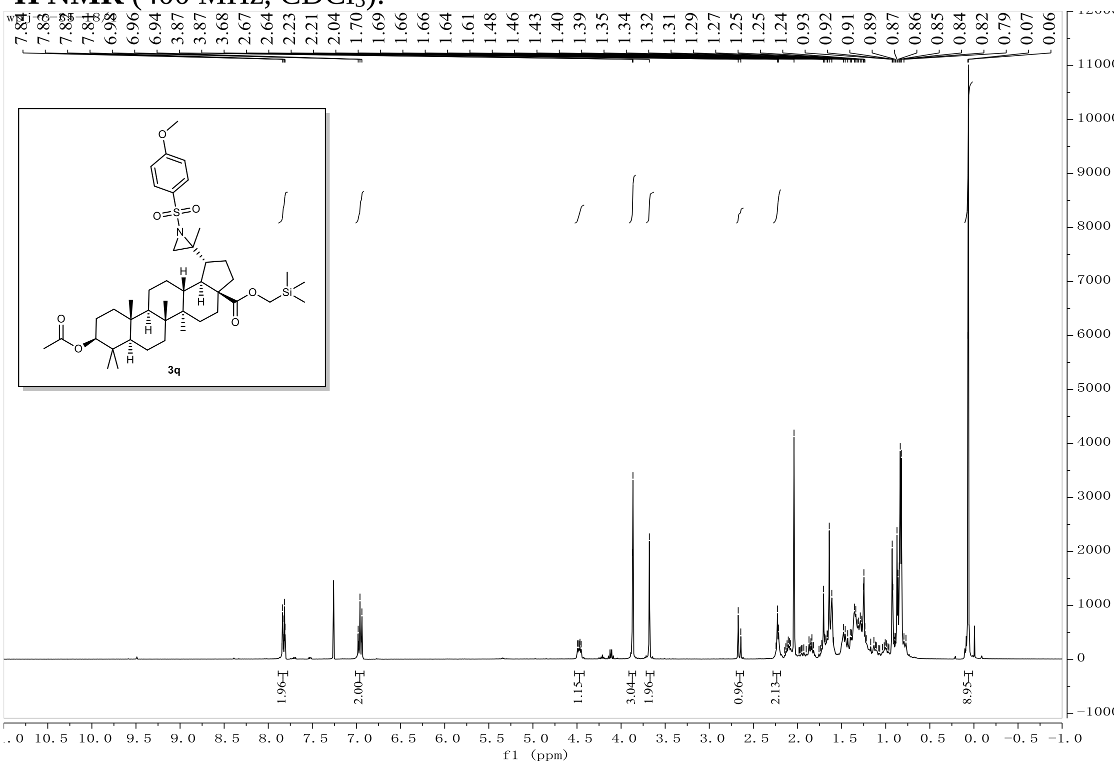

^{13}C NMR (101 MHz, $CDCl_3$):

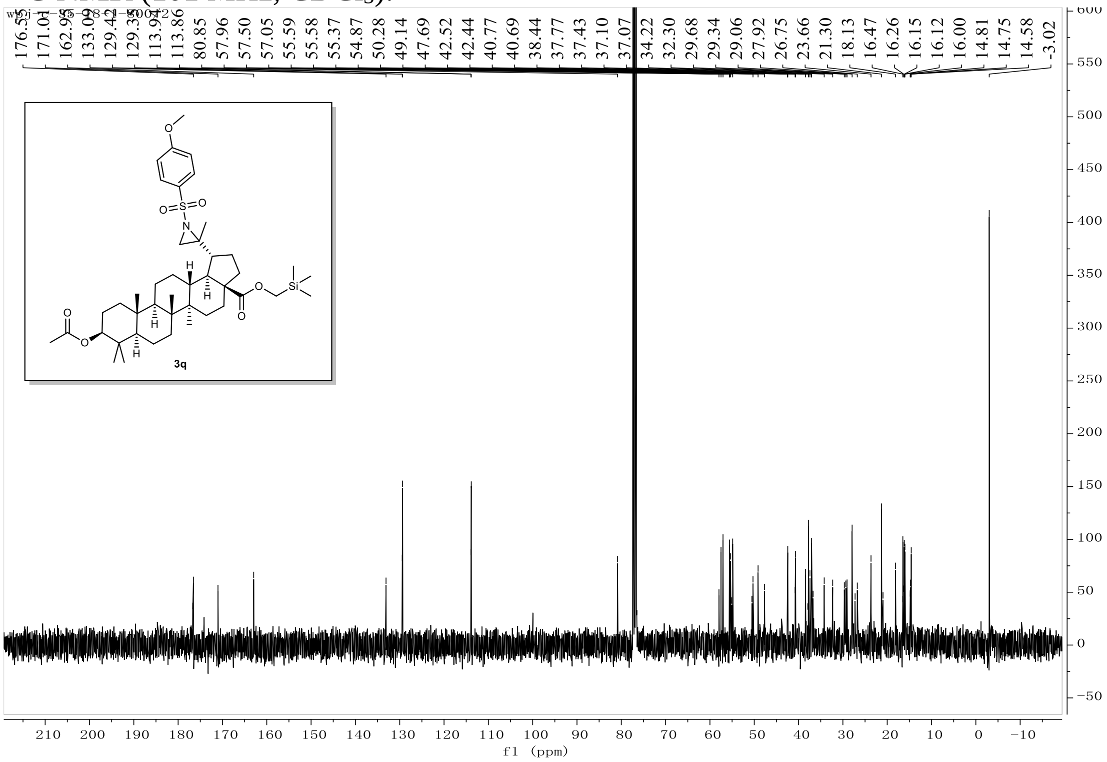

^{1}H NMR (400 MHz, $CDCl_3$):

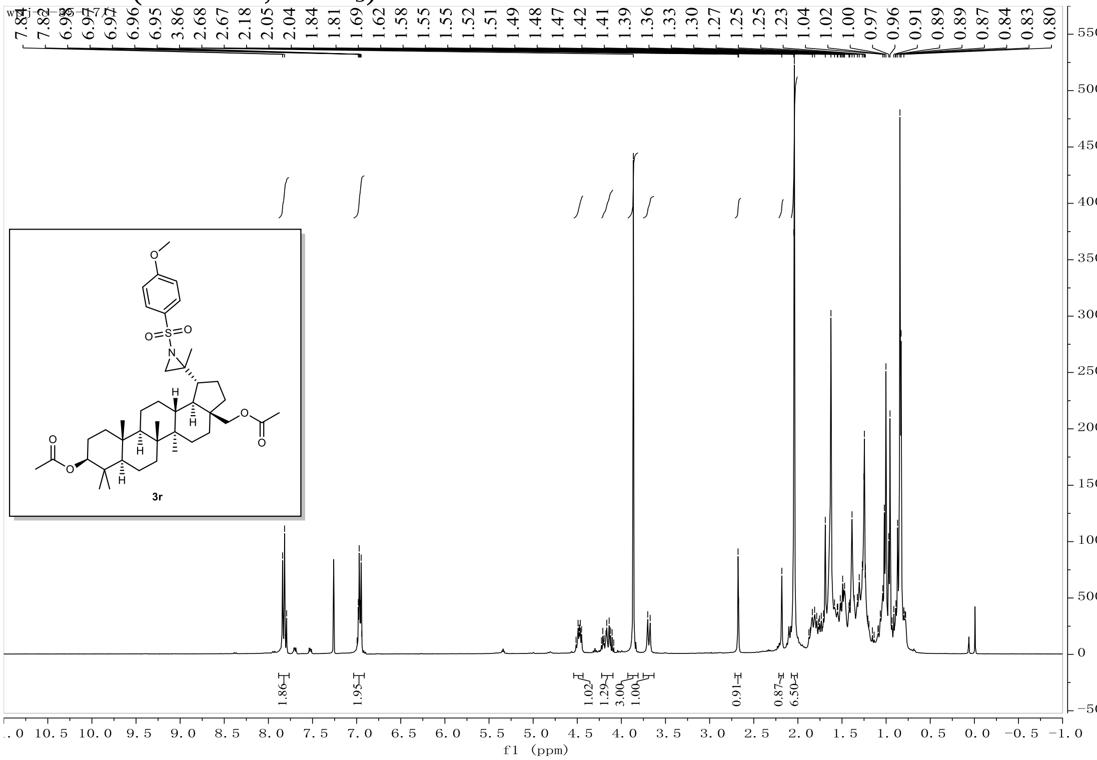

^{13}C NMR (101 MHz, $CDCl_3$):

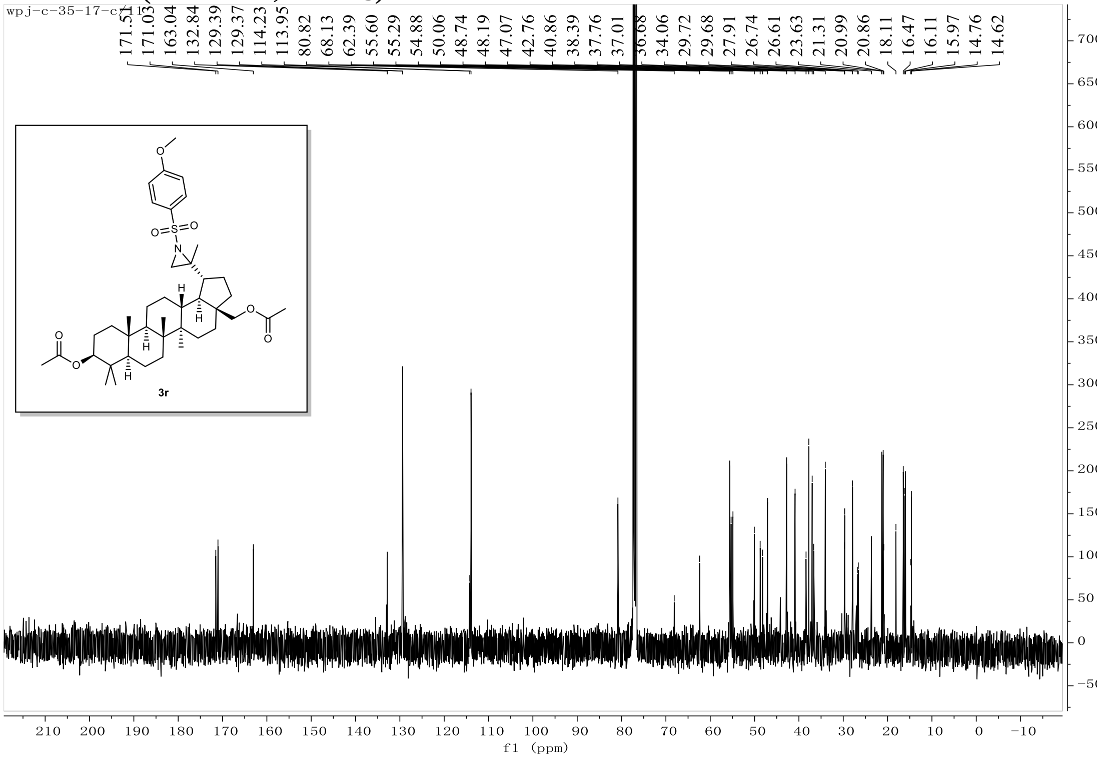

^{1}H NMR (400 MHz, $CDCl_3$):

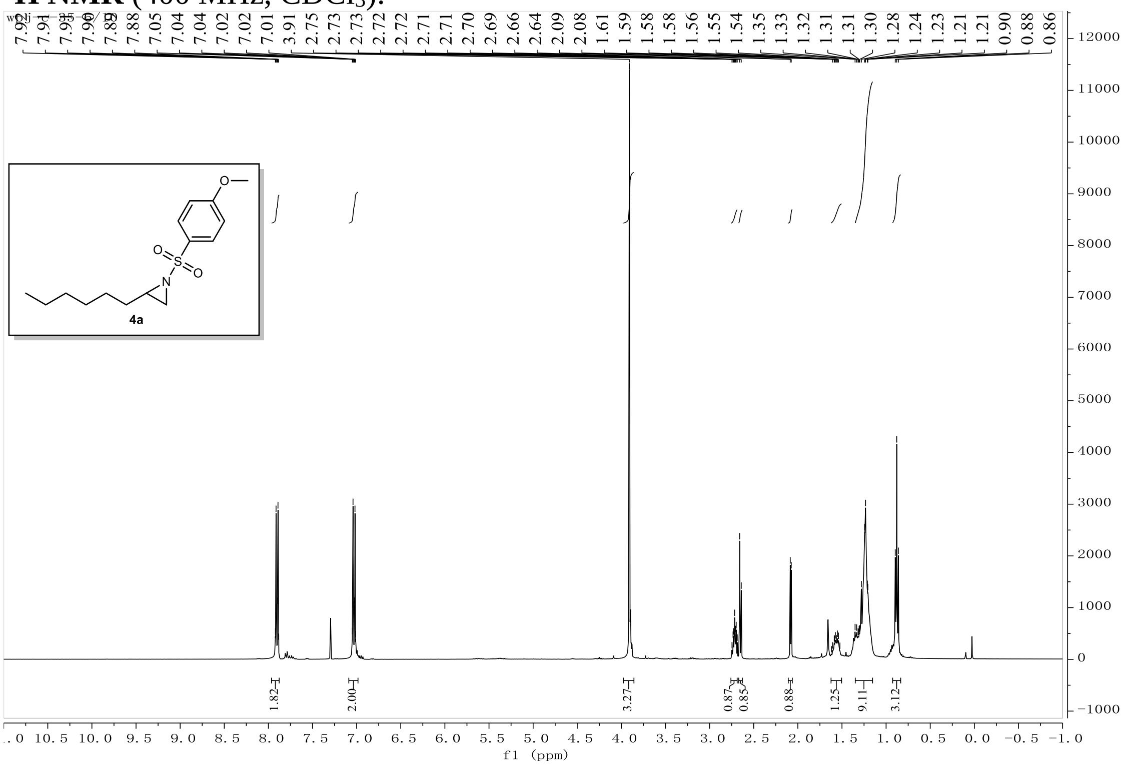

^{13}C NMR (101 MHz, $CDCl_3$):

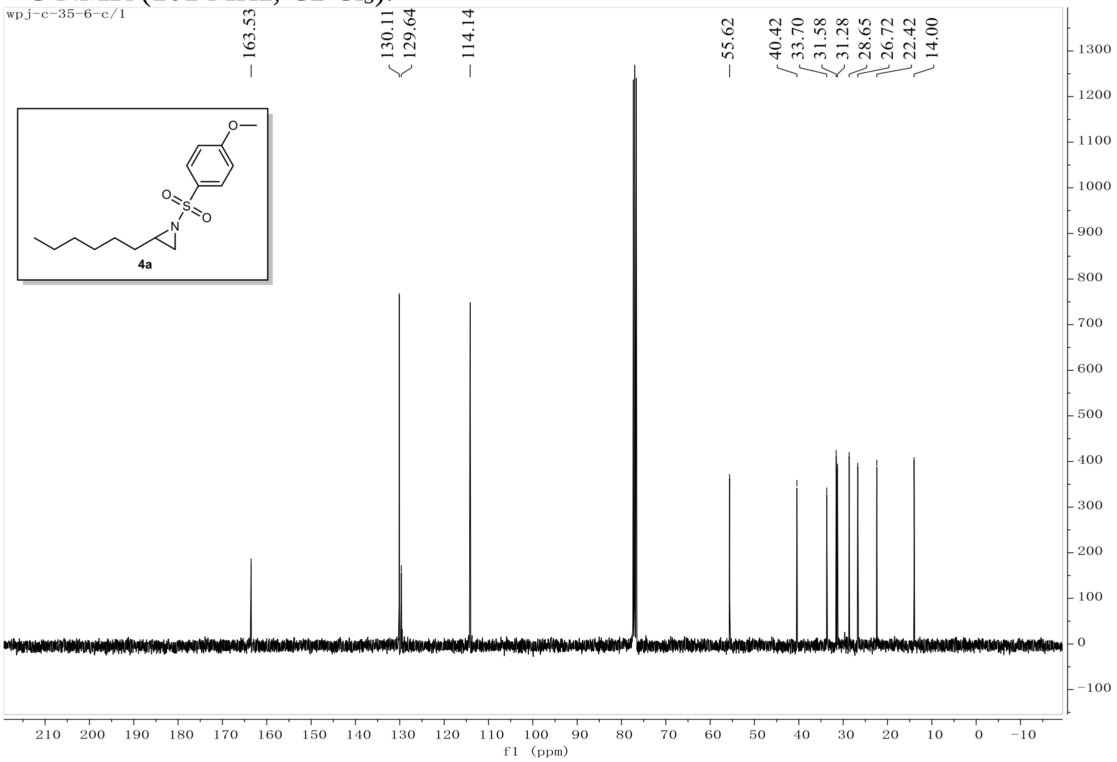

^{1}H NMR (400 MHz, $CDCl_3$):

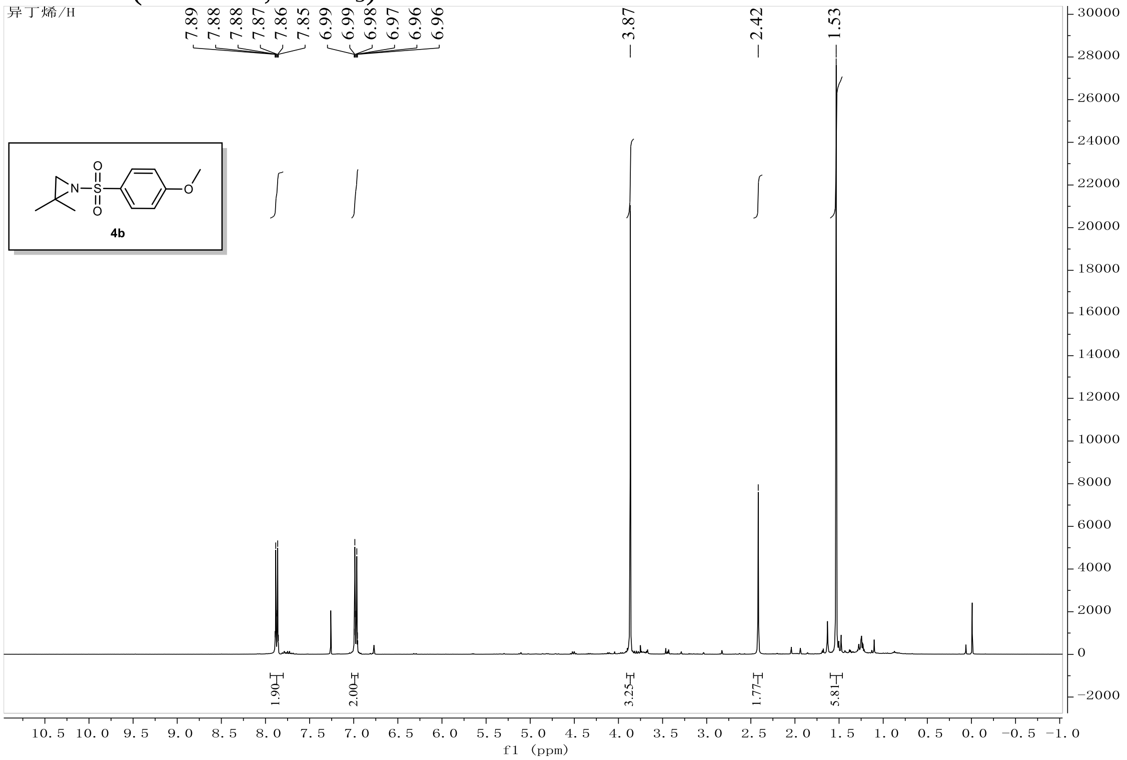

^{13}C NMR (101 MHz, $CDCl_3$):

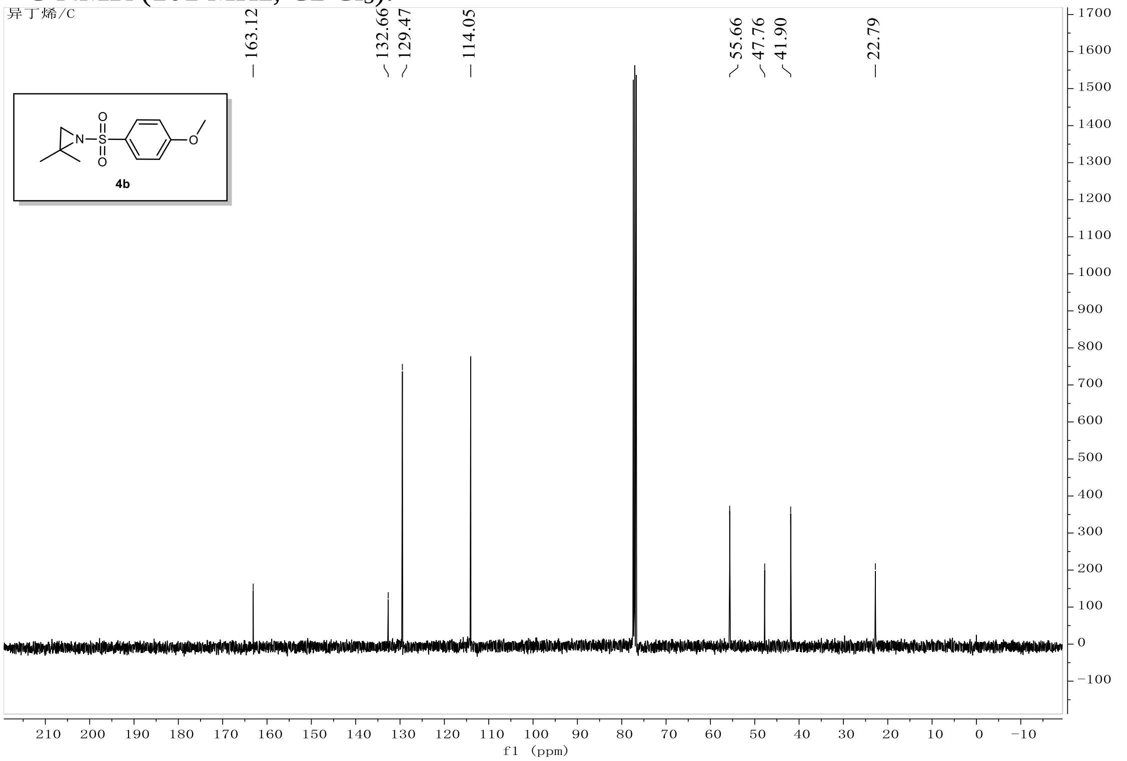

^{1}H NMR (400 MHz, $CDCl_3$):

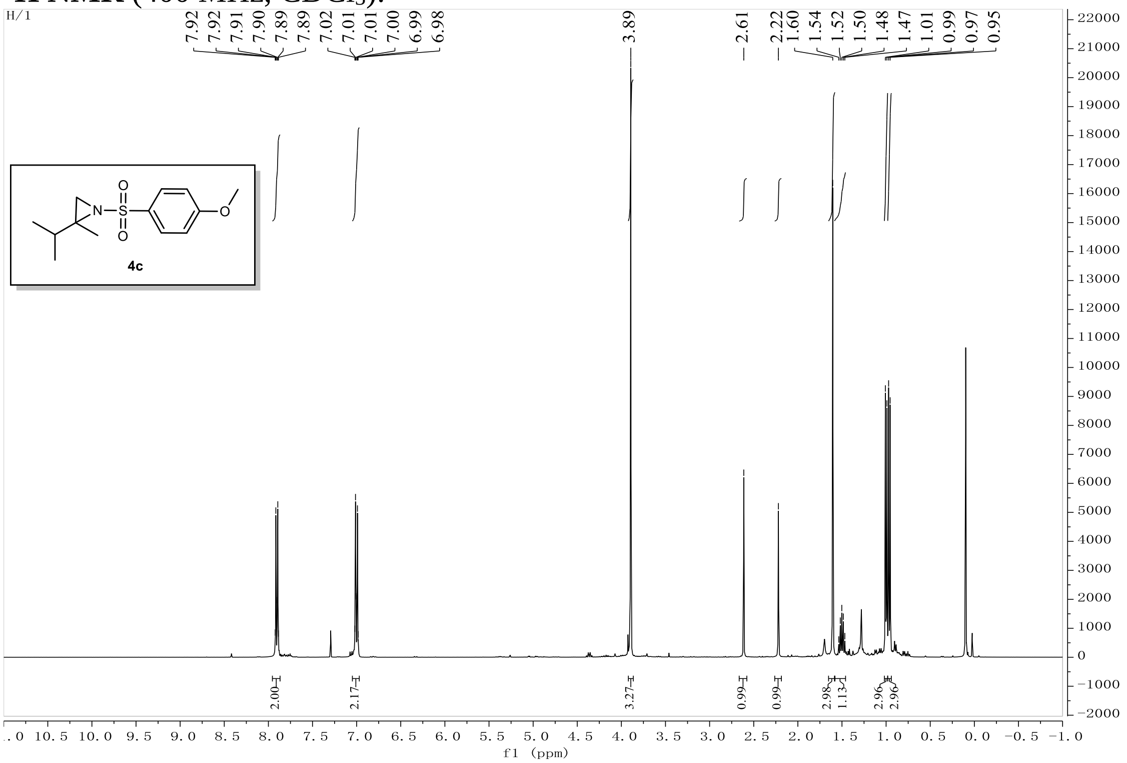

^{13}C NMR (101 MHz, $CDCl_3$):

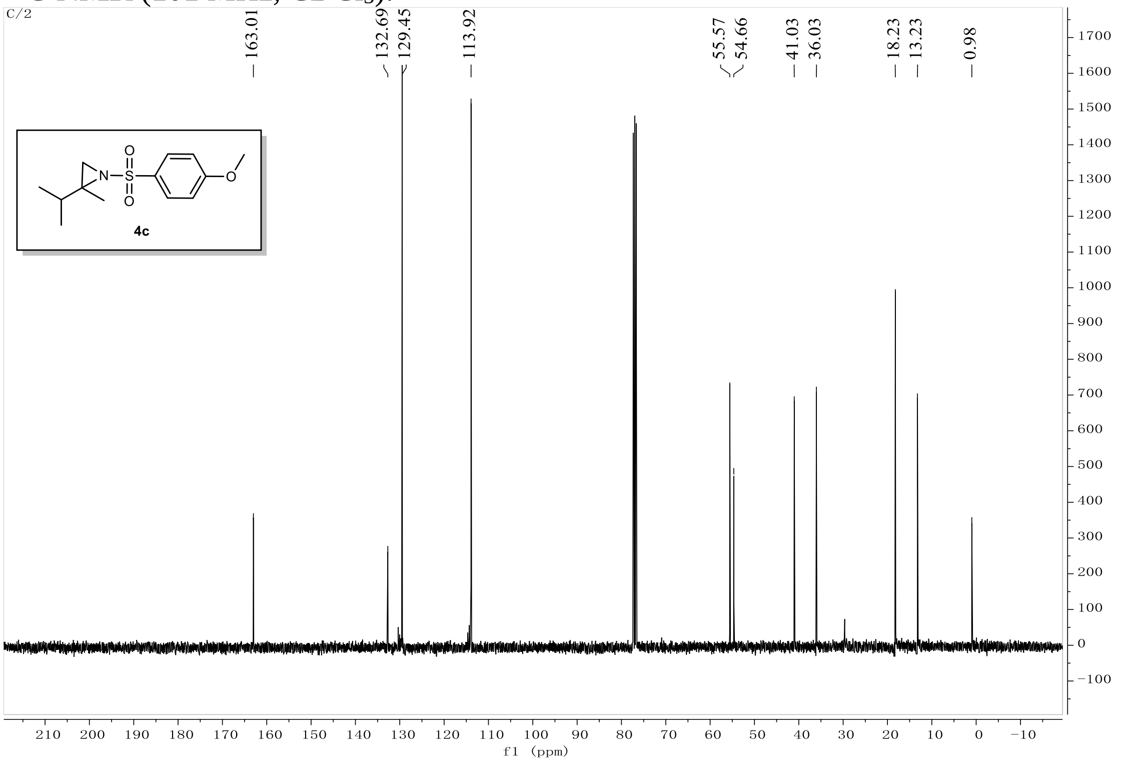

^{1}H NMR (400 MHz, $CDCl_3$):

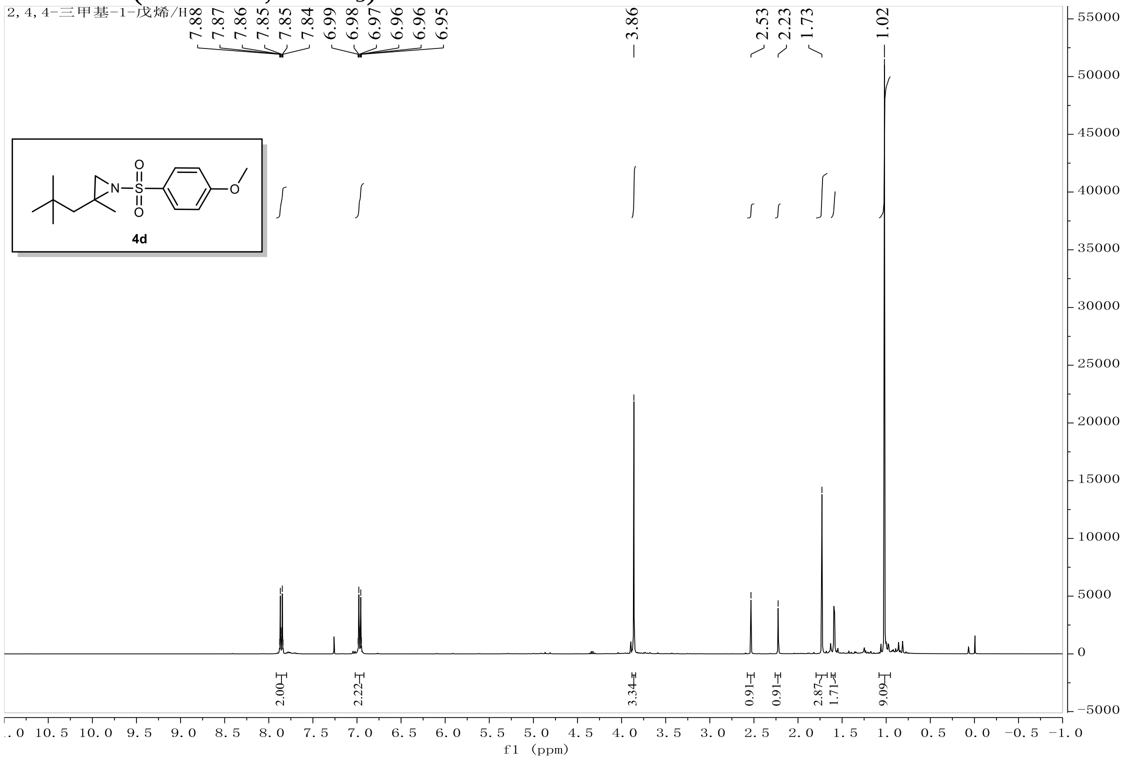

^{13}C NMR (101 MHz, $CDCl_3$):

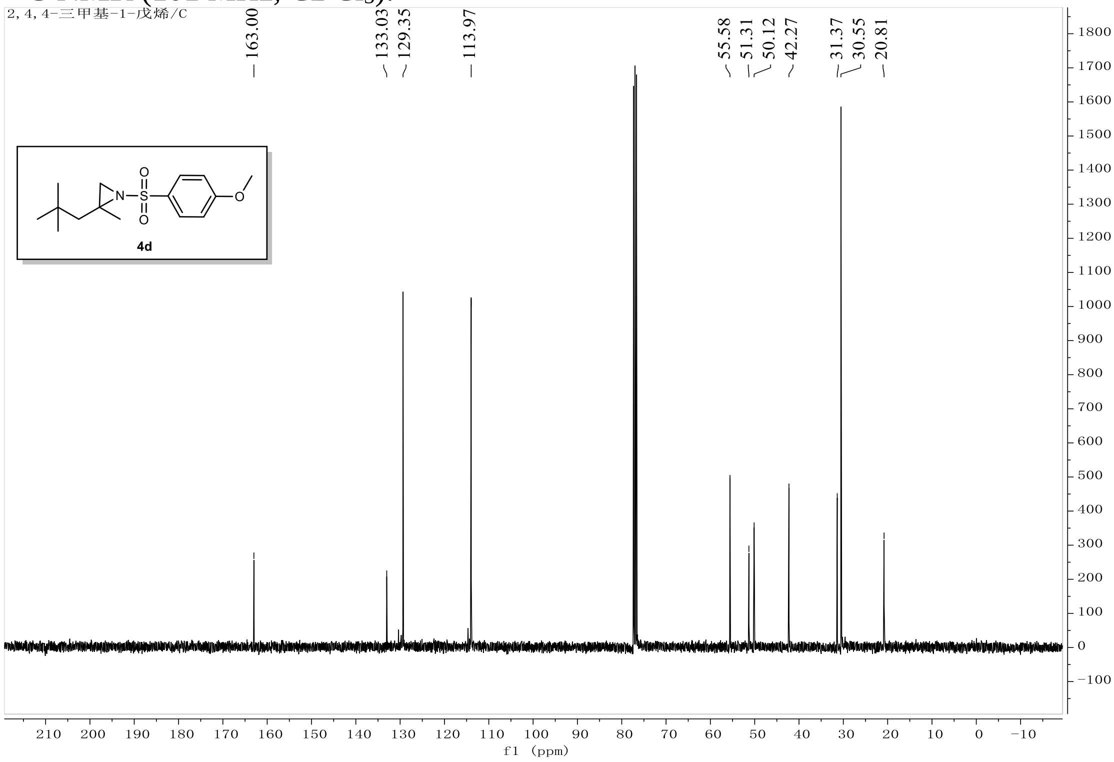

^{1}H NMR (400 MHz, $CDCl_3$):

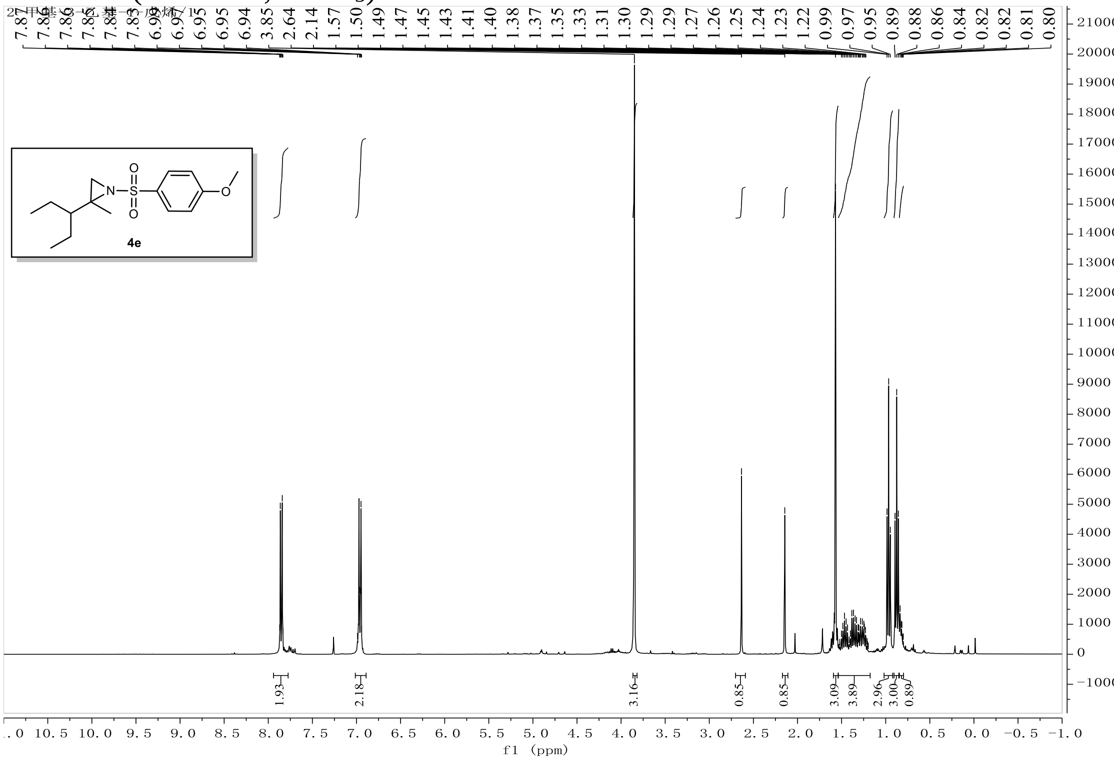

^{13}C NMR (101 MHz, $CDCl_3$):

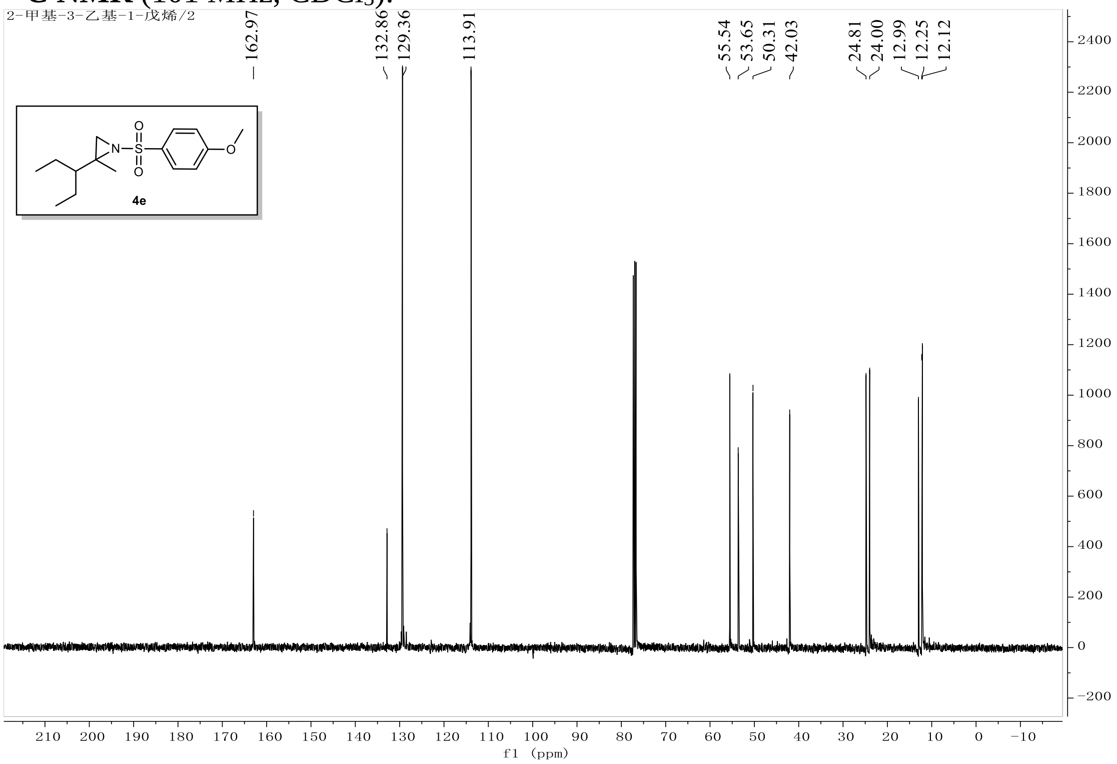

^{1}H NMR (400 MHz, $CDCl_3$):

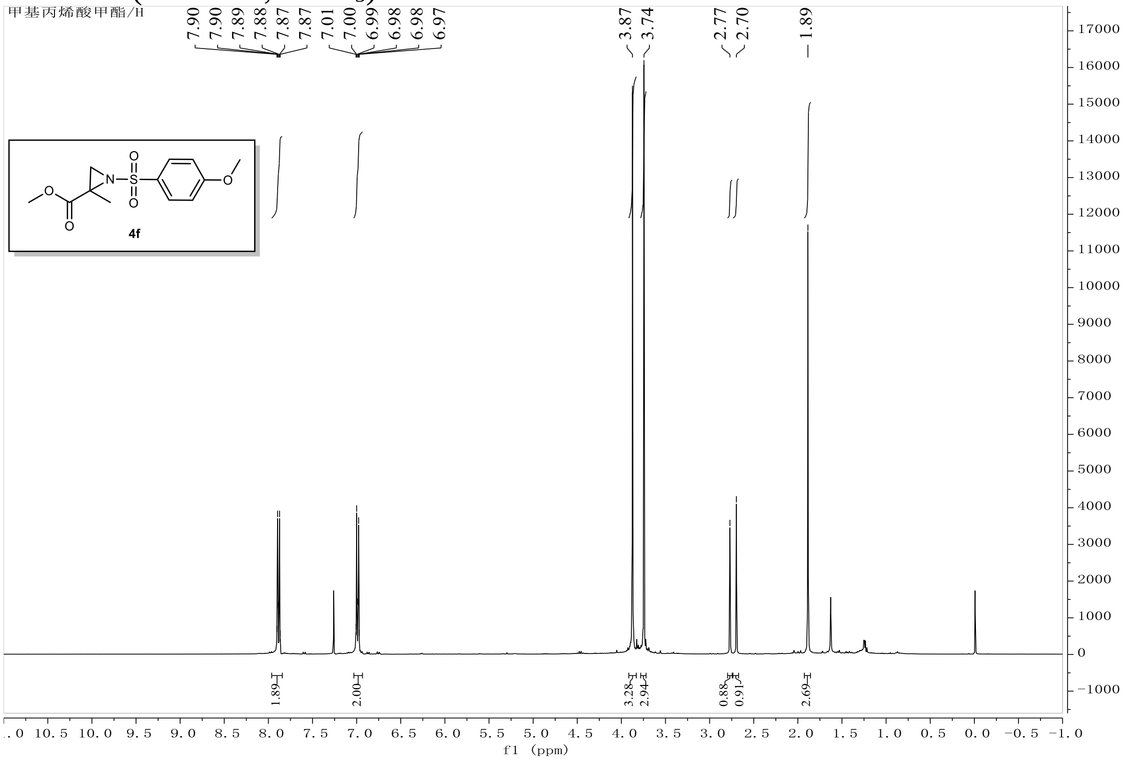

^{13}C NMR (101 MHz, $CDCl_3$):

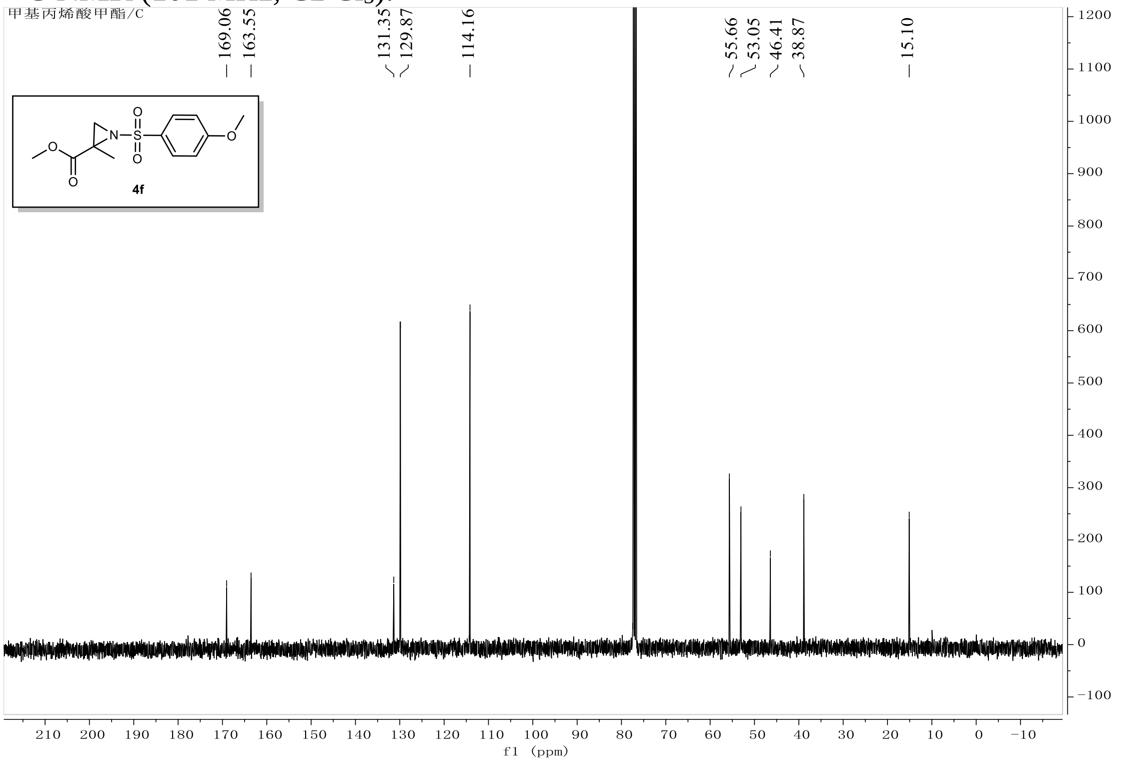

^{1}H NMR (400 MHz, $CDCl_3$):

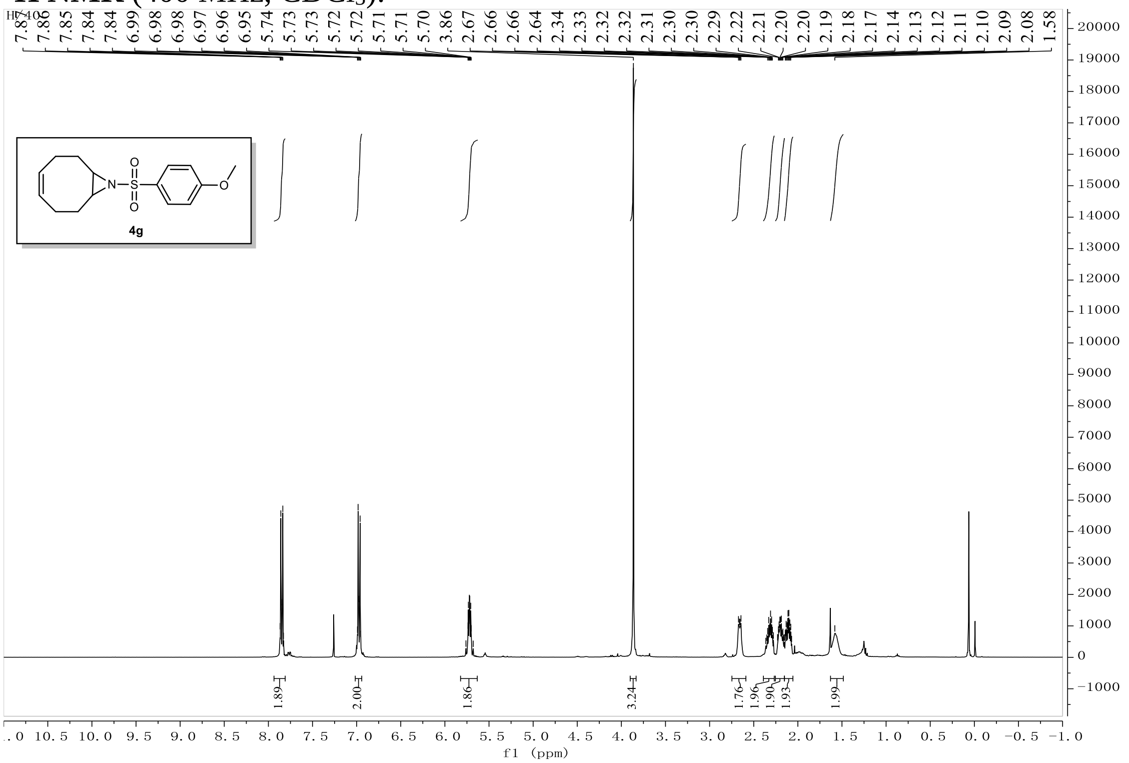

^{13}C NMR (101 MHz, $CDCl_3$):

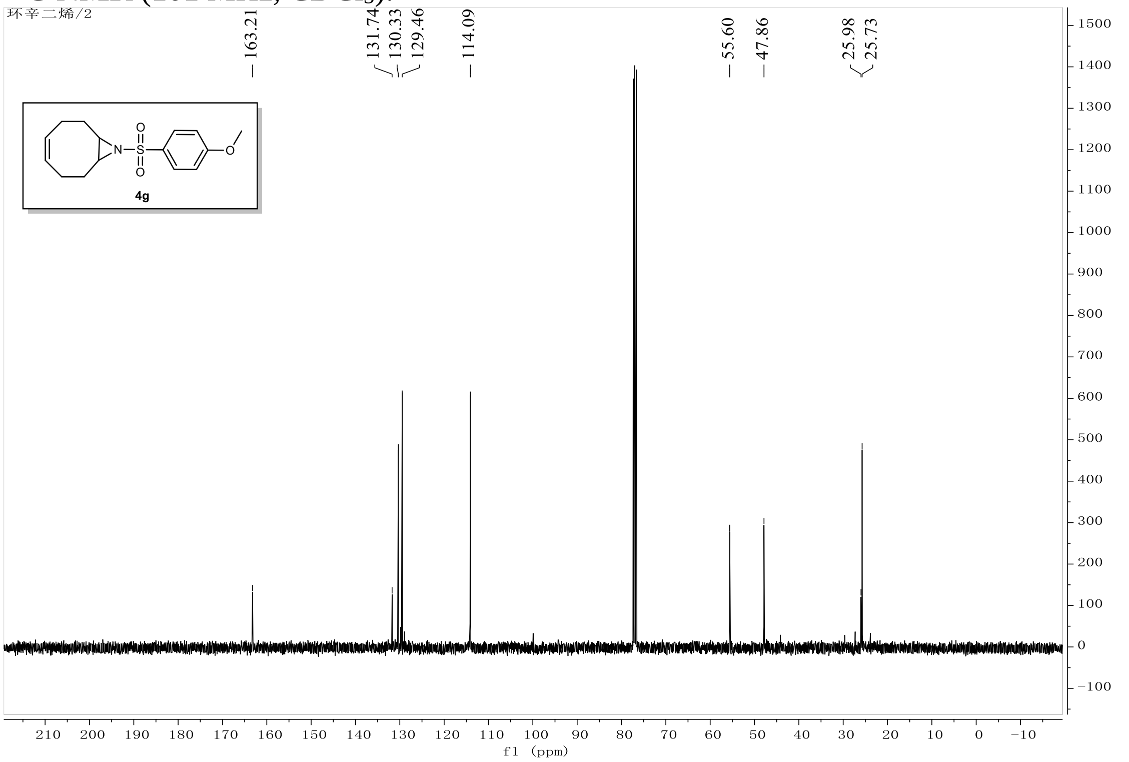

^{1}H NMR (400 MHz, $CDCl_3$):cis

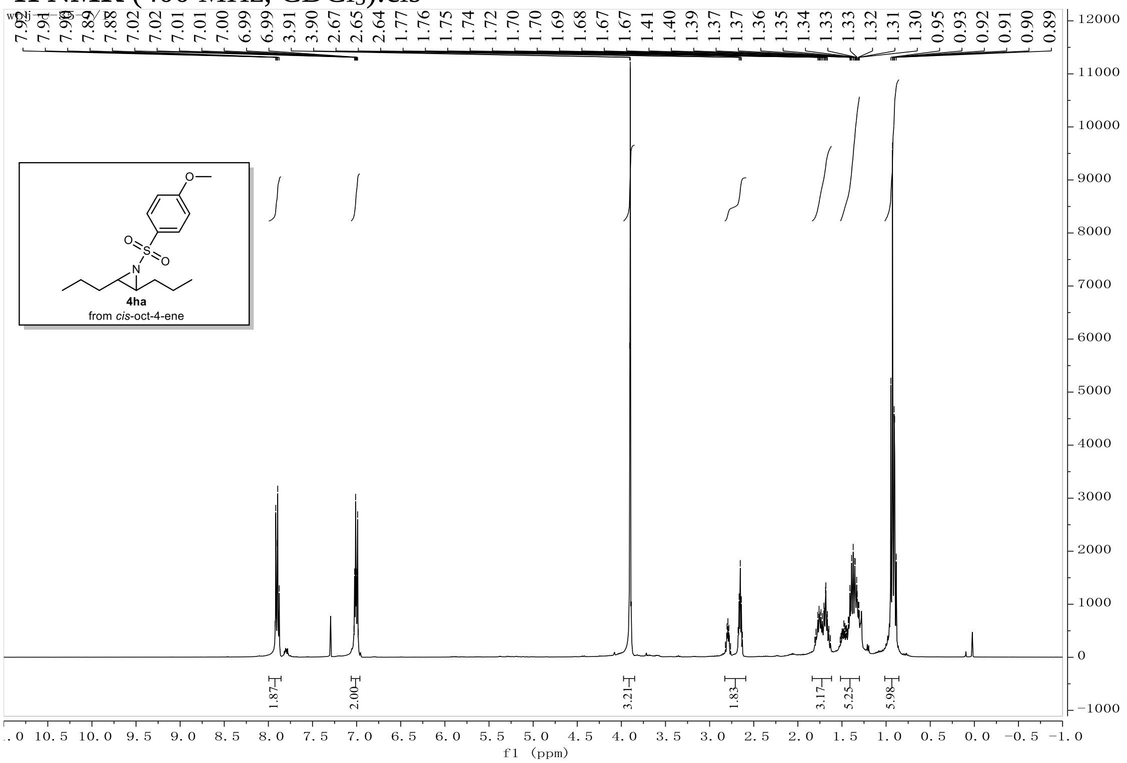

^{13}C NMR (101 MHz, $CDCl_3$):

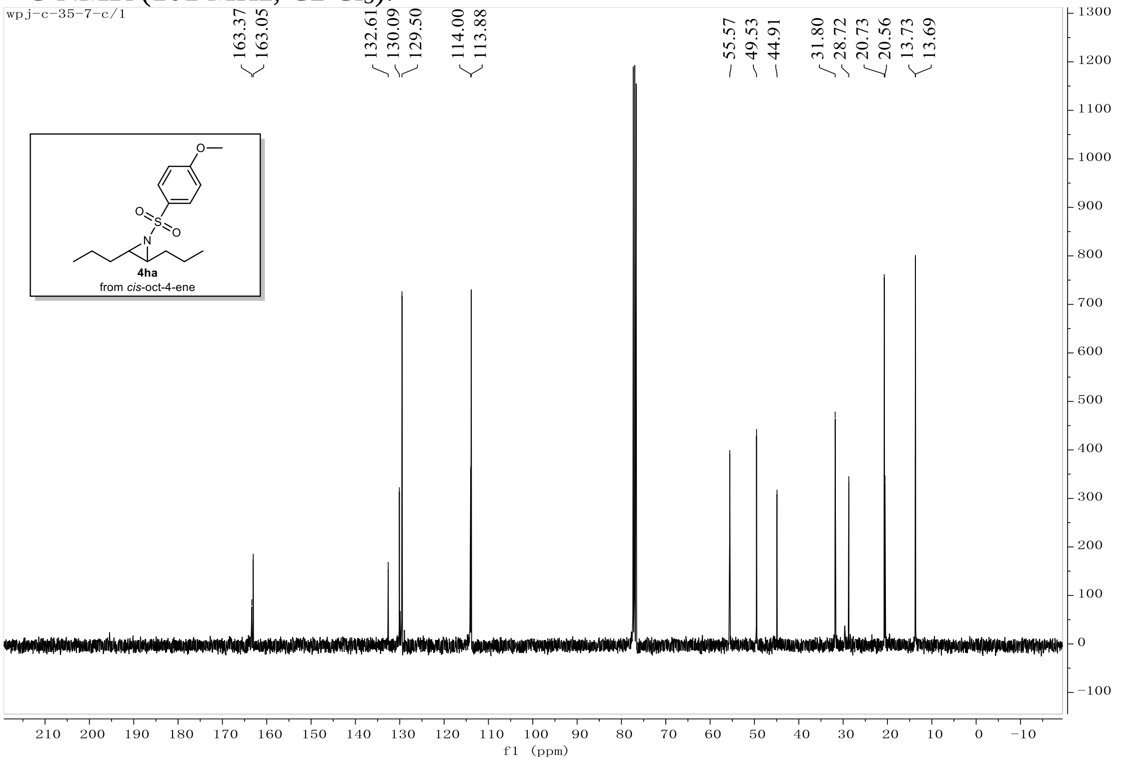

^{1}H NMR (400 MHz, $CDCl_3$):

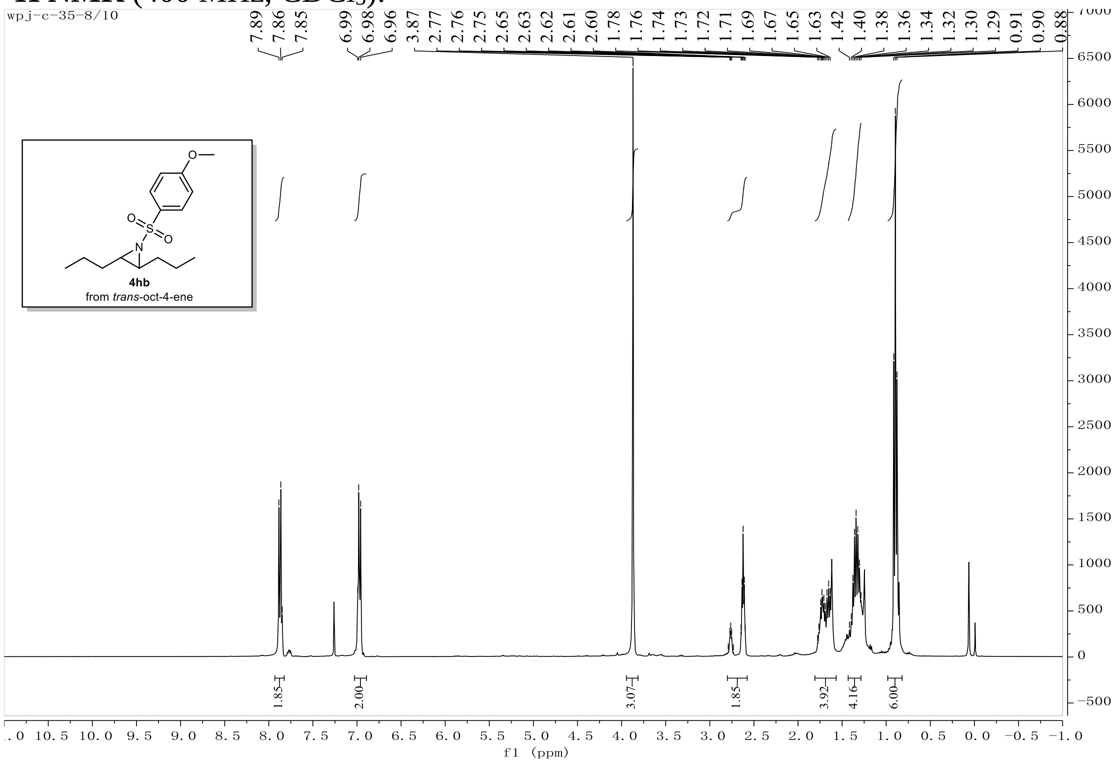

^{13}C NMR (101 MHz, $CDCl_3$):

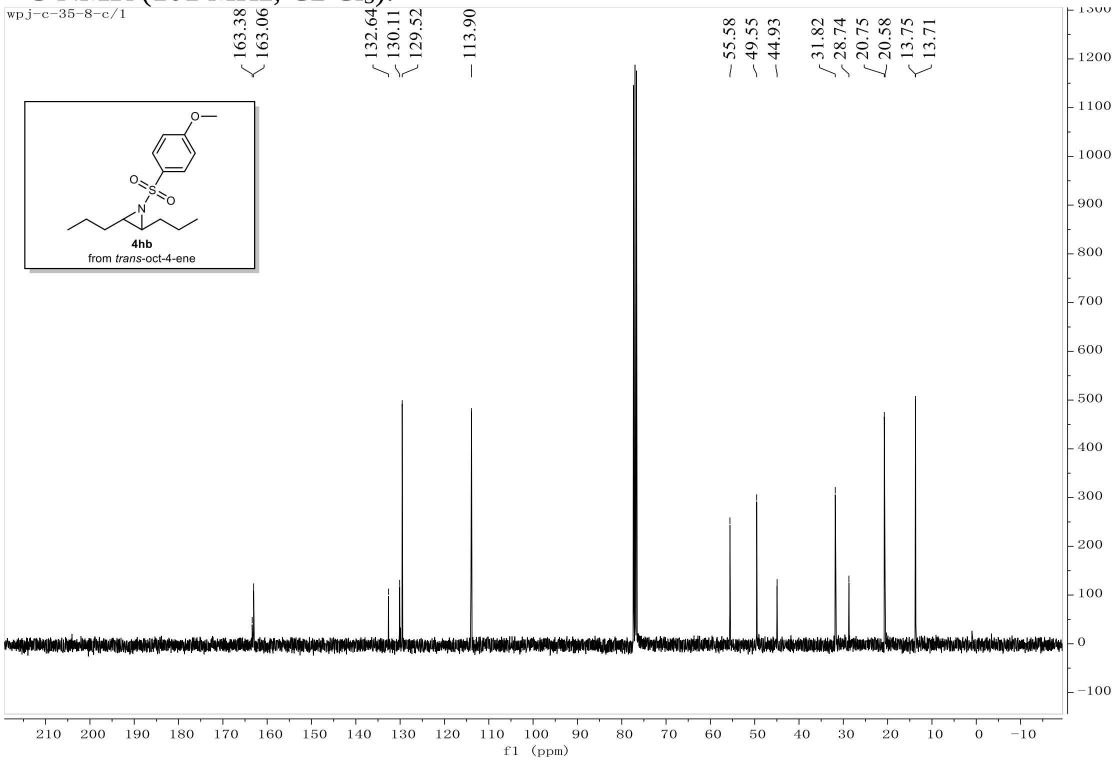

^{1}H NMR (400 MHz, $CDCl_3$):

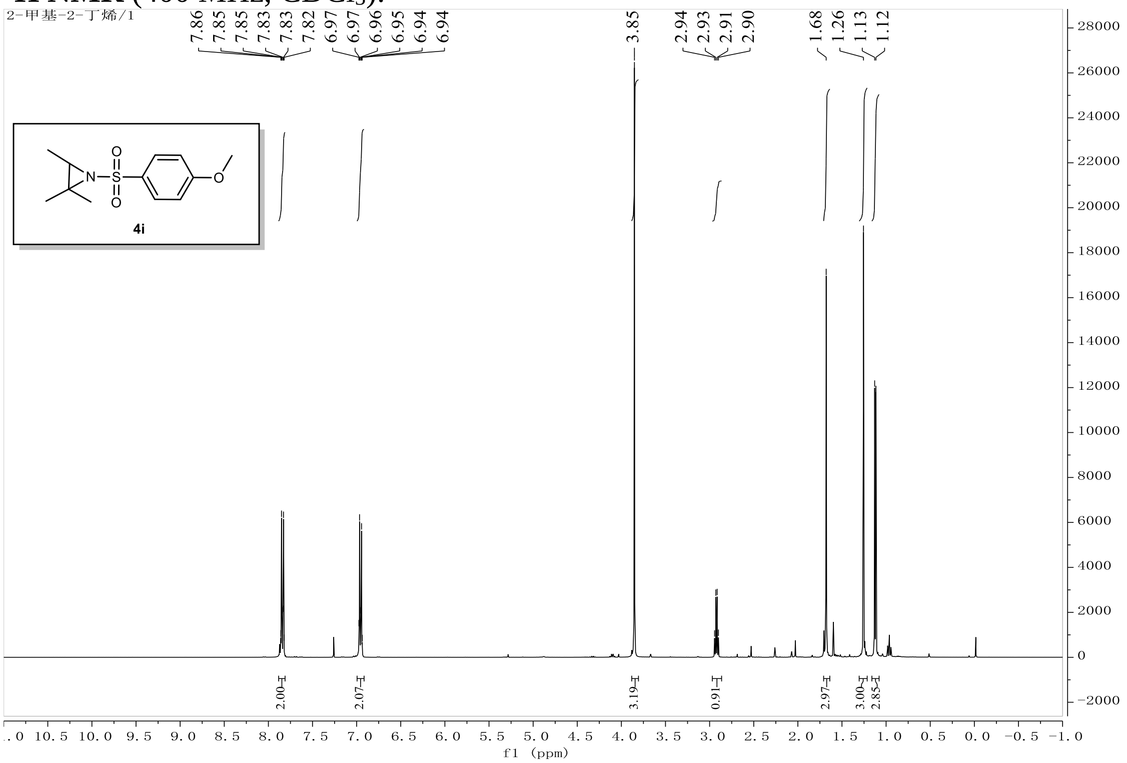

^{13}C NMR (101 MHz, $CDCl_3$):

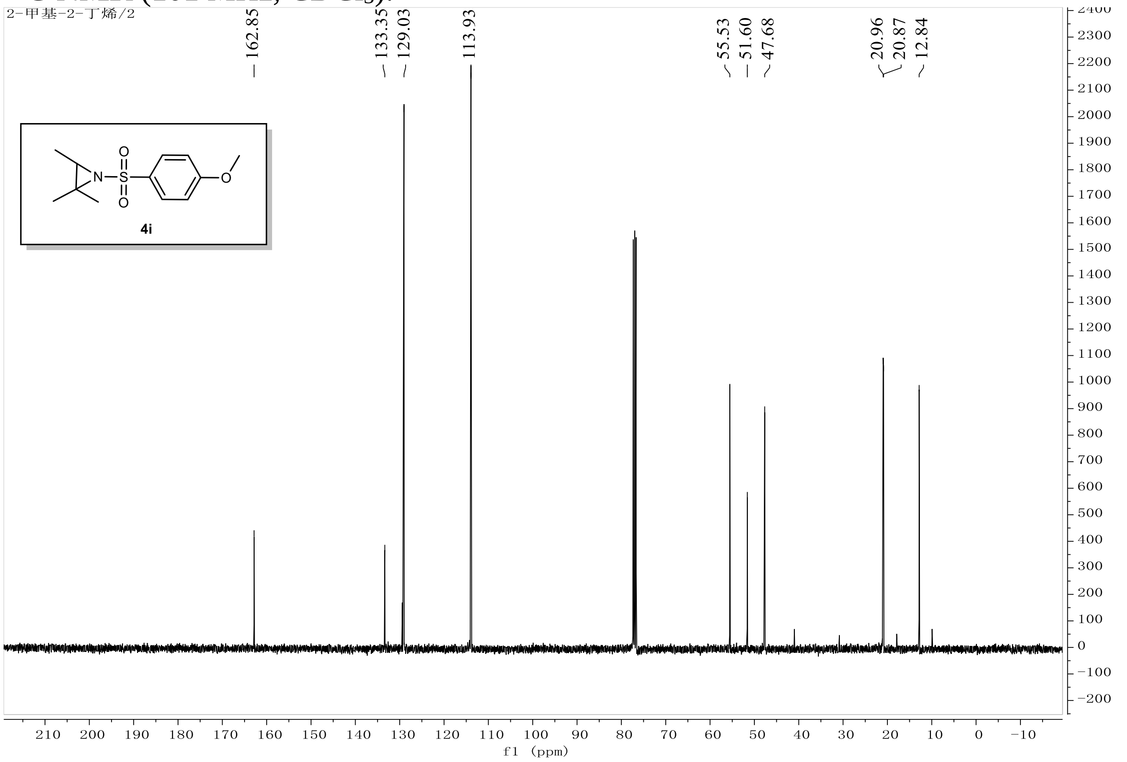

^{1}H NMR (400 MHz, $CDCl_3$):

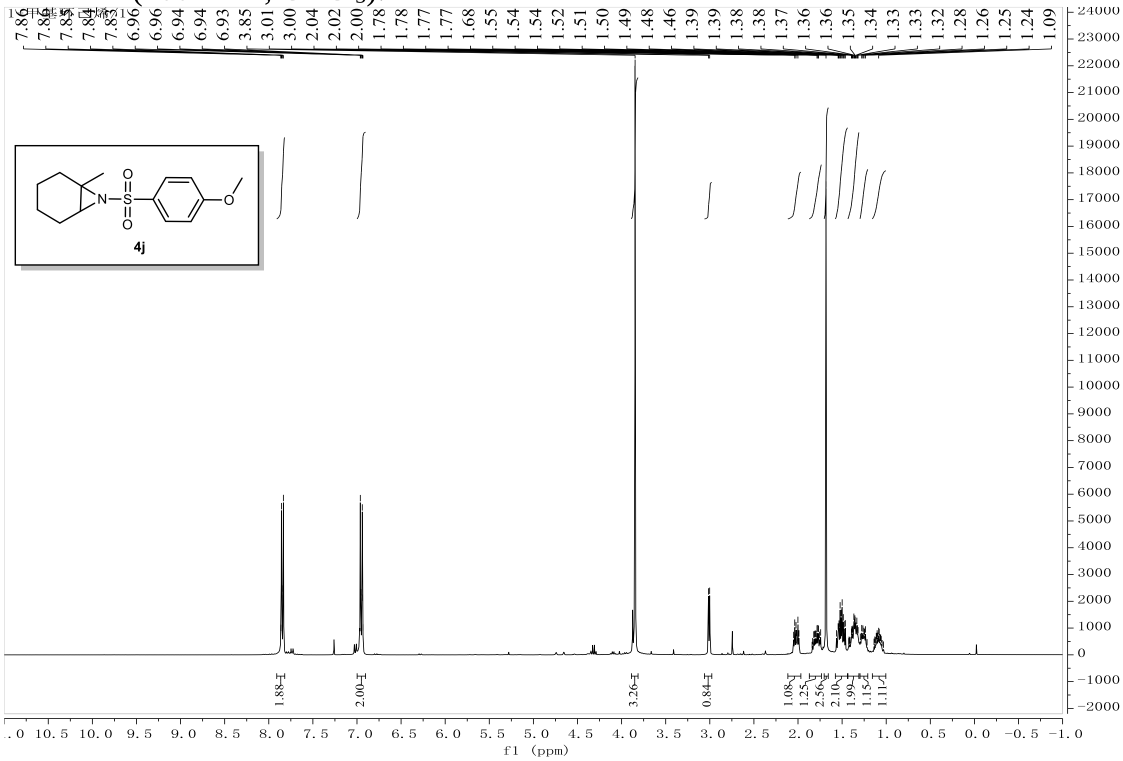

^{13}C NMR (101 MHz, $CDCl_3$):

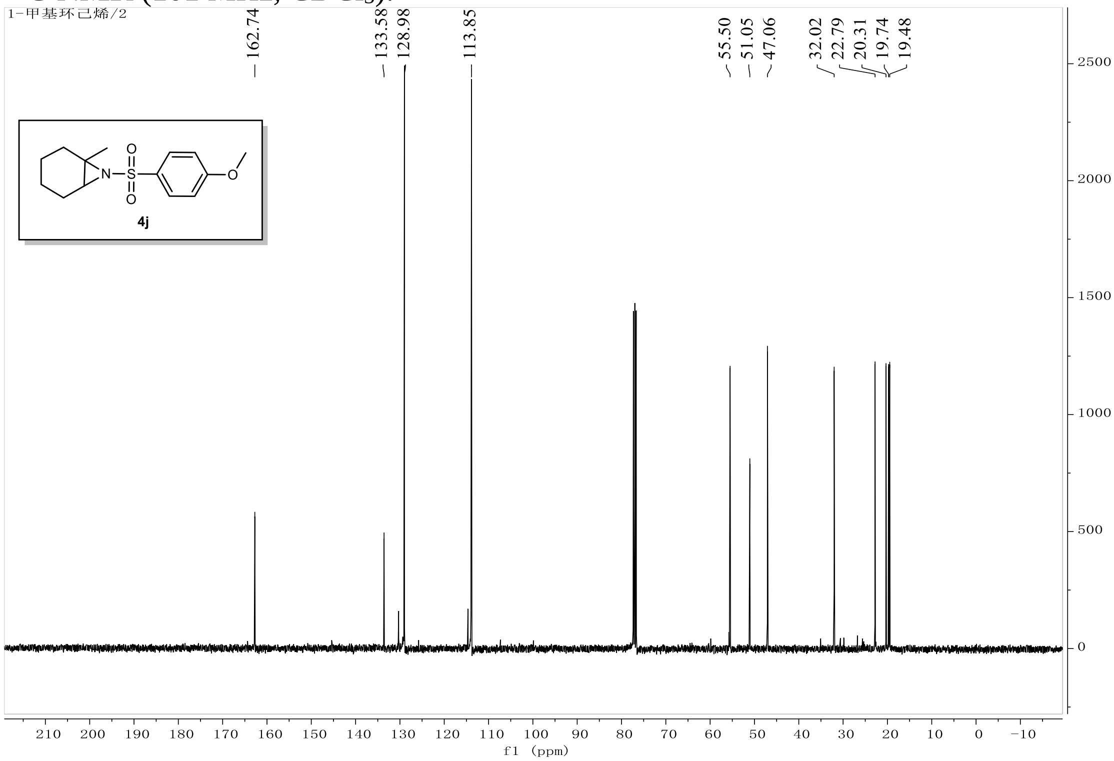

^{1}H NMR (400 MHz, $CDCl_3$):

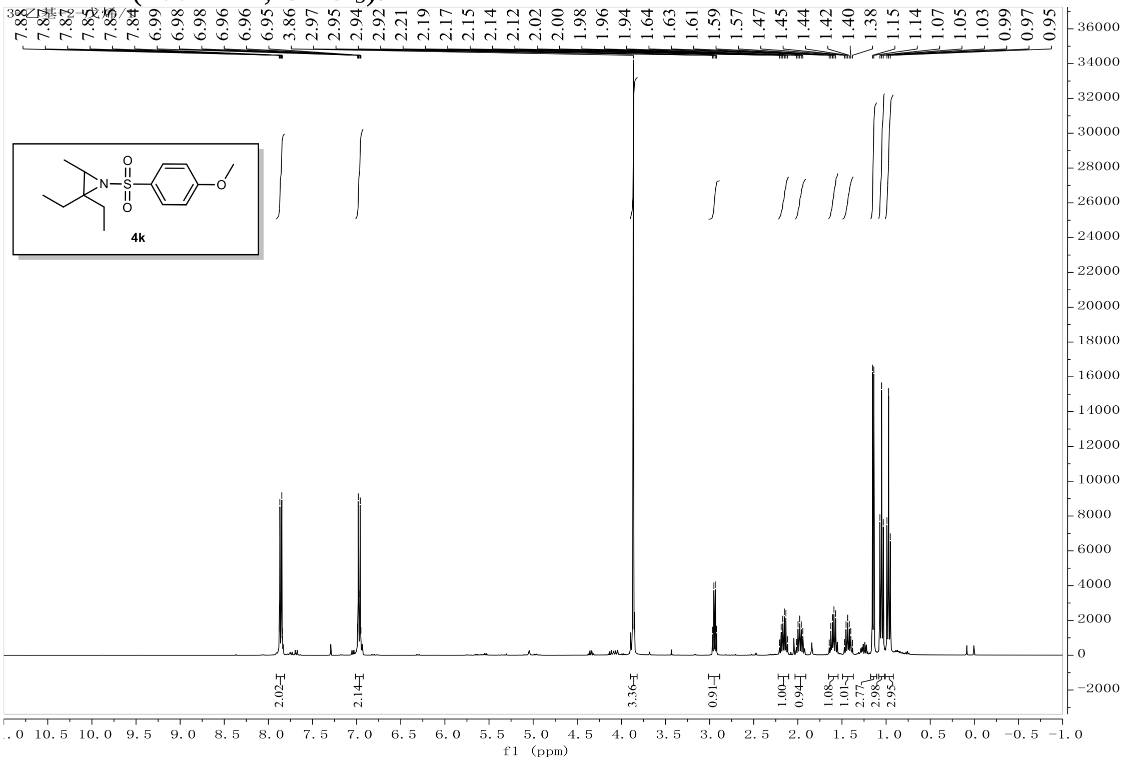

^{13}C NMR (101 MHz, $CDCl_3$):

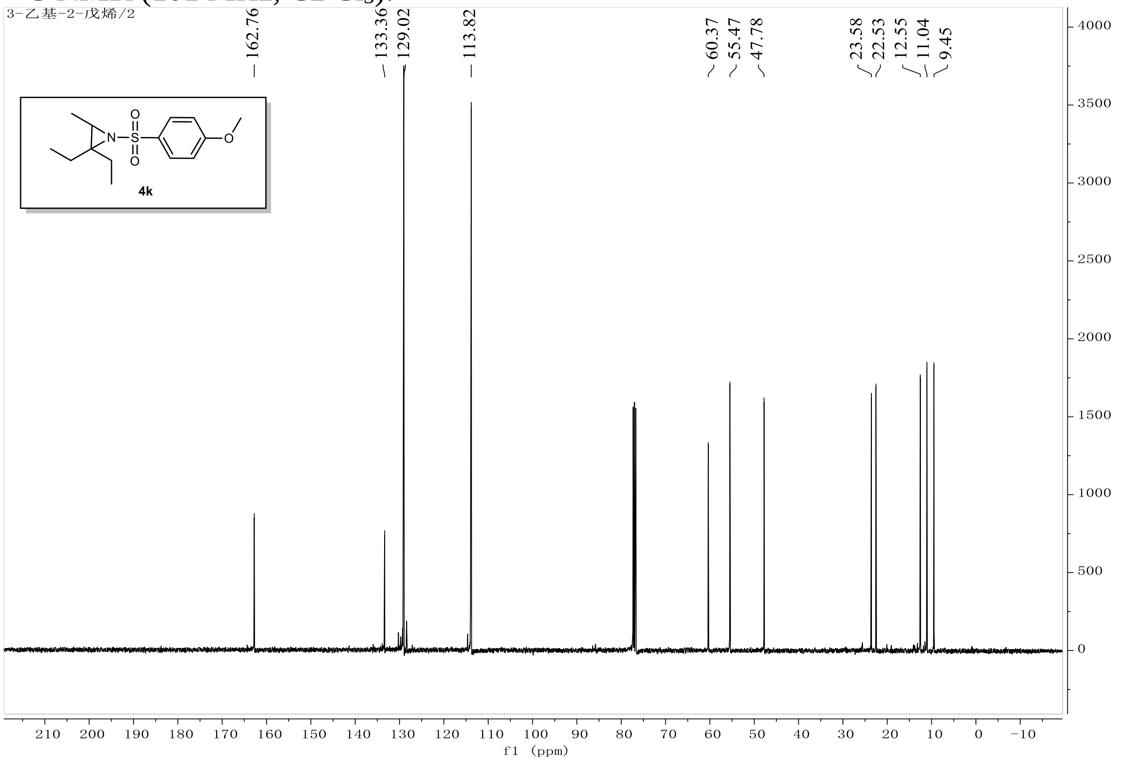

^{1}H NMR (400 MHz, $CDCl_3$):

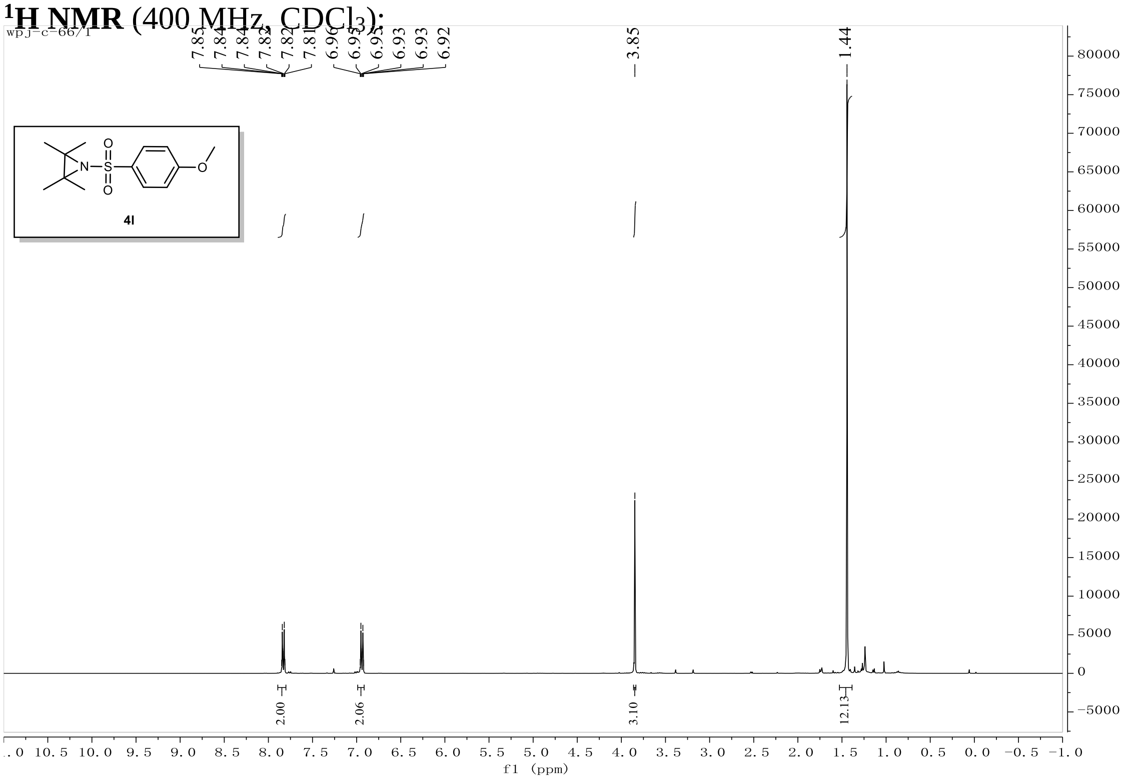

^{13}C NMR (101 MHz, $CDCl_3$):

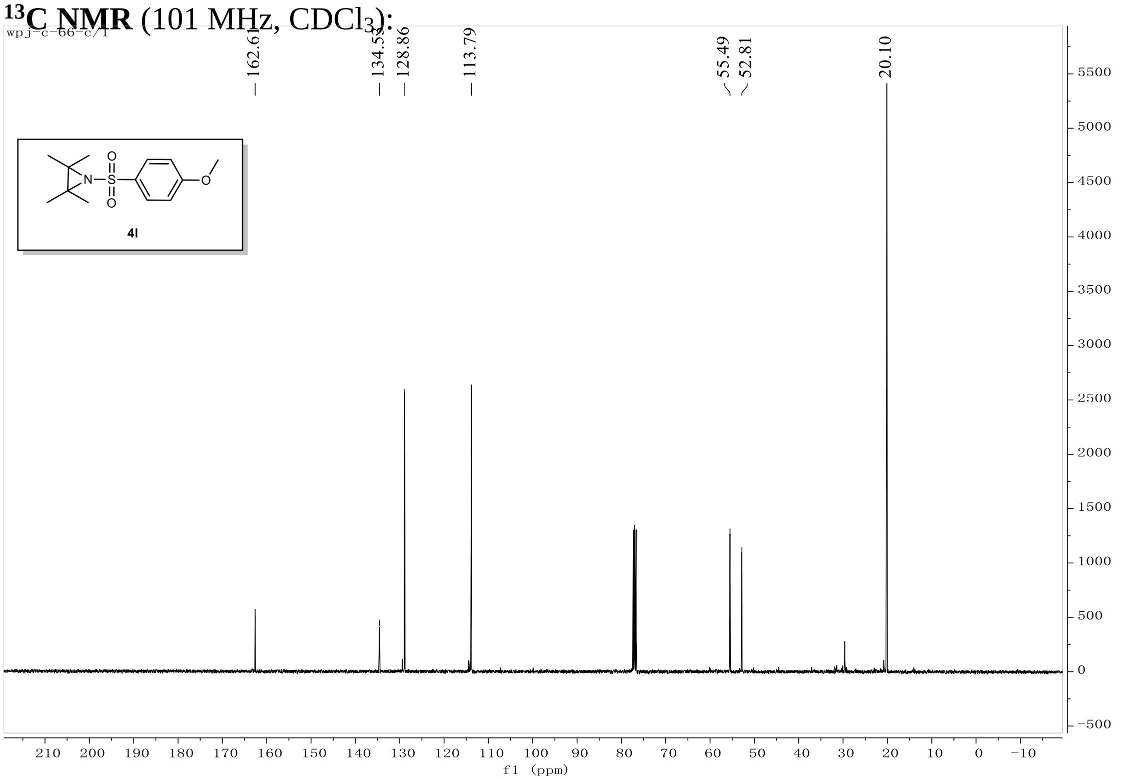

^{1}H NMR (400 MHz, $CDCl_3$):

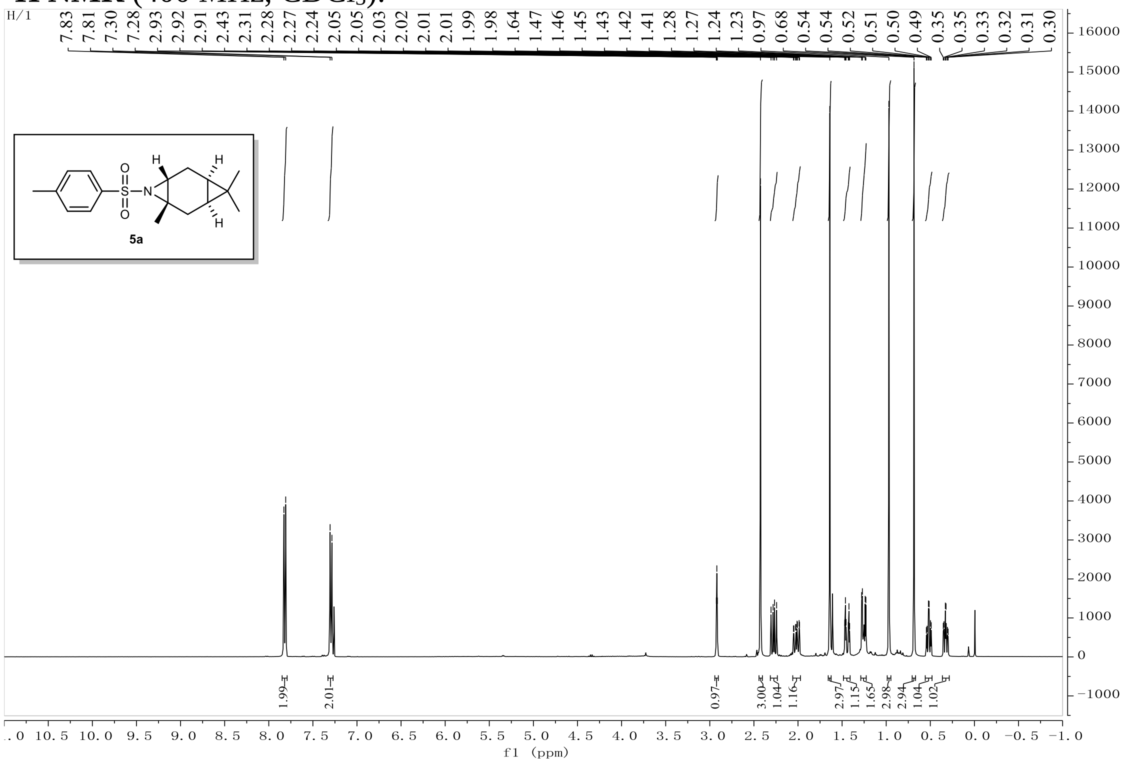

^{13}C NMR (101 MHz, $CDCl_3$):

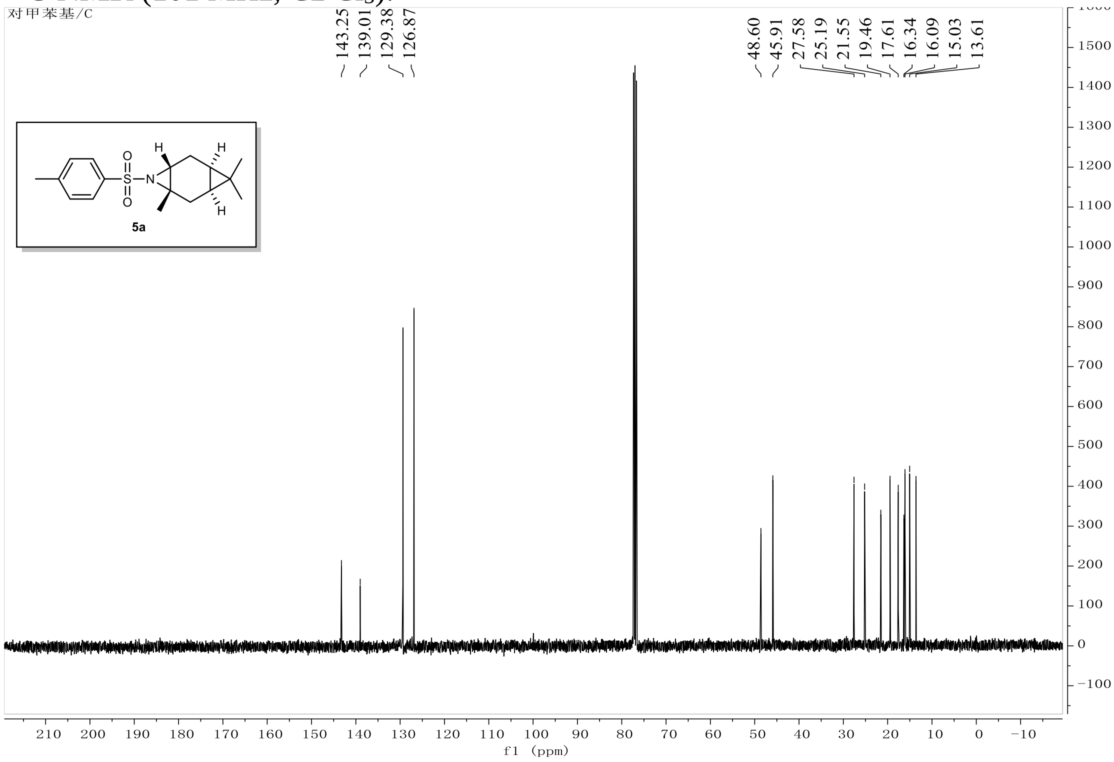

^{1}H NMR (400 MHz, $CDCl_3$):

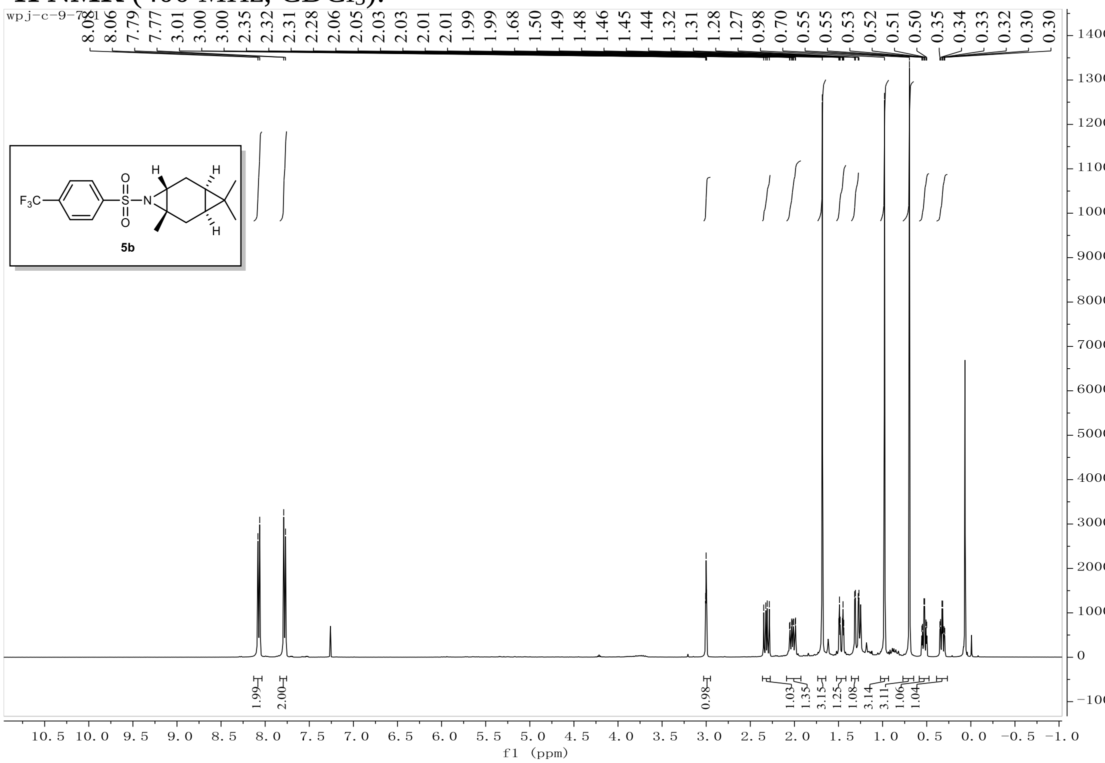

^{13}C NMR (101 MHz, $CDCl_3$):

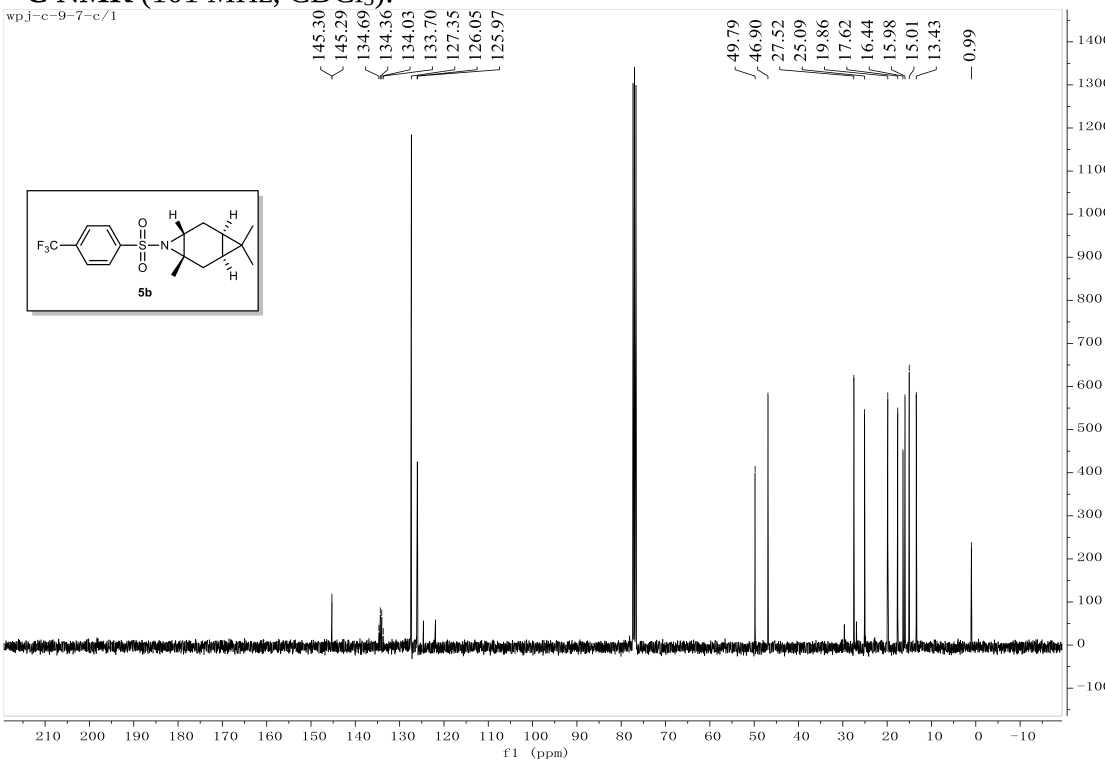

^{19}F NMR (377 MHz, $CDCl_3$):

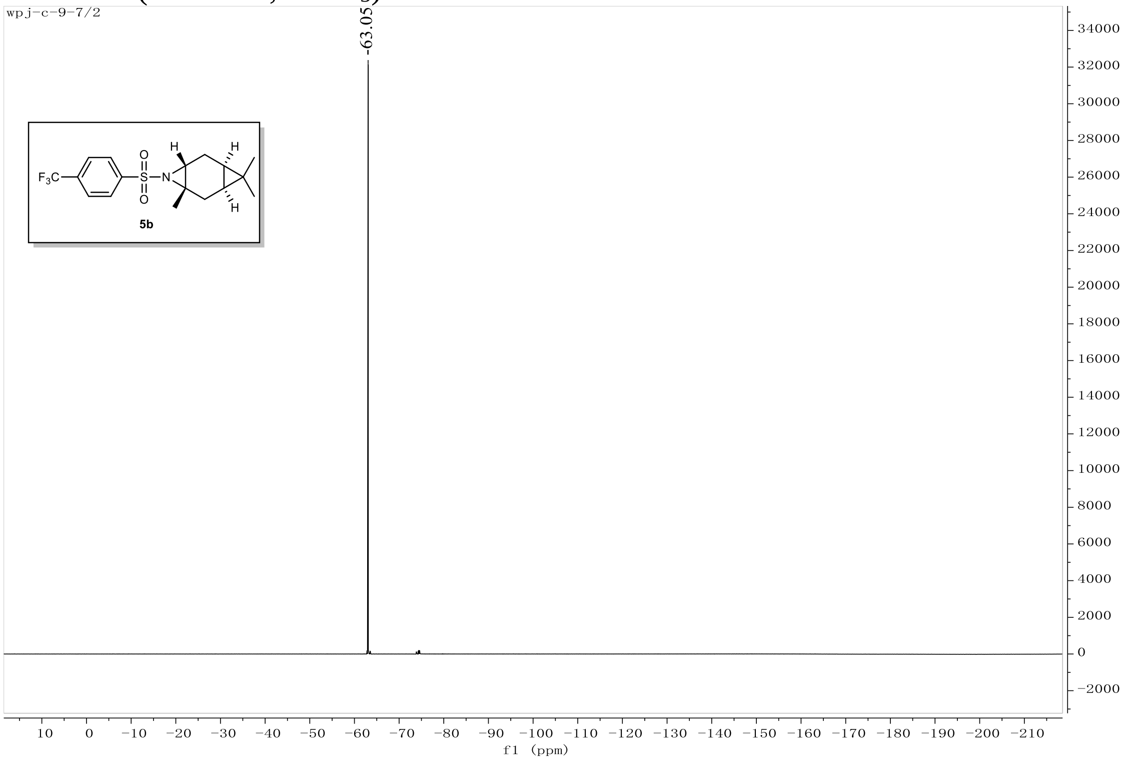

^{1}H NMR (400 MHz, $CDCl_3$):

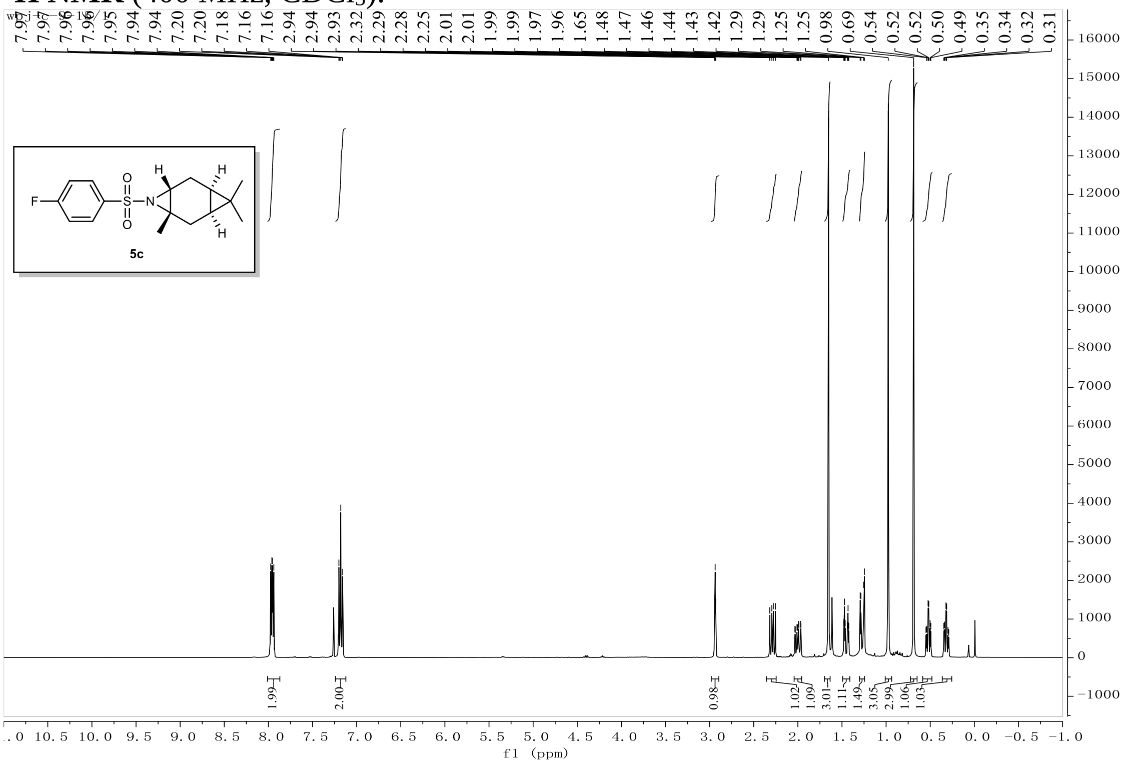

^{13}C NMR (101 MHz, $CDCl_3$):

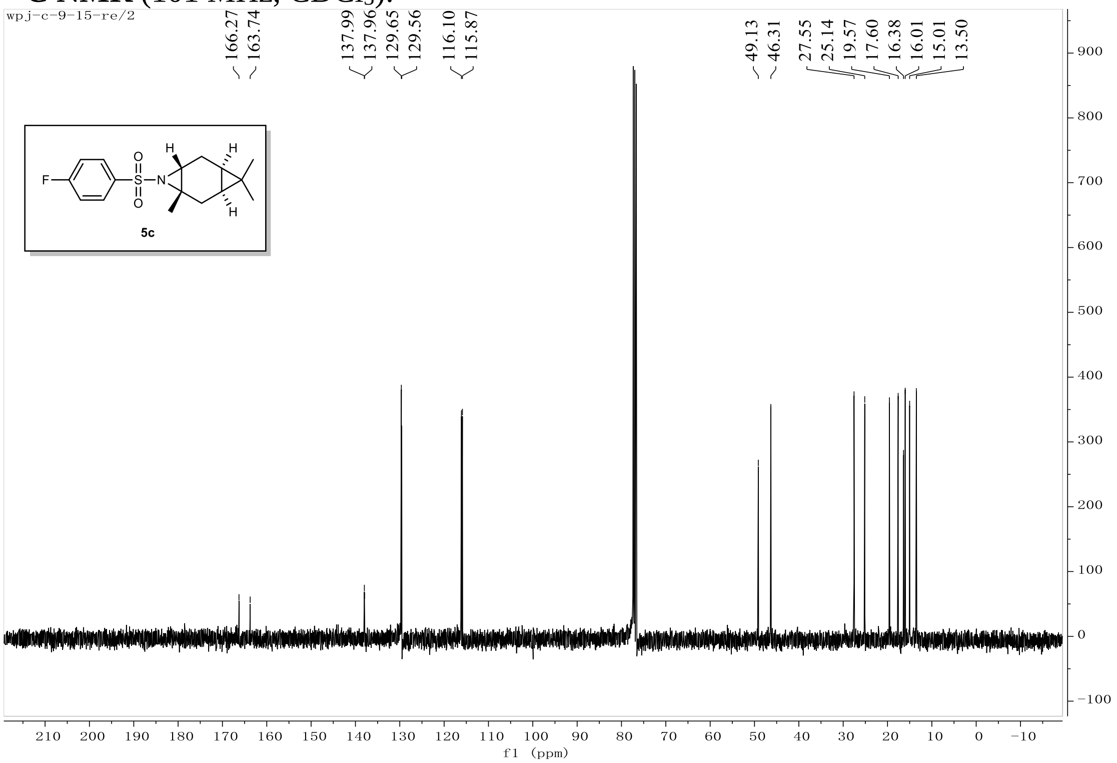

^{19}F NMR (377 MHz, $CDCl_3$):

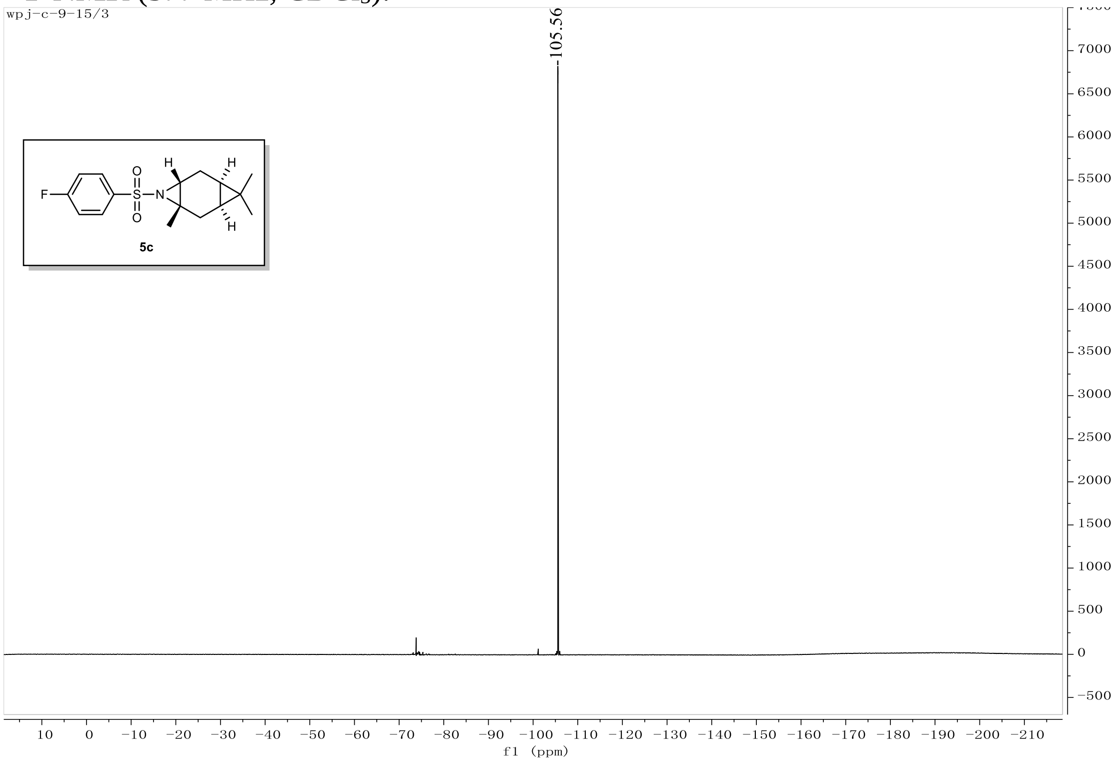

^{1}H NMR (400 MHz, $CDCl_3$):

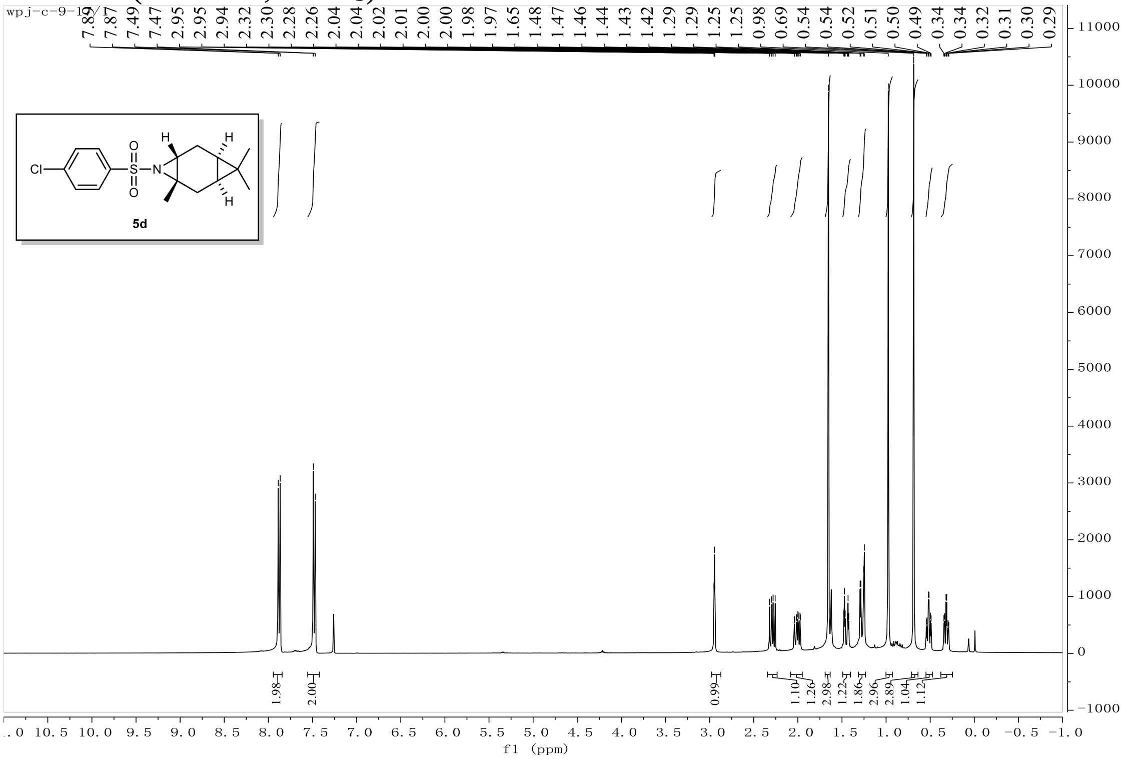

^{13}C NMR (101 MHz, $CDCl_3$):

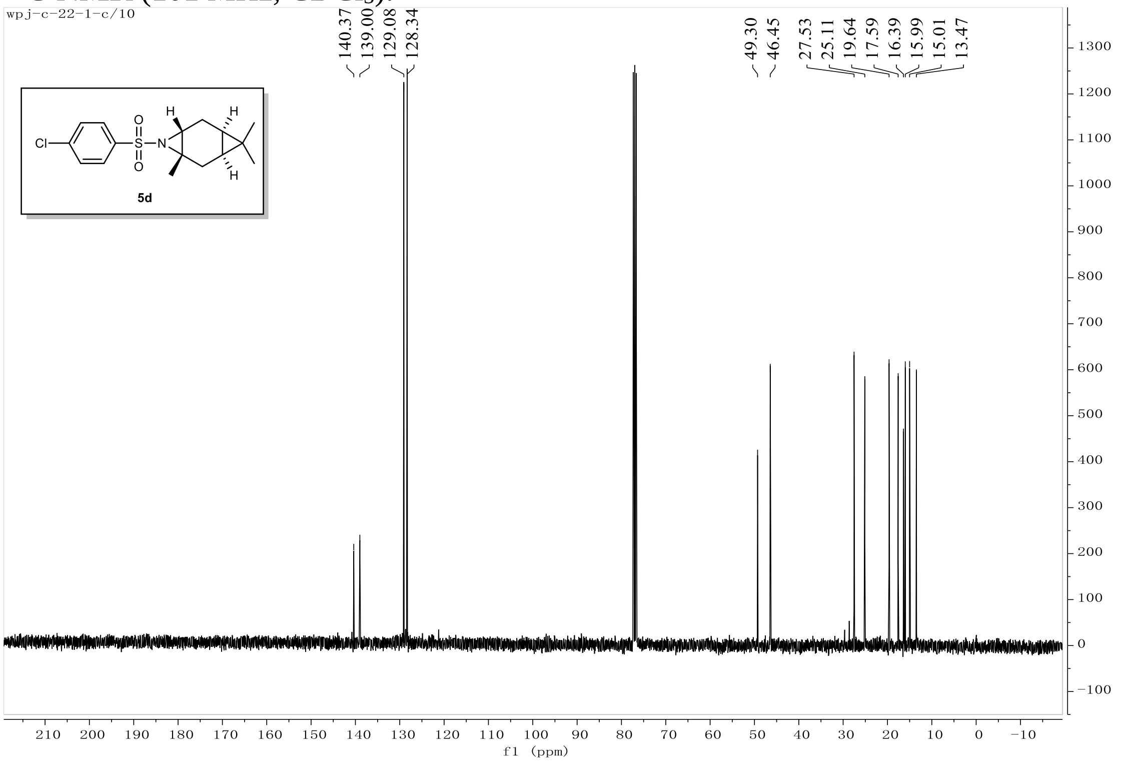

^{1}H NMR (400 MHz, $CDCl_3$):

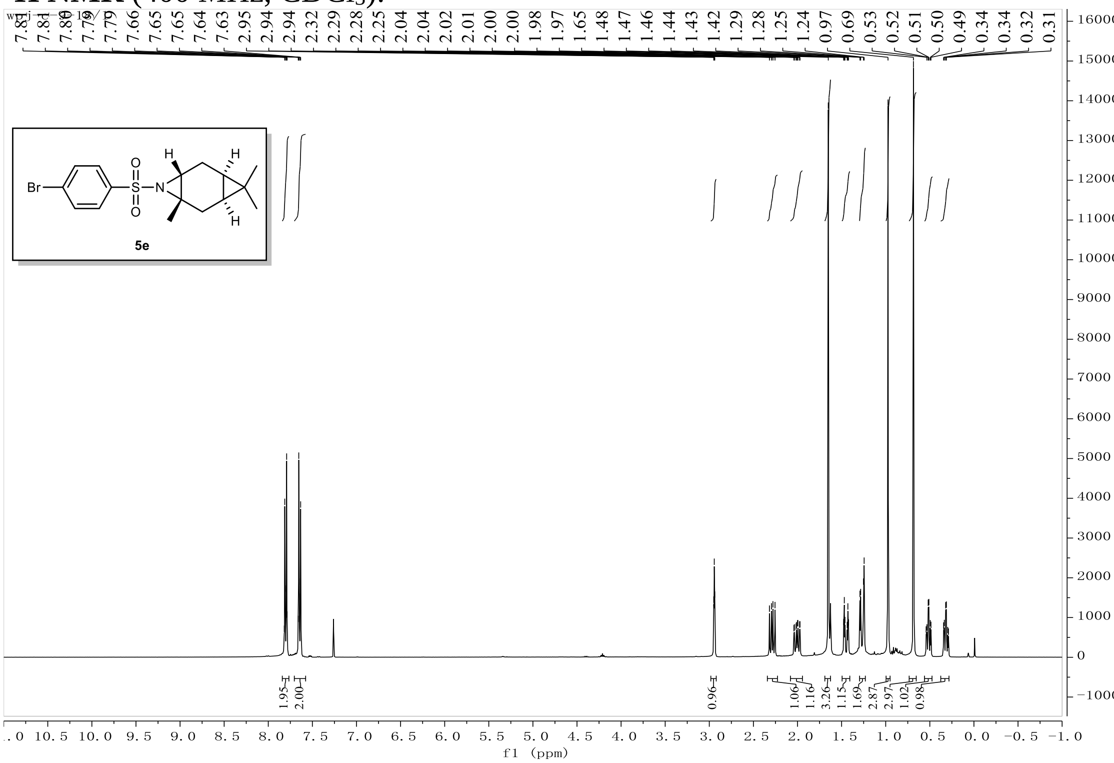

^{13}C NMR (101 MHz, $CDCl_3$):

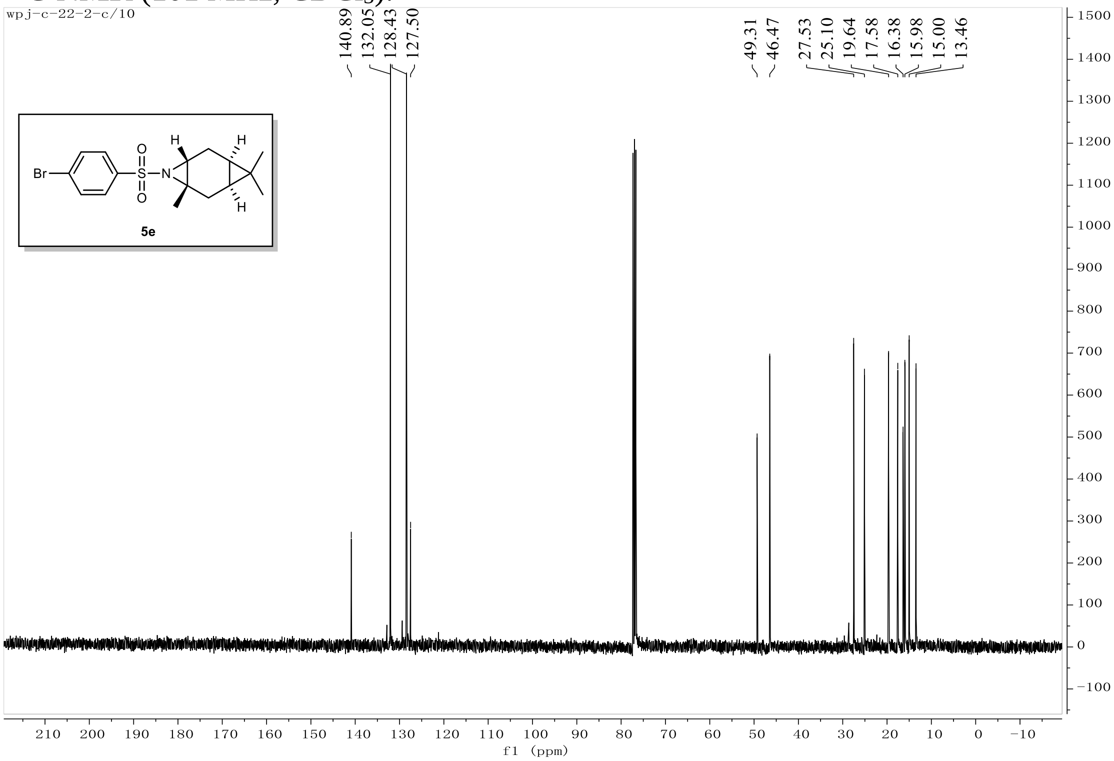

^{1}H NMR (400 MHz, $CDCl_3$):

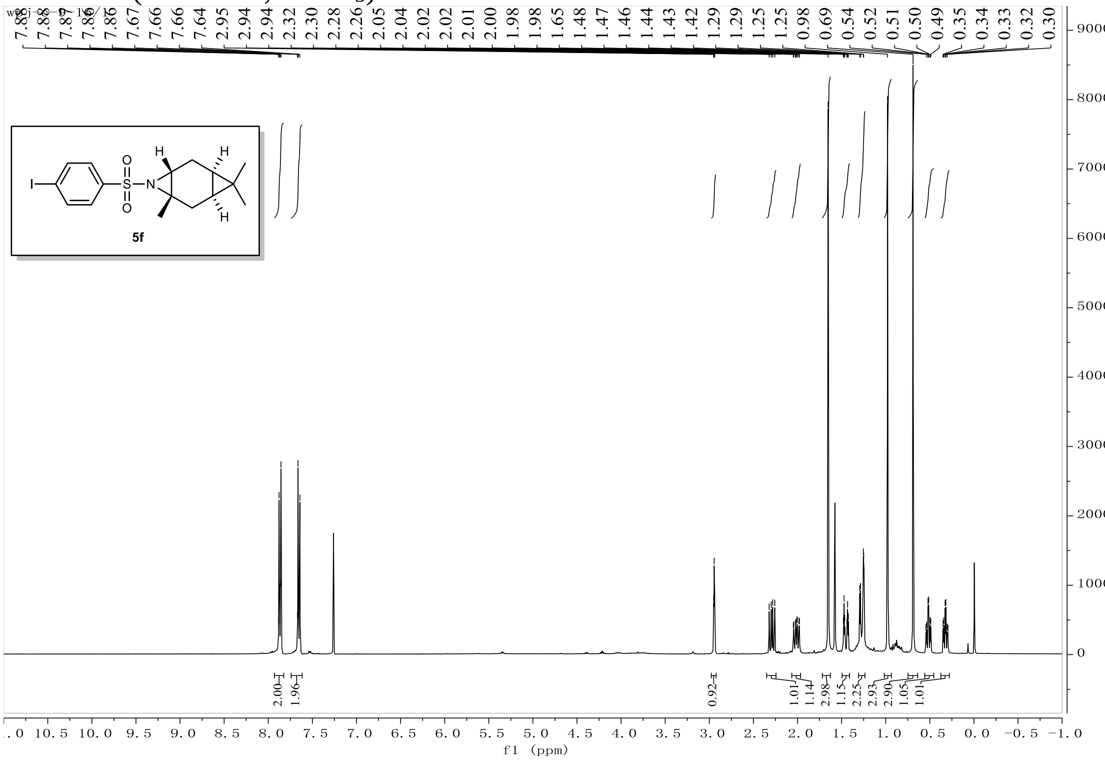

^{13}C NMR (101 MHz, $CDCl_3$):

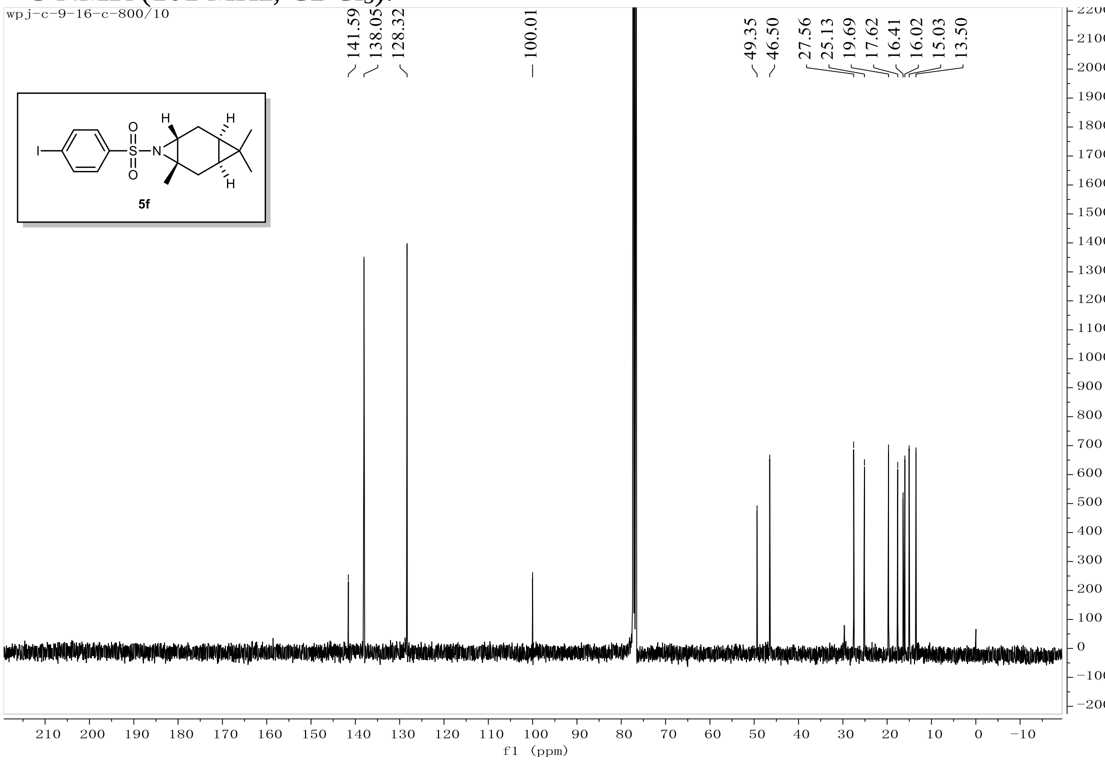

^{1}H NMR (400 MHz, $CDCl_3$):

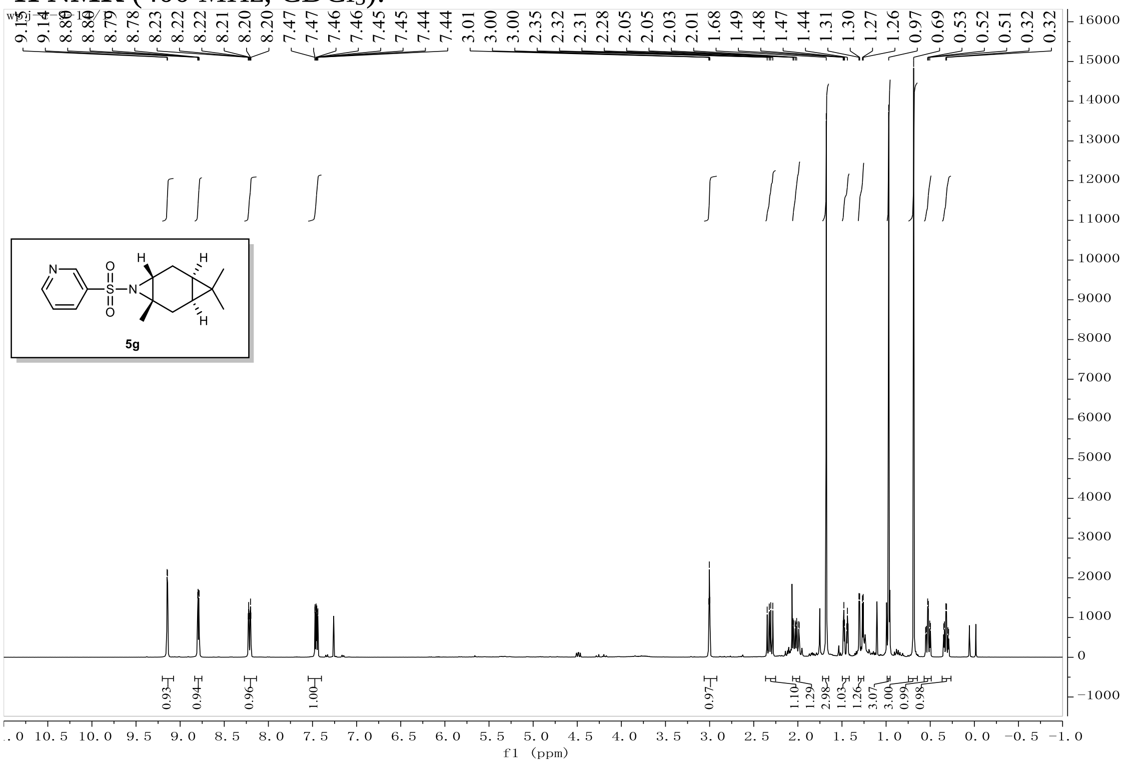

^{13}C NMR (101 MHz, $CDCl_3$):

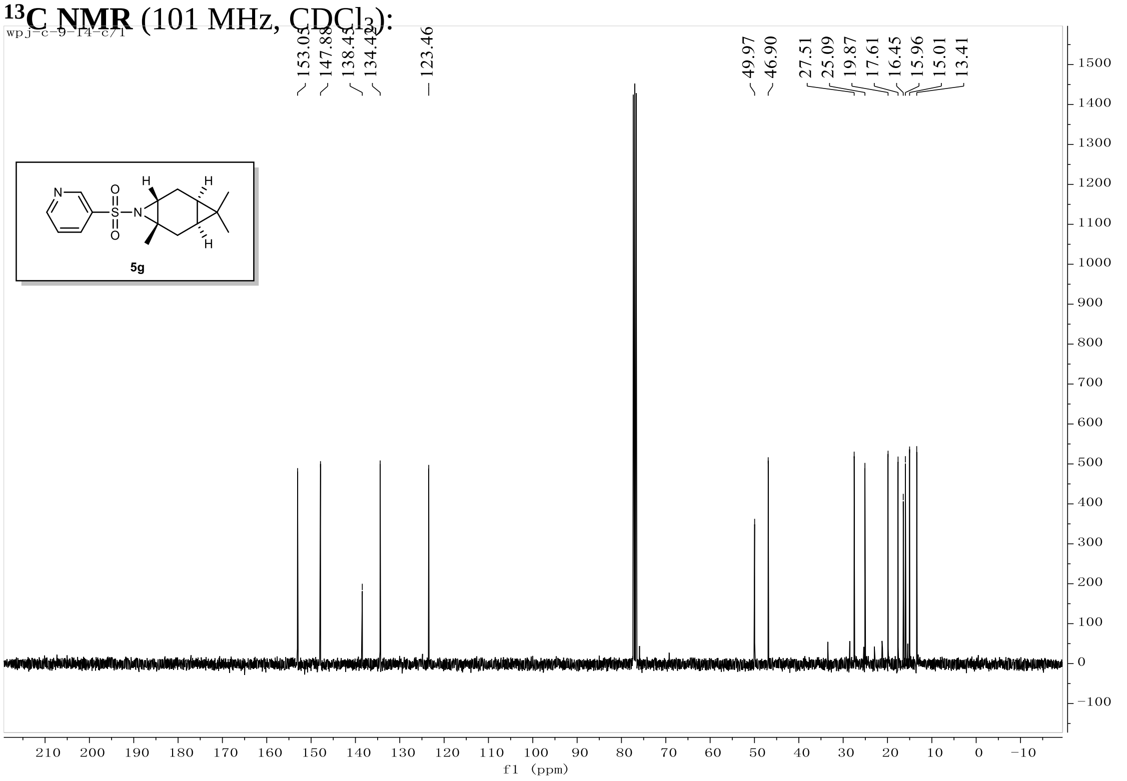

^{1}H NMR (400 MHz, $CDCl_3$):

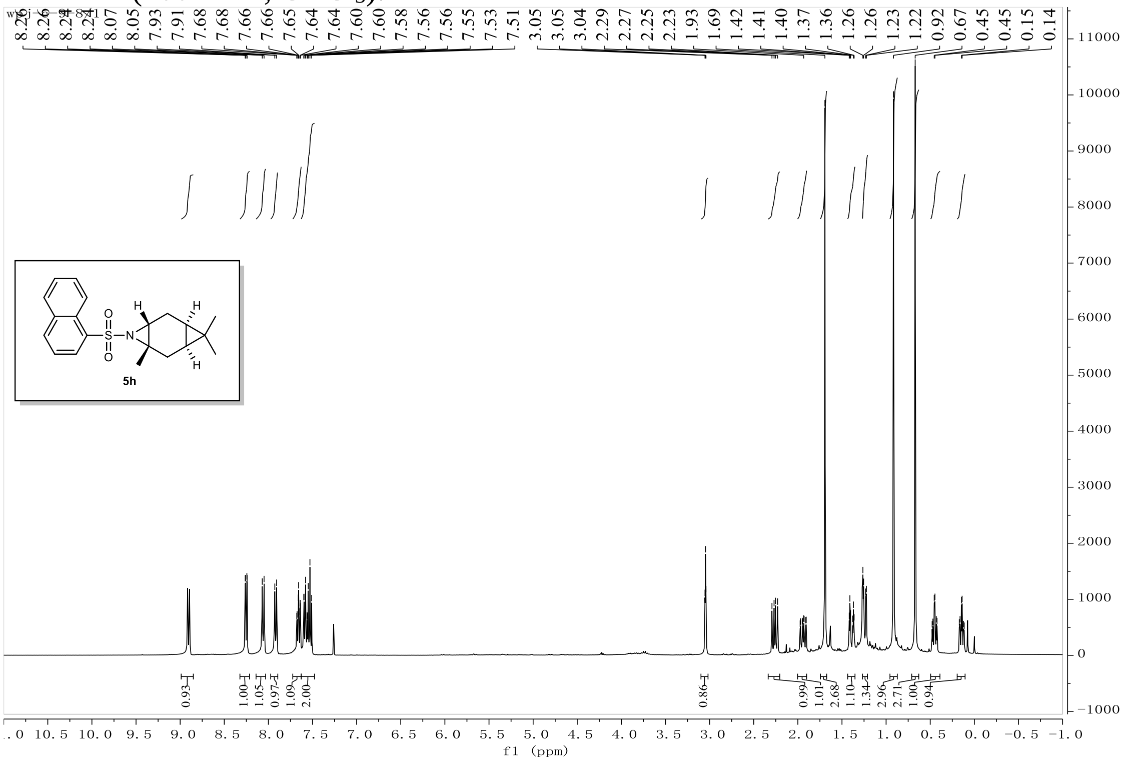

^{13}C NMR (101 MHz, $CDCl_3$):

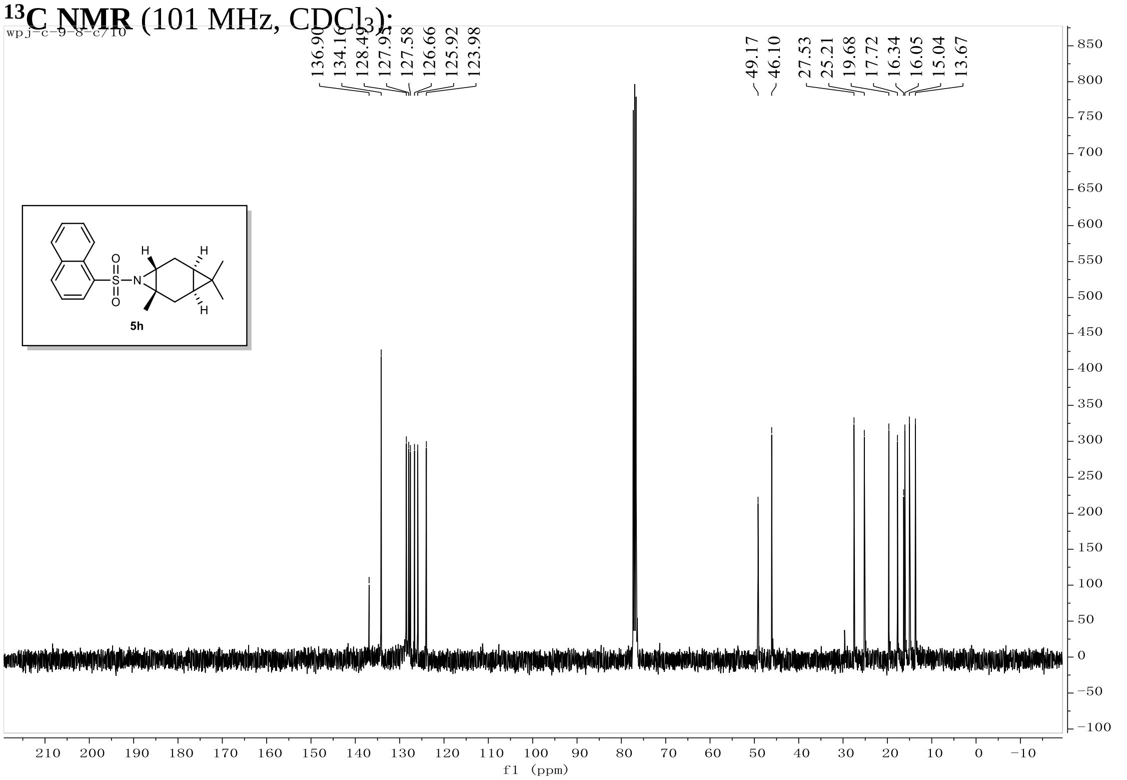

^{1}H NMR (400 MHz, $CDCl_3$):

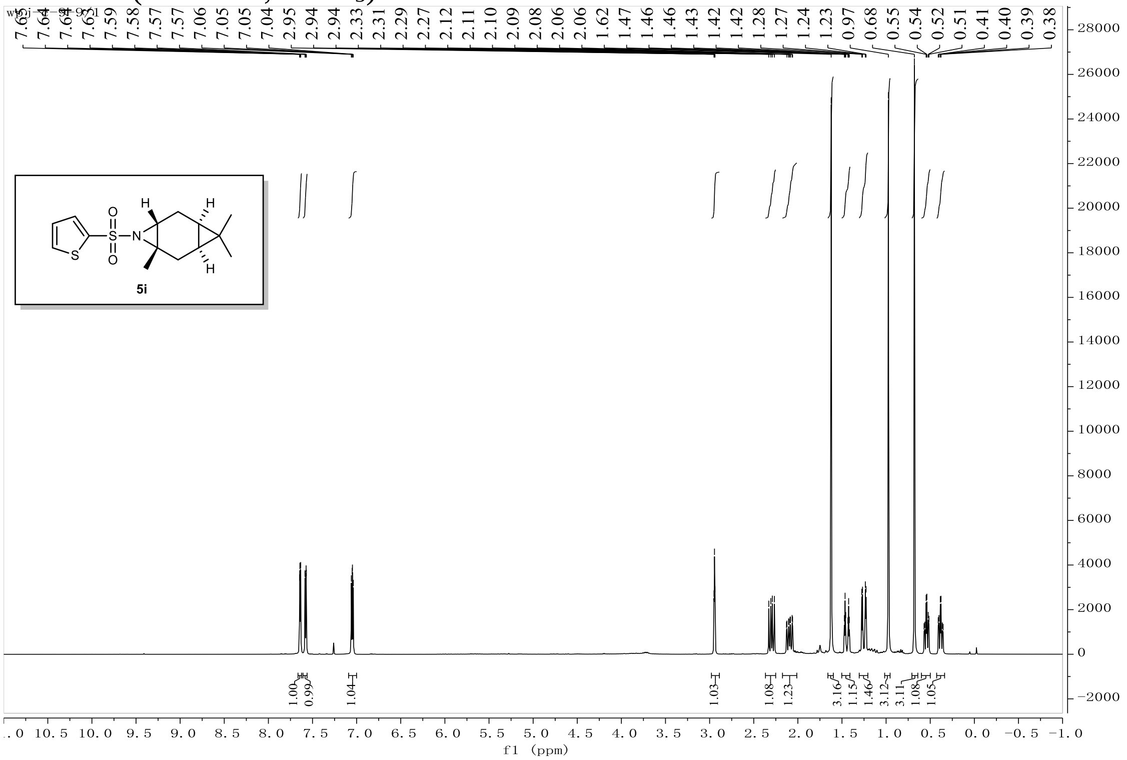

^{13}C NMR (101 MHz, $CDCl_3$):

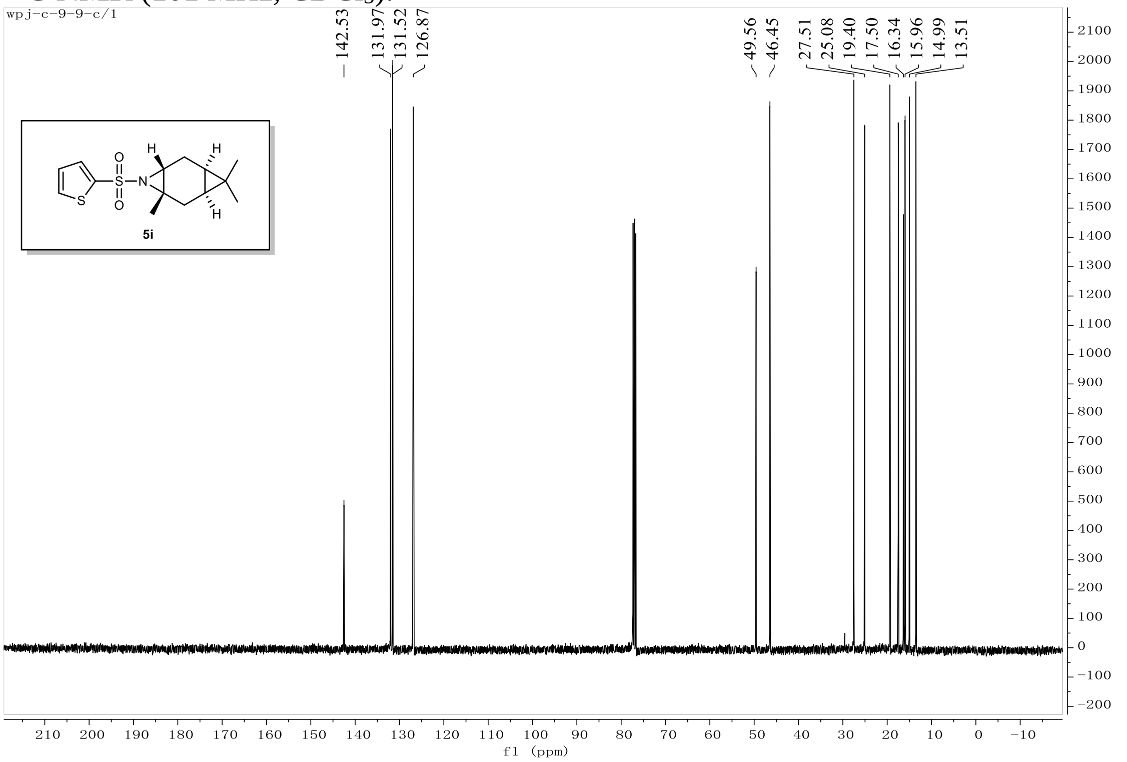

^{1}H NMR (400 MHz, $CDCl_3$):

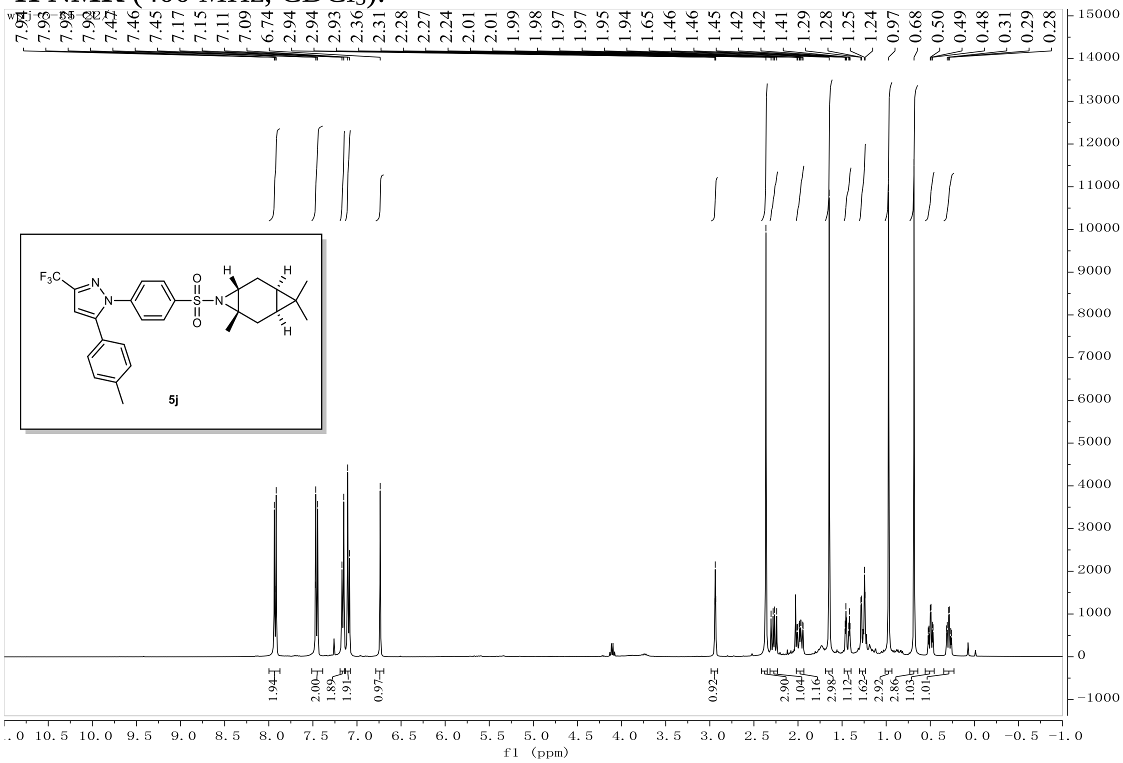

^{13}C NMR (101 MHz, $CDCl_3$):

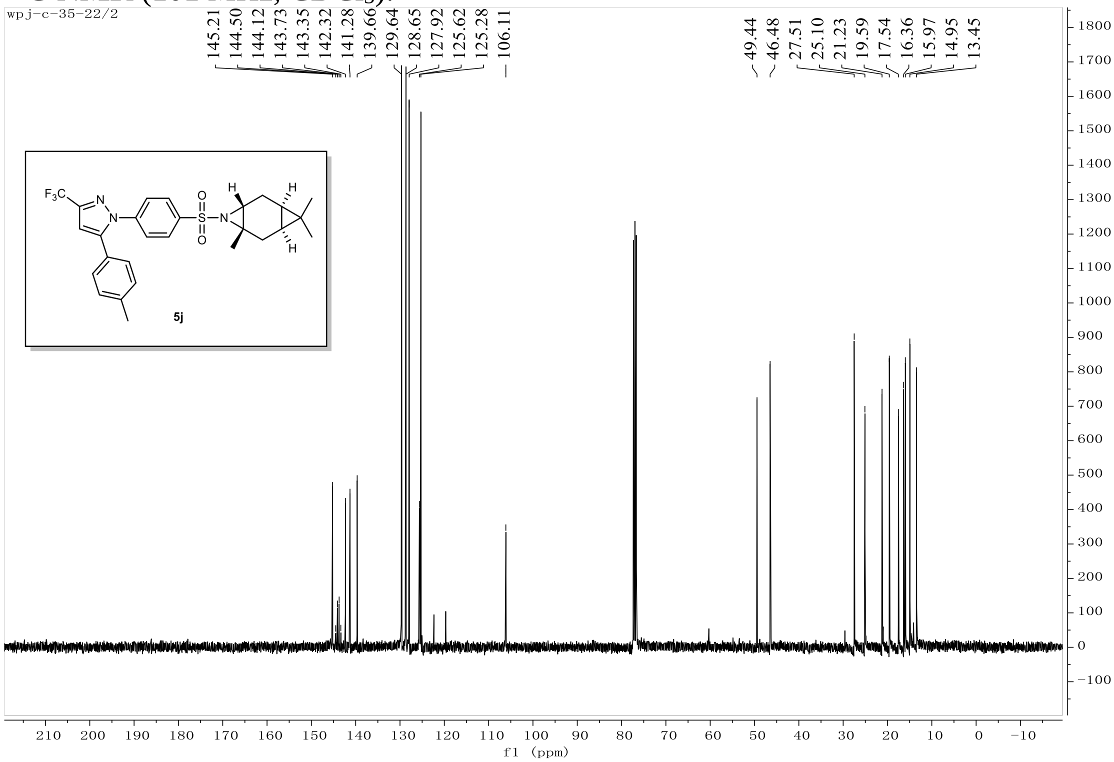

^{19}F NMR (377 MHz, $CDCl_3$):

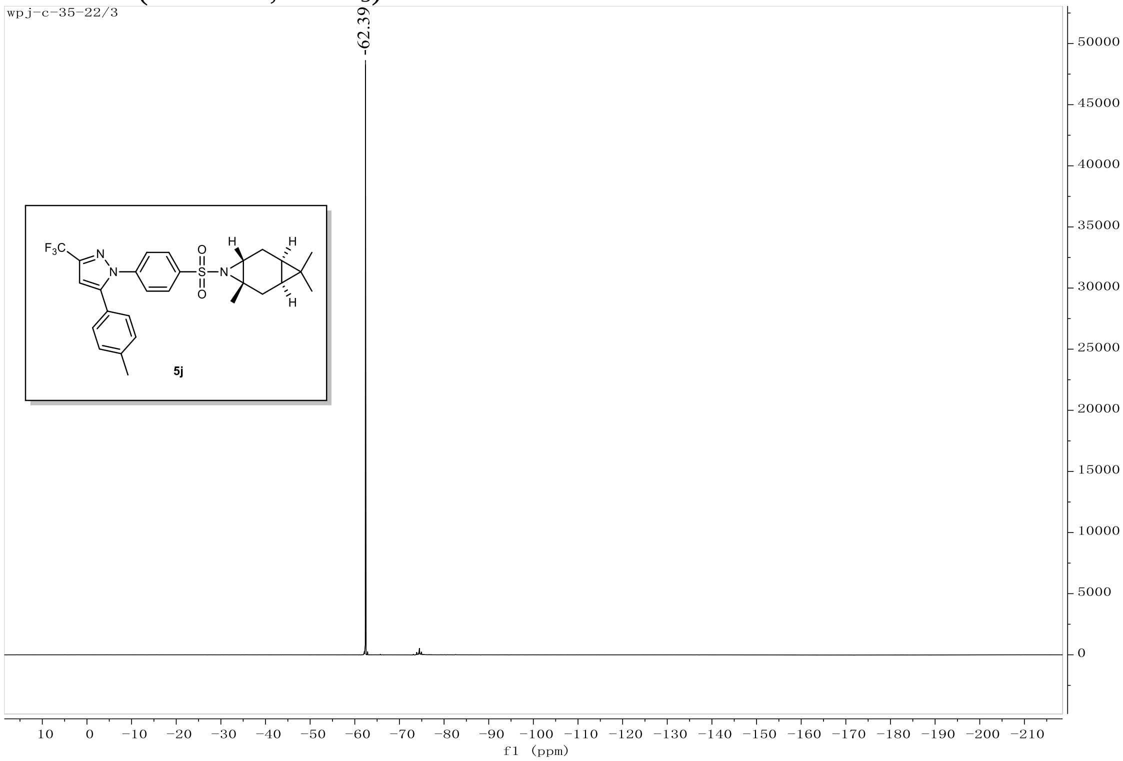

^{1}H NMR (400 MHz, $CDCl_3$):

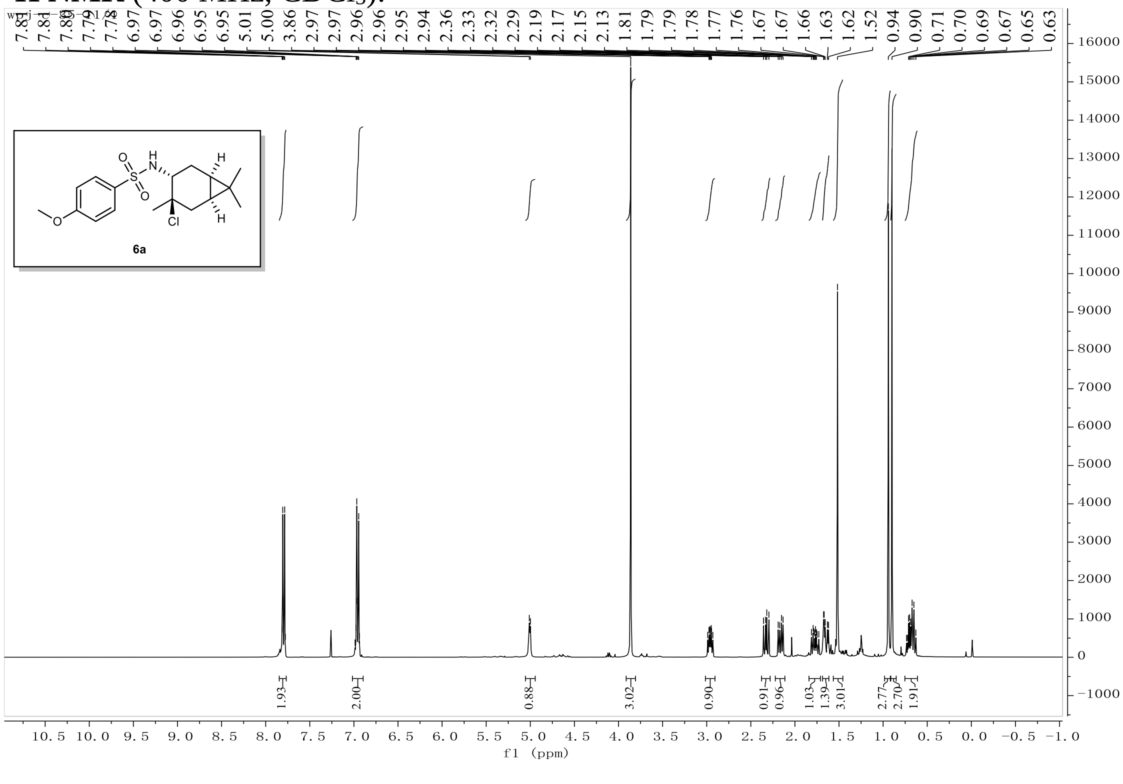

^{13}C NMR (101 MHz, $CDCl_3$):

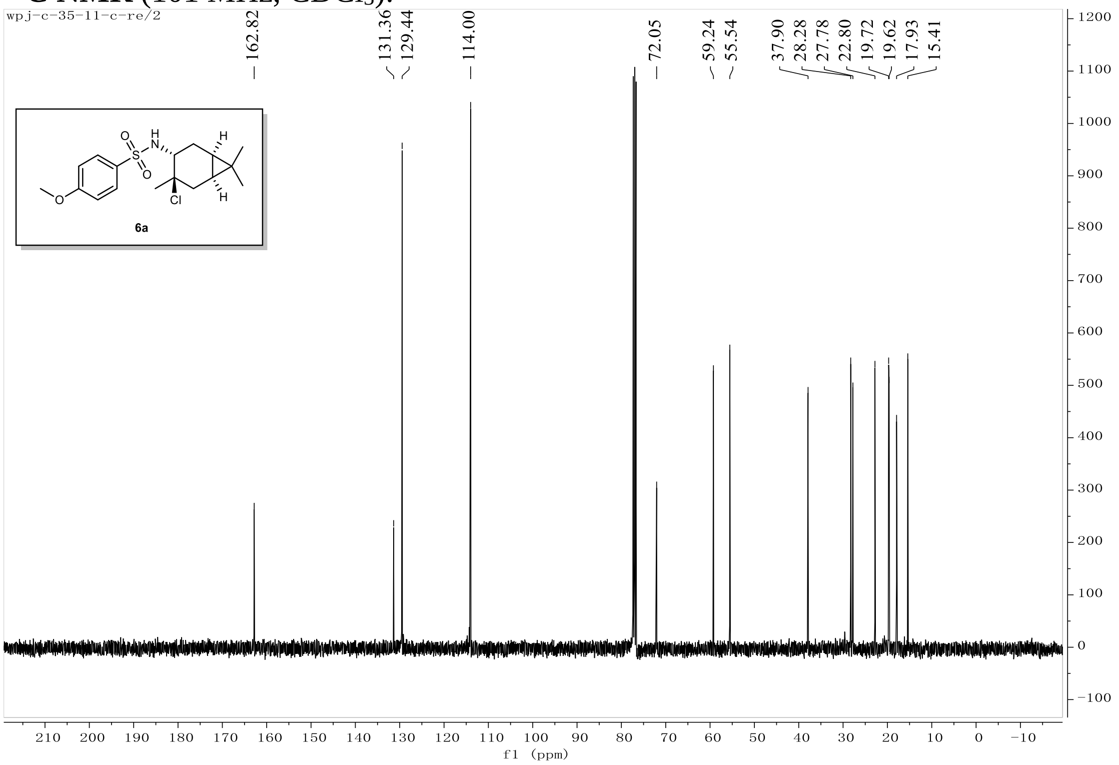

^{1}H NMR (400 MHz, $CDCl_3$):

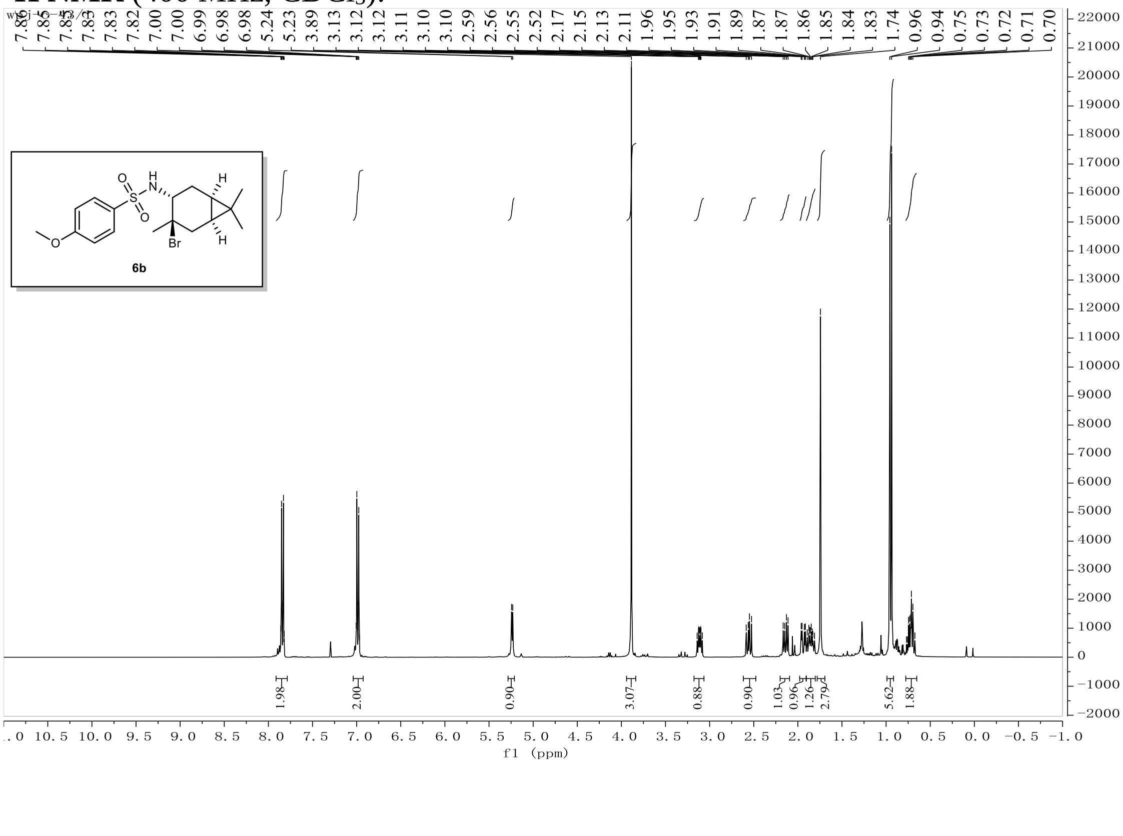

^{13}C NMR (101 MHz, $CDCl_3$):

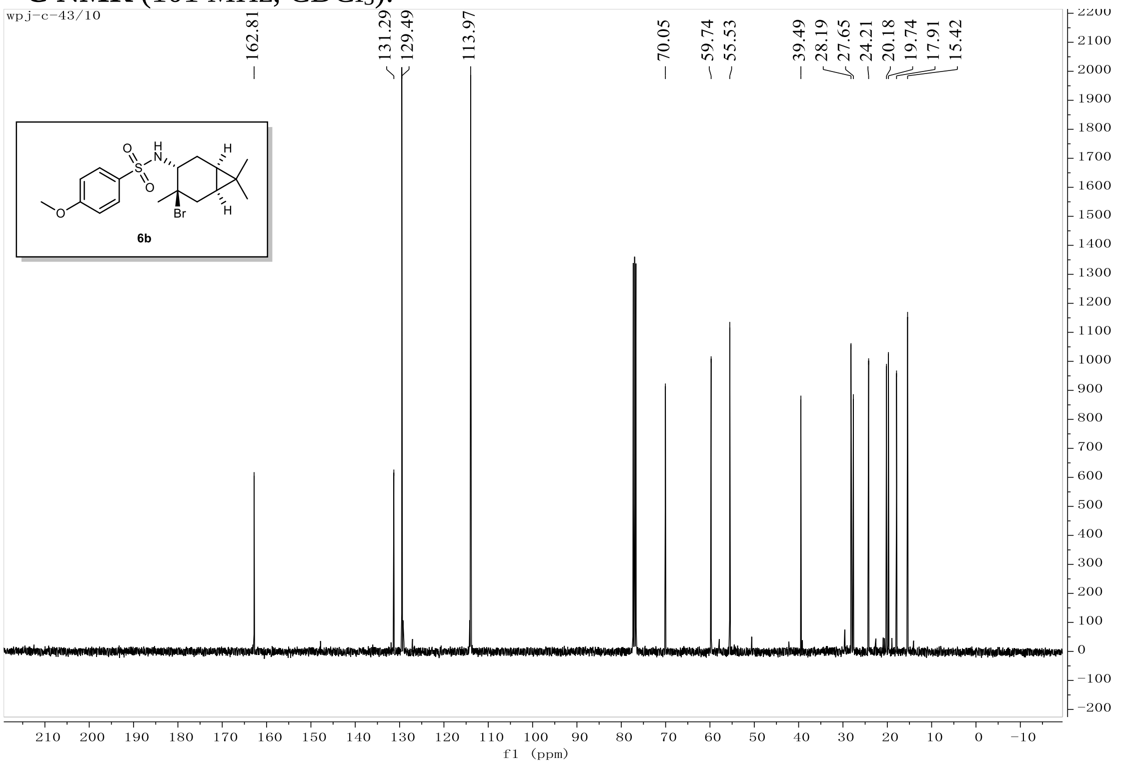

^{1}H NMR (400 MHz, $CDCl_3$):

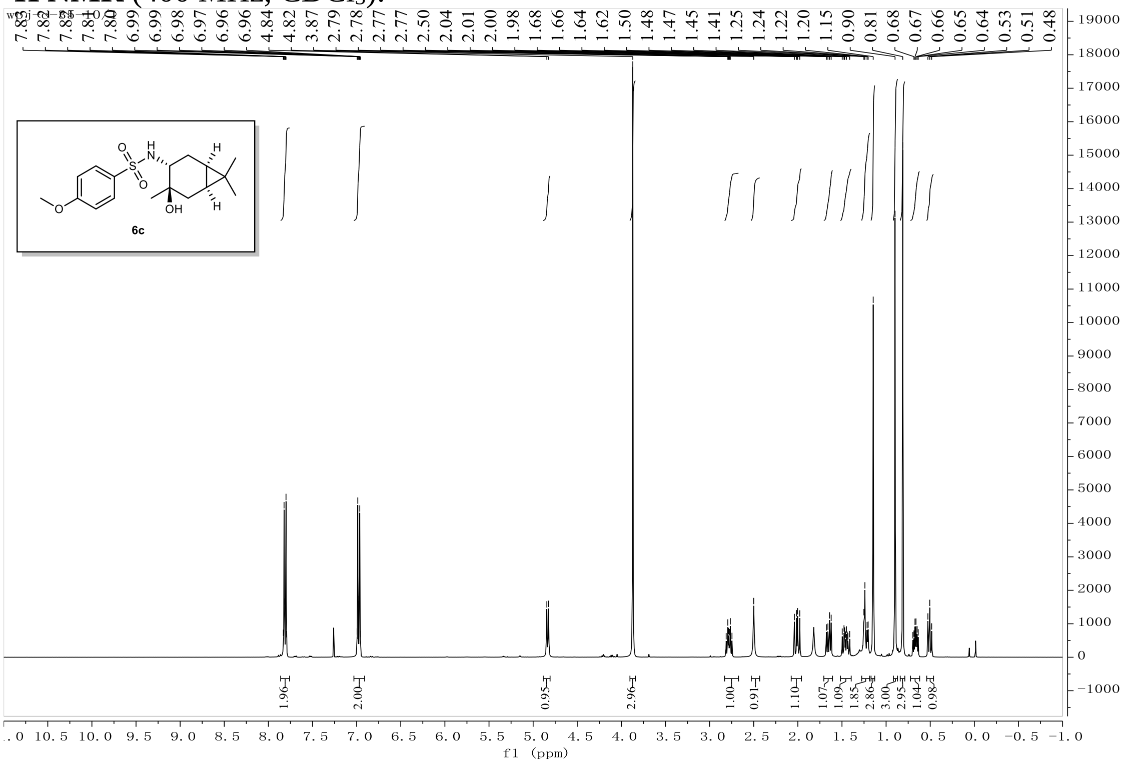

^{13}C NMR (101 MHz, $CDCl_3$):

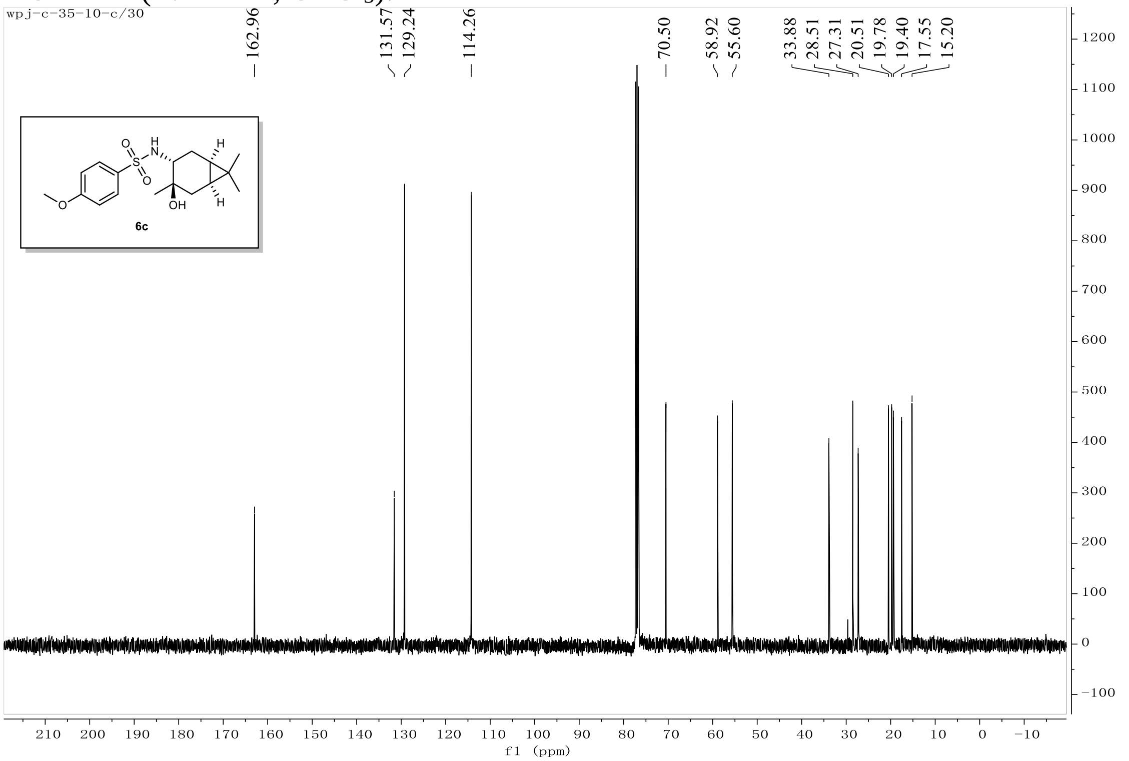

^{1}H NMR (400 MHz, $CDCl_3$):

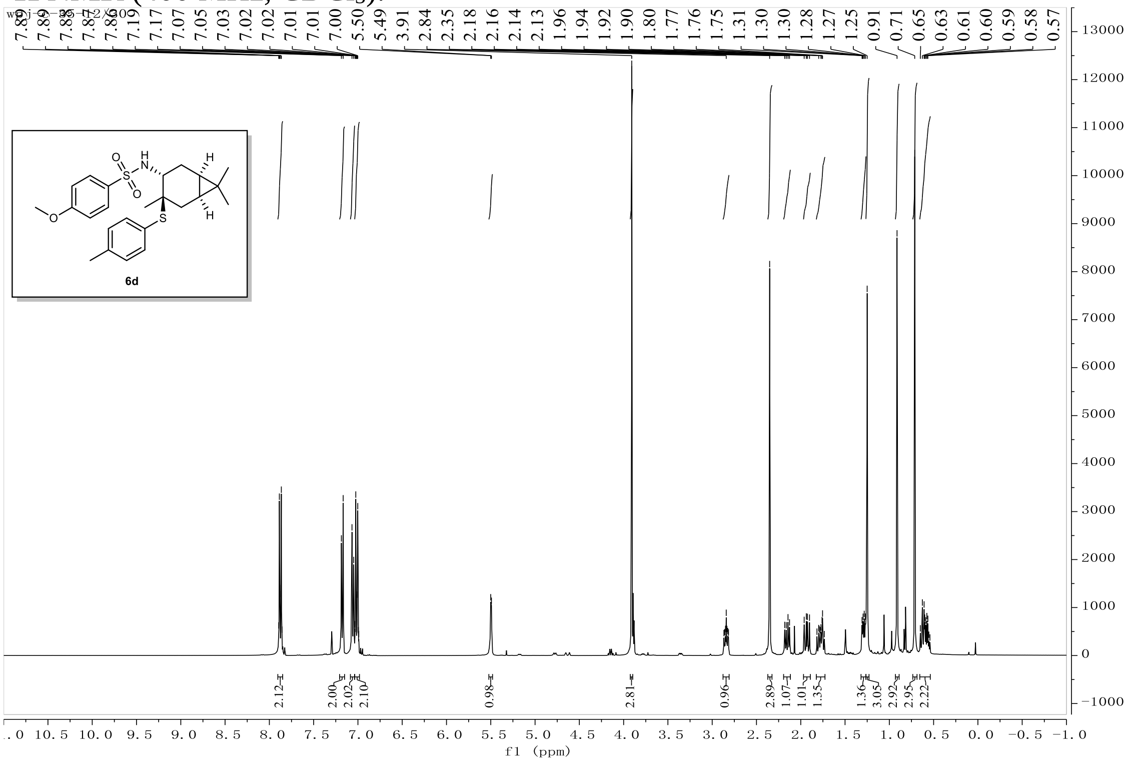

¹³C NMR (101 MHz, $CDCl_3$):

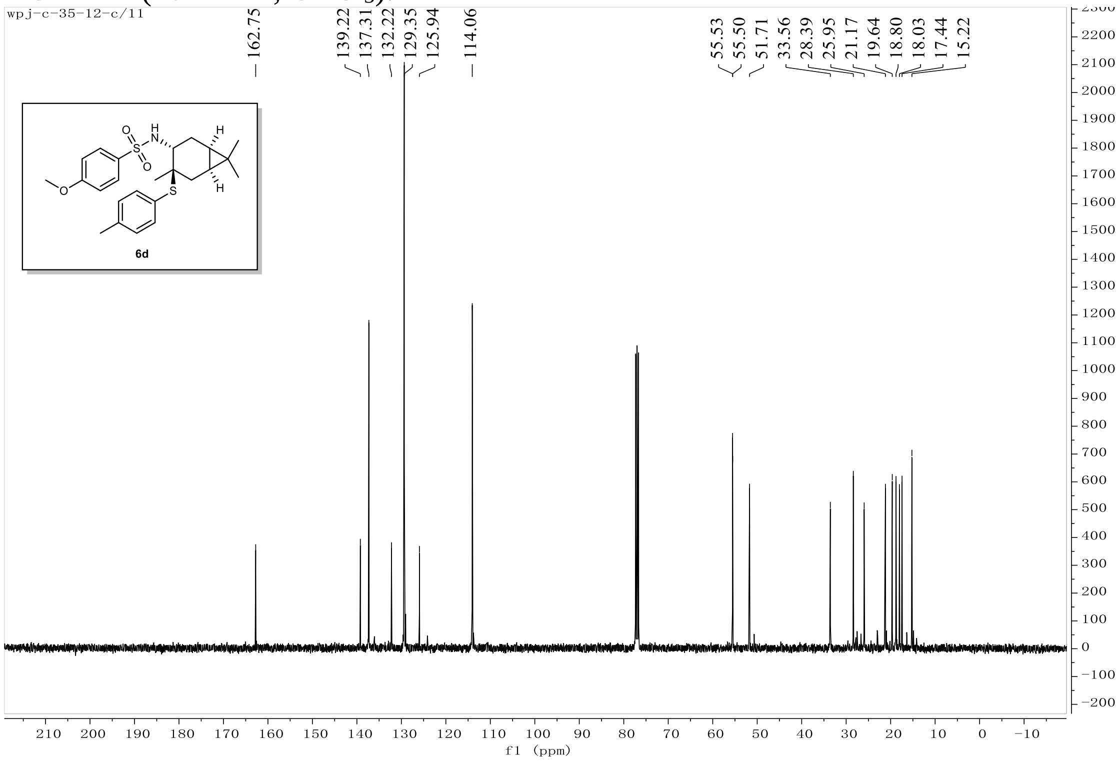

¹H NMR (400 MHz, $CDCl_3$):

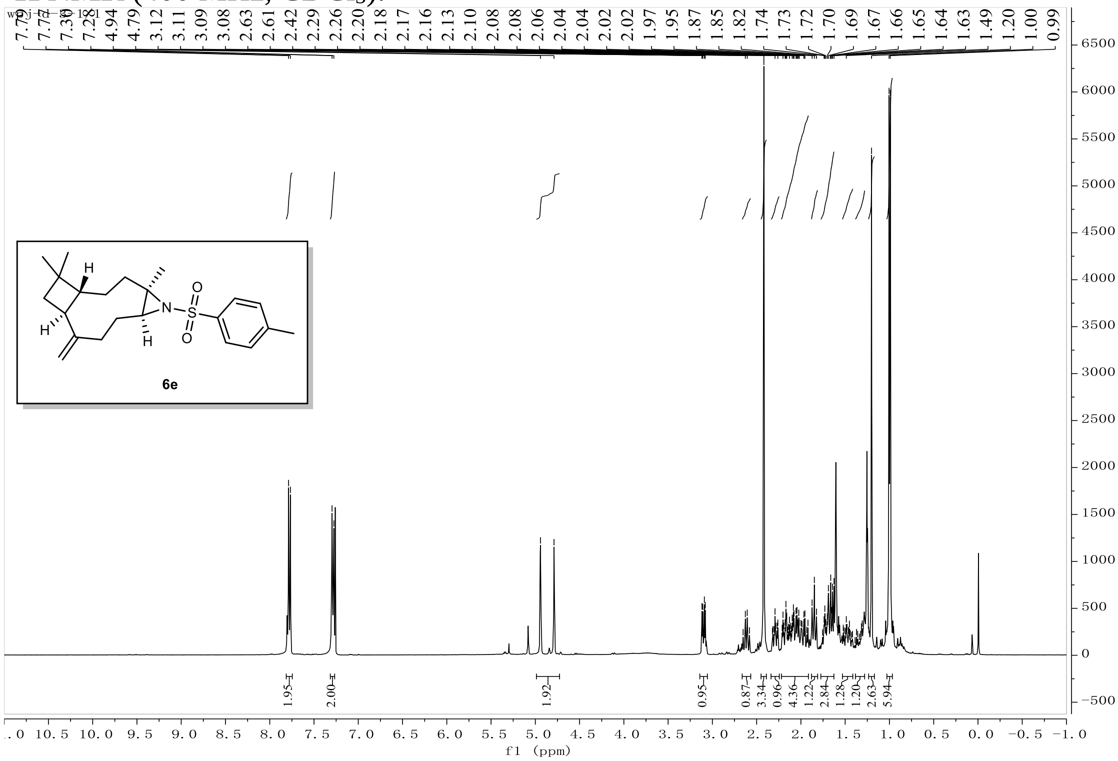

^{13}C NMR (101 MHz, $CDCl_3$):

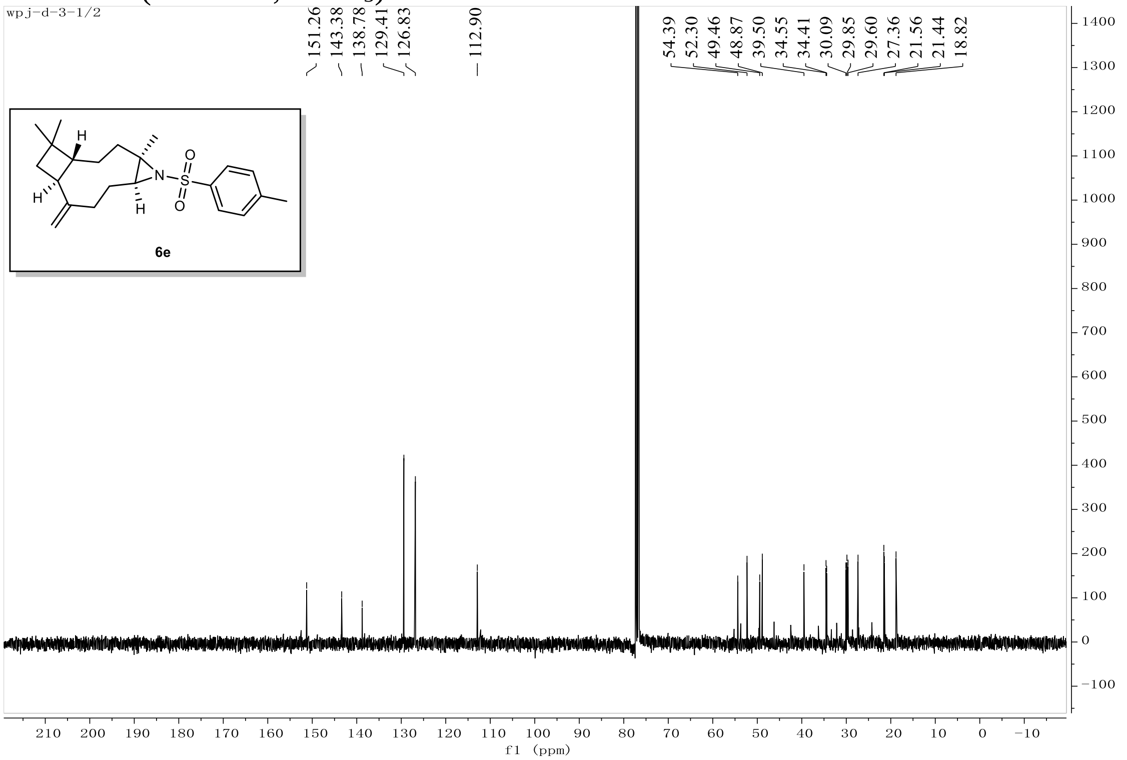

^{1}H NMR (400 MHz, $CDCl_3$):

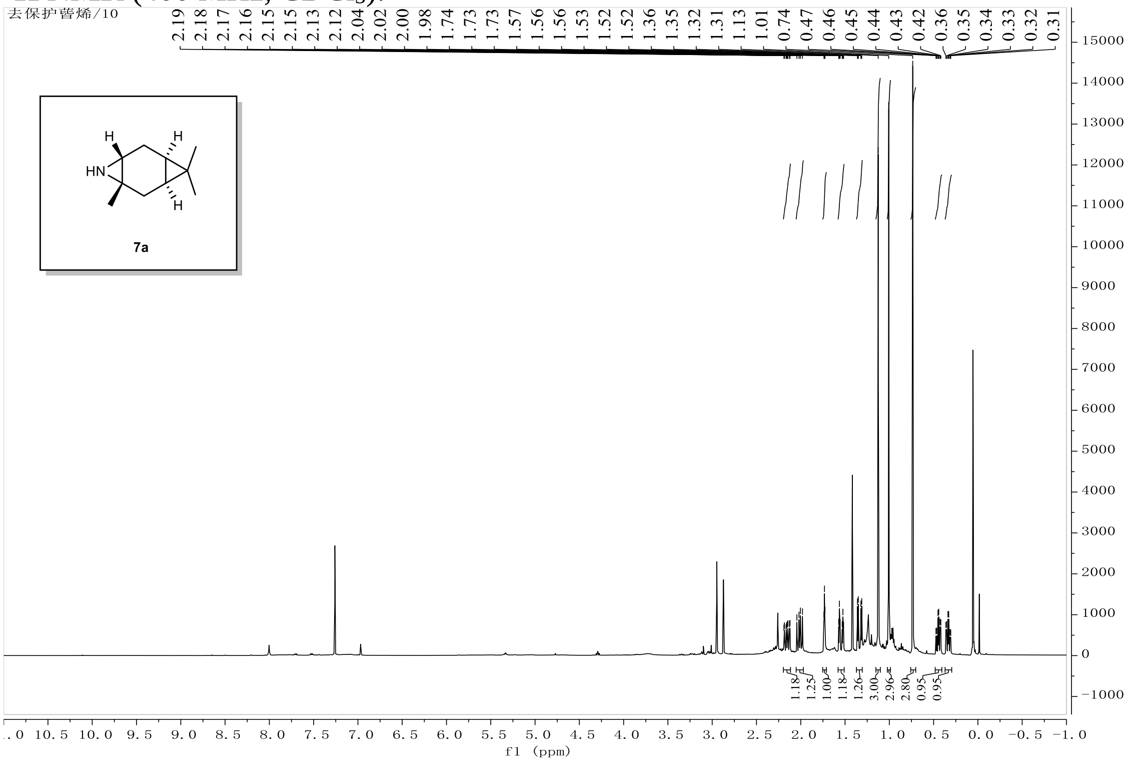

^{13}C NMR (101 MHz, $CDCl_3$):

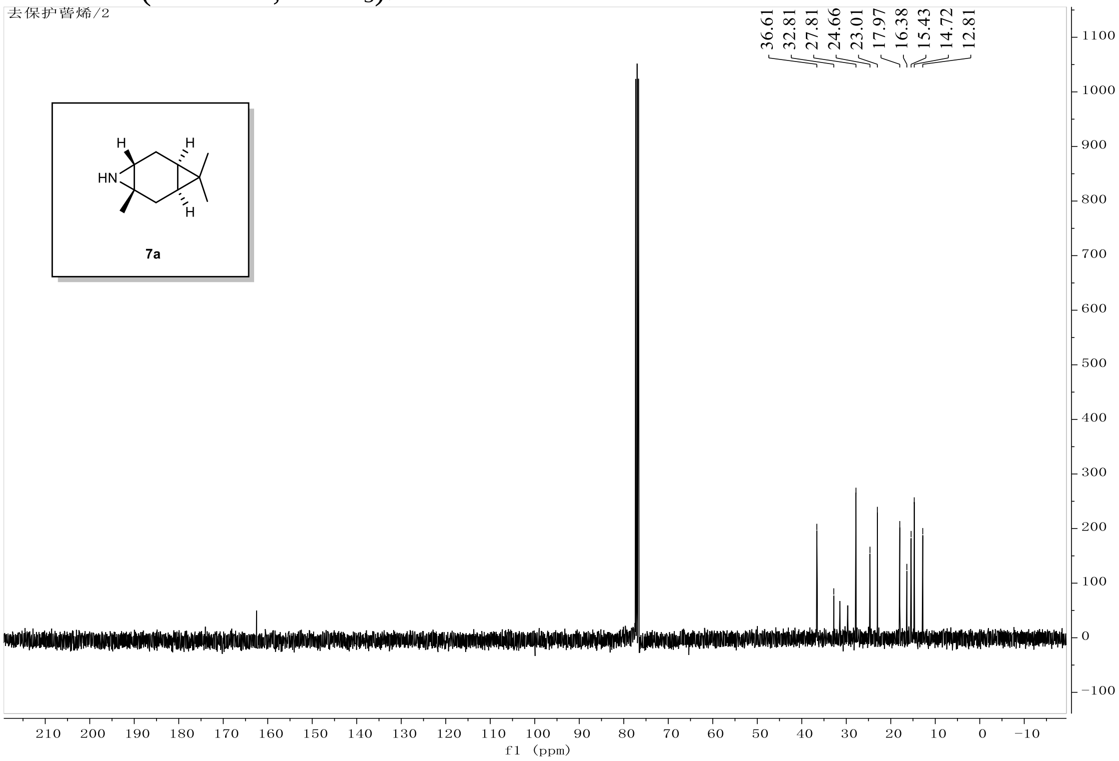

^{1}H NMR (400 MHz, $CDCl_3$):

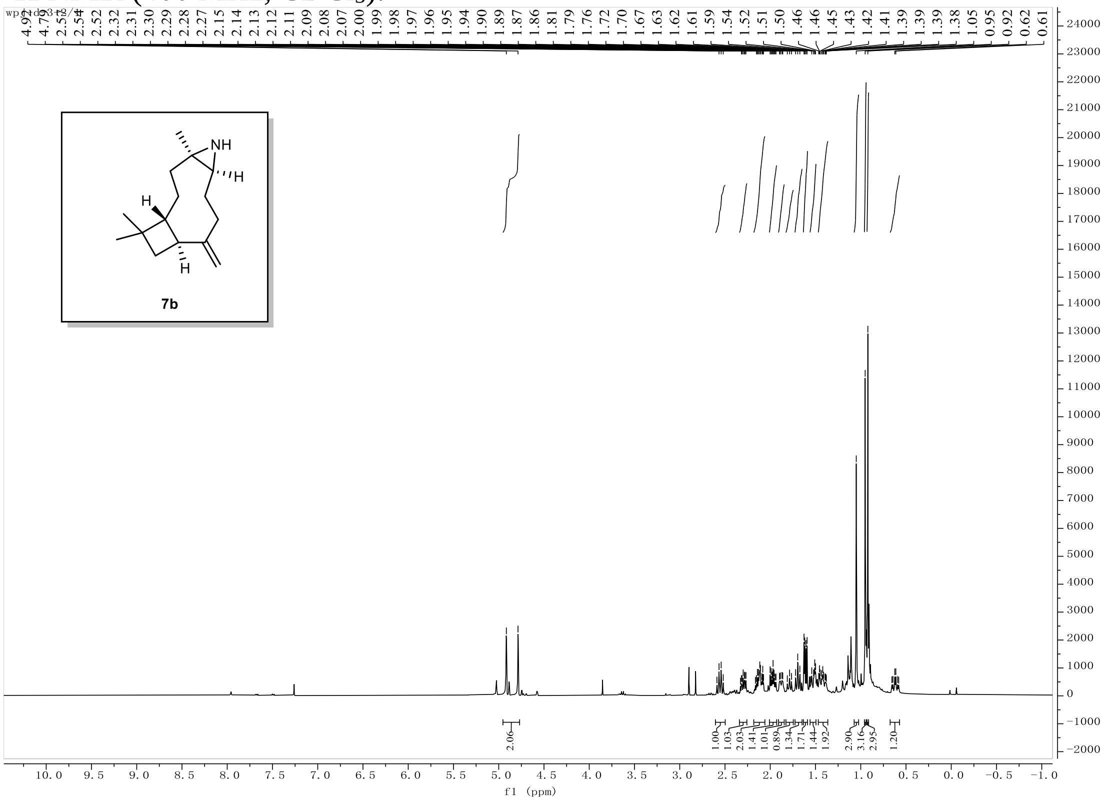

^{13}C NMR (101 MHz, $CDCl_3$):

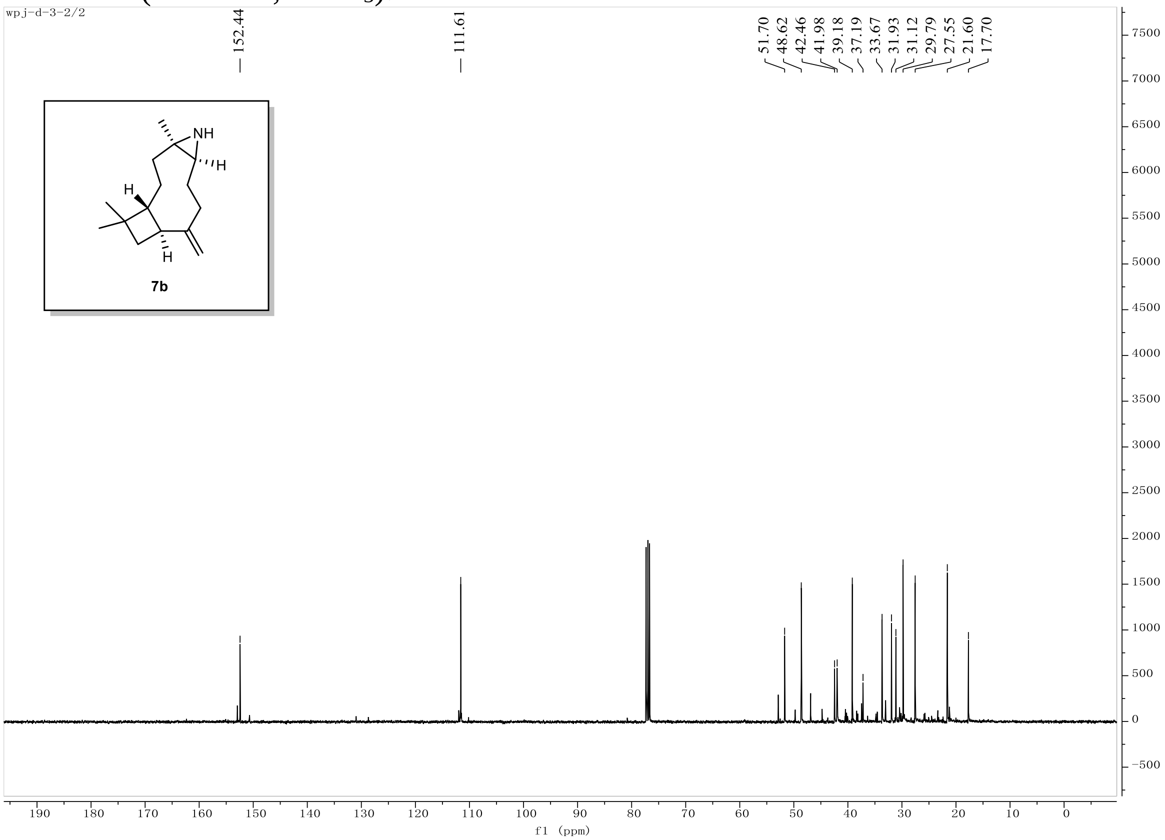

References

1 Wang, D., Wang, P., Wang, S. *et al.*, Direct electrochemical oxidation of alcohols with hydrogen evolution in continuous-flow reactor. *Nat. Commun.* **10**, 2796-2803 (2019).

2 Urban M., Sarek J., Klinot J., Korinkova G. *et al.*, Synthesis of A-seco derivatives of betulinic acid with cytotoxic activity. *J. Nat. Prod.* **67**, 1100-1105 (2004)

3 Thibeault, D., Gauthier, C., Legault, J. *et al.*, Synthesis and structure–activity relationship study of cytotoxic germanicane- and lupane-type 3*β*-*O*-monodesmosidic saponins starting from betulin. *Bioorg. Med. Chem.* **15**, 6144–6157 (2007).

4 Deng, T., Mazumdar, W., Yoshinaga, Y. *et al.*, Rh_2(II)-catalyzed cntermolecular *N*-aryl aziridination of olefins using nonactivated N atom precursors. *J. Am. Chem. Soc.* **143**, 19149−19159 (2021).

5 Yu, Y., Li, M., Zhang, Y. *et al.*, Construction of *N*-alkyl- and *N*-arylaziridines from unprotected amines via C−H oxidative amination strategy. *Org. Lett.* **21**, 904−907, (2019).

6 Ghorai, M. K., Shukla, D., & Bhattacharyya, A. Syntheses of chiral *β*- and *γ*-amino ethers, morpholines, and their homologues via nucleophilic ring-opening of chiral activated aziridines and azetidines. *J. Org. Chem.* **77**, 3740−3753 (2012).

7 Senboku, H., Nakahara, K., Fukuhara, T. *et al.*, Hg cathode-free electrochemical detosylation of *N*,*N*-disubstituted *p*-toluenesulfonamides: mild, effificient, and selective removal of *N*-tosyl group. *Tetrahedron Lett.* **51**, 435–438 (2010).

8 Ma, Z., Zhou, Z. & Kürti, L. Direct and stereospecific synthesis of N-H and N-alkyl aziridines from unactivated olefins using hydroxylamine-O-sulfonic acids. *Angew. Chem. Int. Ed.* **56**, 9886-9890, (2017).

9 Siu, T. & Yudin, A. K. Practical olefin aziridination with a broad substrate scope. *J. Am. Chem. Soc.* **124**, 530-531, (2002).

10 Siu, T., Picard, C. J. & Yudin, A. K. Development of electrochemical processes for nitrene generation and transfer. *J. Org. Chem.* **70**, 932-937, (2005).
